# Bridging the incompatibility gap in dual asymmetric catalysis over a thermoresponsive hydrogel-supported catalyst

**DOI:** 10.1038/s42004-023-01085-z

**Published:** 2024-01-03

**Authors:** Renfu Huang, Shoujin Yang, Zhipeng Hu, Bangtai Peng, Yuanli Zhu, Tanyu Cheng, Guohua Liu

**Affiliations:** Key Laboratory of Resource Chemistry of Ministry of Education, Shanghai Key Laboratory of Rare Earth Functional Materials, Shanghai Normal University, No.100 Guilin Rd, Shanghai, China

**Keywords:** Heterogeneous catalysis, Asymmetric catalysis, Synthetic chemistry methodology

## Abstract

The integration of dual asymmetric catalysis is highly beneficial for the synthesis of organic molecules with multiple stereocenters. However, two major issues that need to be addressed are the intrinsic deactivation of dual-species and the extrinsic conflict of reaction conditions. To overcome these concerns, we have utilized the compartmental and thermoresponsive properties of poly(*N*-isopropylacrylamide) (PNIPAM) to develop a cross-linked PNIPAM-hydrogel-supported bifunctional catalyst. This catalyst is designed with Rh(diene) species situated on the outer surface and Ru(diamine) species positioned within the interior of the hydrogel. The compartmental function of PNIPAM in the middle overcomes intrinsic mutual deactivations between the dual-species. The thermoresponsive nature of PNIPAM allows for precise control of catalytic pathways in resolving external conflicts by controlling the reaction switching between an Rh-catalyzed enantioselective 1,4-addition at 50°C and a Ru-catalyzed asymmetric transfer hydrogenation (ATH) at 25°C. As we envisioned, this sequential 1,4-addition/reduction dual enantioselective cascade reaction achieves a transformation from incompatibility to compatibility, resulting in direct access to γ-substituted cyclic alcohols with dual stereocenters in high yields and enantio/diastereoselectivities. Mechanistic investigation reveals a reversible temperature transition between 50°C and 25°C, ensuring a cascade process comprising a 1,4-addition followed by the ATH process.

## Abstract (web summary)

Dual asymmetric catalysis is powerful for the synthesis of multi-stereocenter molecules, however, the integration of dual catalysts remains challenging due to the intrinsic deactivation of dual-species and extrinsic conflict of reaction conditions. Here, the authors develop a compartmental and thermoresponsive poly(*N*-isopropylacrylamide)-hydrogel-supported bifunctional catalyst to overcome these challenges.

## Introduction

The development of dual catalysis using transition-metal-catalysts and/or organocatalysts in enantioselective cascade reactions has achieved significant progress due to their ability to construct chiral organic molecules with one to multiple stereocenters^1–4^. However, the intrinsic deactivation of dual chiral centers originating from cross-interactions and extrinsic conflicts of reaction conditions render most of these reactions under homogeneous conditions incompatible^3^, which could be well-elucidated in designing dual-catalytic enantioselective processes^5^. Site-isolated immobilization is a practical strategy that has been widely used to overcome mutual deactivation, and numerous site-isolated heterocatalysts have been developed for various enantioselective reactions^5–7^. Among the reported methods used for the fabrication of site-isolated chiral heterocatalysts^6^, polymeric immobilization has received extensive attention because it can match the homogeneous catalytic efficiency^8–15^. A pioneering work conducted by the Fréchet group has successfully integrated an arylsulfonic acid with a chiral imidazolidinone or chiral pyrrolidine within a star polymer to form a site-isolated catalyst, enabling a nucleophilic and Michael addition cascade reaction^10^. Another groundbreaking study reported by the McQuade group has used a polymeric encapsulation strategy to construct a site-isolated catalyst for the enantioselective addition process^11^. Recently, the Weck group has reported a series of polymer-based site-isolated catalysts using amphiphilic poly(2-oxazoline)-based cross-linked micelles, which enable the tandem process to chiral products from alkynes, alcohols, and chalcones^12–14^. Very recently, we have also attempted utilizing a composite material composed of inorganic silica and polymer, fabricating a site-isolated catalyst for enantioselective cascade reaction^15^. Despite these significant achievements, the use of the traditional polymer-supported site-isolated immobilization alone is still inadequate in solving the extrinsic reaction conflicts imparted on the subtle dual chiral environments due to the polymeric swelling nature. Therefore, a stimuli-responsive polymeric immobilization is needed to fabricate a site-isolated bifunctional catalyst, which not only overcomes the mutual deactivations of dual chiral species but also harmonizes the polymeric swelling behaviors, thereby possibly bridging the incompatibility gap in a dual asymmetric catalysis process.

Thermoresponsive polymers, particularly thermoresponsive polymeric hydrogels, as an important subclass of stimuli-responsive materials, have exhibited an intriguing property during a phase transition between the solution and hydrogel forms^16–18^. This property has been successfully utilized in single-step enantioselective Aldol and asymmetric desymmetrization reactions^19–22^, and some supramolecular gels have also exhibited fascinating enantioselective organic transformations^23–27^. In particular, core-shell-structured thermoresponsive polymeric hydrogels are particularly advantageous as they can easily form compartmentalization, which provides a unique opportunity for creating a site-isolated bifunctional catalyst^28–30^. For example, the polymeric compartmental inner and outer layers benefit an isolated immobilization, thereby eliminating cross-interactions of dual-species. Furthermore, the thermoresponsive polymeric intermediate layer of hydrogel enables a reversible phase transition and/or controllable dispersive situation to adjust reaction conditions, thereby overcoming negative interferences imparted on dual chiral environments. Despite the potential superiority, the fabrication of a thermoresponsive hydrogel-supported site-isolated bifunctional catalyst for dual asymmetric catalysis remains unexplored.

**Fig. 1:**
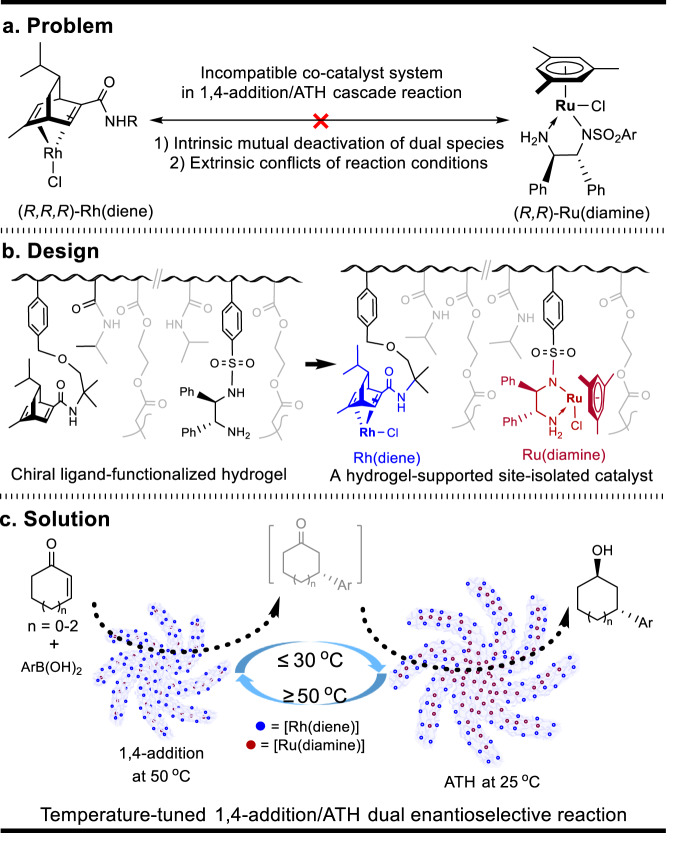
A schematic illustration of the problem, design, and solution. **a** General problems of the mutual deactivation between Rh(diene) and Ru(diamine) and the reaction conflict used in a homogeneous dual-catalysis system. **b** Design of a cross-linked PNIPAM-hydrogel-supported site-isolated bifunctional catalyst. **c** Solution of a temperature-tuned 1,4-addition/ATH dual enantioselective reaction *via* the reversibly thermoresponsive transition of the catalyst.

Chiral Rh(diene)-complexes as a kind of important 1,4-addition catalysts are well-known for the enantioselective reactions of ɑ,ß-unsaturated ketones^31–33^, whereas chiral Ru(diamine)-complexes commonly function as asymmetric transfer hydrogenation (ATH) catalysts for the enantioselective reduction of prochiral ketones^34,35^. In theory, combining these two types of catalysts through a cascade process could be used to synthesize chiral alcohols with two or more stereocenters^36–40^. However, direct dual asymmetric catalysis under homogeneous conditions *via* a 1,4-addition/reduction cascade reaction to prepare γ-substituted cyclic alcohols with dual stereocenters is infeasible due to the problem of intrinsic deactivation and extrinsic reaction conflict (Fig. 1a). Inspired by an elegant phototriggered strategy that utilizes a reversible spiropyran to merocyanine transition on a micelle for the preparation of alcohols from chalcones^12^, the exploration of a thermochemical strategy in the elimination of the above conflicts is particularly attractive. This strategy can complement the synthetic limitation for the preparation of γ-substituted cyclic alcohols in a single-step reaction^41,42^. More importantly, this strategy also offers a cascade method to directly synthesize these cyclic alcohols from simple starting materials. As illustrated in Fig. 1b, we propose that a thermoresponsive polymer-based chiral ligand-functionalized hydrogel can fulfill the requirements of a two-step sequential transformation and design a cross-linked PNIPAM-hydrogel-supported site-isolated catalyst. This bifunctional catalyst contains the Rh(diene) species (blue) situated on the outer surface and Ru(diamine) species entrapped within the interior of the hydrogel. The specificity lies in the compartmental and thermoresponsive functions of the poly(*N*-isopropylacrylamide) (PNIPAM) in the middle^43,44^. The compartmental function acts as a shield to completely separate the two species, thereby overcoming the intrinsic mutual deactivation between Rh(diene) and Ru(diamine). The thermoresponsive function creates an on-and-off mode to manipulate the reaction sequence through the temperature-controlled reaction switching between 50 °C and 25 °C, thereby solving extrinsic conflicts of reaction conditions. As a result, this method bridges the incompatibility gap and makes an infeasible 1,4-addition/reduction cascade reaction under homogeneous conditions possible. As we envisioned (Fig. 1c), this bifunctional catalyst enables a temperature-tuned 1,4-addition/reduction dual enantioselective process, directly accessing 1,3-distereocentered γ-substituted cyclic alcohols from the reaction of cyclic enones and arylboronic acids in 73-97% yields, 91–99% *ee* and up to 99:1 *dr*.

## Results and discussion

### Synthesis and characterizations of the heterogeneous catalyst

**Fig. 2:**
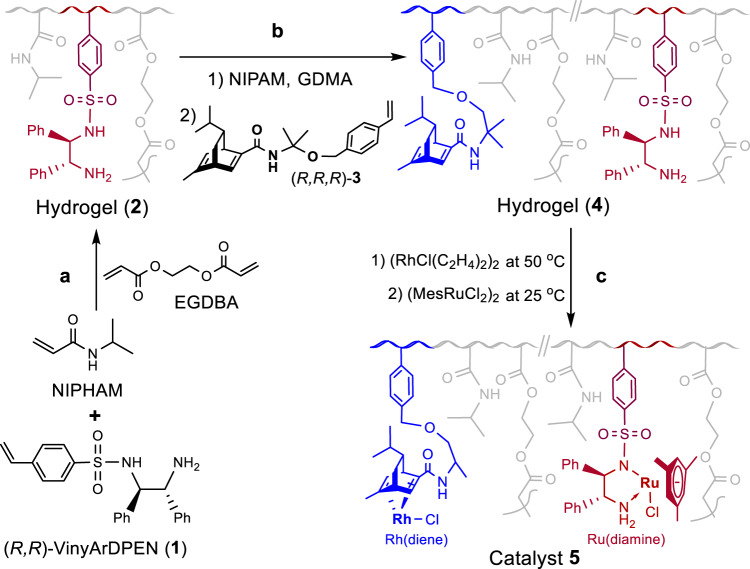
Synthesis of catalyst 5. **a** An emulsion copolymerization step. **b** A precipitation polymerization step. **c** Coordinations step.

Figure 2 illustrated the use of a seeded precipitation polymerization procedure to prepare a thermoresponsive cross-linked PNIPAM-supported Rh(diene)- and Ru(diamine)-functionalized catalyst (**5**) (diamine (VinyArDPEN)^45,46^: *N*-((1 *R*,2 *R*)-2-amino-1,2-diphenylethyl)-4-vinylbenzenesulfonamide (**1**), diene^34,47–51^: (1 *R*,4 *R*,7 *R*)-7-isopropyl-5-methyl-N-(2-methyl-1-((4-vinylbenzyl)oxy)propan-2-yl)bicyclo[2.2.2]octa-2,5-diene-2-carboxamide (**3**)) (Supplementary Experimental part and Fig. S1)^19^. Firstly, an emulsion copolymerization of *N*-isopropylacrylamide (NIPAM) and **1** was carried out using ethane-1,2-diyl bis(2-methylacrylate) (EGDMA) as a cross-linker reagent to produce the corresponding hydrogel. Purification through the use of the dialysis against pure water gave the pure **2** with chiral diamine ligands surrounded by a cross-linked PNIPAM, in which the molar ratio of NIPAM/EGDMA/diamine was 23/1.8/1 evaluated by ^1^H NMR spectroscopy (Supplementary Fig. S2). Upon comparing the initial molar ratio of the feed NIPAM/EGDMA/diamine, we observed a significant variation in the molar ratio of the NIPAM/EGDMA/diamine of the hydrogel (**2**). This observation illustrated the efficacy of the dialysis process used for purifying the resulting coarse polymers. It efficiently eliminated unexpected polymeric by-products that arose from the self-polymerization of NIPAM or monomer **1**. Furthermore, the analysis conducted through gel permeation chromatography (GPC) also revealed that the hydrogel (**2**) exhibited a molecular weight (Mw) of 4.63 kDa with a relatively narrow polydispersity index (PDI) of 1.19 at 25 °C and broad PDI (1.46) at 50 °C. Secondly, further precipitation polymerization was performed using **2** and NIPAM followed by the addition of **3** to give hydrogel (**4**) *via* the purification with the dialysis against pure water, in which the molar ratio of NIPAM/EGDMA/diamine/diene was 143/10/2/1 evaluated by ^1^H NMR spectroscopy (Supplementary Fig. S2)^17^. Upon comparing the molar ratio of NIPAM/EGDMA/diamine (12.8/1) in the hydrogel (**2**) with the ratio of 14.3/1 in the hydrogel (**4**), the increase in the molar ratio could be attributed to the additional NIPAM introduced during the second-step polymerization process. Notably, it was worth mentioning that even a small quantity of ligands incorporated into the hydrogel (**4**) sufficed for the subsequent construction of a bifunctional catalyst (**5**). Furthermore, the purification of the resulting coarse polymer through dialysis yielded a desirable hydrogel (**4**) with a molecular weight (Mw) of 5.82 kDa and a polydispersity index (PDI) of 1.31 at 25 °C that did not exhibit significant changes at 50 °C (1.29) detected by GPC analysis. Finally, hydrogel **4** was subjected to coordination with (RhCl(C_2_H_4_)_2_)_2_ at 50 °C to produce **4’**, followed by coordination with (MesRuCl_2_)_2_ at 25 °C to form the coarse catalyst, which was purified through the Soxhlet extraction in CH_2_Cl_2_ solvent to remove the unreacted Rh- and Ru-complexes and afforded the pure site-isolated bifunctional catalyst **5**.

**Fig. 3:**
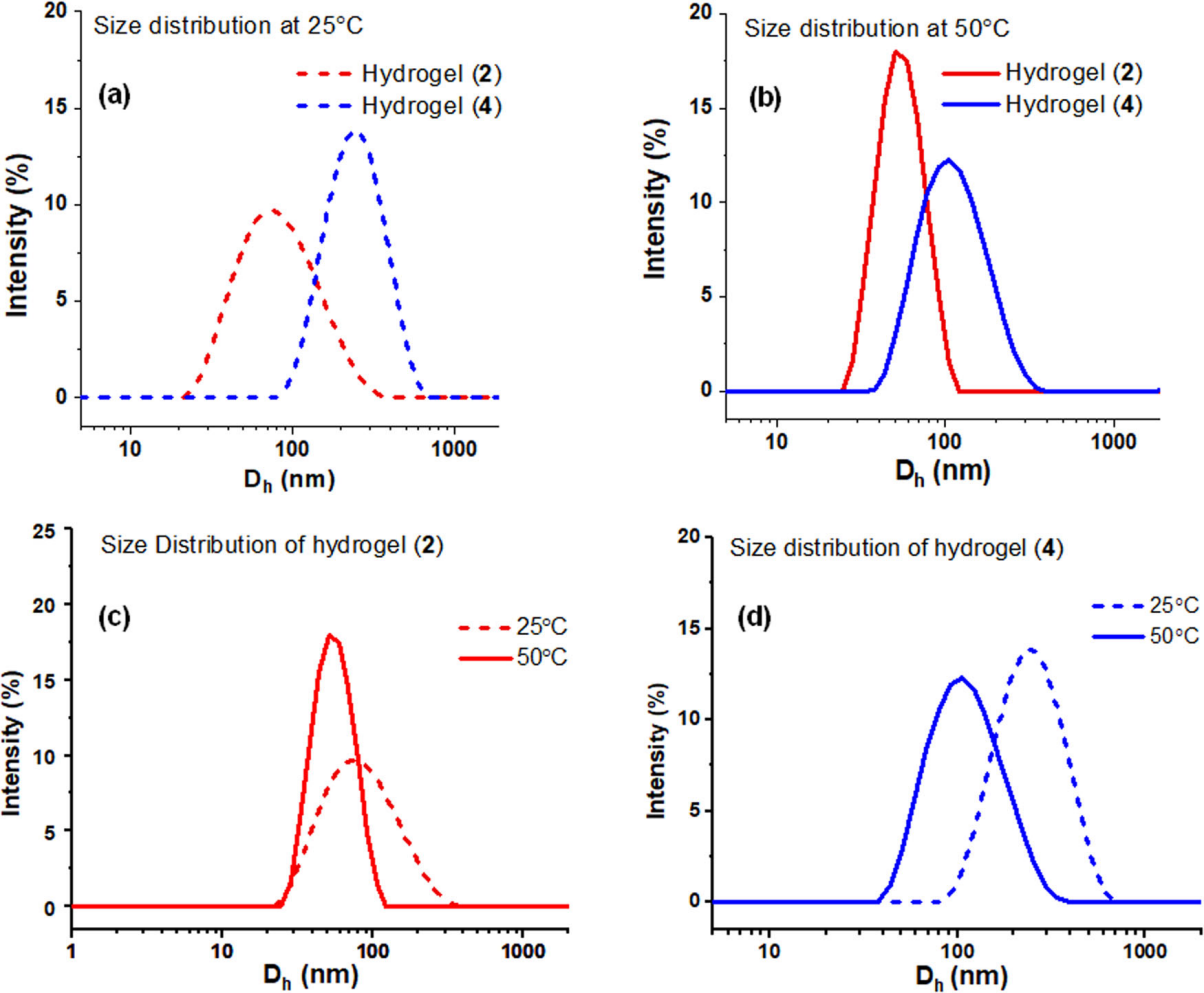
DLS traces of the hydrophobic hydrogels. **a** Hydrogels (**2**) and (**4**) indicated at 25 °C (red dotted trace for **2** and blue dotted trace for **4**). **b** Hydrogels (**2**) and (**4**) indicated at 50 °C (red trace for **2** and blue trace for **4**). **c** Hydrogel (**2**) indicated both at 25 °C (red dotted trace) and at 50 °C (red trace). **d** Hydrogel (**4**) indicated both at 25 °C (blue dotted trace) and at 50 °C (blue trace).

To clarify this synthetic process, dynamic laser scattering (DLS) investigations were performed, as shown in Fig. 3. We found that the hydrogel (**4**), at both 25 °C and 50 °C, exhibited increased hydrodynamic diameters (D_h_) when compared to the hydrogel (**2**). This phenomenon indicated that the enhanced D_h_ can be attributed to the two-step continuous polymerization procedure (Fig. 3a). It was notable that the hydrogel (**4**) displayed a distinct swelling and shrinking behavior between 25 °C and 50 °C, as evidenced by the variations in D_h_ values, while the hydrogel (**2**) did not exhibit significant changes (Fig. 3b). This discovery implies the presence of an efficient on-and-off mode employed to control the catalytic behavior of chiral Ru(diamine) within hydrogel (**2**), facilitating a temperature-tuned 1,4-addition/ATH cascade process discussed below. Furthermore, a turbidity measurement by analyzing the first derivative of the temperature-dependent transmittance decrease revealed that the volume phase transition temperature (VPTT) of hydrogel (**4**) was 30°C in a 0.02 M concentration of pure water (Supplementary Fig. S3a, S3b). Interestingly, a visible hydrogel formation and phase transition of **4** in water were also observed. The solution exhibited viscosity changes, becoming progressively more viscous from 25°C to 50°C, and ultimately transitioning into a hydrogel state from 50°C to 80°C (Supplementary Fig. S3c). For comparison purposes, a parallel analog (**5’**) was also synthesized through the direct coordination of **2** and (MesRuCl_2_)_2_ using a similar procedure.

**Fig. 4:**
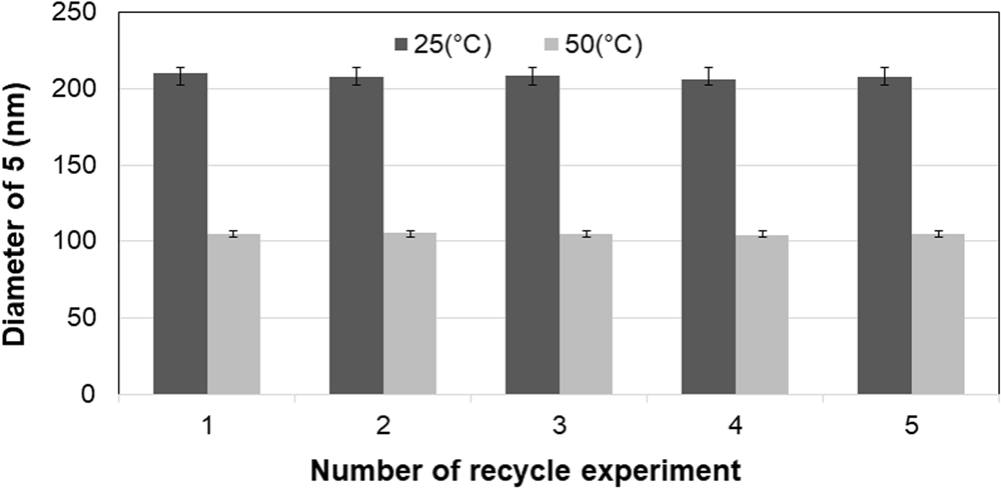
Investigation of average hydrodynamic diameter distribution. Note. Black for hydrodynamic diameters of catalyst **5** indicated at 25 °C and gray for hydrodynamic diameters of catalyst **5** indicated at 50 °C. The error bars represent the standard deviation.

Despite the absence of the hydrogel form observed in catalyst **5** when placed in water, the different dispersive behaviors in the reaction system (H_2_O/dioxane, v-v = 2:5) at 25 °C and 50 °C could be clearly observed (Supplementary Fig. S4). In addition, the scanning electron microscopy (SEM) images for the cross-linked PNIPAM-based morphology of catalyst **5** revealed a greater degree of cross-linking accumulation at 50 °C compared to 25 °C, whereas the viscosity in the rheological analysis with a significant increase between 4.5 mg/mL and 6.5 mg/mL in the H_2_O/dioxane mixture (v:v = 2/5) at 50 °C compared to 25 °C was consistent with the conditions of the 5.55 mg/mL concentration used in the reaction system (Supplementary Fig. S5). Furthermore, the DLS-based investigation of the hydrodynamic diameter (D_h_) distribution of catalyst **5** within the reaction system (H_2_O/dioxane, v-v = 2:5) revealed consistent patterns of swelling and shrinking transformations. As shown in Fig. 4, over five consecutive runs, the average D_h_ of **5** at 50 °C fluctuated around 104 nm, whereas that at 25 °C was around 207 nm (Supplementary Fig. S6). This observation confirmed an expected on-and-off mode originating from the swelling and shrinking forms within the reaction system, indicating the feasibility of controlling the cascade process.

Solid-state ^13^C CP/MAS NMR spectroscopy confirmed a successful immobilization of dual chiral centers in **5** as shown in Table 1 (Supplementary Fig. S7). Generally, the strong carbon signals were observed around δ = 22, 41, 68, 127, and 175 ppm, which corresponded to the alkyl-skeleton, alkoxyl, aryl carbon atoms, and carbonyl carbon atoms in the polymeric skeleton of **5**^15,52^. In the case of the Rh(diene)-complex unit, the weak characteristic carbon signals around δ = 53 and 32 ppm for the cyclic alkyl carbon atoms could be clearly observed. The other signals for the chain isopropyl, methyl, and carbonyl carbon atoms were unclear due to the overlapping of the skeleton carbon atoms of the polymer, but their enhanced intensities suggested efficient immobilization of the chiral Rh(diene)-complexes. These observed and overlapped characteristic carbon signals demonstrated a well-defined single-site center because their chemical shifts were similar to the parent counterpart^47,48^. In the case of Ru(diamine)-complex unit, the weak characteristic signals around δ = 76 and 71 ppm were attributed to the alkyl carbon atoms of N*C*HPh groups in the diamine moiety, and those around δ = 105 and 101 ppm ascribed the aryl carbon atoms of the mesitylene, in which their chemical shifts were the same as its parent MesRuTsDPEN species^45^. All of these observations demonstrated that the synthetic procedure was successful in forming the expected bifunctional catalyst with maintainable original chiral environments.

**Table 1:** Solid-state ^13^C NMR spectra for catalyst 5 with four fragments (**a**-**o** indicated the peaks of different carbon atoms in two chiral centers, and **a’**-**i’** indicated the peaks of the different carbon atoms in the polymeric skeleton).


^13^C-NMR	Peaks	Chemical shift and explanation
Carbon atom signals in skeleton	**a’** and **b'**	16.9 − 25.2 ppm.
	**c'**	25.9 − 34.5 ppm.
	**d'**	35.3-49.4 ppm.
	**e’**	65.3 − 69.9 ppm.
	**f’** and **g'**	113.9 − 153.6 ppm.
	**h’** and **i'**	170.9 − 179.6 ppm.
Carbon atom signals in Rh(diene) center	**a**	13.9 ppm.
	**b** and **c**	Overlapping by the skeleton C-atoms of **a’,** **b’**, and **c’**.
	**d** and **e**	32.2 − 34.6 ppm.
	**f** and **g**	Overlapping by the skeleton C-atoms of **e’**.
	**h** and **i**	Overlapping by the aryl C-atoms of **f’** and **g’**.
	**j**	Overlapping by the skeleton C-atoms of **h’** and **i’**.
Carbon atom signals in Ru(diamine) center	**k**	32.2 ppm.
	**l** and **m**	70.7, 75.9 ppm.
	**n**	101.5, 104.6 ppm.
	**o**	Overlapping by the aryl C-atoms of **f’** and **g’**.

### Orthogonal reactions investigations

Table 2 displays the results of the orthogonal reaction investigations aimed at achieving an optimal 1,4-addition/ATH cascade process. In the single-step 1,4-addition reaction (I) based on the optimizations of reaction conditions (Supplementary Table S1), it was found that the **5**-catalyzed model reaction of **6a** and **7a** in the mixed H_2_O/dioxane (v/v = 2:5) at 50 °C could produce (*R*)-**8a** in a 97% yield with 95%*ee*, which was comparable to that attained with its parent counterpart, suggesting a nearly same catalytic performance as its parent counterpart (entry 1 versus entry 2)^49^. However, the reaction with a mixture of homogeneous Rh(diene) and Ru(diamine) only gave (*R*)-**8a** in 33% yield with 29% *ee* (entry 3 versus entries 1-2). Interestingly, adding HCOONa (the second-step hydrogen resource) as an additive in either the **5**-catalyzed or homogeneous Rh(diene)-catalyzed model reaction did not affect the catalytic performance (entries 4 and 5). These comparisons demonstrated that the Rh(diene) deactivation was due to the Ru(diamine) species rather than the hydrogen resource, indicating that Rh(diene) and Ru(diamine) were incompatible in the single-step 1,4-addition reaction. Moreover, the enantioselectivity was not significantly affected by using **5’** as an additive (entry 6). However, the decreased yield indicated that the partly Rh(diene) were inactive, possibly due to their encapsulation within the polymeric framework leading to inactivation. This finding was further supported by the control contrastive reactions in the cases of a mixed Rh(diene) plus **4** and only **4’** as the catalyst (entries 7-8). These comparisons demonstrated that the Ru(diamine) immobilization could overcome the deactivation imparted on the Rh(diene) to a certain extent rather than completely, thereby confirming the advantage of site-isolated immobilization since catalyst **5** eliminated the negative disturbance imparted on the Rh(diene) centers (entry 1 versus entries 6 and 7).

**Table 2:** Orthogonal reactions investigations^a^.


Entry	Type	Catalyst	%Yield (%*ee*) of 8a^[*b*]^	%Yield (%*ee/dr*) of 9a^[*b*]^
1	I	**5**	97 (95)	–
2	I	[Rh]	99 (95)	–
3	I	[Rh] + [Ru]	33 (29)	–
4	I	**5** + HCO_2_Na	96 (95)	–
5	I	[Rh]+HCO_2_Na	98 (95)	–
6	I	[Rh] + **5'**	76 (86)	–
7	I	[Rh] + **4**	81 (90)	–
8	I	**4'**	97 (95)	–
9	II	**5**	–	95 (92/98:2)
10	II	[Ru]	–	96 (92/98:2)
11 ^[*c*]^	II	**5**	–	ND
12 ^[*c,d*]^	II	**5**	–	ND
13	II	**5** + [Rh]	–	29 (92/78:22)
14	II	**5’** + [Rh]	–	33 (92/80:20)
15	II	**5’** + **4'**	–	88 (91/91:9)
16	III	**5**	trace	93 (96/99:1)
17 ^[*c*]^	III	**5**	96 (95)	trace
18 ^[*e*]^	III	**5**	trace	31 (95/99:1)
19	III	**4’** + [Ru]	trace	66 (90/90:10)
20	III	[Rh] + **5'**	trace	52 (83/75:25)
21	III	[Rh] + [Ru]	trace	23 (68/65:35)
22	III	**4’** + **5'**	trace	85 (92/89:11)

^a^For reaction I, catalyst(s) (2.50 mol% of Rh-loadings), **6a** (0.10 mmol), **7a** (0.15 mmol), KOH (0.05 mmol), and/or additive in 2.0 mL of H_2_O/dioxane (v:v = 2:5) under the Ar atmosphere, 50 °C, 4 h. For reaction II, catalyst(s) (1.84 mol% of Ru-loadings), (*R*)-**8a** (92% *ee*, 0.10 mmol), HCO_2_Na (1.0 mmol) and/or additive in 2.0 mL of H_2_O/dioxane (v:v = 2/5) under the Ar atmosphere, 25 °C, 16 h. For reaction III, catalyst(s) (2.50 mol% of Rh-loadings and 1.84 mol% of Ru-loadings), **7a** (0.15 mmol), KOH (0.05 mmol), HCO_2_Na (1.0 mmol) in 1.70 mL of H_2_O/dioxane (v:v = 2:4) under the Ar atmosphere. After warming to 50 °C, a solution of **6a** (0.10 mmol) in 0.30 mL of dioxane was added dropwise to this solution and stirred at 50 °C for the first 4 h followed by 25 °C for 16 h. ND = not detected. [Rh] = homogeneous Rh(diene). [Ru] = homogeneous Ru(diamine).

^b1^H-NMR Yields, the %*ee*, and/or *dr* values were determined by chiral HPLC analysis.

^c^Data were obtained at 50 °C.

^d^Data were obtained with a mixture of **6a** (0.05 mmol) and (*R*)-**8a** (92% *ee*, 0.05 mmol) at 50 °C.

^e^Data were obtained at 25 °C.

In the ATH step (II) based on the optimization of reaction conditions (Supplementary Table S2), we conducted the **5**-catalyzed model reaction using (*R*)-**8a** with a 92% *ee* as a substrate at 25 °C. The result showed that it produced (*R,R*)-**9a** in 95% yield with 92% *ee* and 98/2 *dr*, which was similar to that achieved with its homogeneous counterparts despite the prolonged reaction time (entry 9 versus entry 10). We also observed that the **5**-catalyzed model reaction of (*R*)-**8a** at 50 °C did not work, further confirming a shrinking form of PNIPAM based on the designed on-and-off mode (entry 11). Additionally, we found that the model reaction with a mixed (*R*)-**8a** and **6a** as dual substrates at 50 °C only yielded tiny by-products coming from the reductive **6a**, suggesting the inactivity of **6a** in the single-step ATH transformation (entry 12). These results suggested the potential feasibility of a sequential 1,4-addition/ATH enantio-relay process by manipulating the reaction temperature and avoiding the reduction of the starting material itself. However, when we added homogeneous Rh(diene) to the reaction system, the **5**-catalyzed control reaction yielded a markedly decreased yield and diminished diastereoselectivity (entry 13), similar to that observed in a mixture of **5’** and homogeneous Rh(diene) system (entry 14). This finding suggested that the homogeneous Rh(diene) significantly affected the reactivity of the Ru(diamine), leading to Ru(diamine) deactivation in the single-step ATH transformation. Interestingly, this deactivation could be partially overcome in the case of the mixed **5’** plus **4’** conditions (entry 15 versus entries 13 and 14), indicating the immobilization advantage although it was inferior to that achieved with catalyst **5** (entry 15 versus entry 9).

In our final investigation, we incorporated the optimal two simple-step reactions into a one-pot 1,4-addition/ATH cascade process (III). During this investigation, three aspects of the evaluations were examined in the model cascade reaction of **6a** and **7a**. In the cases of only using **5** as a single catalyst, we found that the **5**-catalyzed model cascade reaction at an initial reaction temperature of 50°C for 4 h followed by subsequent reaction at 25°C for 16 h could produce the final (*R,R*)-**9a** in 93% yield with 96% *ee* and 99/1 *dr* (entry 16). However, when the **5**-catalyzed model reaction was conducted at only 50°C, it mainly produced the intermediate (*R*)-**8a** with trace amounts of (*R,R*)-**9a**, while conducting the reaction at only 25°C yielded (*R,R*)-**9a** in 31% yield (entries 17-18). These comparisons demonstrated that the manipulation of the reaction switching between the Rh-catalyzed 1,4-addition at 50 °C and Ru-catalyzed ATH at 25 °C enabled an efficient 1,4-addition/ATH cascade process (entry 16 versus entries 17-18). In the cases of using the mixed homogeneous catalyst plus the corresponding immobilized one as co-catalysts at an initial reaction temperature of 50°C for 4 h followed by subsequent reaction at 25°C for 16 h, we found that the model cascade reaction with either the mixed **4’** plus Ru(diamine) or the mixed Rh(diene) plus **5’** as co-catalysts only provided the final targeting products with the low yield and selectivity (entries 19–20). This suggested that a single immobilization did not solve the issue of mutual deactivations between the Rh(diene)-complex and Ru(diamine)-complex. In the cases of using mixed two homogeneous catalysts or mixed two immobilized catalysts as co-catalysts, we found that the model cascade reaction in the mixed homogeneous Ru(diamine) and Rh(diene) system only produced (*R,R*)-**9a** with poor catalytic results (entry 21). Despite the drawback of their mutual deactivations could be improved in the case of using mixed **4’** plus **5’** as co-catalysts (entry 22), the still low yield and enantio/diastereoselectivity relative to that obtained with catalyst **5** revealed that the physically mixed two immobilized catalysts did not completely overcome their mutual deactivations due to the flexible polymeric swelling nature (entry 22 versus entry 16). All these comparisons elucidated the superiority of the designed thermoresponsive site-isolated bifunctional catalyst, which enabled a 1,4-addition/ATH cascade process to be accomplished from incompatibility to compatibility.

### Substrate scope and application

**Fig. 5:**
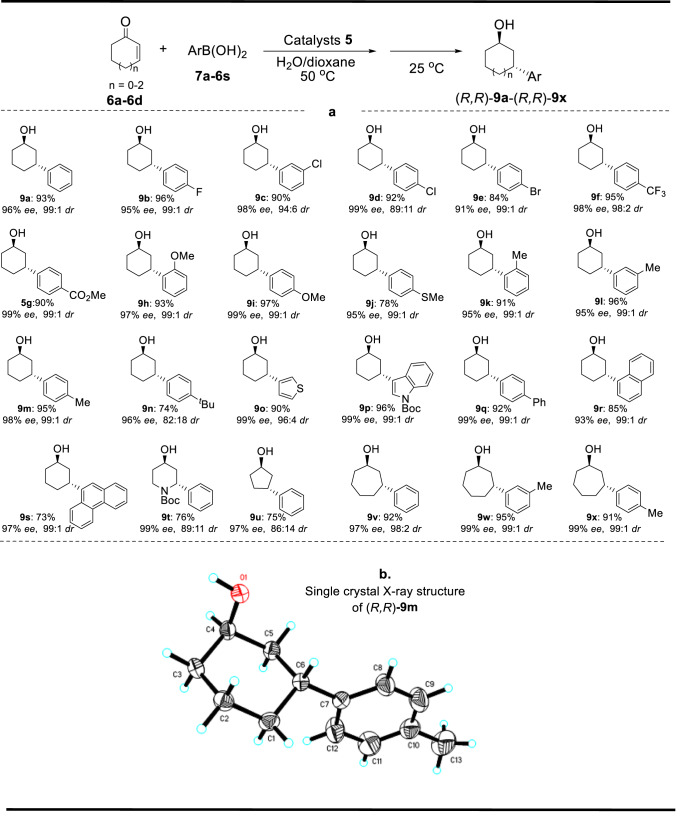
Scope for the 1,4-addition/reduction reaction and the crystal structure of (*R,R*)-9m. **a** Standard reaction condition. Catalyst 5 (11.11 mg, 2.50 mol% of Rh-loadings and 1.84 mol% of Ru-loadings based on ICP analysis), **7** (0.15 mmol), KOH (0.05 mmol), HCO_2_Na (1.0 mmol) in 1.70 mL of H_2_O/dioxane (v:v = 2:4) were added sequentially to a 10.0 mL round−bottom flask purged with nitrogen in turn. After warming to 50 °C, a solution of **6** (0.10 mmol) in 0.30 mL of dioxane was added dropwise to this solution and stirred at 50 °C for 2-5 h. After completion of the first transformation monitored by the thin-layer chromatography (TLC) and cooling down to 25 °C, the mixture was allowed to react at 25 °C for a further 10-24 h. **b** Crystal structure of (*R,R*)-**9m**.

Based on the optimal reaction conditions, catalyst **5** was further investigated with a series of substrates to confirm its general feasibility in this 1,4-addition/ATH dual enantioselective cascade process, as shown in Fig. 5 (Supplementary Figs. S8 and S9). It was observed that all tested cyclohexenones reacted smoothly with various arylboronic acids, producing the corresponding 1,3-distereocentered γ-substituted cyclohexanols in high yields and enantio/diastereoselectivities. Furthermore, it was noteworthy that the enantio/diastereoselectivities of the reaction were not significantly influenced by the structures and electronic properties of substituents at the aryl group since various electron-withdrawing and -donating substituents substrates were equally efficient in the 1,4-addition/ATH cascade reaction ((*R,R*)-**9b-**(*R,R*)-**9g** versus (*R,R*)-**9h-**(*R,R*)-**9n**). Additionally, the cascade reactions with other substrates such as thiazyl, biphenyl, and fused rings also proceeded smoothly, yielding the final products with high yields and *ee/dr* values ((*R,R*)-**9o-**(*R,R*)-**9s**).

Interestingly, the cascade process was not limited to cyclohexenones as substrates. One practical dihydropyridinone, one typical cyclopentanone, and three representative cyclohexanones could also be used in the construction of 1,3-distereocentered γ-substituted cyclic alcohols ((*R,R*)-**9t-**(*R,R*)-**9x)** in good yields with high *ee* and *dr* values. Fortunately, the crystal of (*R,R*)-**9m** could be obtained, which disclosed an (*R*,*R*)–configuration determined by a single–crystal X–ray diffraction analysis (CCDC–2235252, Supplementary Data 1 and 2, and also Supplementary Table S3).

**Fig. 6:**
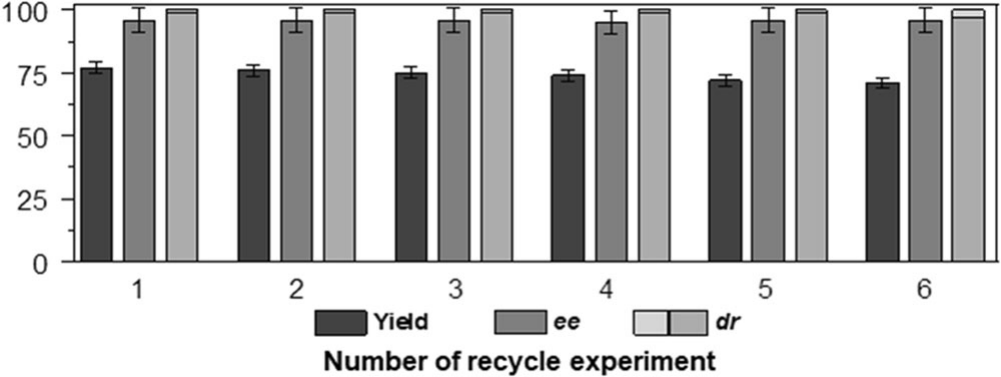
Reusability of catalyst 5 in the 1,4-addition/ATH reaction of 6a and 7a. Reaction condition. Catalyst **5** (111.10 mg, 2.50 mol% of Rh-loadings and 1.84 mol% of Ru-loadings based on ICP analysis), **7a** (1.50 mmol), KOH (0.50 mmol), HCO_2_Na (10.0 mmol) in 17.0 mL of H_2_O/dioxane (v:v = 2:4) were added sequentially to a 50.0 mL round−bottom flask purged with nitrogen in turn. After warming to 50 °C, a solution of **6a** (1.0 mmol) in 3.0 mL of dioxane was added dropwise to this solution at 50 °C. The mixture was stirred at 50 °C for the first 4 h followed by a continuous reaction at 25 °C for the second 16 h. The error bars represent the standard deviation.

Furthermore, a gram-scale preparation was also performed through the use of (*R*,*R*)–**9** **m** as a representative example. It was found that when using 6.0 mmol of **6a** and 9.0 mmol of **7i** as substrates, the **5**-catalyzed cascade reaction produced 1.09 grams of (*R,R*)-**9m** in 96% yield with 98% *ee* and 99/1 *dr*, demonstrating the synthetic utility of this cascade process. Aside from the synthetic utility, it was worth mentioning that catalyst **5** could also be easily recovered through simple filtration and used repeatedly in this cascade process. To examine the catalyst’s stability, the **5**-catalyzed cascade reaction of **6a** and **7a** by setting the reaction time to be within 10 h was further investigated, as shown in Fig. 6 (Supplementary Table S4 and Fig. S10). It was found that in six consecutive runs, catalyst **5** was still able to produce (*R*,*R*)-**9a** with a slightly decreased yield from 77% to 71% with a nearly maintainable enantio/diastereoselectivity, thereby confirming the stability of the catalyst. This observation was consistent with the hydrodynamic diameters of catalyst **5**, suggesting that the designed catalyst with the on-and-off mode of the thermoresponsive PNIPAM in **5** was suitable for the preparation of γ-substituted cyclic alcohols with 1,3-position dual stereocenters for the potential applications.

### Mechanistic study

**Fig. 7:**
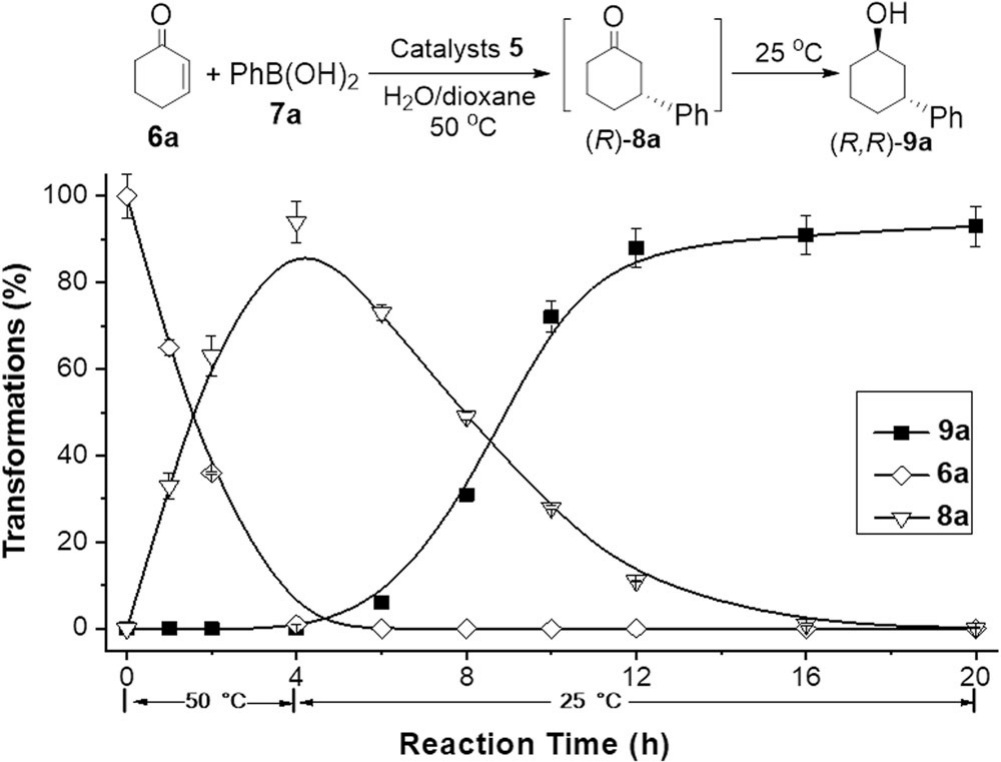
The time course of the 5-catalyzed addition/ATH cascade process of 6a and 7a by using the standard reaction conditions. Reaction procedure. To a suspension of catalyst **5** (2.50 mol% of Rh-loadings and 1.84 mol% of Ru-loadings), 1.5 equiv of **7a**, 0.5 equiv of KOH, and 10.0 equiv of HCOONa in 1.70 mL of H_2_O/dioxane (v:v = 2:4) was added a solution of 1.0 equiv of **6a** in 0.30 mL of dioxane at 50 °C. The mixture was stirred at 50 °C for the first 4 h followed by a continuous reaction at 25 °C for the second 16 h. The error bars represent the standard deviation.

It was notable that the switchable on-and-off mode of the thermoresponsive PNIPAM in **5** played a crucial role in the harmonization of an enantio-relay catalytic process, which could enhance the diastereoselectivity of products since high enantioselectivity in the initial 1,4-addition reaction often resulted in high diastereoselectivity in the subsequent ATH transformation. To gain insight into the catalytic process *via* a favorable enantio-relay reaction sequence, a kinetic investigation of the **5**-catalyzed cascade reaction of **6a** and **7a** was performed, as shown in Fig. 7. Initially, we found that the 1,4-addition reaction occurs over the first 4 h at 50 °C as the concentration of **6a** sharply decreases. During this transformation, a maximum yield of 94% for the formation of (*R*)-**8a** was observed. Subsequently, when the reaction temperature is lowered to 25 °C, the ATH reduction of (*R*)-**8a** is triggered. The ATH transformation proceeds rapidly between 4 to 12 h with a decreased yield of (*R*)-**8a**. Finally, the reduction of (*R*)-**8a** steadily provides (*R,R*)-**9a** for a further 8 h. This investigation discloses a reaction sequence comprised of a 1,4-addition followed by the subsequent ATH process.

**Fig. 8:**
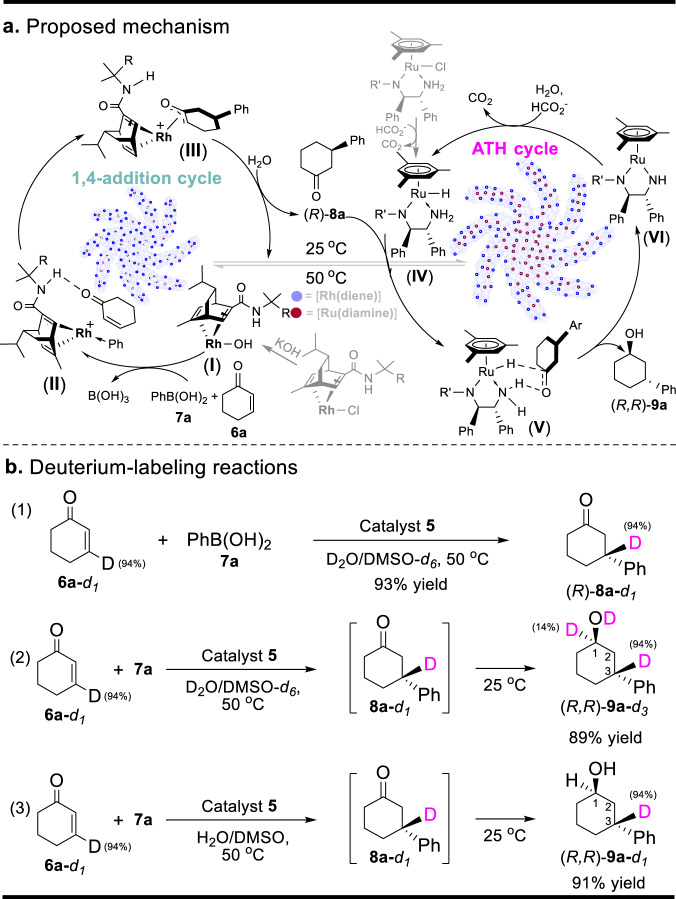
Proposed mechanism for the 1,4-addition/ATH process verified by the deuterium-labeling reactions. **a** Proposed mechanism for a dual catalytic cycle. **b** Deuterium-labeling reactions: (1) The deuterium-labeling 1,4-addition reaction of **6a**–*d*_*1*_ in the D_2_O/DMSO-*d*_6_, (2) The deuterium-labeling cascade reaction of **6a**–*d*_*1*_ and **7a** in the D_2_O/DMSO-*d*_6_, (3) The deuterium-labeling cascade reaction of **6a**–*d*_*1*_ and **7a** in the H_2_O/DMSO.

After obtaining a clear time course for the **5**–catalyzed 1,4-addition/ATH reaction of **6a** and **7a**, a plausible cascade reaction mechanism through dual catalysis is proposed, as shown in Fig. 8. In the first-step 1,4-addition catalytic cycle^53,54^, the Rh(diene) precursor on the outer surface of **5** under basic conditions initially produces the hydroxorhodium (**I**). The starting material **7** reacts with this hydroxorhodium to lead to the formation of the arylrhodium species with the binding of **6** (**II**). The 1,4-addition then takes place, leading to an oxa-π-allylrhodium intermediate (**III**) *via* the insertion of α,β-unsaturated carbonyl **6**. Finally, the hydrolysis of this intermediate releases **8** and regenerates the hydroxorhodium, completing the first-step catalytic cycle. The deuterium-labeling reaction of **6a**–*d*_*1*_ (with a 94% deuterium ratio) and **7a** supported this formal 1,4-addition pathway since the **5**-catalyzed 1,4-addition reaction in the D_2_O/DMSO-*d*_6_ produces (*R*)–**8a**–*d*_*1*_ with the maintainable deuterium ratios (Eq. 1 in Fig. 8) (Supplementary Fig. S11a, 11b).

In the second-step ATH catalytic cycle^46,55,56^, the releasing **8** enters the inner lay of **5** to reach the *in situ–*generated chiral ruthenium hydride species (**IV**) that comes from the reaction of Ru(diamine) precursor with HCO_2_^-^. The ATH transformation then occurs *via* an adduct (**V**) formed by the reaction of the ruthenium hydride and (*R*)–**8**. Finally, the release of (*R,R*)–**9** through an intermediate ruthenium complex (**V**) and the regeneration of the ruthenium hydride species with the assistance of HCO_2_^-^ complete the second-step cycle. The deuterium–labeling reaction of **6a**–*d*_*1*_ and **7a** confirmed this cycle because this adduct formed by the reaction of the in-situ generated ruthenium deuteride species and (*R*)–**8** in the D_2_O/DMSO-*d*_6_ conditions leads to the product (*R,R*)–**9a**–*d*_*3*_ with a 14% deuterium ratio at 1-position relative to (*R,R*)–**9a**–*d*_*1*_ without deuterium incorporation at 1-position in the H_2_O/DMSO conditions (Eq. 2 versus Eq. 3 in Fig. 8) (Supplementary Fig. S11c–11e).

In conclusion, we have developed a cross-linked PNIPAM-hydrogel-supported site-isolated bifunctional catalyst. This catalyst features a thermoresponsive poly(*N*-isopropylacrylamide) in the intermediate layer, with chiral Rh(diene)-complexes tethered on the outer surface and chiral Ru(diamine)-complexes entrapped within the interior of the hydrogel. By utilizing the compartmental and thermoresponsive functions of poly(*N*- isopropylacrylamide), this catalyst eliminates the mutual deactivations between Rh(diene)-complexes and chiral Ru(diamine)-complexes, and harmonizes the conflicting reaction conditions, enabling a cascade to be accomplished under incompatible conditions. As presented in this study, the temperature-tuned 1,4-addition/asymmetric transfer hydrogenation dual enantioselective cascade reaction enables direct access to 1,3-distereocentered γ-substituted cyclic alcohols from the reaction of cyclic enones and arylboronic acids in high yields and enantio/diastereoselectivities. The kinetic investigation and deuterium-labeling experiments reaction reveal an enantio-relay reaction sequence comprised of a 1,4-addition followed by the subsequent ATH process.

## Methods

### General procedure for the 1,4-addition/ATH cascade process

Catalyst **5** (11.11 mg, 2.50 mol% of Rh-loadings and 1.84 mol% of Ru-loadings based on ICP analysis), **7** (0.15 mmol), KOH (0.05 mmol), HCO_2_Na (1.0 mmol) in 1.70 mL of H_2_O/dioxane (v:v = 2:4) were added sequentially to a 10.0 mL round−bottom flask purged with nitrogen in turn. After warming to 50 °C, a solution of **6** (0.10 mmol) in 0.30 mL of dioxane was added dropwise to this solution and stirred at 50 °C for 2-5 h. After completion of the first transformation monitored by the thin-layer chromatography (TLC) and cooling down to 25 °C, the mixture was allowed to react at 25 °C for a further 10-24 h. After completion of the reaction, the heterogeneous catalyst was separated for the recycling experiment. The aqueous solution was extracted by Et_2_O (3 × 3.0 mL). The combined Et_2_O was washed with brine twice and dehydrated with Na_2_SO_4_. After the evaporation of Et_2_O, the residue was purified by silica gel flash column chromatography to afford the desired product. The yield was the isolated yield, and the *ee* and *dr* values were determined using an HPLC analysis with a Photo-Diode Array detector using a Daicel chiral cell column (Φ 0.46 × 25 cm).

### Supplementary information

Supplementary Information

Description of Additional Supplementary Files

Supplementary Data 1

Supplementary Data 2

## Data Availability

Supplementary information, Supplementary Data 1 for the CIF file of the crystal structure of (R,R)-9m, and Supplementary Data 2 for the check of CIF file of the crystal structure of (R,R)-9m are available in the online version of the paper. Data supporting the findings of this work are also available from the corresponding author upon reasonable request.
