# Supplementary material for: Bridging the incompatibility gap in dual asymmetric catalysis over a thermoresponsive hydrogel-supported catalyst
Source: Commun Chem. 2024 Jan 3;7:2. doi: 10.1038/s42004-023-01085-z (PMC10764871; doi:10.1038/s42004-023-01085-z)

Supporting Information

Bridging the Incompatibility Gap in Dual Asymmetric Catalysis over a Thermoresponsive Hydrogel-Supported Catalyst

Renfu Huang, Shoujin Yang, Zhipeng Hu, Bangtai Peng, Yuanli Zhu, Tanyu Cheng, and Guohua Liu*

Key Laboratory of Resource Chemistry of Ministry of Education, Shanghai Key Laboratory of Rare Earth Functional Materials, Shanghai Normal University, No.100 Guilin Rd, Shanghai, China.

Email: ghliu@shnu.edu.cn.

CONTENTS

Experimental. S2

Figure S1. IR spectra of **2**, **4**, and catalyst **5**. S11

Figure S2. ^{1}H-NMR spectra of **2** and **4** S12

Figure S3. Temperature-dependent transmittance and the dispersive situations of **4** S13

Figure S4. The dispersive situations of catalyst **5** in the co-solvent system S13

Figure S5. Morphology and rheology investigations of catalyst **5** at 25 ℃ and 50 ℃ S14

Figure S6. Average hydrodynamic diameters distribution measurement of **5**... .... S15

Figure S7. Solid-state ^{13}C MAS NMR spectra of **2, 4,** and catalyst **5**. S20

Table S1. Optimizing reaction conditions for the 1,4-addition reaction. S21

Table S2 Optimizing reaction conditions for the ATH transformation S21

Figure S8. HPLC analyses of chiral products S22

Figure S9. Characterizations of chiral products S46

Table S3. Crystal data and structure refinement for (*R,R*)-**9m** S72

Table S4. Reusability of catalyst **5**. S73

Figure S10. Reusability of catalyst **5** in the 1,4-addition/ATH cascade process of **6a** and **7a**. S73

Figure S11. Contrastive ^{1}H-NMR spectra for the deuterium labeling experiments. S77

Experimental

1. General: All reactions involving air- or moisture-sensitive reagents or intermediates were carried out in oven-dried glassware using standard Schlenk techniques. All commercially available reagents were purchased from Sigma-Aldrich, Alfa Aesar, TCI Chemicals, Acros Organics, or ABCR in the highest purity grade and used without further purification.

2. Characterization: Ru loading amounts in the catalysts were analyzed using an inductively coupled plasma optical emission spectrometer (ICP-OES, Varian VISTA−MPX). Molecular weights and molecular weight distributions were determined by gel permeation chromatography (GPC) equipped with Waters 1515 pump and Waters 2414 differential refractive index detector (set at 30 ℃), employing a series of three linear Styragel columns (HR1, HR2, and HR4) at an oven temperature of 45 ℃. The eluent was DMF at a flow rate of 1.0 mL/min. A series of low polydispersity polystyrene standards were employed for calibration. Solid-state NMR experiments were explored on a Bruker AVANCE spectrometer at a magnetic field strength of 9.4 T with ^{1}H frequency of 400.1 MHz, and ^{13}C frequency of 100.5 MHz with 4 mm rotor at two spinning frequencies of 5.5 kHz and 8.0 kHz, TPPM decoupling is applied in the during the acquisition period. ^{1}H cross-polarization in the solid-state NMR experiments was employed using a contact time of 2 ms and pulse lengths of 4μs. Liquid-state NMR (^{1}H NMR and ^{13}C NMR) spectra were performed on a Bruker AVANCE spectrometer at a magnetic field strength of 9.4 T with a ^{1}H frequency of 400 MHz and a ^{13}C frequency of 100 MHz. Spectra were calibrated relative to the solvent's residual proton and carbon chemical shift: $CHCl_3$ (δ (ppm) = 7.26 ppm for ^{1}H NMR and δ (ppm) = 77.36 ppm for ^{13}C NMR). Data are reported as follows: chemical shift, multiplicity (s = single, d = doublet, t = triplet, q = quartet, brs = broad single, m = multiplet), coupling constants (Hz), and integration. Mass spectra were recorded on a Finnigan MAT 4200S, a Bruker Daltonics Micro Tof, and a Waters-Micromass Quatro LCZ (ESI); peaks are given in m/z (% of basis peak).

3. Preparation of catalyst 5: A representative synthetic procedure is as follows. *(The first step for the synthesis of the diamine-functionalized hydrogel (**2**)),* to a solution of sodium lauryl sulfate (surfactant: SDS) (125.0 mg, 0.43 mmol) in 50.0 mL of pure water was added ethylene glycol dimethacrylate (EGDMA) (2.30 μL, 0.012 mmol), and purged with nitrogen at 25 °C. The ArDPEN-functionalized monomer (**1:** VinylArPDEN = *N*-((1*R*,2*R*)-2-amino-1,2-diphenylethyl)-4-vinylbenzenesulfonamide) (151.26 mg, 0.40 mmol) and *N*-isopropylacrylamide (NIPAM) (454.23 mg, 4.02 mmol) were then added to the solution under constant stirring under nitrogen condition at 35 °C, and the mixture was allowed to stir under nitrogen for 30 minutes. The initiator potassium persulfate 5.0 mg, 0.018 mmol) was added and the reaction flask was immersed in a pre-heated oil bath set to 70 °C. The reaction was

allowed to stir for 12 hours under continuous stirring at 800 rpm. After completion of the polymerization, the reaction was quenched by cooling to RT and exposure of the flask contents to oxygen. The polymer was purified by dialysis against pure water to remove excess SDS, and the resulting mixture was freeze-dried to afford hydrogel (**2**) (464.12 mg, 76% yield) for the second synthesis. *(The second step for the synthesis of the diene-/diamine-functionalized hydrogel (4)),* A suspension of hydrogel (**2**) (400.0 mg) in 25.0 mL of pure water was previously prepared in a pre-heated oil bath at 70 °C and purged with nitrogen at 70 °C. To this suspension was added dropwise a solution of SDS (11.52 mg, 0.04 mmol), EGDMA (1.84 μL, 0.01 mmol), NIPAM (144.64 mg, 1.28 mmol), and the initiator potassium persulfate (2.0 mg, 0.007 mmol) in 20.0 mL of the pure water, and the resulting mixture was polymerized at 70 °C for 6 hours under continuous stirring at 800 rpm. After that, another solution of SDS (2.88 mg, 0.01 mmol), NIPAM (36.16 mg, 0.32 mmol), and (1*R*,4*R*,7*R*)-7-isopropyl-5-methyl-N-(2-methyl-1-((4-vinylbenzyl)oxy)propan-2-yl)bicyclo[2.2.2]octa-2,5-diene-2-carboxamide (**3**) (157.31 mg, 0.40 mmol) in 5.0 mL was added dropwise to this suspension and the further polymerization at 70 °C for 2 hours. After completion of the polymerization, the reaction was quenched by cooling to RT and exposure of the flask contents to oxygen. The polymers were purified by dialysis against pure water to remove excess SDS, and the resulting mixture was freeze-dried to afford hydrogel (**4**) (503.14 mg, 68% yield) for the second synthesis. *(The third step for the synthesis of hydrogel-supported* ***5****)* The hydrogel (**4**) (500.0 mg) was suspended in 20 mL of 1,4-dioxane, and KOH (2.0 mL, 3.0 mmol; 1.5 M aqueous) and $(RhCl(C_2H_4)_2)_2$ (77.57 mg, 0.20 mmol) was added. The resulting mixture was stirred at 50 °C for 1.0 h. The resulting solids were filtered, rinsed with excess H_2O, and washed with excess CH_2Cl_2. After Soxhlet extraction in CH_2Cl_2 solvent to remove the remaining $(RhCl(C_2H_4)_2)_2$ for 24 h, the solid was dried at 60 °C under reduced pressure overnight to afford **4'** (447.20 mg, 83% yield). The collected **4'** (400.0 mg) was suspended in 20 mL of deionized H_2O and $(MesRuCl_2)_2$ (46.74 mg, 0.08 mmol) was added. The resulting mixture was stirred at 25 °C for 12 h. The resulting solids were filtered, rinsed with excess distilled H_2O, and washed with excess CH_2Cl_2. After Soxhlet extraction in CH_2Cl_2 solvent to remove the remaining $(MesRuCl_2)_2$ for 24 h, the solid was dried at 60 °C under reduced pressure overnight to afford catalyst **5** as a greyish purple powder (416.66 mg, 86% yield) as a brown powder. Inductively coupled plasma optical emission spectroscopy (ICP-OES) showed that the Rh-loading and Ru-loading were 23.17 mg (0.2251 mmol) and 16.91 mg (0.1659 mmol) per gram of catalyst. IR (KBr) cm^{-1}: 3405.3 (s), 3193.4 (s), 3105.5 (s), 2968.8 (s), 2921.9 (m), 2858.1 (m), 1657.2 (s), 1601.2 (s), 1553.3 (s), 1449.5 (s), 1401.5 (s), 1305.7 (w), 1257.8 (w), 1233.8 (w), 1122.0 (w), 1026.2 (w), 882.5 (w), 834.5 (w), 810.6 (w), 762.7 (w), 798.8 (m), 674.8 (w), 618.9 (w), 579.0 (w). ^{13}C CP/MAS NMR (161.9 MHz): 179.6−170.9 ($\underline{C}$=O), 153.6−113.9 ($\underline{C}$ of C=C, and $\underline{C}$ of Ar and Ph), 104.6, 101.5 (−$\underline{C}$H of mesitylene), 75.9, 70.7 (−N$\underline{C}$H$\underline{C}$HN−), 69.9−65.3 (−O$\underline{C}H_2$C−, −O$\underline{C}H_2$$\underline{Ar}$−, −O$\underline{C}H\underline{C}$HO−), 54.8−35.3 (−$\underline{C}$H(C=CH)$_2$ in

cycling moiety, −N*C*H(CH_3)$_2$, −*C*HCO−skeleton, −*C*HAr-skeleton), 34.5−25.9 (−CO*C*H(CH_3)$_2$−, −*C*H_3 in mesitylene, and *C* of −*C*− in cycling moiety without connected double bonds), 25.2−16.9 (−*C*H_2−skeleton), 13.9 (−*C*H_3 and −CH(*C*H_3)$_2$) ppm

4. General procedure for the 1,4-addition process. A typical procedure was as follows: The catalyst (2.50 mol% of Rh-loadings), **7a** (0.15 mmol), KOH (0.05 mmol), and/or additive in 1.70 mL of H_2O/dioxane (v:v = 2:4) were added sequentially to a 10.0 mL round−bottom flask purged with nitrogen in turn. After warming to 50 °C, a solution of **6a** (0.10 mmol) in 0.30 mL of dioxane was added dropwise to this solution and stirred at 50 °C for 4 h. After completion of the reaction, the aqueous solution was extracted by Et_2O (3 × 3.0 mL). The combined Et_2O was washed with brine twice and dehydrated with Na_2SO_4. After the evaporation of Et_2O, the residue was purified by silica gel flash column chromatography to afford the desired product (*R*)-**8a**. The yield was determined by the H-NMR spectra, and the *ee* values were determined using an HPLC analysis with a Photo-Diode Array detector using a Daicel chiral cell column (Φ 0.46 × 25 cm)

5. General procedure for the ATH process. A typical procedure was as follows: The catalyst (1.84 mol% of Ru-loading), (*R*)-**8a** (92% *ee*, 0.10 mmol), HCO_2Na (1.0 mmol) in 2.0 mL of H_2O/dioxane (v:v = 2:5) were added sequentially to a 10.0 mL round−bottom flask purged with nitrogen in turn at room temperature. The resulting mixture was stirred at 25 °C for 16 h. After completion of the reaction, The aqueous solution was extracted by Et_2O (3 × 3.0 mL). The combined Et_2O was washed with brine twice and dehydrated with Na_2SO_4. After the evaporation of Et_2O, the residue was purified by silica gel flash column chromatography to afford the desired product (*R,R*)-**9a**. The yield was determined by the H-NMR spectra, and the *ee* and *dr* values were determined using an HPLC analysis with a Photo-Diode Array detector using a Daicel chiral cell column (Φ 0.46 × 25 cm).

6. A gram-scale preparation of 9m in the 1,4-addition/ATH cascade process of 6a and 7i.

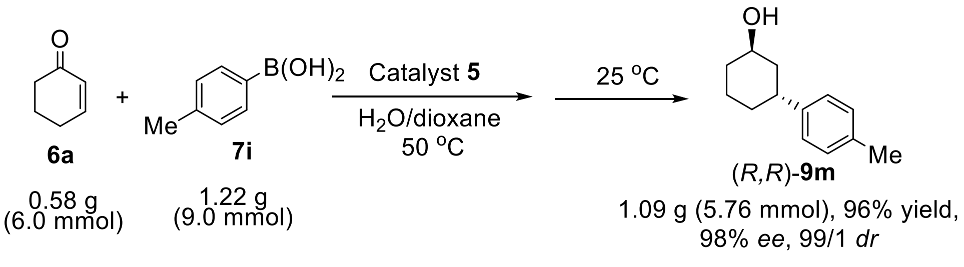

A typical procedure was as follows: The catalyst **5** (2.50 mol% of Rh-loadings and 1.84 mol% of Ru-loadings based on ICP analysis), **7i** (9.0 mmol), KOH (3.0 mmol), HCO_2Na (60.0 mmol) in 30.0 mL of H_2O/dioxane (v:v = 2:4) were added sequentially to a 100.0 mL round−bottom flask purged with nitrogen in turn. After warming to 50 °C, a solution of **6a** (6.0 mmol) in 5.0 mL of dioxane was added

dropwise to this solution and stirred at 50 °C for 6 h. After completion of the first transformation monitored by the thin-layer chromatography (TLC) and cooling down to 25 °C, the mixture was allowed to react at 25 °C for a further 20 h. After completion of the reaction, the catalyst was separated and the aqueous solution was extracted by Et_2O (3 × 3.0 mL). The combined Et_2O was washed with brine twice and dehydrated with Na_2SO_4. After the evaporation of Et_2O, the residue was purified by silica gel flash column chromatography to afford the desired product.

7. Reusability of catalyst 5 in the 1,4-addition/ATH cascade process of 6a and 7a. A typical procedure was as follows: The catalyst **5** (111.10 mg, 2.50 mol% of Rh-loadings and 1.84 mol% of Ru-loadings based on ICP analysis), **7a** (1.50 mmol), KOH (0.50 mmol), HCO_2Na (10.0 mmol) in 17.0 mL of H_2O/dioxane (v:v = 2:4) were added sequentially to a 50.0 mL round−bottom flask purged with nitrogen in turn. After warming to 50 °C, a solution of **6a** (1.0 mmol) in 3.0 mL of dioxane was added dropwise to this solution and stirred at 50 °C for 4 h. After completion of the first transformation monitored by the thin-layer chromatography (TLC) and cooling down to 25 °C, the mixture was allowed to react at 25 °C for a further 16 h. After completion of the reaction, the heterogeneous catalyst was separated for the recycling experiment. The aqueous solution was extracted by Et_2O (3 × 3.0 mL). The combined Et_2O was washed with brine twice and dehydrated with Na_2SO_4. After the evaporation of Et_2O, the residue was purified by silica gel flash column chromatography to afford the desired product.

9. Data of chiral products.

9a: (1*R*,3*R*)-3-phenylcyclohexan-1-ol. White solid, 93% yield, 96% *ee*, 99/1 *dr*. ^{1}H NMR (400 MHz,

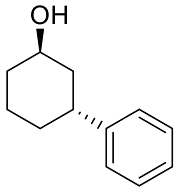

$CDCl_3$) δ 7.22 (t, J = 7.54 Hz, 2H), 7.19 – 7.06 (m, 3H), 4.16 (p, J = 3.06 Hz, 1H), 2.93 (tt, J = 12.35, 3.50 Hz, 1H), 1.93 – 1.68 (m, 4H), 1.67 – 1.49 (m, 3H), 1.51 – 1.31 (m, 2H). ^{13}C NMR (101 MHz, $CDCl_3$) δ 144.21, 135.48, 129.18 (d, J = 5.13 Hz), 126.82 (d, J = 15.25 Hz), 66.93, 40.67, 37.18, 33.96, 32.48, 20.55. HPLC (Chiralpak IC, elute: Hexanes/*i*-PrOH =95/5, detector: 254 nm, flow rate: 1.0 mL/min, 25 ℃).

9b: (1*R*,3*R*)-3-(4-fluorophenyl)cyclohexan-1-ol. White solid, 96% yield, 95% *ee*, 99/1 *dr*. ^{1}H NMR

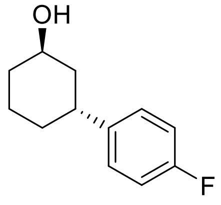

(400 MHz, $CDCl_3$) δ 7.08 (dd, J = 8.23, 5.33 Hz, 2H), 6.89 (t, J = 8.76 Hz, 2H), 4.15 (s, 1H), 3.40 (d, J = 3.36 Hz, 1H), 2.91 (tt, J = 12.51, 3.53 Hz, 1H), 1.92 – 1.67 (m, 4H), 1.67 – 1.40 (m, 5H), 1.40 – 1.25 (m, 1H). ^{13}C NMR (101 MHz, $CDCl_3$) δ 162.50, 160.08, 142.85 (d, J = 3.12 Hz), 128.32 (d, J = 7.86 Hz), 115.13 (d, J = 20.82 Hz), 66.83, 40.71, 36.93, 34.06, 32.47, 20.49. HPLC (Chiralpak AD, elute: Hexanes/*i*-PrOH =98/2, detector: 254 nm, flow rate: 1.0 mL/min, 25 ℃).

9c: (1*R*,3*R*)-3-(3-chlorophenyl)cyclohexan-1-ol. Colorless oil, 90% yield, 98% *ee*, 94/6 *dr*. ^{1}H NMR

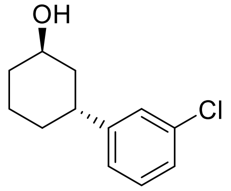

(400 MHz, $CDCl_3$) δ 7.16 – 7.02 (m, 3H), 7.00 (dt, *J* = 7.46, 1.60 Hz, 1H), 4.14 (p, *J* = 3.04 Hz, 1H), 3.63 (t, *J* = 4.28 Hz, 0H), 2.91 (tt, *J* = 12.38, 3.43 Hz, 1H), 1.98 – 1.92 (m, 1H), 1.90 – 1.66 (m, 4H), 1.61 – 1.26 (m, 4H). ^{13}C NMR (101 MHz, $CDCl_3$) δ 149.27, 134.16, 129.70, 127.17, 126.14, 125.33, 66.64, 40.31, 37.42, 33.61, 32.35, 20.38. HPLC (Chiralpak AD, elute: Hexanes/*i*-PrOH =99/1, detector: 220 nm, flow rate: 1.0 mL/min, 25 ℃).

9d: (1*R*,3*R*)-3-(4-chlorophenyl)cyclohexan-1-ol. Colorless oil, 92% yield, 99% *ee*, 89/11 *dr*. ^{1}H NMR

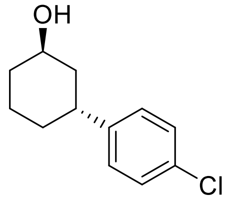

(400 MHz, $CDCl_3$) δ 7.21 – 7.13 (m, 2H), 7.09 – 7.02 (m, 2H), 4.14 (q, J = 3.02 Hz, 1H), 2.91 (tt, J = 12.41, 3.39 Hz, 1H), 1.86 (dq, J = 3.60, 1.83 Hz, 1H), 1.84 – 1.74 (m, 3H), 1.72 (s, 2H), 1.60 – 1.41 (m, 3H), 1.32 (qd, J = 13.16, 12.71, 3.76 Hz, 1H). ^{13}C NMR (101 MHz, $CDCl_3$) δ145.66, 131.58, 128.53, 128.37, 66.72, 40.49, 37.08, 33.81, 32.47, 20.44. HPLC (Chiralpak OD, elute: Hexanes/*i*-PrOH =99/1, detector: 254 nm, flow rate: 1.0 mL/min, 27 ℃).

9e: (1*R*,3*R*)-3-(4-bromophenyl)cyclohexan-1-ol. Colorless oil, 84% yield, 91% *ee*, 99/1 *dr*. ^{1}H NMR

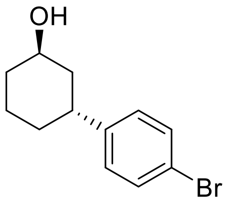

(400 MHz, $CDCl_3$) δ 7.36 – 7.29 (m, 2H), 7.06 – 6.98 (m, 2H), 4.16 (p, *J* = 3.02 Hz, 1H), 2.91 (tt, *J* = 12.41, 3.38 Hz, 1H), 1.91 – 1.62 (m, 5H), 1.61 (s, 3H), 1.55 – 1.15 (m, 7H). ^{13}C NMR (101 MHz, $CDCl_3$) δ 146.19, 131.52, 128.85, 119.66, 66.77, 40.44, 37.18, 33.78, 32.48, 20.44. HPLC (Chiralpak OD, elute: Hexanes/*i*-PrOH =98/2, detector: 220 nm, flow rate: 1.0 mL/min, 27 ℃).

9f: (1*R*,3*R*)-3-(4-(trifluoromethyl)phenyl)cyclohexan-1-ol.White solid, 95% yield, 98% *ee*, 98/2 *dr*.

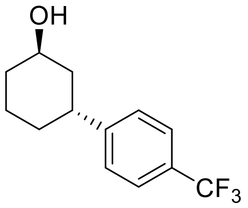

^{1}H NMR (400 MHz, $CDCl_3$) δ 7.48 (dd, J = 8.23, 3.95 Hz, 2H), 7.25 (dd, J = 8.11, 4.37 Hz, 2H), 4.19 (t, J = 3.07 Hz, 0.76H), 3.68 (tt, J = 10.86, 4.31 Hz, 0.25H), 3.02 (tt, J = 12.50, 3.51 Hz, 0.78H), 2.58 (tt, J = 12.09, 3.39 Hz, 0.27H), 2.14 – 2.04 (m, 0H), 2.01 (d, J = 12.41 Hz, 0H), 1.95 – 1.71 (m, 4H), 1.67 – 1.42 (m, 3H), 1.37 – 1.14 (m, 1H). ^{13}C NMR (101 MHz, $CDCl_3$) δ 151.27, 150.26, 127.38, 127.24, 125.43 (p, J = 3.85 Hz), 70.84, 66.62, 42.92, 42.78, 40.26, 37.62, 35.32, 33.61, 33.28, 32.43, 24.46, 20.38. ^{19}F NMR (376 MHz, $CDCl_3$) δ -62.27, -62.31.HPLC (Chiralpak AD, elute: Hexanes/*i*-PrOH =98/2, detector: 254 nm, flow rate: 1.0 mL/min, 25 ℃).

9g: methyl 4-((1*R*,3*R*)-3-hydroxycyclohexyl)benzoate. White solid, 90% yield, 99% *ee*, 99/1 *dr*. ^{1}H

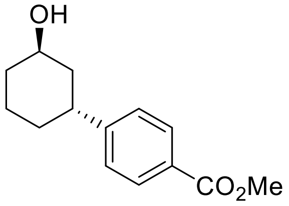

NMR (400 MHz, $CDCl_3$) δ 7.91 – 7.84 (m, 2H), 7.23 – 7.16 (m, 2H), 4.16 (p, *J* = 3.02 Hz, 1H), 3.81 (d, *J* = 1.18 Hz, 3H), 3.01 (tt, *J* = 12.39, 3.35 Hz, 1H), 1.95 – 1.85 (m, 2H), 1.84 – 1.69 (m, 3H), 1.65 – 1.53 (m, 2H), 1.53 – 1.31 (m, 2H).^{13}C NMR (101 MHz, $CDCl_3$) δ 167.29, 152.71, 129.82, 127.89, 127.06, 66.55, 52.07, 40.18, 37.72, 33.51, 32.39, 20.36. HPLC (Chiralpak OD, elute: Hexanes/*i*-PrOH =98/2, detector: 254 nm, flow rate: 1.0 mL/min, 27 ℃).

9h: (1*R*,3*R*)-3-(2-methoxyphenyl)cyclohexan-1-ol. Colorless oil, 93% yield, 97% *ee*, 99/1 *dr*. ^{1}H

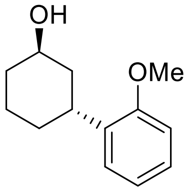

NMR (400 MHz, $CDCl_3$) δ 7.14 – 7.06 (m, 2H), 6.85 (t, J = 6.89 Hz, 1H), 6.78 (d, J = 8.61 Hz, 1H), 4.16 (t, J = 3.05 Hz, 1H), 3.75 (s, 3H), 3.43 – 3.31 (m, 1H), 1.85 (td, J = 3.44, 1.78 Hz, 1H), 1.76 (ddt, J = 14.48, 9.20, 3.50 Hz, 3H), 1.59 (td, J = 13.21, 2.60 Hz, 2H), 1.53 – 1.40 (m, 3H), 1.36 (dd, J = 12.17, 3.52 Hz, 1H). ^{13}C NMR (101 MHz, $CDCl_3$) δ 156.70, 134.95, 126.54, 126.41, 120.32, 110.15, 66.95, 55.15, 39.06, 32.22 (d, J = 5.04 Hz), 30.00, 20.49. HPLC (Chiralpak OD, elute: Hexanes/*i*-PrOH =95/5, detector: 220 nm, flow rate: 1.0 mL/min, 25 ℃).

9i: (1*R*,3*R*)-3-(4-methoxyphenyl)cyclohexan-1-ol. Colorless oil, 97% yield, 99% *ee*, 99/1 *dr*. ^{1}H

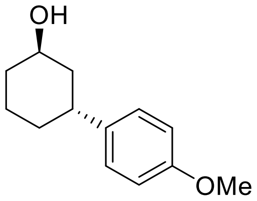

NMR (400 MHz, $CDCl_3$) δ 7.07 (d, J = 8.61 Hz, 2H), 6.77 (d, J = 8.64 Hz, 2H), 4.15 (p, J = 3.04 Hz, 1H), 3.71 (s, 3H), 2.88 (tt, J = 12.41, 3.58 Hz, 1H), 1.90 – 1.79 (m, 2H), 1.81 – 1.68 (m, 3H), 1.63 – 1.52 (m, 2H), 1.48 (d, J = 9.73 Hz, 2H). ^{13}C NMR (101 MHz, $CDCl_3$) δ 157.85, 139.41, 127.87, 113.85, 66.99, 55.35, 40.83, 36.76, 34.13, 32.52, 20.57. HPLC (Chiralpak AD, elute: Hexanes/*i*-PrOH =98/2, detector: 254 nm, flow rate: 1.0 mL/min, 25 ℃).

9j: (1*R*,3*R*)-3-(4-(methylthio)phenyl)cyclohexan-1-ol. White solid, 78% yield, 95% *ee*, 99/1 *dr*. ^{1}H

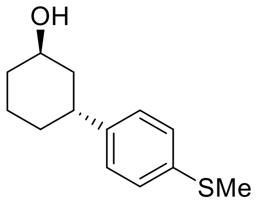

NMR (400 MHz, $CDCl_3$) δ 7.19 (s, 0H), 7.14 (d, *J* = 8.34 Hz, 2H), 7.07 (d, *J* = 8.33 Hz, 2H), 4.16 (p, *J* = 3.02 Hz, 1H), 2.90 (tt, *J* = 12.51, 3.51 Hz, 1H), 2.39 (s, 3H), 1.90 – 1.76 (m, 3H), 1.76 – 1.69 (m, 2H), 1.64 – 1.24 (m, 6H), 1.20 (d, *J* = 11.38 Hz, 1H). ^{13}C NMR (101 MHz, $CDCl_3$) δ 144.37, 135.42, 127.58,

127.22, 66.86, 40.53, 37.12, 33.83, 32.49, 20.47, 16.37. HPLC (Chiralpak AD, elute: Hexanes/*i*-PrOH =98/2, detector: 254 nm, flow rate: 1.0 mL/min, 27 ℃).

9k: (1*R*,3*R*)-3-(o-tolyl)cyclohexan-1-ol. Colorless oil, 91% yield, 95% *ee*, 99/1 *dr*. ^{1}H NMR (400 MHz, $CDCl_3$) δ 7.14 – 6.96 (m, 4H), 4.16 (p, *J* = 2.95 Hz, 1H), 3.17 (tt, *J* = 12.19, 3.16 Hz, 1H), 2.28 (s, 3H), 1.78 (ddd, *J* = 23.57, 13.31, 3.36 Hz, 4H), 1.65 – 1.43 (m, 4H), 1.36 (qd, *J* = 13.07, 3.52 Hz, 1H). ^{13}C NMR (101 MHz, $CDCl_3$) δ 130.42, 126.22, 125.75, 125.45, 67.07, 39.90, 33.13, 32.97, 32.58, 20.86, 19.43. HPLC (Chiralpak AD, elute: Hexanes/*i*-PrOH =98/2, detector: 220 nm, flow rate: 1.0 mL/min, 25 ℃).

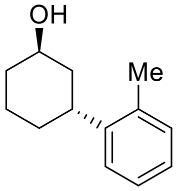

9l: (1*R*,3*R*)-3-(m-tolyl)cyclohexan-1-ol.White solid, 98% yield, 96% *ee*, 99/1 *dr*. ^{1}H NMR (400 MHz, $CDCl_3$) δ 7.11 (t, *J* = 7.50 Hz, 1H), 6.98 – 6.89 (m, 3H), 4.14 (p, *J* = 3.03 Hz, 1H), 2.89 (tt, *J* = 12.39, 3.48 Hz, 1H), 2.25 (s, 3H), 1.92 – 1.68 (m, 5H), 1.66 – 1.30 (m, 4H). ^{13}C NMR (101 MHz, $CDCl_3$) δ 147.17, 137.95, 128.36, 127.88, 126.79, 124.01, 66.92, 40.62, 37.56, 33.83, 32.47, 21.59, 20.54. HPLC (Chiralpak IC, elute: Hexanes/*i*-PrOH =98/2, detector: 220 nm, flow rate: 1.0 mL/min, 25 ℃).

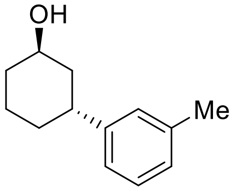

9m: (1*R*,3*R*)-3-(p-tolyl)cyclohexan-1-ol.White solid, 95% yield, 98% *ee*, 99/1 *dr*. ^{1}H NMR (400 MHz, $CDCl_3$) δ 7.03 (s, 4H), 4.13 (p, *J* = 3.03 Hz, 0.87H), 3.70 – 3.58 (m, 0.13H), 2.89 (tt, *J* = 12.38, 3.53 Hz, 0.90H), 2.46 (tt, *J* = 12.16, 3.37 Hz, 0.14H), 2.24 (s, 3H), 1.90 – 1.78 (m, 2H), 1.78 – 1.67 (m, 2H), 1.66 – 1.51 (m, 3H), 1.53 – 1.38 (m, 1H), 1.40 – 1.28 (m, 1H).^{13}C NMR (101 MHz, $CDCl_3$) δ 144.21, 135.48, 129.18 (d, *J* = 5.13 Hz), 126.82 (d, *J* = 15.25 Hz), 66.93, 40.67, 37.18, 33.96, 32.48, 20.55. HPLC (Chiralpak AD, elute: Hexanes/*i*-PrOH =98/2, detector: 254 nm, flow rate: 1.0 mL/min, 25 ℃).

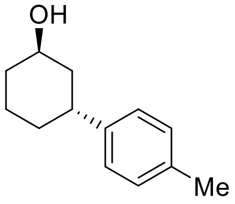

9n: (1*R*,3*R*)-3-(4-butylphenyl)cyclohexan-1-ol. Colorless oil,74% yield, 96% *ee*, 82/18 *dr*. ^{1}H NMR (400 MHz, $CDCl_3$) δ 7.08 – 6.99 (m, 4H), 4.12 (p, J = 3.09 Hz, 1H), 2.96 – 2.85 (m, 1H), 2.49 (td, J = 7.94, 2.67 Hz, 2H), 1.90 – 1.68 (m, 4H), 1.60 – 1.20 (m, 8H), 1.20 – 1.08 (m, 1H), 0.84 (td, J = 7.30, 3.09 Hz, 3H). ^{13}C NMR (101 MHz, $CDCl_3$) δ144.64 – 143.27 (m), 140.46 (d, J = 21.03 Hz), 128.37 (d, J = 5.51 Hz), 126.67 (d, J = 14.33 Hz), 67.05, 66.71 (d, J = 4.82 Hz), 43.26, 42.37 (d, J = 1.77 Hz), 40.60, 37.07, 35.24, 34.02 – 33.38 (m), 24.52, 22.46, 21.02, 20.48 (d, J = 1.81 Hz), 14.18, 13.99. HPLC (Chiralpak IC, elute: Hexanes/i-PrOH =99/1, detector: 220 nm, flow rate: 1.0 mL/min, 25 ℃).

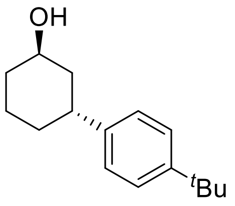

9o: (1*R*,3*R*)-3-(thiophen-3-yl)cyclohexan-1-ol. Colorless oil, 90% yield, 99% *ee*, 96/4 *dr*. ^{1}H NMR (400 MHz, $CDCl_3$) δ 7.22 – 7.15 (m, 1H), 6.90 (s, 1H), 6.88 (dt, *J* = 2.98, 1.16 Hz, 1H), 4.12 (p, *J* = 3.29 Hz, 1H), 3.05 (tt, *J* = 11.56, 3.67 Hz, 1H), 1.96 – 1.86 (m, 2H), 1.74 – 1.63 (m, 2H), 1.63 – 1.44 (m, 4H), 1.44 – 1.29 (m, 1H). ^{13}C NMR (101 MHz, $CDCl_3$) δ 148.12, 127.13, 125.28, 118.80 (d, *J* = 2.84 Hz), 67.20, 66.79, 40.40, 33.45, 33.16,

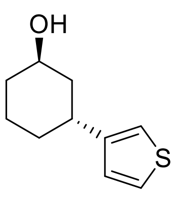

32.81, 20.34. HPLC (Chiralpak AD, elute: Hexanes/*i*-PrOH =98/2, detector: 220 nm, flow rate: 1.0 mL/min, 27 ℃).

9p: *tert*-butyl 3-((1*R*,3*R*)-3-hydroxycyclohexyl)-1H-indole-1-carboxylate. White solid, 96% yield, 99% *ee*, 99/1 *dr*. ^{1}H NMR (400 MHz, $CDCl_3$) δ 8.02 (d, *J* = 8.25 Hz, 1H), 7.53 – 7.47 (m, 1H), 7.29 – 7.16 (m, 2H), 7.16 – 7.03 (m, 1H), 4.14 (p, *J* = 3.15 Hz, 1H), 3.19 (tt, *J* = 11.74, 3.56 Hz, 1H), 2.10 – 1.92 (m, 2H), 1.88 – 1.68 (m, 3H), 1.63 (ddd, *J* = 14.15, 11.92, 2.73 Hz, 1H), 1.57 (s, 9H), 1.54 – 1.31 (m, 3H). ^{13}C NMR (101 MHz, $CDCl_3$) δ 150.04, 135.68, 130.16, 126.54, 124.25, 122.26, 121.06, 119.46, 115.39, 83.41, 66.71, 39.38, 32.89, 32.65, 28.67, 28.32, 20.47.HPLC (Chiralpak AS, elute: Hexanes/*i*-PrOH =98/2, detector: 254 nm, flow rate: 1.0 mL/min, 27 ℃).

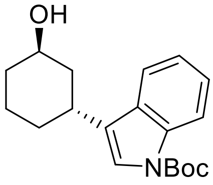

9q: (1*R*,3*R*)-3-([1,1'-biphenyl]-4-yl)cyclohexan-1-ol. White solid, 92% yield, 99% *ee*, 99/1 *dr*. ^{1}H NMR (400 MHz, $CDCl_3$) δ 7.53 – 7.47 (m, 2H), 7.47 – 7.42 (m, 2H), 7.37 – 7.30 (m, 2H), 7.27 – 7.19 (m, 3H), 4.17 (p, *J* = 3.02 Hz, 1H), 2.98 (tt, *J* = 12.35, 3.50 Hz, 1H), 1.90 (dddt, *J* = 13.79, 10.23, 3.52, 1.95 Hz, 2H), 1.84 (td, *J* = 3.35, 1.47 Hz, 1H), 1.76 (ddt, *J* = 14.02, 12.09, 3.49 Hz, 2H), 1.70 – 1.32 (m, 5H). ^{13}C NMR (101 MHz, $CDCl_3$) δ 146.33, 141.18, 139.00, 128.82, 127.47, 127.17 (d, *J* = 10.90 Hz), 66.91, 40.59, 37.32, 33.86, 32.53, 20.54.HPLC (Chiralpak IC, elute: Hexanes/*i*-PrOH =98/2, detector: 254 nm, flow rate: 1.0 mL/min, 27 ℃).

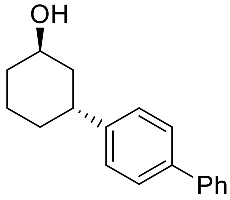

9r: (1*R*,3*R*)-3-(naphthalen-1-yl)cyclohexan-1-ol. White solid, 85% yield, 93% *ee*, 99/1 *dr*. ^{1}H NMR (400 MHz, $CDCl_3$) δ 8.13 (d, *J* = 8.39 Hz, 1H), 7.78 (dd, *J* = 7.95, 1.63 Hz, 1H), 7.63 (dt, *J* = 8.17, 1.09 Hz, 1H), 7.52 – 7.27 (m, 4H), 4.25 (p, *J* = 3.05 Hz, 1H), 3.83 (tt, *J* = 12.23, 3.15 Hz, 1H), 2.13 – 2.03 (m, 1H), 1.94 – 1.74 (m, 3H), 1.58 – 1.39 (m, 5H). ^{13}C NMR (101 MHz, $CDCl_3$) δ 171.36, 143.11, 134.05, 131.51, 129.01, 126.55, 125.90, 125.71, 125.47, 123.43, 122.42, 67.31, 60.56, 40.61, 33.50, 32.90, 29.84, 22.92 – 22.70 (m), 21.08, 14.30 (d, *J* = 6.26 Hz), 11.57. HPLC (Chiralpak AD, elute: Hexanes/*i*-PrOH =98/2, detector: 220 nm, flow rate: 1.0 mL/min, 27 ℃).

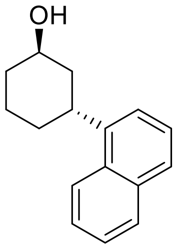

9s: (1*R*,3*R*)-3-(phenanthren-9-yl)cyclohexan-1-ol. White solid, 73% yield, 97% *ee*, 99/1 *dr*. ^{1}H NMR (400 MHz, $CDCl_3$) δ 8.61 – 8.54 (m, 1H), 8.52 – 8.46 (m, 1H), 8.14 (dt, *J* = 6.96, 2.71 Hz, 1H), 7.68 (dd, *J* = 6.18, 3.13 Hz, 1H), 7.53 – 7.38 (m, 5H), 4.16 (p, *J* = 3.00 Hz, 1H), 3.75 (tt, *J* = 12.03, 2.96 Hz, 1H), 2.10 – 2.02 (m, 1H), 2.02 – 1.89 (m, 3H), 1.85 (dt, *J* = 13.33, 3.52 Hz, 1H), 1.80 – 1.72 (m, 1H), 1.57 (t, *J* = 3.41 Hz, 1H), 1.55 – 1.38 (m, 2H). ^{13}C NMR (101 MHz, $CDCl_3$) δ 141.13, 131.95, 130.79 129.44, 128.30, 126.59 (d, *J* = 2.46 Hz), 126.05 (d, *J* = 9.82 Hz), 124.01, 123.32, 122.90, 122.44, 67.11, 40.25, 33.30, 32.84, 32.17, 21.03. HPLC (Chiralpak IC, elute: Hexanes/*i*-PrOH =98/2, detector: 220 nm, flow rate: 1.0 mL/min, 27 ℃).

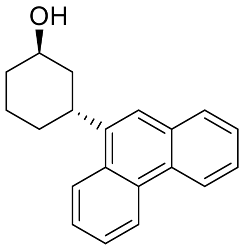

9t: *tert*-butyl (2*R*,4*R*)-4-hydroxy-2-phenylpiperidine-1-carboxylate. White solid, 76% yield, 99% *ee*, 89/11 *dr*. ^{1}H NMR (400 MHz, $CDCl_3$) δ 7.26 (t, *J* = 7.62 Hz, 2H), 7.21 – 7.12 (m, 3H), 5.22 – 5.15 (m, 1H), 4.11 – 4.00 (m, 1H), 3.95 (m, *J* = 13.74, 5.56, 2.67 Hz, 1H), 3.32 – 3.18 (m, 1H), 2.40 – 2.24 (m, 1H), 2.07 (m, *J* = 14.47, 6.61, 3.38 Hz, 1H), 1.85 – 1.63 (m, 2H), 1.62 – 1.52 (m, 1H), 1.40 (s, 1H), 1.32 (s, 9H), 1.23 – 1.14 (m, 1H). ^{13}C NMR (101 MHz, $CDCl_3$) δ 155.68, 142.04, 128.75, 126.74, 126.63, 126.18, 125.55, 79.99, 65.07, 52.16, 36.01, 35.52, 32.25, 28.42.HPLC (Chiralpak OD+OD, elute: Hexanes/*i*-PrOH =98/2, detector: 254 nm, flow rate: 1.0 mL/min, 25 ℃).

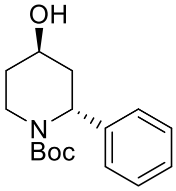

9u: (1*R*,3*R*)-3-phenylcyclopentan-1-ol. Colorless oil, 75% yield, 97% *ee*, 86/14 *dr*. ^{1}H NMR (400 MHz, $CDCl_3$) δ 7.29 – 7.15 (m, 4H), 7.15 – 7.04 (m, 1H), 4.37 (qdd, *J* = 5.89, 3.67, 1.85 Hz, 1H), 2.96 (tt, *J* = 9.88, 7.78 Hz, 1H), 2.45 – 2.33 (m, 1H), 2.04 – 1.93 (m, 1H), 1.91 – 1.78 (m, 2H), 1.77 – 1.66 (m, 2H), 1.64 – 1.52 (m, 1H). ^{13}C NMR (101 MHz, $CDCl_3$) δ 145.78, 128.44 (d, *J* = 5.12 Hz), 127.20 (t, *J* = 6.95 Hz), 126.03 (d, *J* = 5.34 Hz), 73.69 (d, *J* = 5.45 Hz), 44.33 (t, *J* = 4.11 Hz), 44.08 (d, *J* = 4.90 Hz), 36.08 (d, *J* = 5.45 Hz), 32.73 (d, *J* = 5.02 Hz). HPLC (Chiralpak OZ+OB, elute: Hexanes/*i*-PrOH =98/2, detector: 220 nm, flow rate: 1.0 mL/min, 25 ℃).

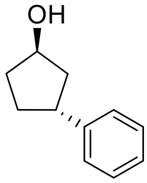

9v: (1*R*,3*R*)-3-phenylcycloheptan-1-ol. Colorless oil, 92% yield, 97% *ee*, 98/2 *dr*. ^{1}H NMR (400 MHz, $CDCl_3$) δ 7.24 – 7.16 (m, 2H), 7.16 – 7.05 (m, 3H), 4.09 (dq, *J* = 8.86, 4.60 Hz, 1H), 2.95 (tt, *J* = 10.20, 3.23 Hz, 1H), 2.03 (ddd, *J* = 13.92, 9.68, 4.07 Hz, 1H), 1.98 – 1.82 (m, 3H), 1.81 – 1.71 (m, 2H), 1.70 – 1.42 (m, 3H), 1.41 – 1.28 (m, 2H). ^{13}C NMR (101 MHz, $CDCl_3$) δ 149.63, 128.56, 126.80, 125.79, 70.20, 45.24, 39.61, 37.34, 37.02, 29.13, 23.63. HPLC (Chiralpak AD, elute: Hexanes/*i*-PrOH =98/2, detector: 254 nm, flow rate: 1.0 mL/min, 27 ℃).

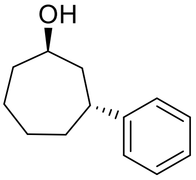

9w: (1*R*,3*R*)-3-(m-tolyl)cycloheptan-1-ol. Colorless oil, 95% yield, 99% *ee*, 99/1 *dr*. ^{1}H NMR (400 MHz, $CDCl_3$) δ 7.09 (t, *J* = 7.51 Hz, 1H), 6.98 – 6.85 (m, 3H), 4.07 (dq, *J* = 8.86, 4.57 Hz, 1H), 2.90 (tt, *J* = 10.02, 3.21 Hz, 1H), 2.25 (s, 3H), 2.00 (ddd, *J* = 13.97, 9.70, 4.09 Hz, 1H), 1.95 – 1.80 (m, 3H), 1.80 – 1.69 (m, 2H), 1.68 – 1.40 (m, 4H), 1.40 – 1.25 (m, 1H). ^{13}C NMR (101 MHz, $CDCl_3$) δ 149.60, 138.01, 128.44, 127.64, 126.49, 123.73, 70.17, 45.24, 39.52, 37.31, 36.97, 29.14, 23.63, 21.59. HPLC (Chiralpak AS, elute: Hexanes/*i*-PrOH =98/2, detector: 254 nm, flow rate: 1.0 mL/min, 27 ℃).

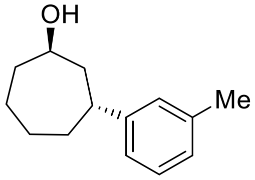

9x: (1*R*,3*R*)-3-(p-tolyl)cycloheptan-1-ol. Colorless oil, 91% yield, 99% *ee*, 99/1 *dr*. ^{1}H NMR (400 MHz, $CDCl_3$) δ 7.01 (s, 4H), 4.15 – 3.97 (m, 1H), 2.98 – 2.79 (m, 1H), 2.23 (s, 3H), 2.00 (m, *J* = 14.67, 9.65, 4.05 Hz, 1H), 1.95 – 1.69 (m, 5H), 1.68 – 1.40 (m, 4H), 1.39 – 1.24 (m, 1H). ^{13}C NMR (101 MHz, $CDCl_3$) δ 146.65, 135.16, 129.20, 126.64, 70.18, 45.32, 39.17, 37.40, 36.99, 29.08, 23.67, 21.07. HPLC (Chiralpak IC, elute: Hexanes/*i*-PrOH =98/2, detector: 254 nm, flow rate: 1.0 mL/min, 27 ℃).

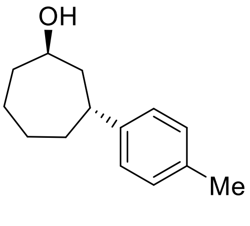

Figure 1. IR spectra of **2**, **4**, and catalyst **5**.

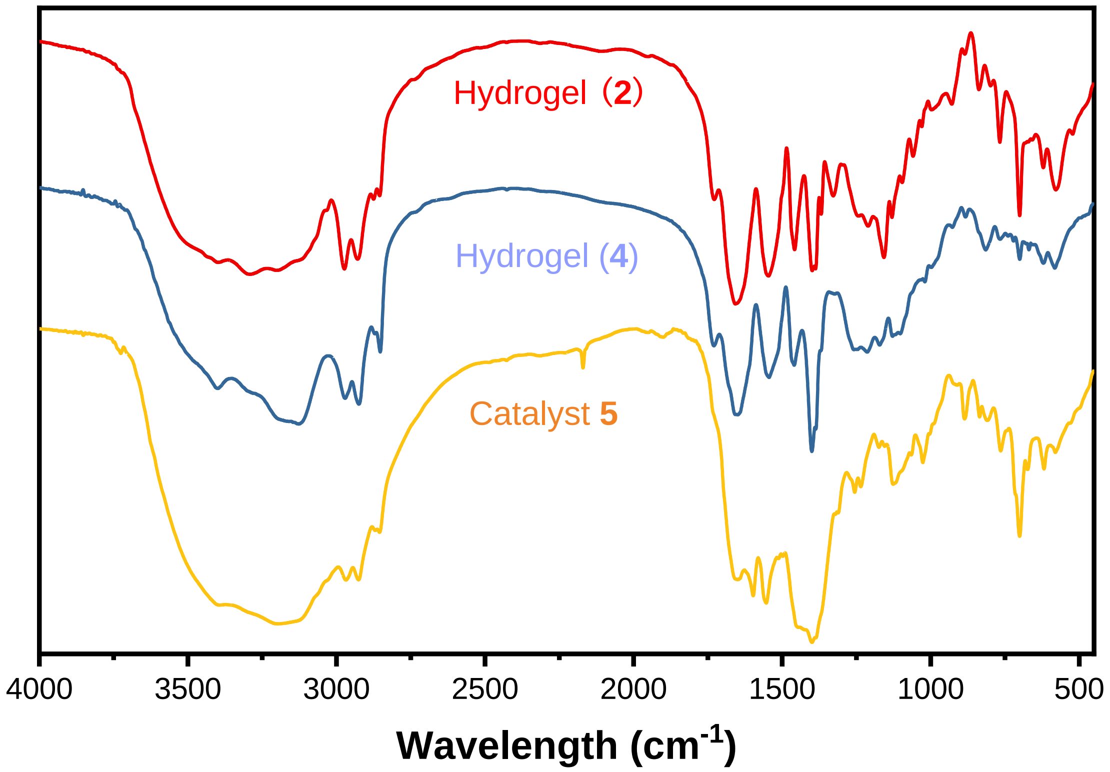

Figure S2. ^{1}H NMR spectra **2** and **4** (DMSO-d_6).

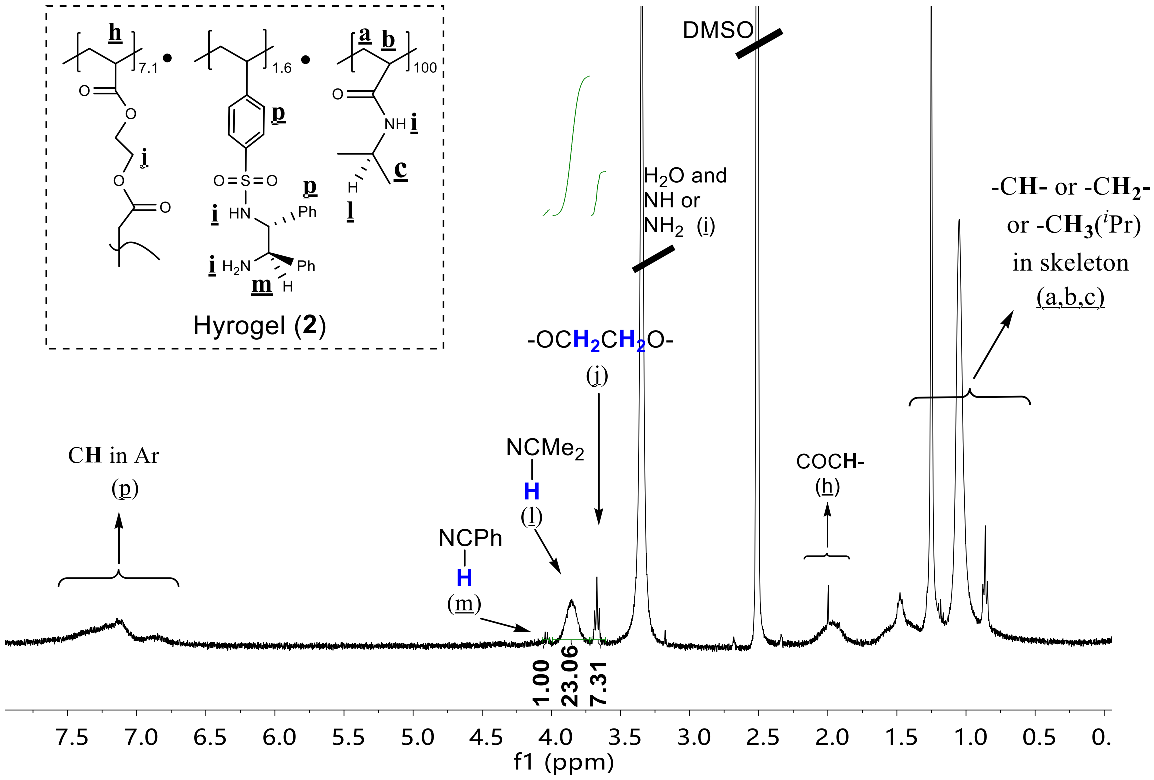

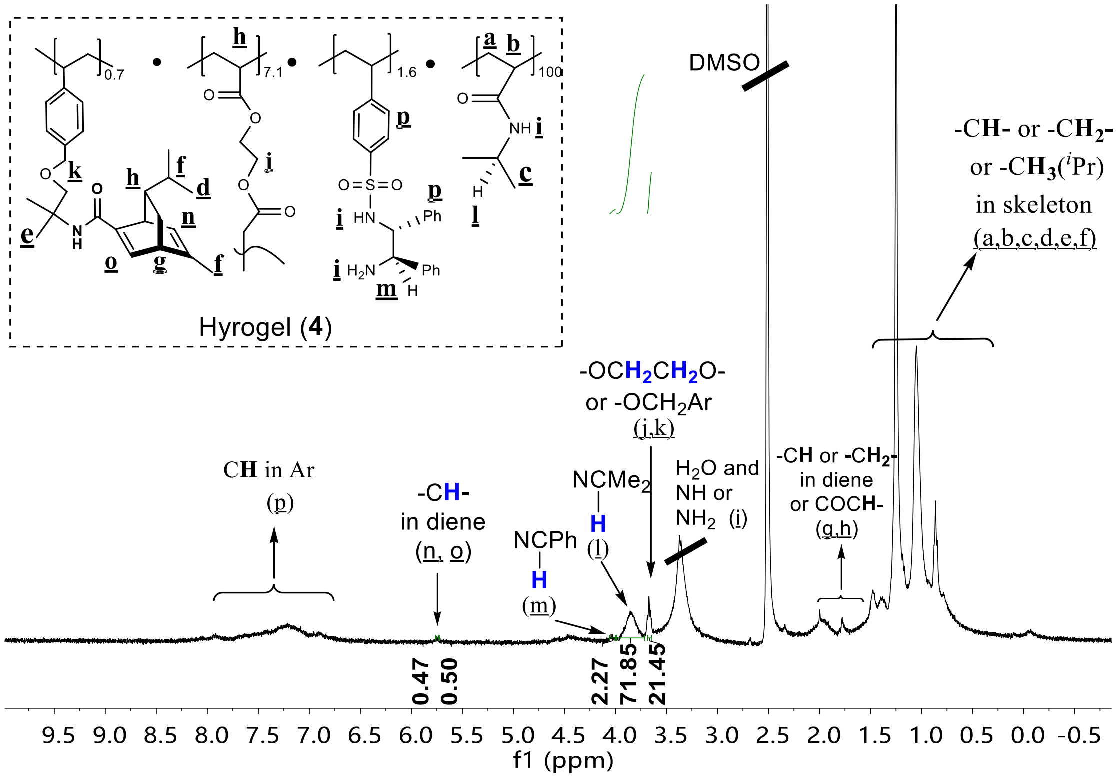

Figure S3. Temperature-dependent transmittance and the dispersive situations of **4**. (a) The turbidity measurement of **4** with a 0.02 M concentration in pure water was performed on a custom-modified Tepper turbidity photometer TP1-D at a wavelength of 670 nm, a cell path length of 10 mm, and magnetic stirring. The heating program started at a low temperature of 20 ℃, and it was heated to 60 ℃ at a constant cooling rate of 1.0 ℃/min. (b) Temperature-dependent transmittance for the determination of the volume phase transition temperature (VPTT) as the temperature corresponding to the transmittance decrease at the wavelength of 680 nm). (c) The dispersive situations in the water.

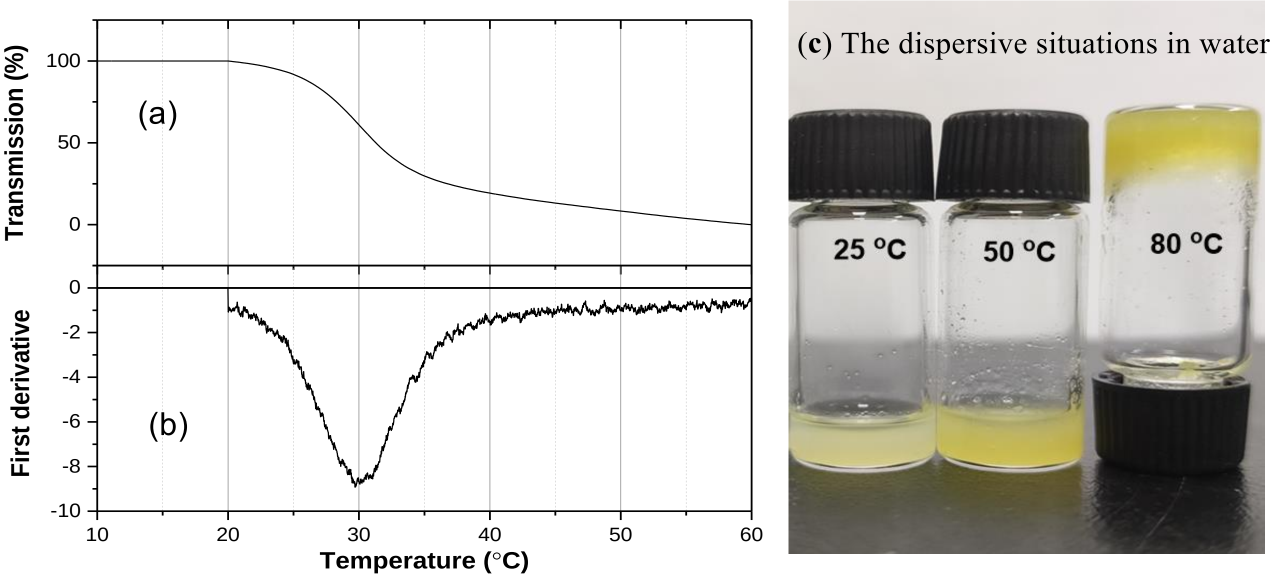

Figure S4. The dispersive situations of catalyst **5** in the co-solvent system (H_2O/dioxane (v:v = 2:5)).

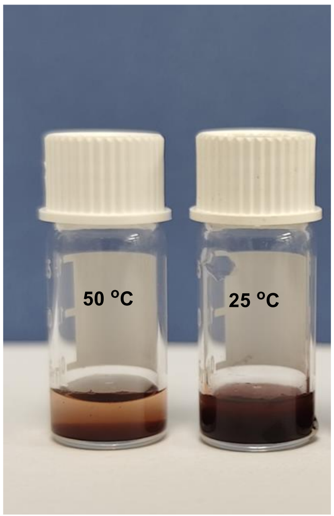

Figure S5. Morphology and rheology investigations of catalyst **5** at 25 ℃ and 50 ℃. (a) The scanning electron microscopy (SEM) images of catalyst **5**. (b) The viscosity curves obtained by using the different concentrations of catalyst **5** in the H_2O/dioxane (v:v = 2/5) at 25 ℃ and 50 ℃. (Rheological measurements were performed on an Anton Paar MCR-102 (Austria) using a PP50 Parallel Plate measuring system with 1000 (1/s) shear rate and 1.01 (Pa) shear stress in the range of 2.0 mg/mL and 6.5 mg/mL concentrations).

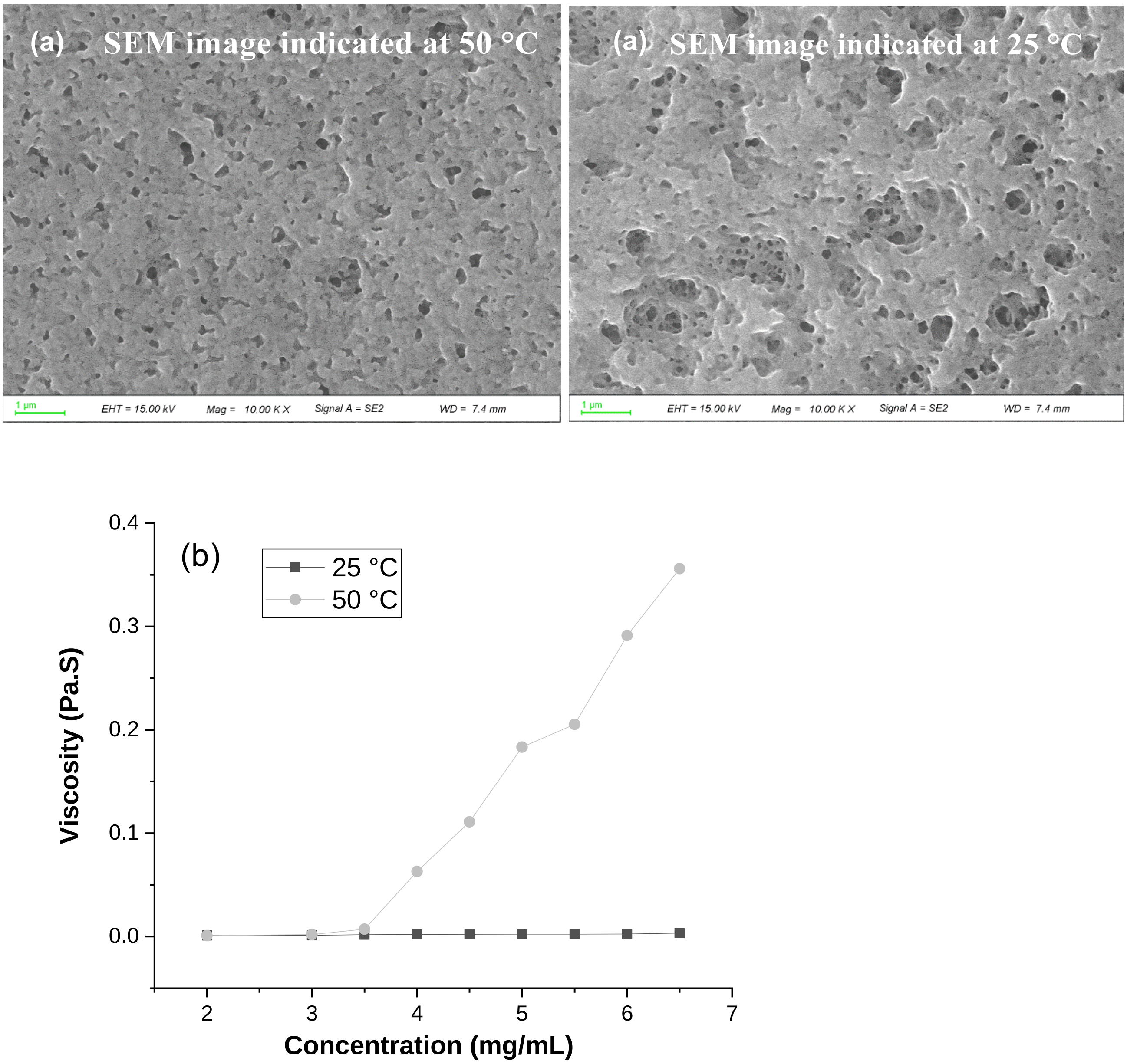

Figure S6. Average hydrodynamic diameters distribution measurement of the recycled catalyst **5**.

Run 1.

Results (25 °C for the first run)

			Size (d.nm):	% Intensity:	St Dev (d.nm):
Z-Average (d.nm):	210.5	Peak 1:	258.9	100.0	100.4
PdI:	0.183	Peak 2:	0.000	0.0	0.000
Intercept:	0.914	Peak 3:	0.000	0.0	0.000
Result quality :	Good				

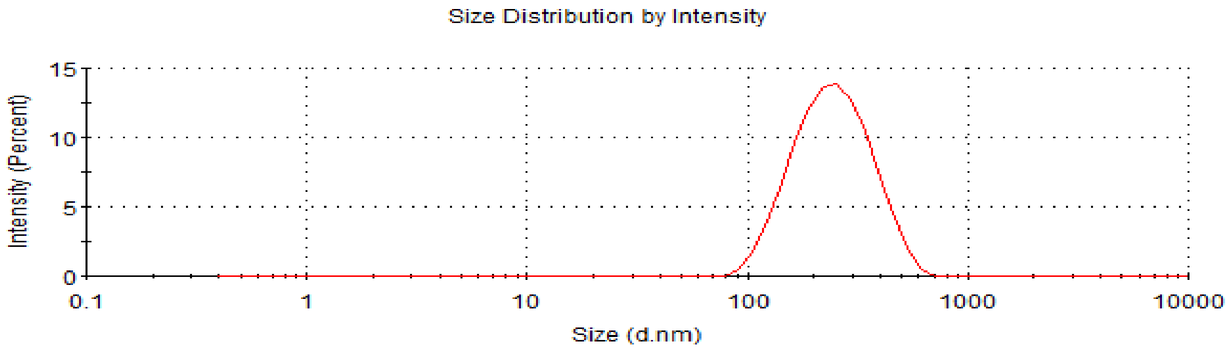

Results (50 °C for the first run)

			Size (d.nm):	% Intensity:	St Dev (d.nm):
Z-Average (d.nm):	104.8	Peak 1:	119.1	96.0	52.25
PdI:	0.229	Peak 2:	4226	4.0	994.4
Intercept:	0.894	Peak 3:	0.000	0.0	0.000
Result quality :	Good				

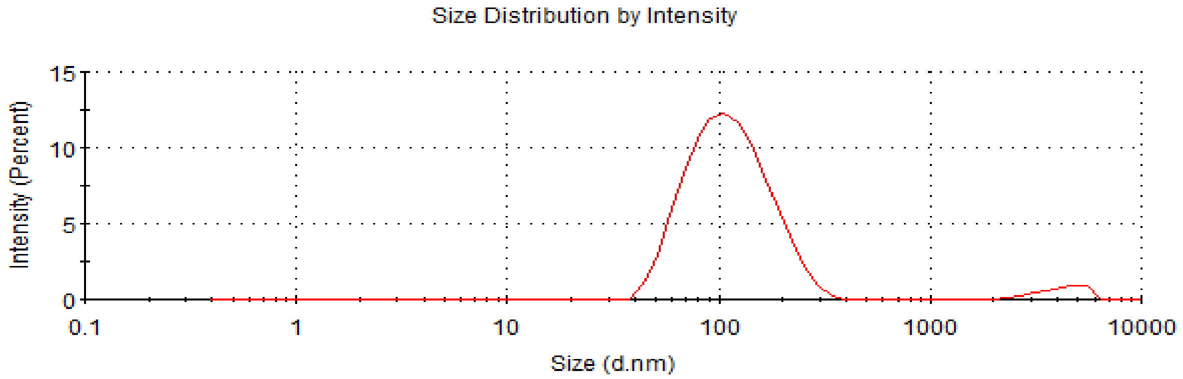

Run 2.

Results (25 °C for the second run)

			Size (d.nm):	% Intensity:	St Dev (d.nm):
Z-Average (d.nm):	207.9	Peak 1:	266.5	100.0	134.1
PdI:	0.204	Peak 2:	0.000	0.0	0.000
Intercept:	0.911	Peak 3:	0.000	0.0	0.000
Result quality :	Good				

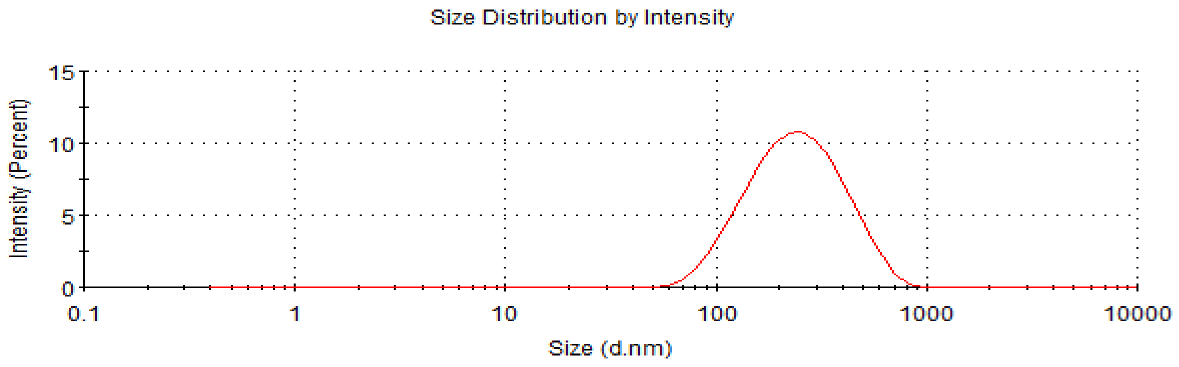

Results (50 °C for the second run)

			Size (d.nm):	% Intensity:	St Dev (d.nm):
Z-Average (d.nm):	105.3	Peak 1:	145.3	100.0	91.37
PdI:	0.230	Peak 2:	0.000	0.0	0.000
Intercept:	0.898	Peak 3:	0.000	0.0	0.000
Result quality :	Good				

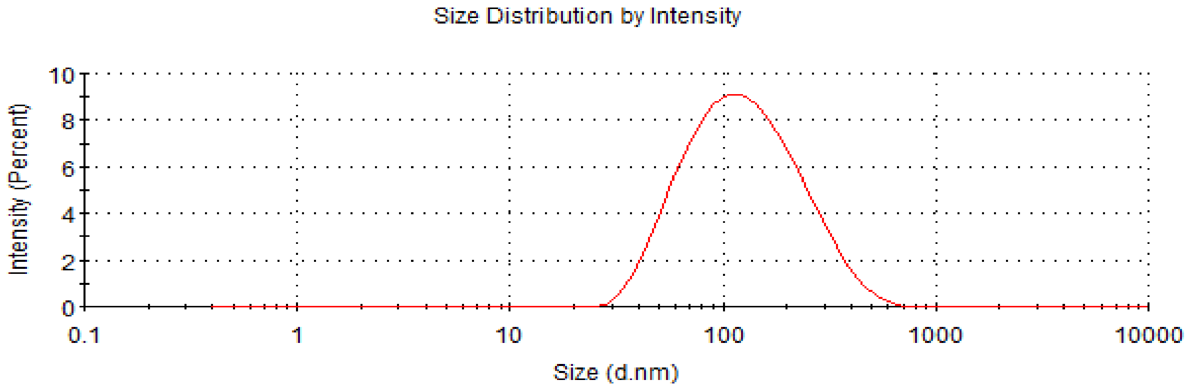

Run 3.

Results (25 °C for the third run)

			Size (d.nm):	% Intensity:	St Dev (d.nm):
Z-Average (d.nm):	208.4	Peak 1:	241.7	98.6	96.56
PdI:	0.214	Peak 2:	5016	1.4	600.1
Intercept:	0.906	Peak 3:	0.000	0.0	0.000
Result quality :	Good				

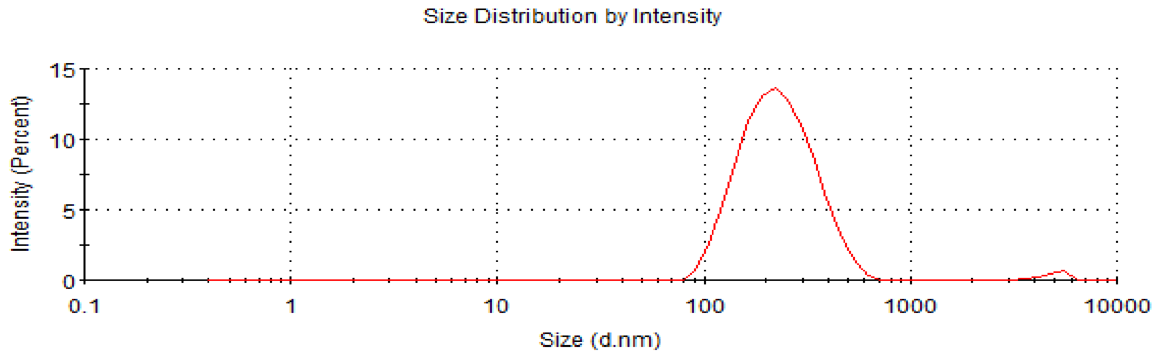

Results (50 °C for the third run)

			Size (d.nm):	% Intensity:	St Dev (d.nm):
Z-Average (d.nm):	104.4	Peak 1:	125.2	98.1	58.95
PdI:	0.215	Peak 2:	4576	1.9	837.0
Intercept:	0.892	Peak 3:	0.000	0.0	0.000
Result quality :	Good				

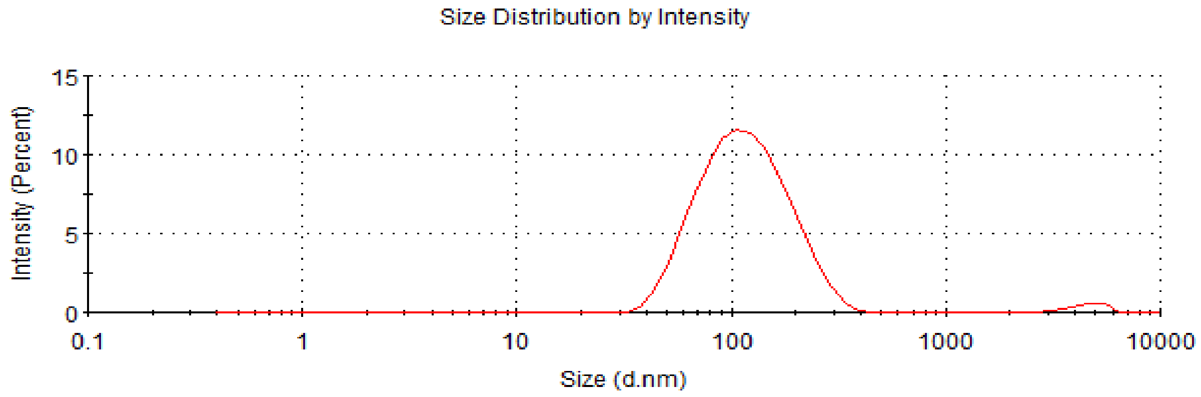

Run 4.

Results (25 °C for the fourth run)

			Size (d.nm):	% Intensity:	St Dev (d.nm):
Z-Average (d.nm):	206.4	Peak 1:	275.6	100.0	149.9
PdI:	0.229	Peak 2:	0.000	0.0	0.000
Intercept:	0.907	Peak 3:	0.000	0.0	0.000
Result quality :	Good				

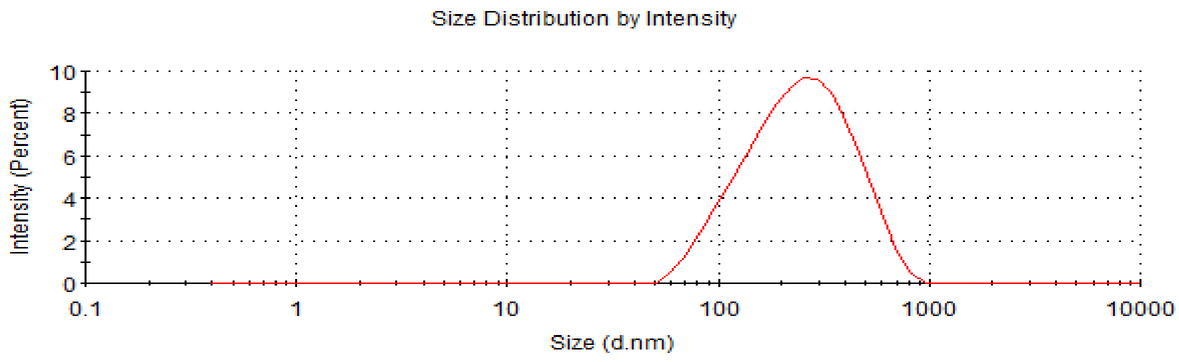

Results (50 °C for the fourth run)

			Size (d.nm):	% Intensity:	St Dev (d.nm):
Z-Average (d.nm):	104.0	Peak 1:	124.4	97.6	59.85
PdI:	0.229	Peak 2:	4590	2.4	832.1
Intercept:	0.891	Peak 3:	0.000	0.0	0.000
Result quality :	Good				

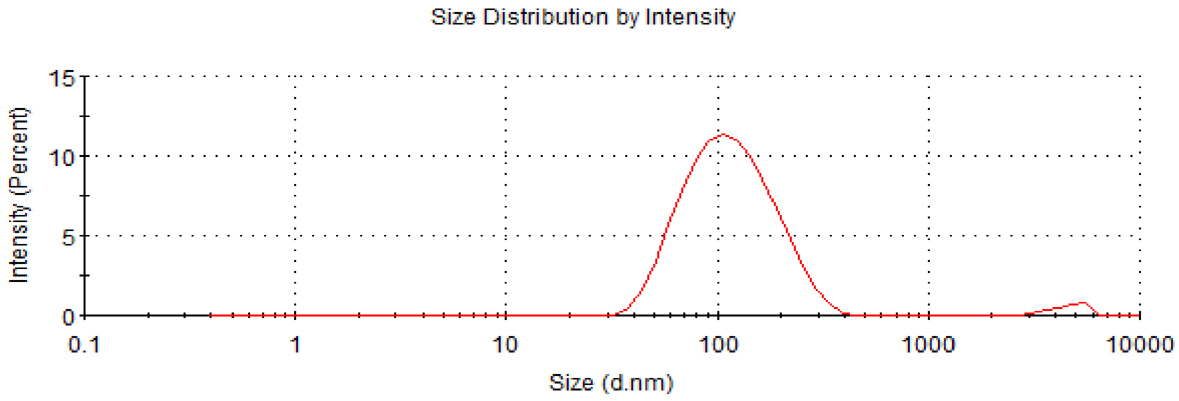

Run 5.

Results (25 °C for the fifth run)

			Size (d.nm):	% Intensity:	St Dev (d.nm):
Z-Average (d.nm):	207.6	Peak 1:	260.6	100.0	108.6
PdI:	0.209	Peak 2:	0.000	0.0	0.000
Intercept:	0.903	Peak 3:	0.000	0.0	0.000
Result quality :	Good				

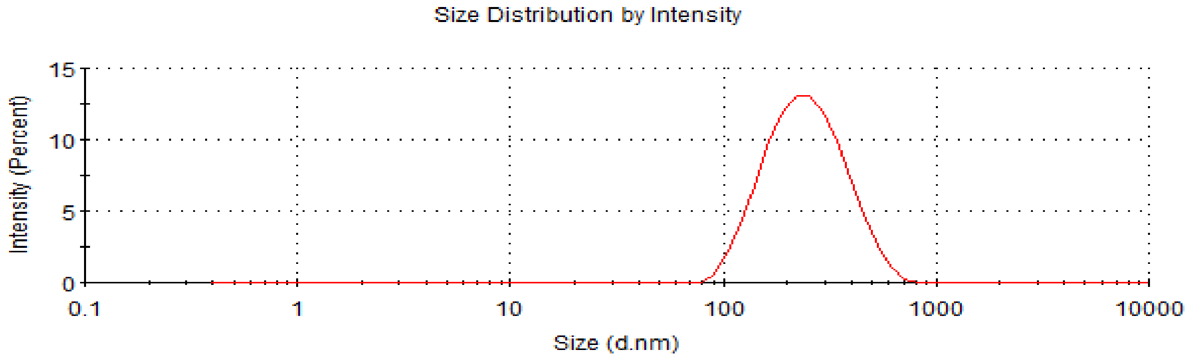

Results (50 °C for the fifth run)

			Size (d.nm):	% Intensity:	St Dev (d.nm):
Z-Average (d.nm):	104.4	Peak 1:	131.4	98.9	64.63
PdI:	0.211	Peak 2:	20.14	1.1	3.734
Intercept:	0.890	Peak 3:	0.000	0.0	0.000
Result quality :	Good				

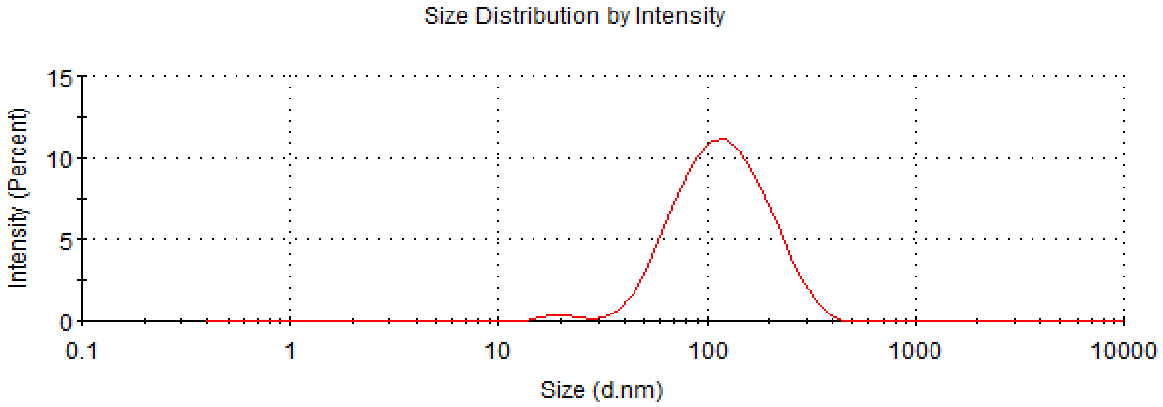

Figure 7. Solid-state ^{13}C MAS NMR spectra of **2, 4,** and catalyst **5.**

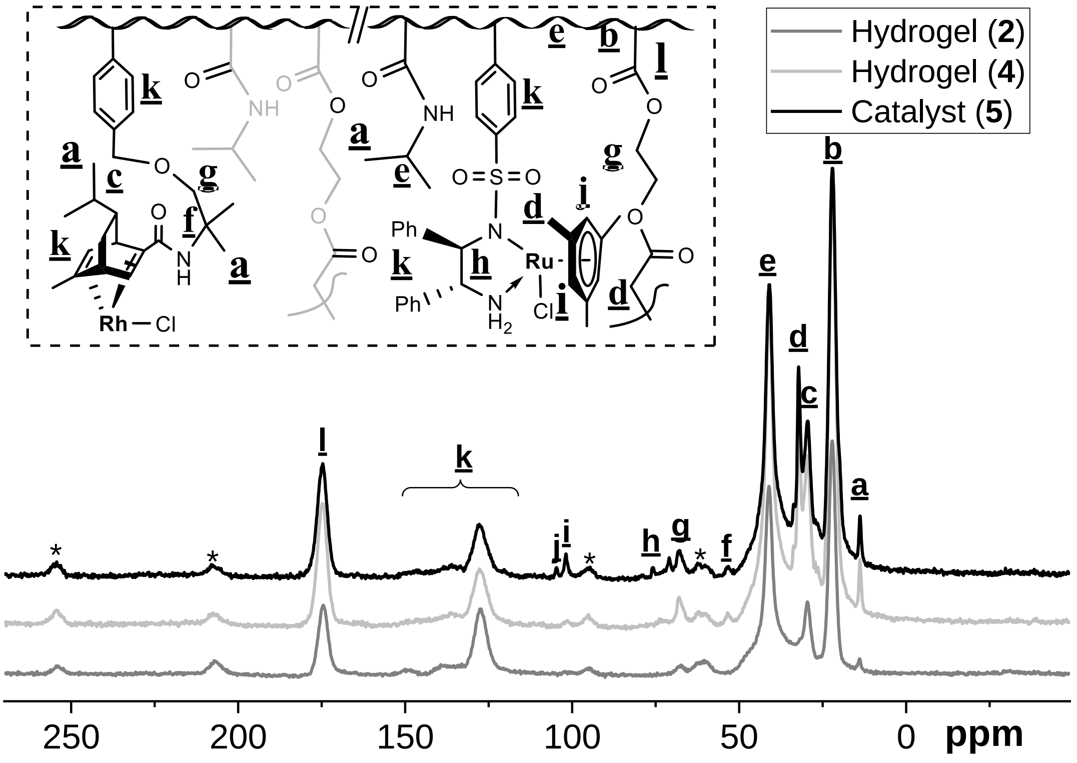

Table S1. Optimizing reaction conditions for the 1,4-addition reaction.[a]

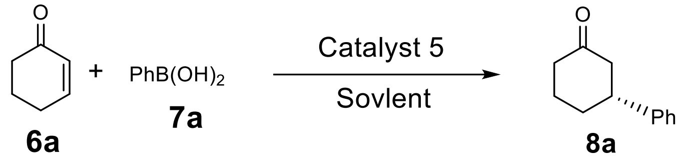

Entry	Solvent	Additive	℃	Time (h)	Yield (%)	*ee* (%)
1	dioxane	/	50	4	99	95
2	H_2O/dioxane (1:1)	/	50	4	98	93
3	H_2O/dioxane (1:2)	/	50	4	98	90
4	H_2O/dioxane (2:5)	/	50	4	97	95
5	H_2O/dioxane (1:3)	/	50	4	98	86
6	H_2O/dioxane (2:5)	/	30	4	81	93
7	H_2O/dioxane (2:5)	/	45	4	92	92
8	H_2O/dioxane (2:5)	/	55	4	99	91
9[b]	H_2O/dioxane (2:5)	HCOOH/Et_3N	50	4	73	81
9[b]	H_2O/dioxane (2:5)	HCOONa	50	4	95	95

[a] Reaction conditions: Catalyst (2.50 mol% of Rh-loadings), **6a** (0.10 mmol), **7a** (0.15 mmol), and 2.0 mL of solvent under the Ar atmosphere. [b] Data was obtained in the presence of 10 equivalents of HCOONa.

Table S2. Optimizing reaction conditions for the ATH transformation.[a]

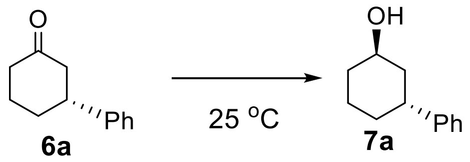

**Entry**	**H-resource, Solvent**	**°C**	**Yield (%)**	***ee* (%)**	***dr* (%)**
1	HCO_2Na, H_2O/dioxane (2:5)	20	89	92	98:2
2	HCO_2Na, H_2O/dioxane (2:5)	25	96	92	98:2
3	HCO_2Na, H_2O/dioxane (2:5)	30	96	92	97:3
4	HCO_2Na, H_2O/dioxane (2:5)	40	97	92	94:6
5	HCO_2Na, H_2O/dioxane (2:5)	50	99	92	91:9

[a] Reaction conditions: Catalyst (1.84 mol% of Ru-loadings), (*R*)-**8a** (92% *ee*, 0.10 mmol), HCO_2Na (1.0 mmol) in 2.0 mL of H_2O/dioxane (v:v = 2/5) under the Ar atmosphere, 16 h.

Figure S8. HPLC analyses for chiral products.

(*R*,*R*)-9a: (1*R*,3*R*)-3-phenylcyclohexan-1-ol (HPLC: Chiracel IC, detected at 254 nm, eluent: n-hexane/2-propanol = 95/5, flow rate = 1.0mL/min, 25°C).

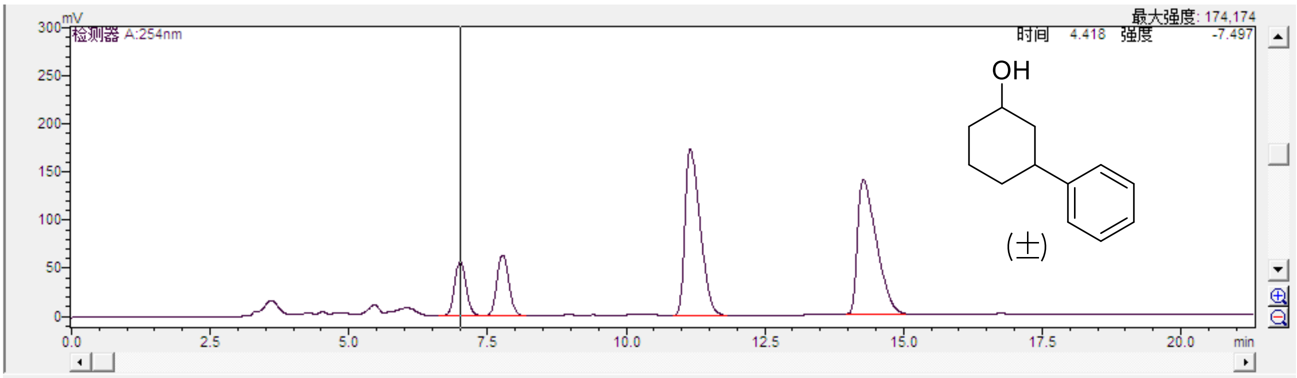

化合物表视图

ID#	名称	保留时间	峰#	面积	高度	面积%
1	RT7.005	7.005	1	817916	55914	9.7598
2	RT7.767	7.767	2	946319	62842	11.2919
3	RT11.154	11.154	3	3306506	173204	39.4548
4	RT14.273	14.273	4	3309743	140034	39.4935

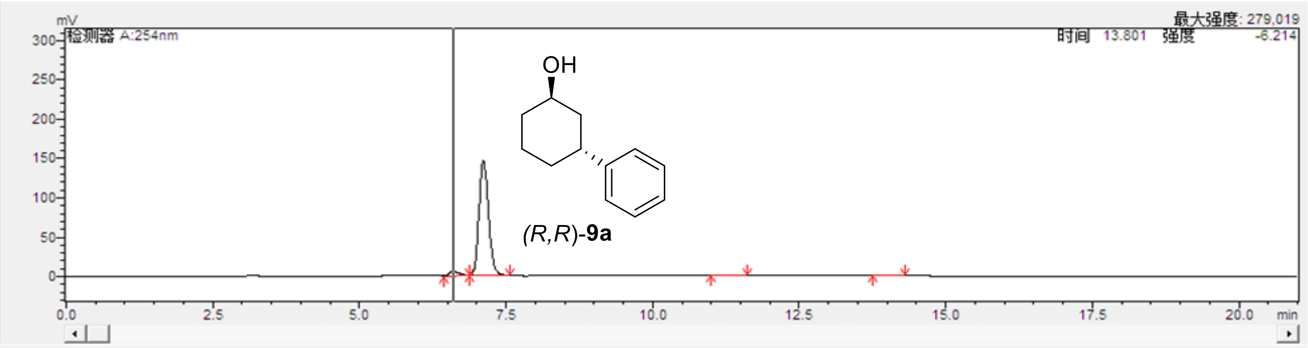

化合物表视图

ID#	名称	保留时间	峰#	面积	高度	面积%
1	RT6.592	6.592	1	68489	5997	3.8817
2	RT7.110	7.110	2	1683693	146623	95.4247
3	RT11.576	11.576	3	4692	181	0.2659
4	RT14.292	14.292	4	7547	346	0.4277

<u>**Translation of all characters (Chinese) in the above two frameworks to English is as follows:**</u>

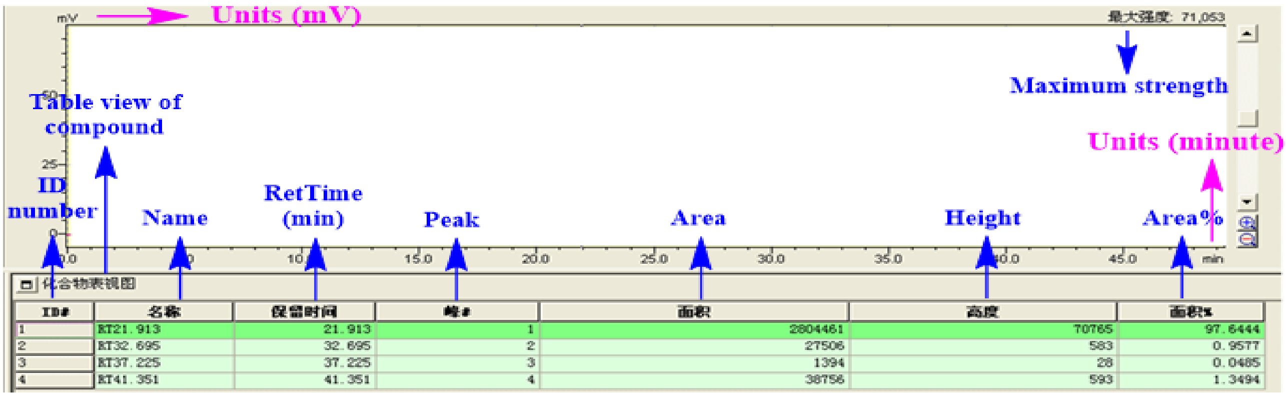

(*R*,*R*)-9b: (1*R*,3*R*)-3-(4-fluorophenyl)cyclohexan-1-ol (HPLC: Chiracel AD, detected at 254 nm, eluent: n-hexane/2-propanol = 98/2, flow rate = 1.0mL/min, 25°C).

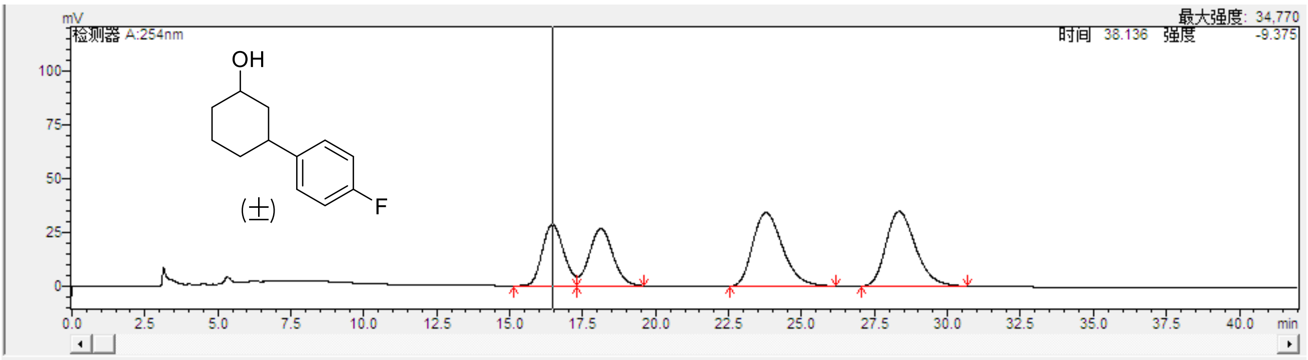

化合物表视图

ID#	名称	保留时间	峰#	面积	高度	面积%
1	RT16.461	16.461	1	1519116	28905	19.1617
2	RT18.120	18.120	2	1526086	26881	19.2497
3	RT23.776	23.776	3	2436542	34142	30.7339
4	RT28.338	28.338	4	2446110	34666	30.8546

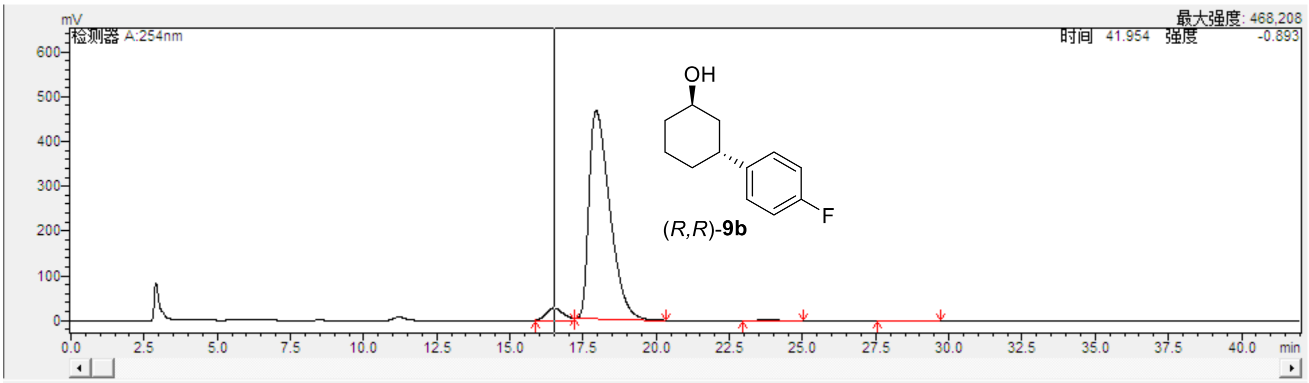

化合物表视图

ID#	名称	保留时间	峰#	面积	高度	面积%
1	RT16.497	16.497	1	1224985	28639	5.0472
2	RT17.947	17.947	2	22963233	464633	94.6133
3	RT23.797	23.797	3	61753	1133	0.2544
4	RT28.356	28.356	4	20636	336	0.0850

Translation of all characters (Chinese) in the above two frameworks to English is as follows:

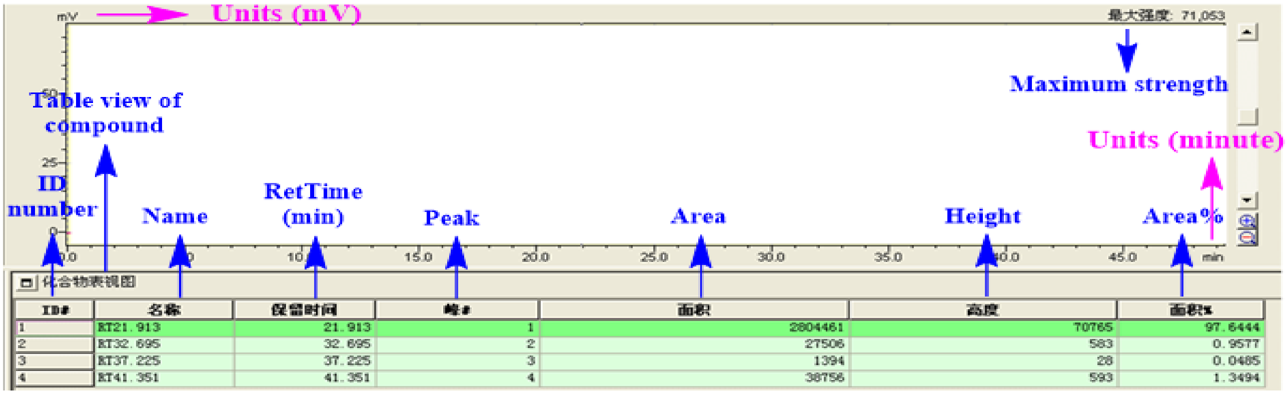

(*R*,*R*)-9c: (1*R*,3*R*)-3-(3-chlorophenyl)cyclohexan-1-ol (HPLC: Chiracel AD, detected at 220 nm, eluent: n-hexane/2-propanol = 99/1, flow rate = 1.0mL/min, 25°C).

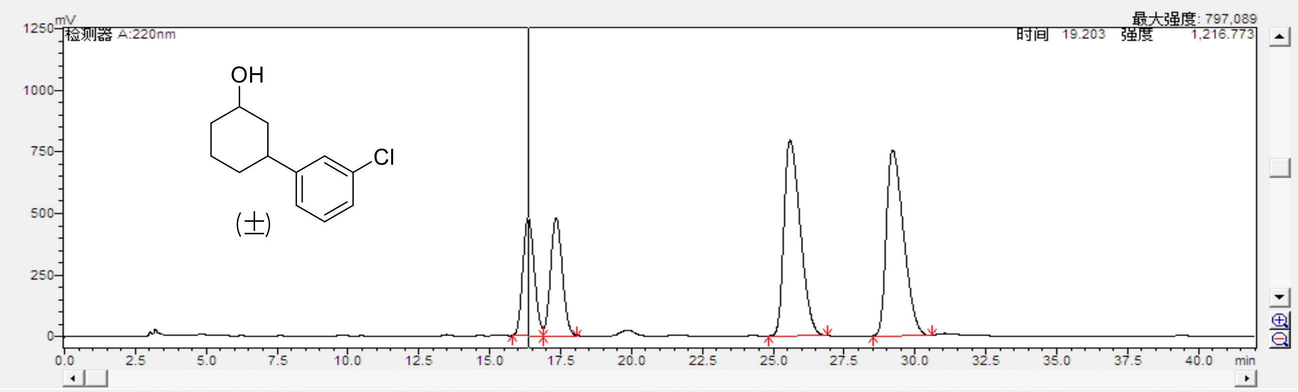

化合物表视图

ID#	名称	保留时间	峰#	面积	高度	面积%
1	RT16.352	16.352	1	13529110	478801	14.8104
2	RT17.335	17.335	2	13914077	479913	15.2318
3	RT25.594	25.594	3	31929437	792894	34.9533
4	RT29.213	29.213	4	31976191	754254	35.0045

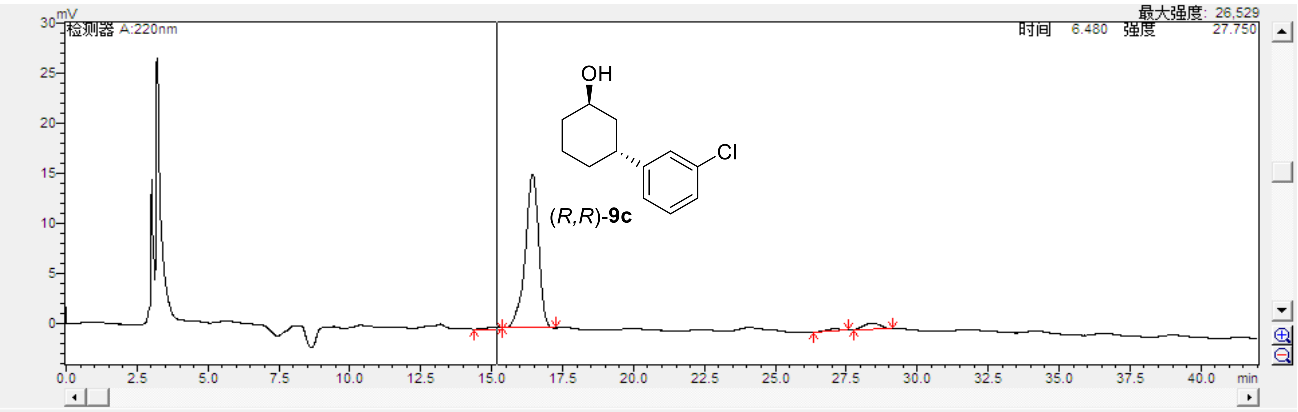

化合物表视图

ID#	名称	保留时间	峰#	面积	高度	面积%
1	RT15.176	15.176	1	8445	264	1.5553
2	RT16.439	16.439	2	500101	15332	92.1019
3	RT27.062	27.062	3	8324	219	1.5331
4	RT28.392	28.392	4	26116	636	4.8097

<u>**Translation of all characters (Chinese) in the above two frameworks to English is as follows:**</u>

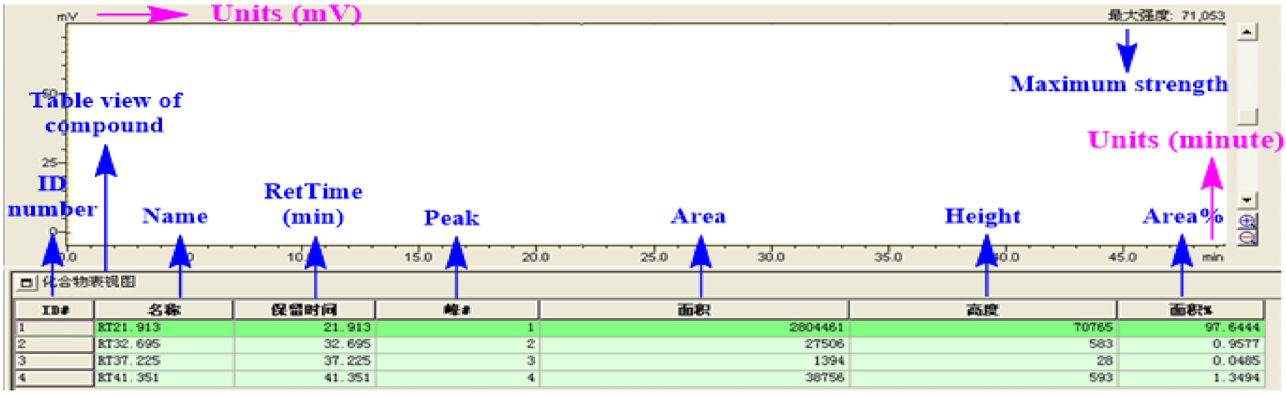

(*R*,*R*)-9d: (1*R*,3*R*)-3-(4-chlorophenyl)cyclohexan-1-ol: (HPLC: Chiracel OD, detected at 254 nm, eluent: n-hexane/2-propanol = 99/1, flow rate = 1.0mL/min, 27°C).

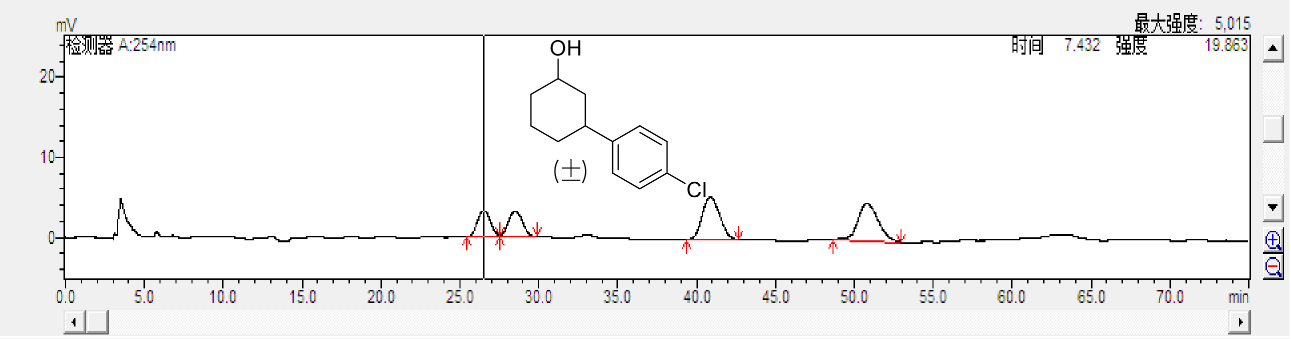

化合物表视图

ID#	名称	保留时间	峰#	面积	高度	面积%
1	RT26.488	26.488	1	191218	3194	15.5958
2	RT28.512	28.512	2	198242	3113	16.1687
3	RT40.848	40.848	3	408927	5270	33.3523
4	RT50.824	50.824	4	427695	4735	34.8831

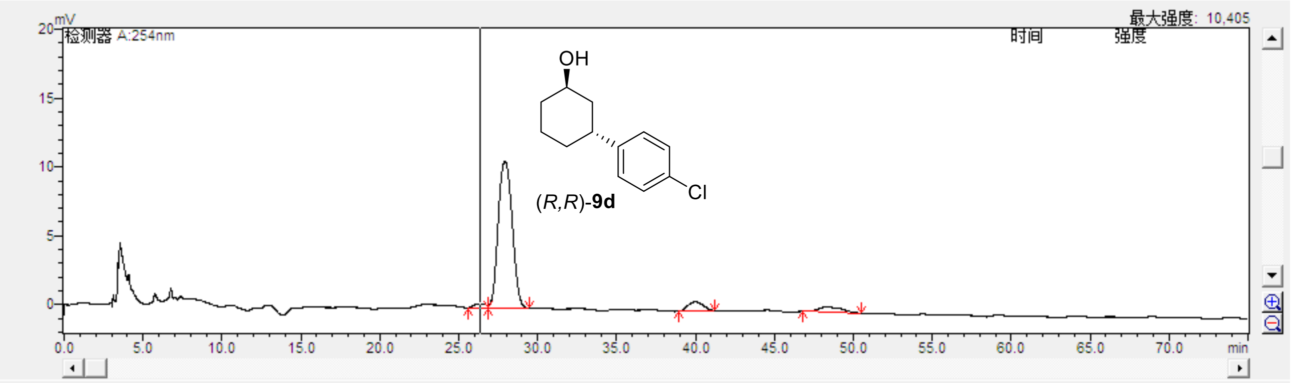

化合物表视图

ID#	名称	保留时间	峰#	面积	高度	面积%
1	RT26.353	26.353	1	15544	308	2.0427
2	RT27.917	27.917	2	660088	10648	86.7475
3	RT40.003	40.003	3	44723	662	5.8774
4	RT48.426	48.426	4	40575	378	5.3323

<u>Translation of all characters (Chinese) in the above two frameworks to English is as follows:</u>

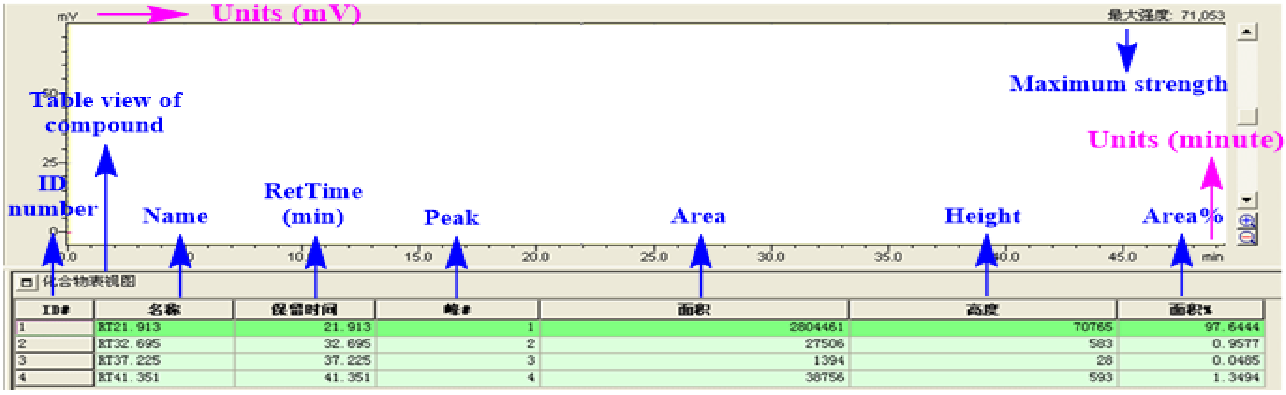

(*R*,*R*)-9e: (1*R*,3*R*)-3-(4-bromophenyl)cyclohexan-1-ol (HPLC: Chiracel OD, detected at 220 nm, eluent: n-hexane/2-propanol = 98/2, flow rate = 1.0mL/min, 27°C).

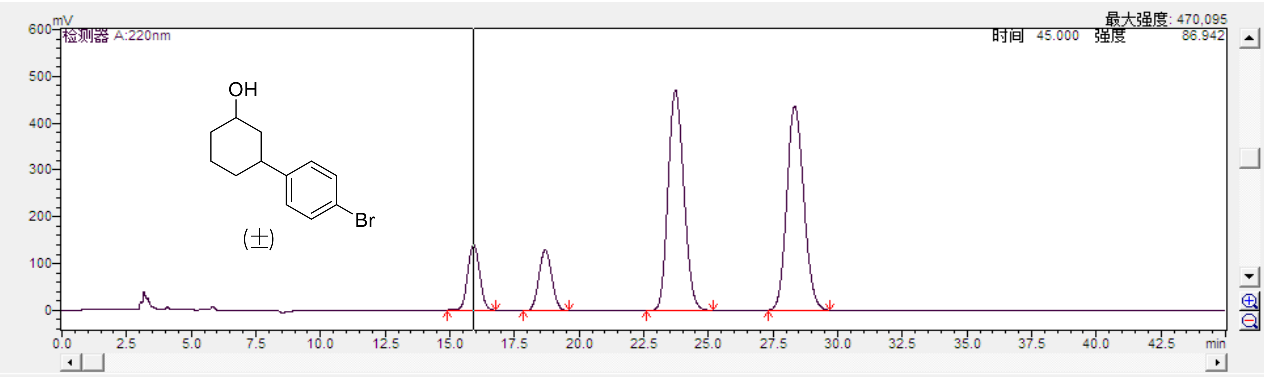

化合物表视图

ID#	名称	保留时间	峰#	面积	高度	面积%
1	RT15.917	15.917	1	4516230	138945	9.2056
2	RT18.687	18.687	2	4500487	129799	9.1735
3	RT23.719	23.719	3	19996642	470405	40.7599
4	RT28.335	28.335	4	20046214	435752	40.8610

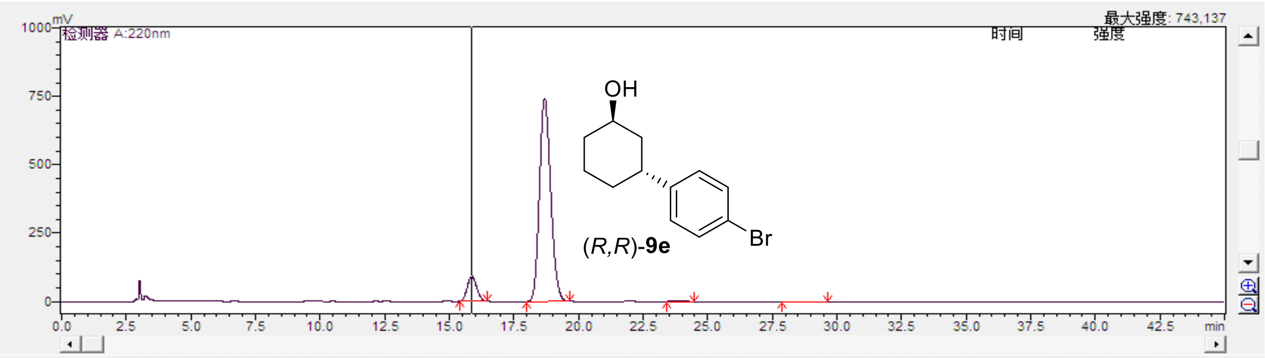

化合物表视图

ID#	名称	保留时间	峰#	面积	高度	面积%
1	RT15.866	15.866	1	2284655	90885	9.1507
2	RT18.686	18.686	2	22577626	743003	90.4295
3	RT23.823	23.823	3	81278	2567	0.3255
4	RT28.610	28.610	4	23542	444	0.0943

Translation of all characters (Chinese) in the above two frameworks to English is as follows:

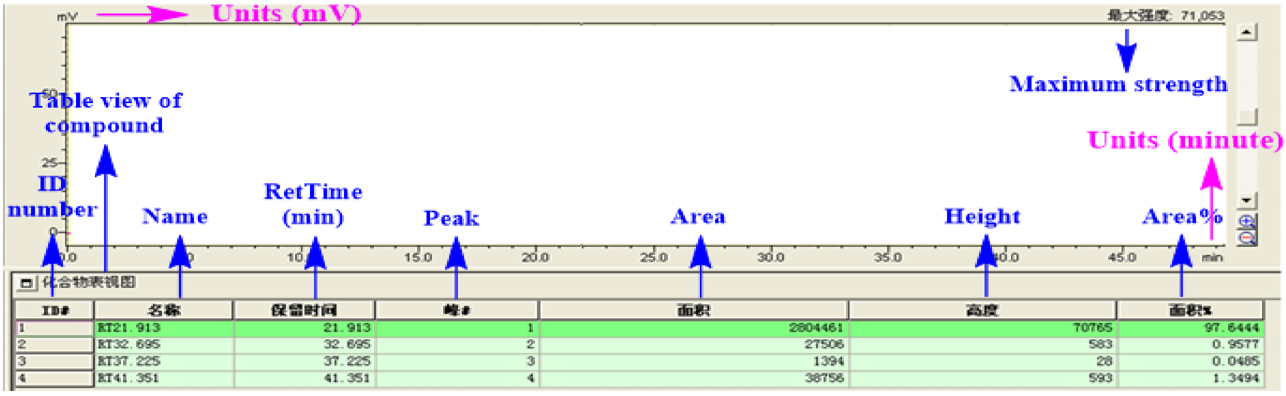

ID#	名称	保留时间	峰#	面积	高度	面积%
1	RT21.913	21.913	1	2804461	70765	97.6444
2	RT32.695	32.695	2	27506	583	0.9577
3	RT37.225	37.225	3	1394	28	0.0485
4	RT41.351	41.351	4	38756	593	1.3494

(*R*,*R*)-9f: (1*R*,3*R*)-3-(4-(trifluoromethyl)phenyl)cyclohexan-1-ol (HPLC: Chiracel AD, detected at 254 nm, eluent: n-hexane/2-propanol = 98/2, flow rate = 1.0mL/min, 25°C).

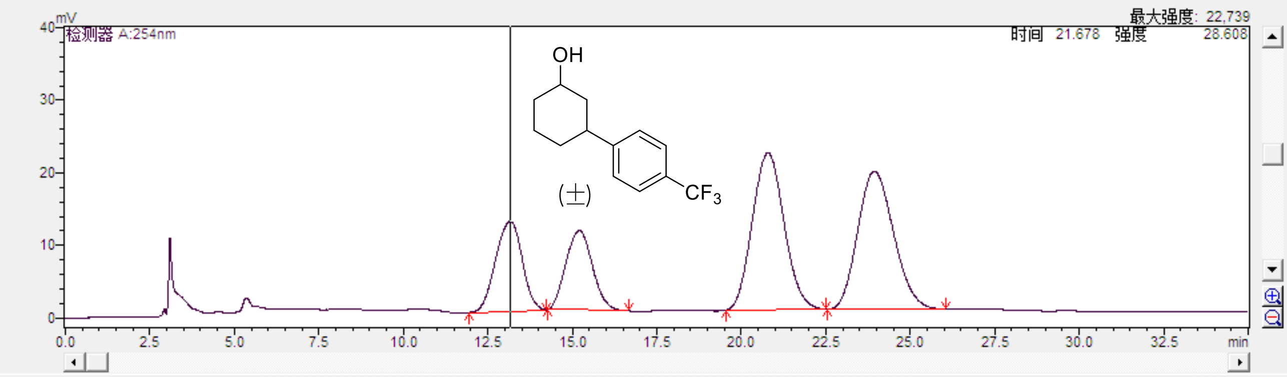

化合物表视图

ID#	名称	保留时间	峰#	面积	高度	面积%
1	RT13.160	13.160	1	674806	12333	16.4673
2	RT15.208	15.208	2	602641	10950	14.7062
3	RT20.789	20.789	3	1416876	21622	34.5760
4	RT23.935	23.935	4	1403533	19011	34.2504

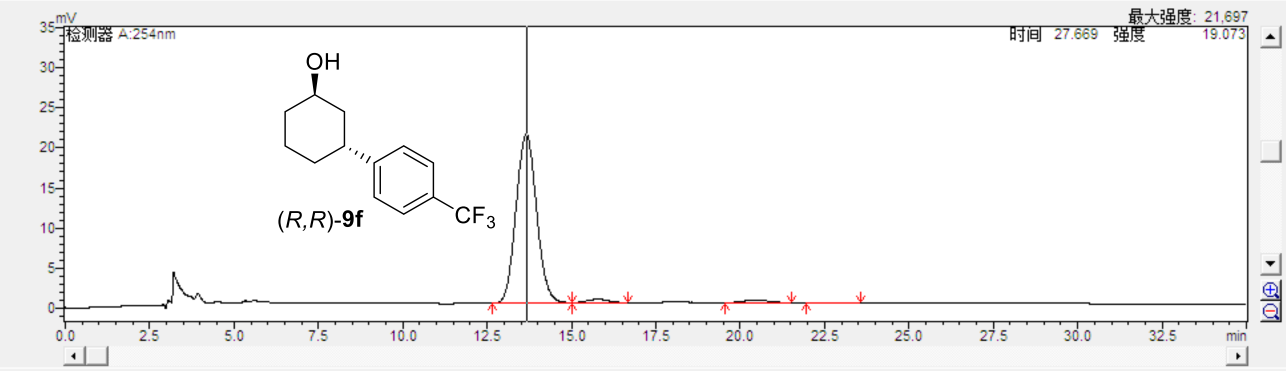

化合物表视图

ID#	名称	保留时间	峰#	面积	高度	面积%
1	RT13.659	13.659	1	891121	21034	95.8035
2	RT15.737	15.737	2	17670	413	1.8997
3	RT20.468	20.468	3	18029	356	1.9383
4	RT22.933	22.933	4	3335	66	0.3586

<u>**Translation of all characters (Chinese) in the above two frameworks to English is as follows:**</u>

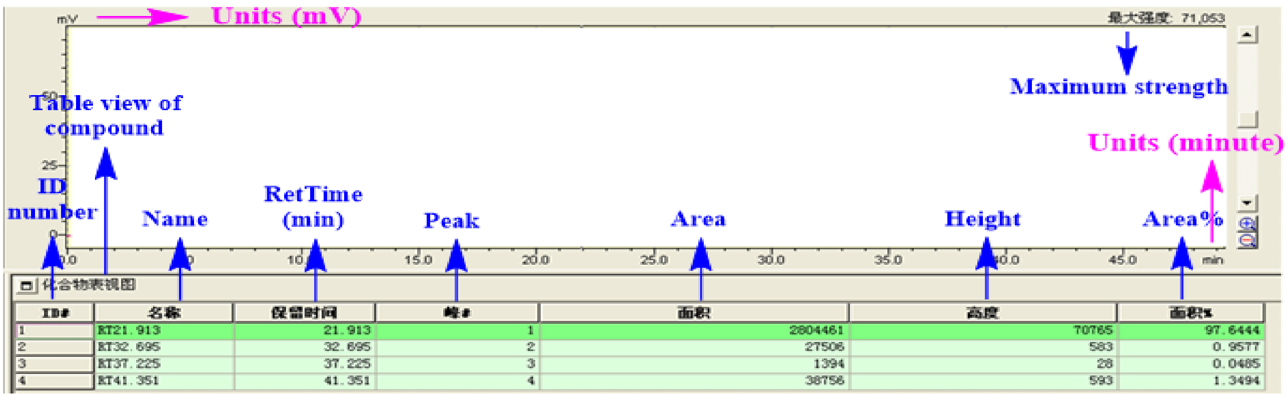

ID#	名称	保留时间	峰#	面积	高度	面积%
1	RT21.913	21.913	1	2804461	70765	97.6444
2	RT32.695	32.695	2	27506	583	0.9577
3	RT37.225	37.225	3	1394	28	0.0485
4	RT41.351	41.351	4	38756	593	1.3494

(*R*,*R*)-9g: methyl 4-((1*R*,3*R*)-3-hydroxycyclohexyl)benzoate: (HPLC: Chiracel OD, detected at 254 nm, eluent: n-hexane/2-propanol = 98/2, flow rate = 1.0mL/min, 27°C).

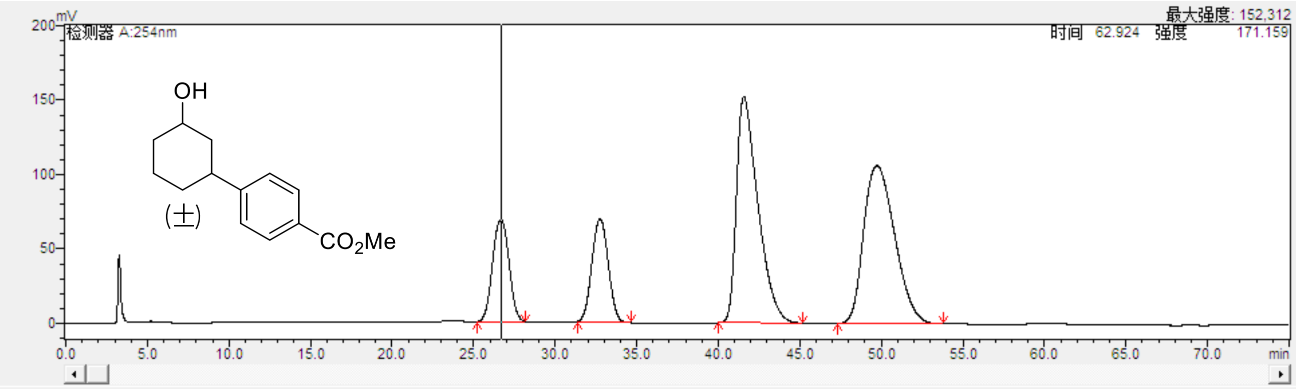

化合物表视图

ID#	名称	保留时间	峰#	面积	高度	面积%
1	RT26.672	26.672	1	4817225	68459	12.7790
2	RT32.749	32.749	2	4853640	69344	12.8756
3	RT41.569	41.569	3	13945064	152024	36.9931
4	RT49.724	49.724	4	14080439	106221	37.3522

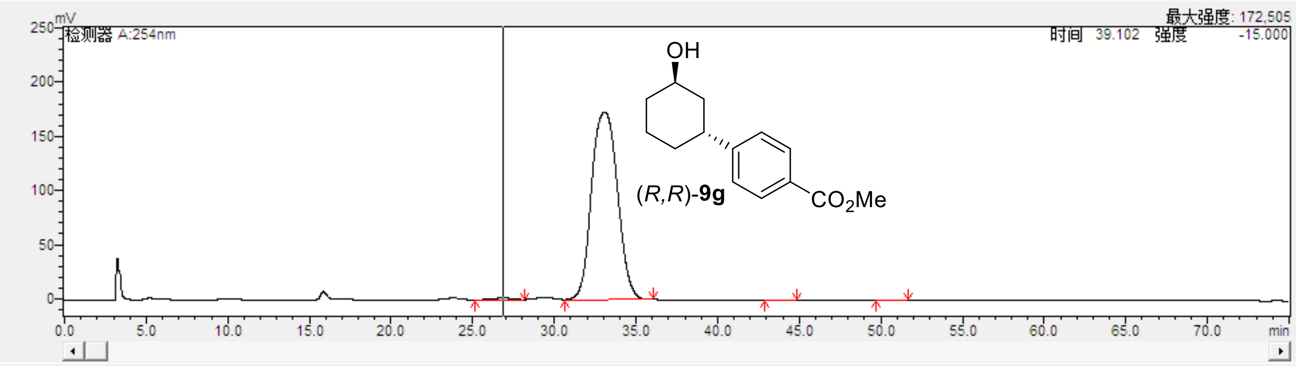

化合物表视图

ID#	名称	保留时间	峰#	面积	高度	面积%
1	RT26.852	26.852	1	120303	1570	0.6240
2	RT33.087	33.087	2	19136009	173259	99.2502
3	RT43.652	43.652	3	15501	230	0.0804
4	RT50.539	50.539	4	8757	155	0.0454

<u>**Translation of all characters (Chinese) in the above two frameworks to English is as follows:**</u>

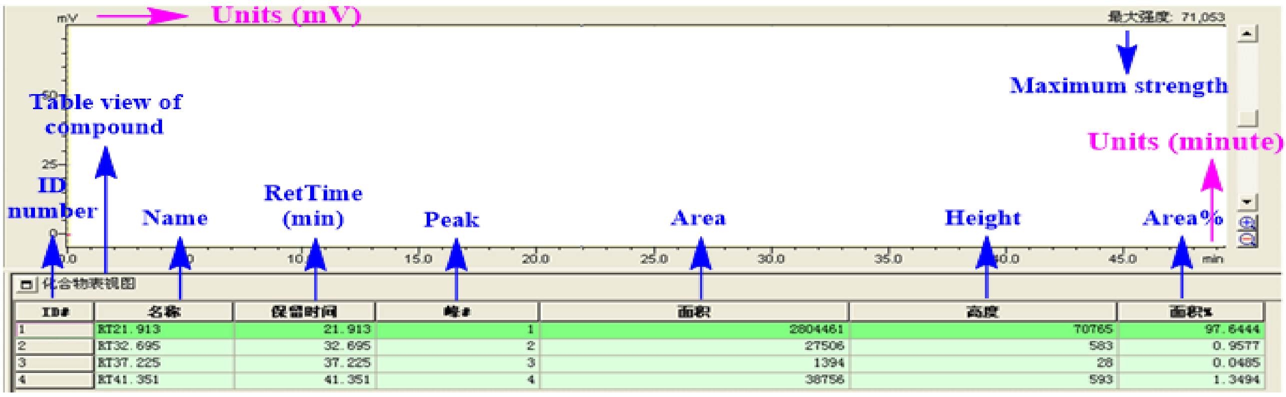

(*R*,*R*)-9h: (1*R*,3*R*)-3-(2-methoxyphenyl)cyclohexan-1-ol (HPLC: Chiracel OD, detected at 220 nm, eluent: n-hexane/2-propanol = 95/5, flow rate = 1.0mL/min, 25°C).

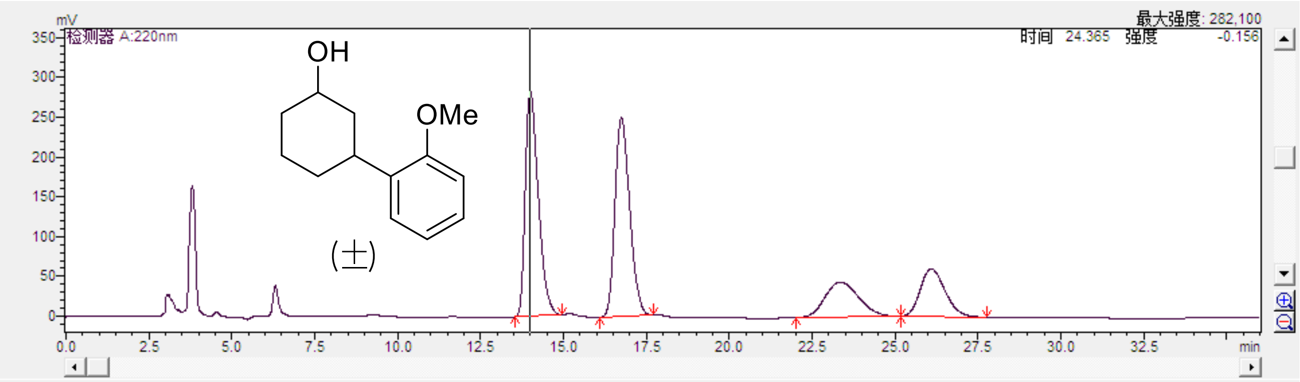

化合物表视图

ID#	名称	保留时间	峰#	面积	高度	面积%
1	RT13.999	13.999	1	7734333	282265	35.6689
2	RT16.744	16.744	2	7718115	250252	35.5941
3	RT23.347	23.347	3	3100887	44092	14.3006
4	RT26.088	26.088	4	3130342	60429	14.4364

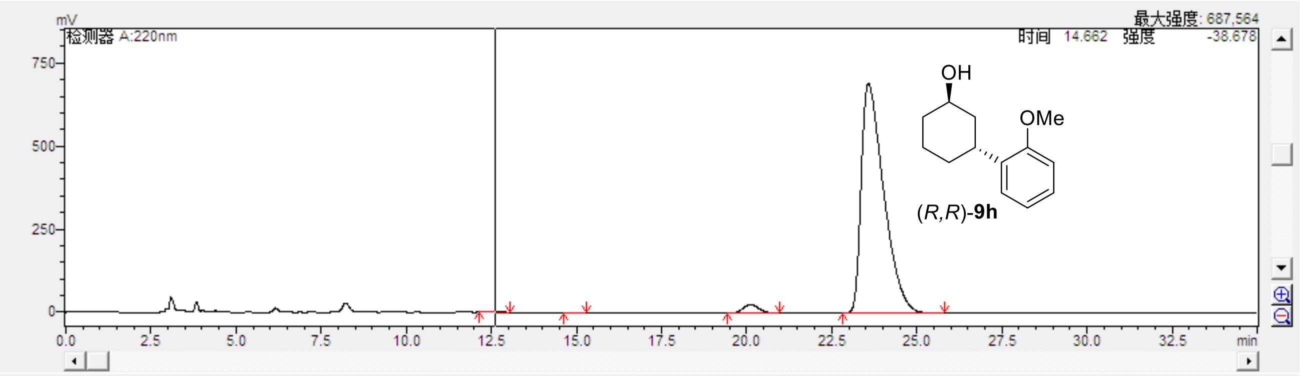

化合物表视图

ID#	名称	保留时间	峰#	面积	高度	面积%
1	RT12.605	12.605	1	28800	1282	0.0880
2	RT14.914	14.914	2	8939	411	0.0273
3	RT20.107	20.107	3	842584	25550	2.5741
4	RT23.578	23.578	4	31852206	690843	97.3106

Translation of all characters (Chinese) in the above two frameworks to English is as follows:

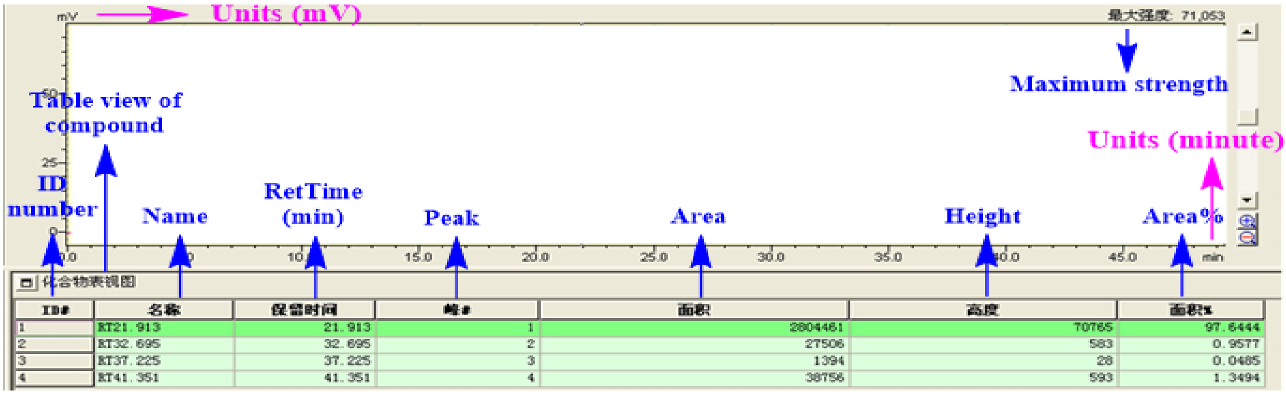

(*R*,*R*)-9i: (1*R*,3*R*)-3-(4-methoxyphenyl)cyclohexan-1-ol (HPLC: Chiracel AD, detected at 254 nm, eluent: n-hexane/2-propanol = 98/2, flow rate = 1.0mL/min, 25°C).

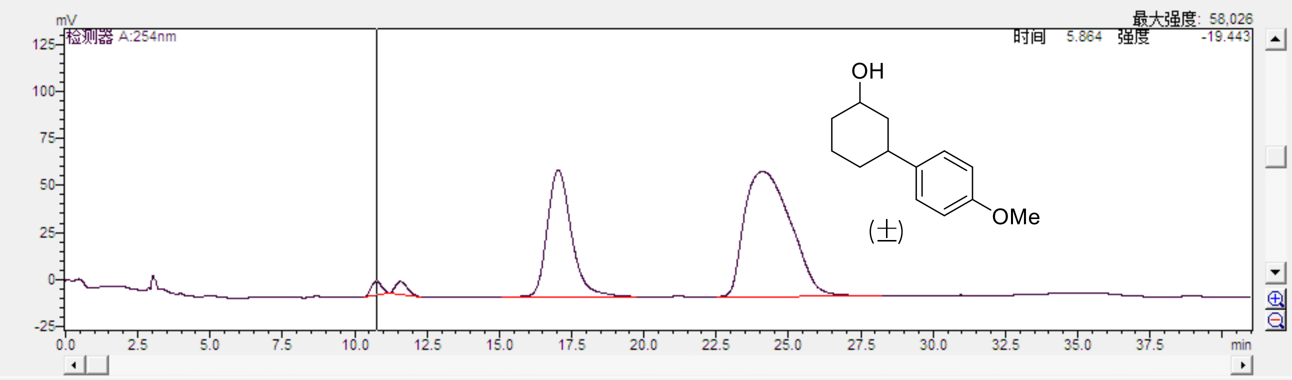

化合物表视图

ID#	名称	保留时间	峰#	面积	高度	面积%
1	RT10.736	10.736	1	181788	7282	1.5473
2	RT11.574	11.574	2	195865	6825	1.6671
3	RT17.033	17.033	3	3934405	67472	33.4875
4	RT24.096	24.096	4	7436806	66702	63.2981

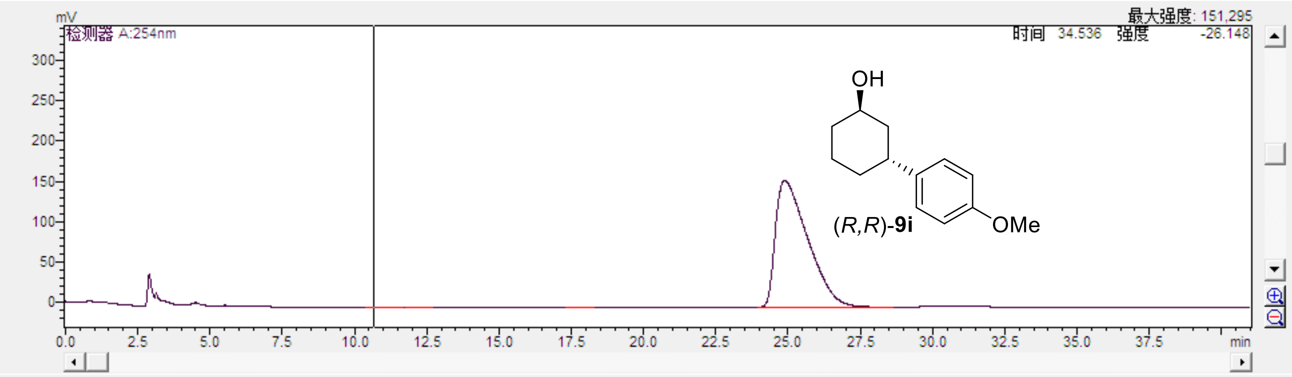

化合物表视图

ID#	名称	保留时间	峰#	面积	高度	面积%
1	RT10.647	10.647	1	7050	137	0.0573
2	RT12.360	12.360	2	11224	519	0.0912
3	RT17.721	17.721	3	2241	65	0.0182
4	RT24.884	24.884	4	12282874	157610	99.8333

<u>**Translation of all characters (Chinese) in the above two frameworks to English is as follows:**</u>

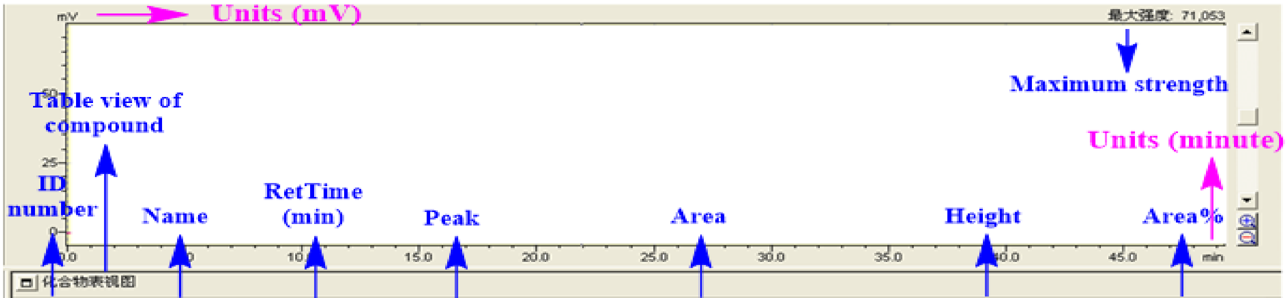

ID#	名称	保留时间	峰#	面积	高度	面积%
1	RT21.913	21.913	1	2804461	70765	97.6444
2	RT32.695	32.695	2	27506	583	0.9577
3	RT37.225	37.225	3	1394	28	0.0485
4	RT41.351	41.351	4	38756	593	1.3494

(*R*,*R*)-9j: (1*R*,3*R*)-3-(4-(methylthio)phenyl)cyclohexan-1-ol (HPLC: Chiracel AD, detected at 254 nm, eluent: n-hexane/2-propanol = 98/2, flow rate = 1.0mL/min, 27°C).

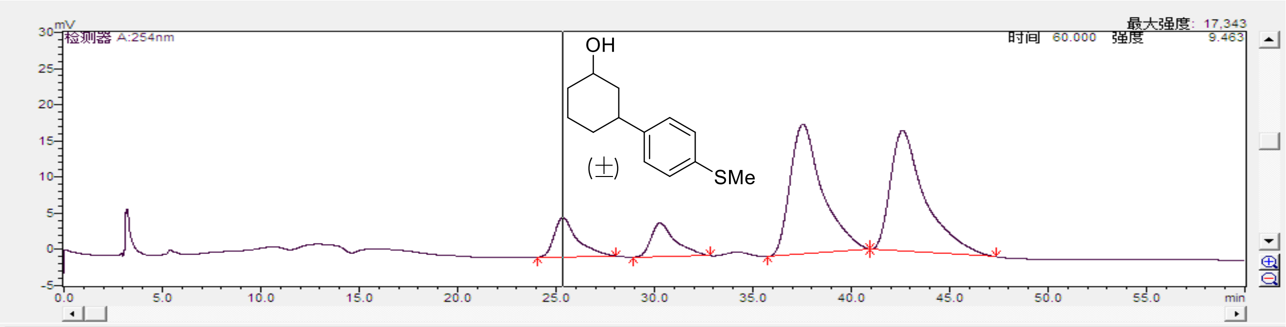

ID#	名称	保留时间	峰#	面积	高度	面积%
1	RT25.361	25.361	1	438830	5416	8.9099
2	RT30.290	30.290	2	398342	4648	8.0879
3	RT37.528	37.528	3	2047371	17975	41.5694
4	RT42.587	42.587	4	2040648	16659	41.4329

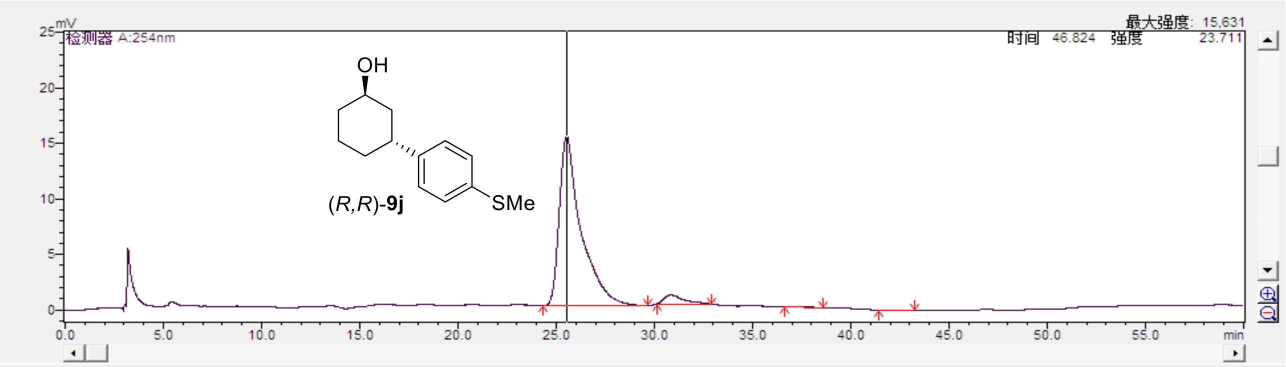

ID#	名称	保留时间	峰#	面积	高度	面积%
1	RT25.528	25.528	1	1164046	15221	94.7133
2	RT30.820	30.820	2	59390	821	4.8323
3	RT37.325	37.325	3	3948	74	0.3213
4	RT41.929	41.929	4	1636	33	0.1332

<u>Translation of all characters (Chinese) in the above two frameworks to English is as follows:</u>

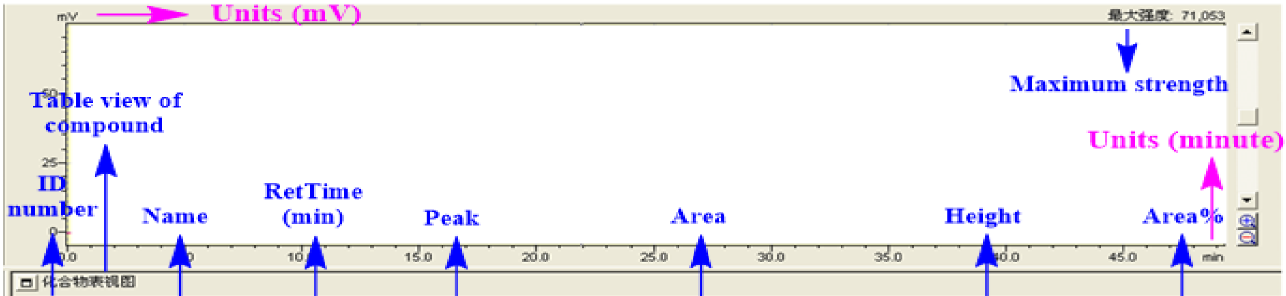

ID#	名称	保留时间	峰#	面积	高度	面积%
1	RT21.913	21.913	1	2804461	70765	97.6444
2	RT32.695	32.695	2	27506	583	0.9577
3	RT37.225	37.225	3	1394	28	0.0485
4	RT41.351	41.351	4	38756	593	1.3494

(*R*,*R*)-9k: (1*R*,3*R*)-3-(o-tolyl)cyclohexan-1-ol (HPLC: Chiracel AD, detected at 220 nm, eluent: n-hexane/2-propanol = 98/2, flow rate = 1.0mL/min, 25°C).

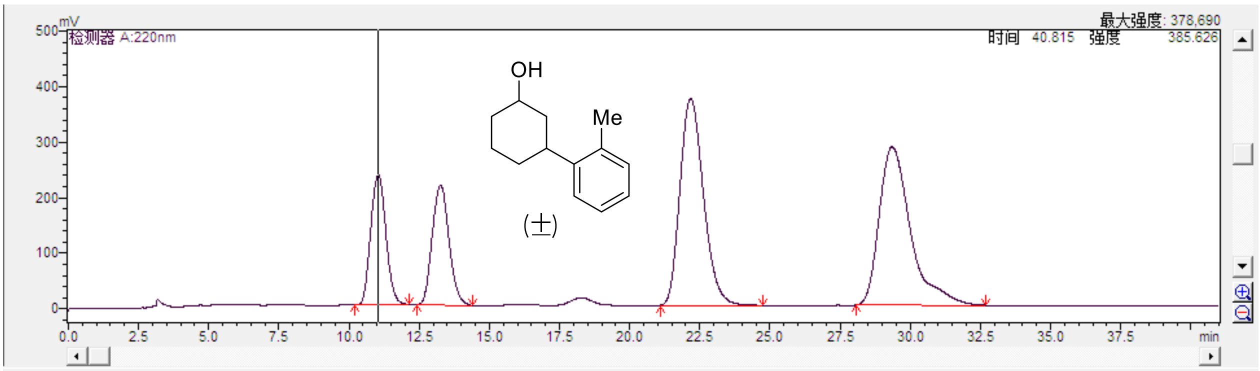

化合物表视图

ID#	名称	保留时间	峰#	面积	高度	面积%
1	RT11.040	11.040	1	8803514	233947	14.2510
2	RT13.258	13.258	2	9029928	215768	14.6175
3	RT22.170	22.170	3	21750824	372651	35.2099
4	RT29.351	29.351	4	22190449	284974	35.9216

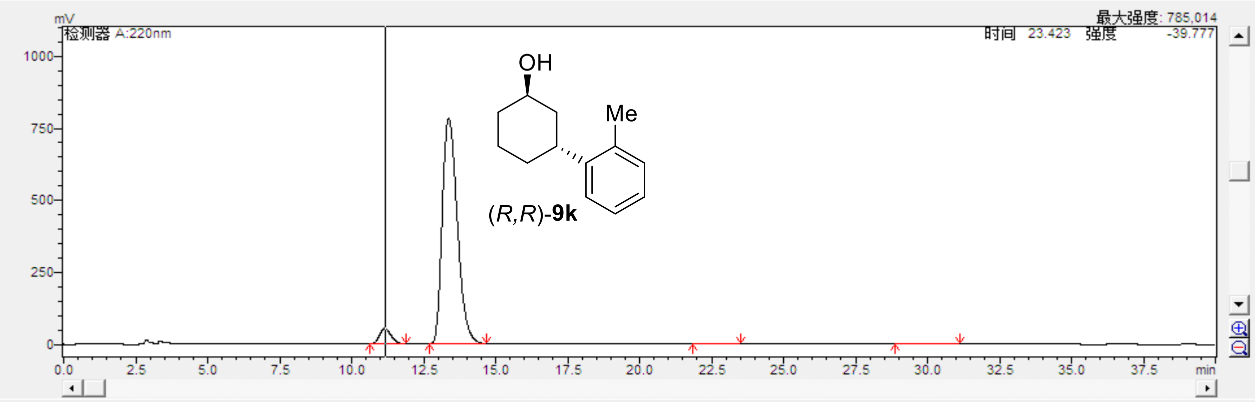

化合物表视图

ID#	名称	保留时间	峰#	面积	高度	面积%
1	RT11.150	11.150	1	1485998	53996	4.9352
2	RT13.370	13.370	2	28570877	783904	94.8886
3	RT22.384	22.384	3	25217	501	0.0837
4	RT29.624	29.624	4	27838	392	0.0925

<u>Translation of all characters (Chinese) in the above two frameworks to English is as follows:</u>

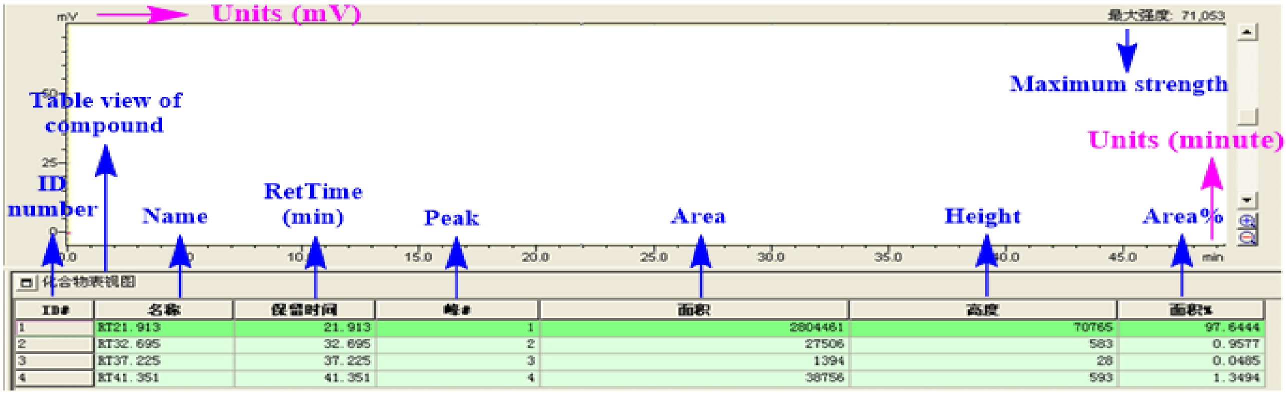

(*R*,*R*)-9l: (1*R*,3*R*)-3-(m-tolyl)cyclohexan-1-ol (HPLC: Chiracel IC, detected at 220 nm, eluent: n-hexane/2-propanol = 98/2, flow rate = 1.0mL/min, 25°C).

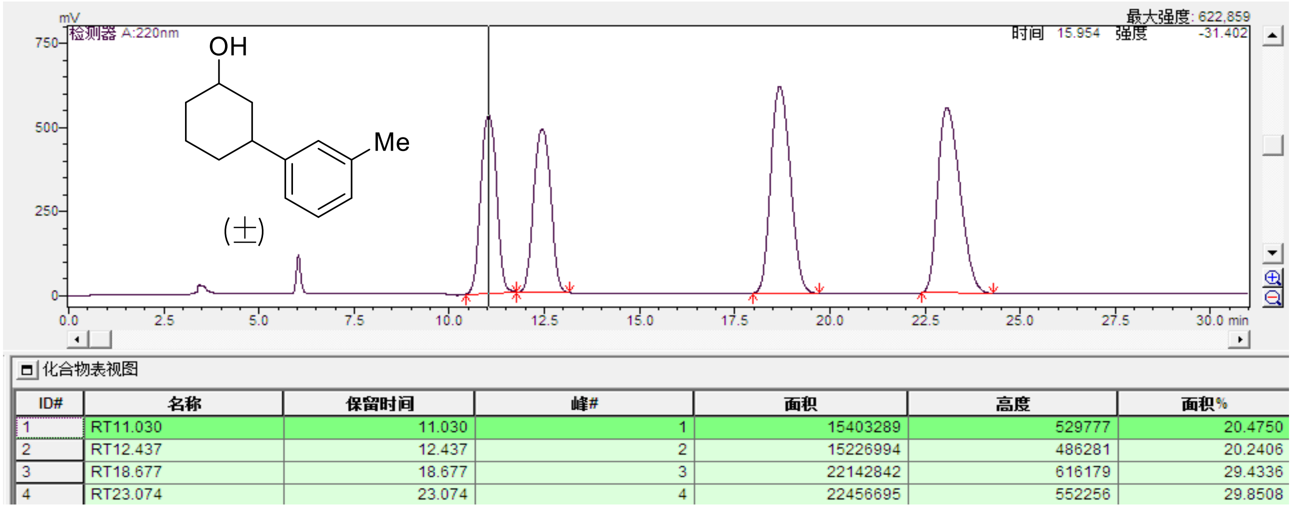

ID#	名称	保留时间	峰#	面积	高度	面积%
1	RT11.030	11.030	1	15403289	529777	20.4750
2	RT12.437	12.437	2	15226994	486281	20.2406
3	RT18.677	18.677	3	22142842	616179	29.4336
4	RT23.074	23.074	4	22456695	552256	29.8508

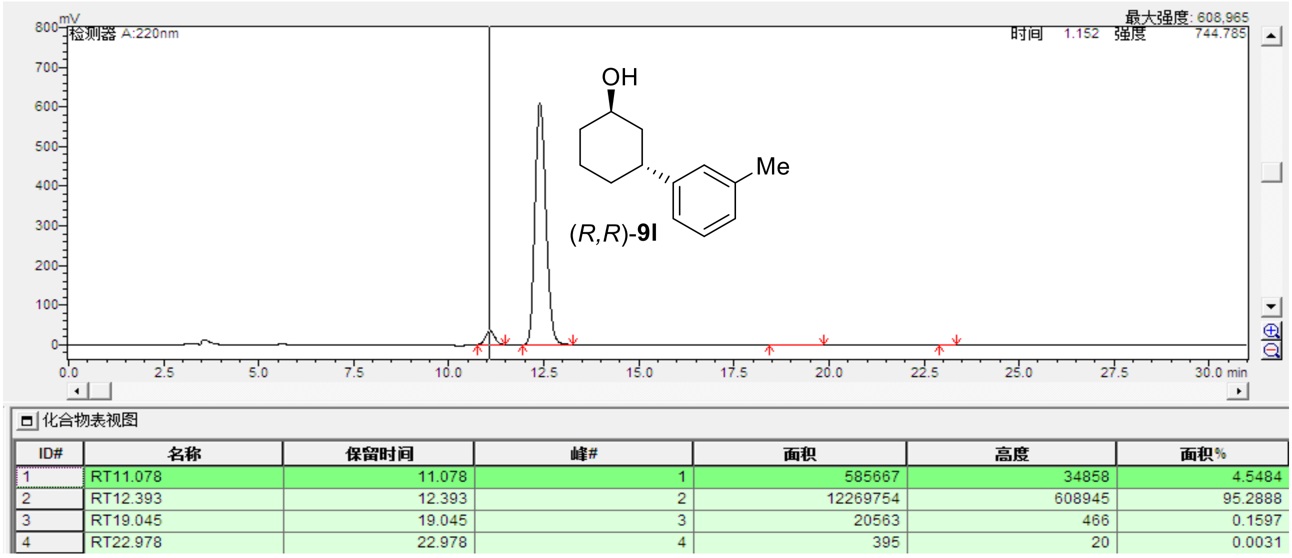

ID#	名称	保留时间	峰#	面积	高度	面积%
1	RT11.078	11.078	1	585667	34858	4.5484
2	RT12.393	12.393	2	12269754	608945	95.2888
3	RT19.045	19.045	3	20563	466	0.1597
4	RT22.978	22.978	4	395	20	0.0031

Translation of all characters (Chinese) in the above two frameworks to English is as follows:

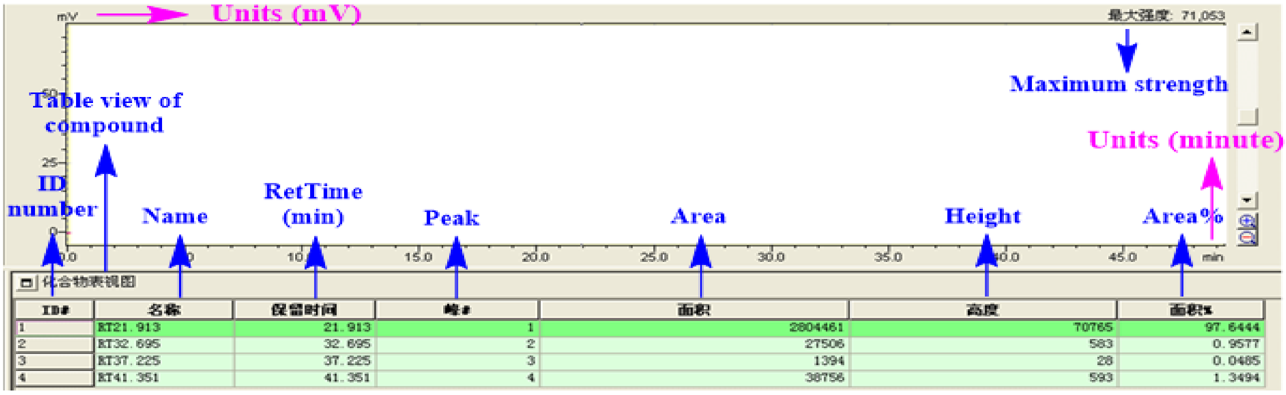

ID#	名称	保留时间	峰#	面积	高度	面积%
1	RT21.913	21.913	1	2804461	70765	97.6444
2	RT32.695	32.695	2	27506	583	0.9577
3	RT37.225	37.225	3	1394	28	0.0485
4	RT41.351	41.351	4	38756	593	1.3494

(*R*,*R*)-9m: (1*R*,3*R*)-3-(p-tolyl)cyclohexan-1-ol: (HPLC: Chiracel AD, detected at 254 nm, eluent: n-hexane/2-propanol = 98/2, flow rate = 1.0mL/min, 25°C).

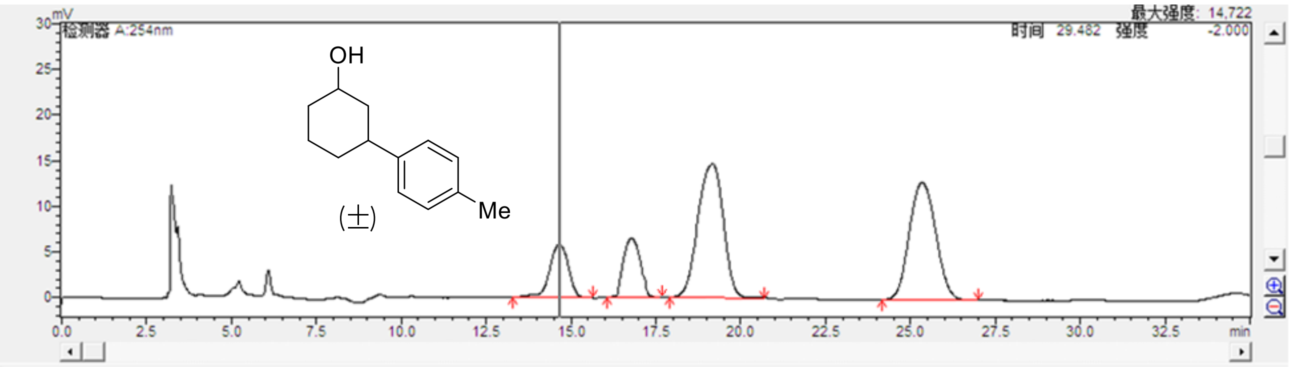

化合物表视图

ID#	名称	保留时间	峰#	面积	高度	面积%
1	RT14.661	14.661	1	225160	5812	12.2207
2	RT17.041	17.041	2	224695	5374	12.1955
3	RT19.154	19.154	3	696858	14753	37.8226
4	RT25.338	25.338	4	695724	12870	37.7611

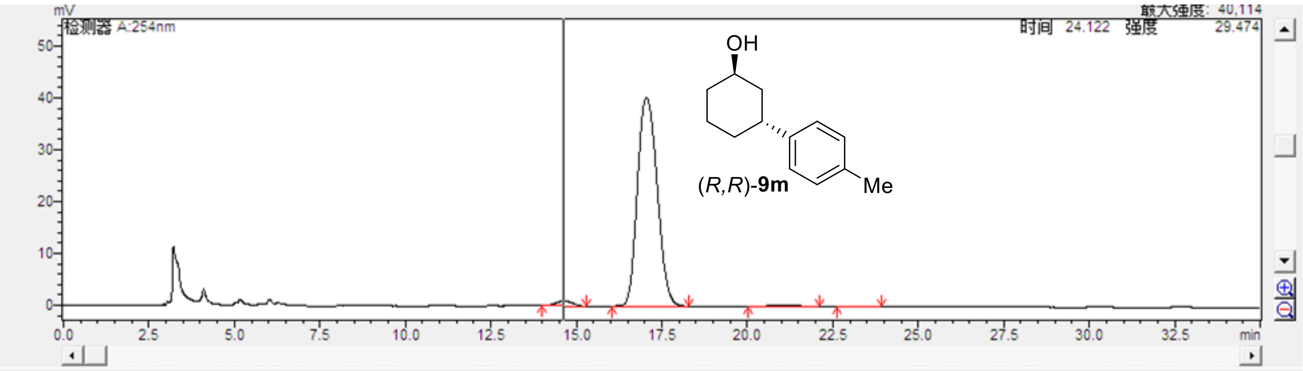

化合物表视图

ID#	名称	保留时间	峰#	面积	高度	面积%
1	RT14.641	14.641	1	33672	960	2.0300
2	RT17.049	17.049	2	1610274	40271	97.0799
3	RT20.936	20.936	3	12162	180	0.7332
4	RT23.077	23.077	4	2602	67	0.1569

<u>Translation of all characters (Chinese) in the above two frameworks to English is as follows:</u>

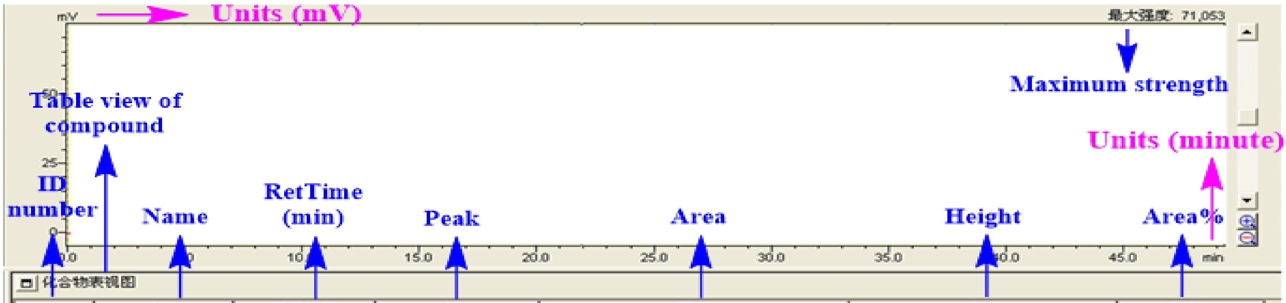

ID#	名称	保留时间	峰#	面积	高度	面积%
1	RT21.913	21.913	1	2804461	70765	97.6444
2	RT32.695	32.695	2	27506	583	0.9577
3	RT37.225	37.225	3	1394	28	0.0485
4	RT41.351	41.351	4	38756	593	1.3494

(*R*,*R*)-9n: (1*R*,3*R*)-3-(4-butylphenyl)cyclohexan-1-ol (HPLC: Chiracel IC, detected at 220 nm, eluent: n-hexane/2-propanol = 99/1, flow rate = 1.0mL/min, 25°C).

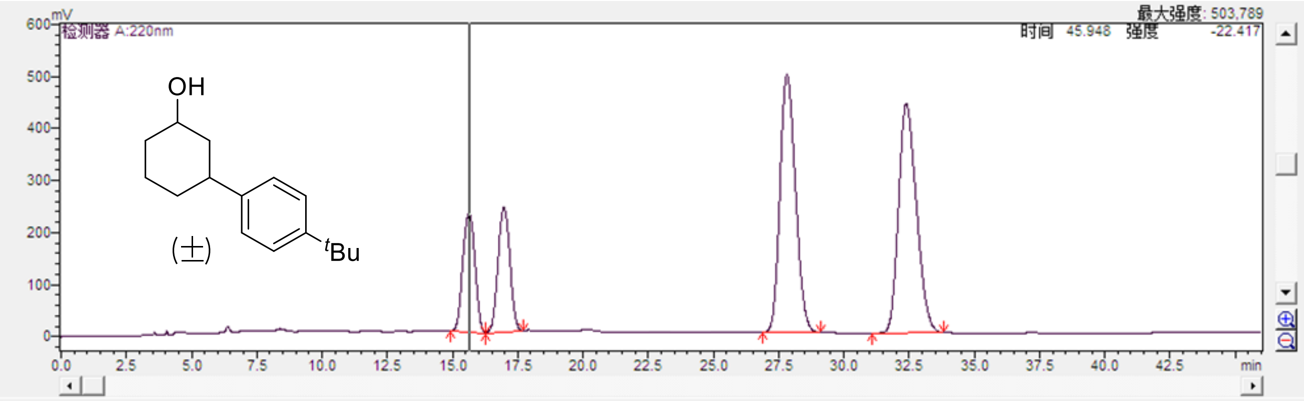

化合物表视图

ID#	名称	保留时间	峰#	面积	高度	面积%
1	RT15.627	15.627	1	7472712	227778	13.1608
2	RT16.969	16.969	2	7653497	239032	13.4792
3	RT27.812	27.812	3	20583125	495502	36.2505
4	RT32.385	32.385	4	21070889	440180	37.1096

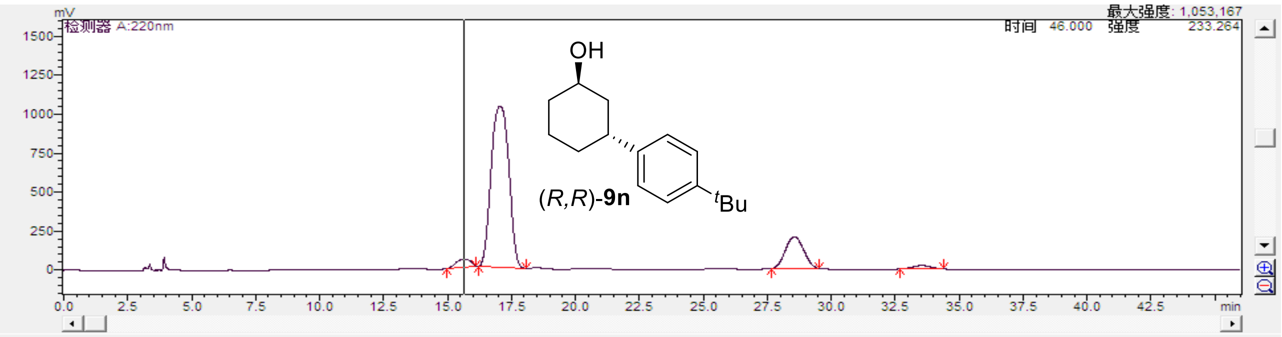

化合物表视图

ID#	名称	保留时间	峰#	面积	高度	面积%
1	RT15.656	15.656	1	2038605	49221	3.2074
2	RT17.054	17.054	2	50174149	1037427	78.9398
3	RT28.551	28.551	3	10217007	204707	16.0746
4	RT33.543	33.543	4	1130257	22069	1.7783

<u>**Translation of all characters (Chinese) in the above two frameworks to English is as follows:**</u>

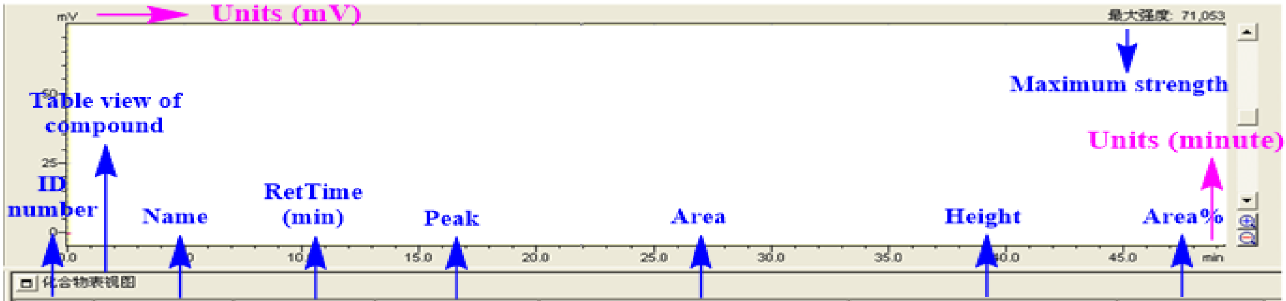

ID#	名称	保留时间	峰#	面积	高度	面积%
1	RT21.913	21.913	1	2804461	70765	97.6444
2	RT32.695	32.695	2	27506	583	0.9577
3	RT37.225	37.225	3	1394	28	0.0485
4	RT41.351	41.351	4	38756	593	1.3494

(*R*,*R*)-9o: (1*R*,3*R*)-3-(thiophen-3-yl)cyclohexan-1-ol: (HPLC: Chiracel AD, detected at 220 nm, eluent: n-hexane/2-propanol = 98/2, flow rate = 1.0mL/min, 27°C).

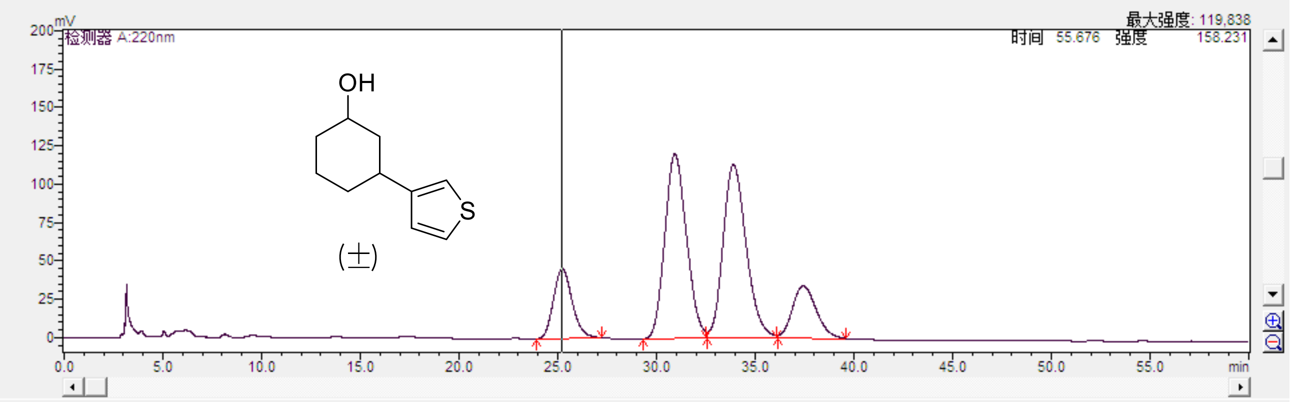

化合物表视图

ID#	名称	保留时间	峰#	面积	高度	面积%
1	RT25.224	25.224	1	2993918	45369	12.3017
2	RT30.927	30.927	2	9219815	120510	37.8834
3	RT33.894	33.894	3	9201649	113221	37.8087
4	RT37.419	37.419	4	2921980	34356	12.0061

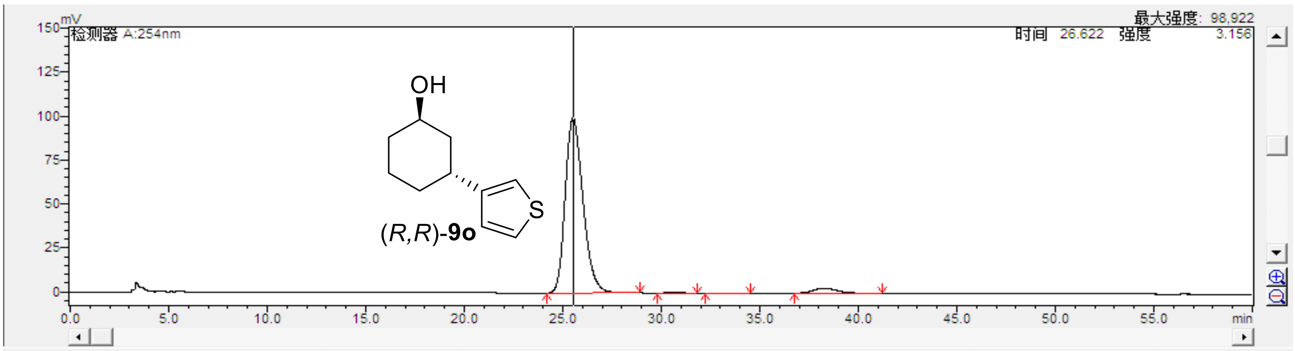

化合物表视图

ID#	名称	保留时间	峰#	面积	高度	面积%
1	RT25.514	25.514	1	6257717	99664	95.8273
2	RT30.717	30.717	2	9358	173	0.1433
3	RT32.658	32.658	3	5252	83	0.0804
4	RT38.242	38.242	4	257878	2902	3.9490

<u>**Translation of all characters (Chinese) in the above two frameworks to English is as follows:**</u>

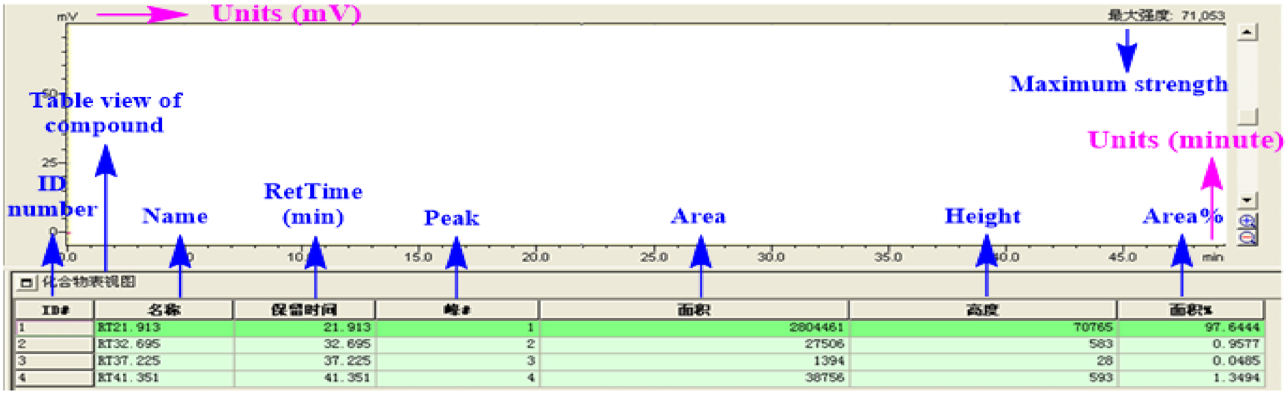

(*R*,*R*)-9p: *tert*-butyl 3-((1*R*,3*R*)-3-hydroxycyclohexyl)-1H-indole-1-carboxylate: (HPLC: Chiracel AS, detected at 254 nm, eluent: n-hexane/2-propanol = 98/2, flow rate = 1.0mL/min, 27°C).

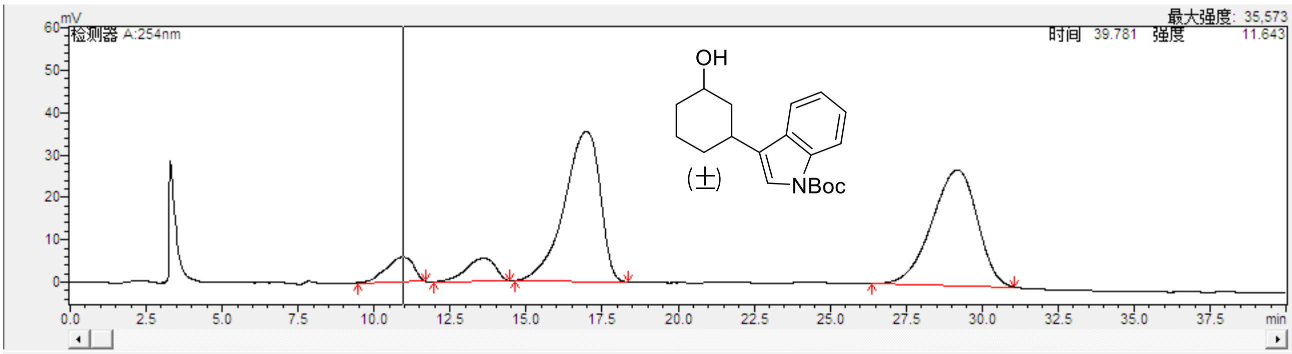

化合物表视图

ID#	名称	保留时间	峰#	面积	高度	面积%
1	RT10.943	10.943	1	363785	5779	5.6422
2	RT13.601	13.601	2	355921	5366	5.5202
3	RT16.983	16.983	3	2897832	35411	44.9442
4	RT29.189	29.189	4	2830087	27323	43.8935

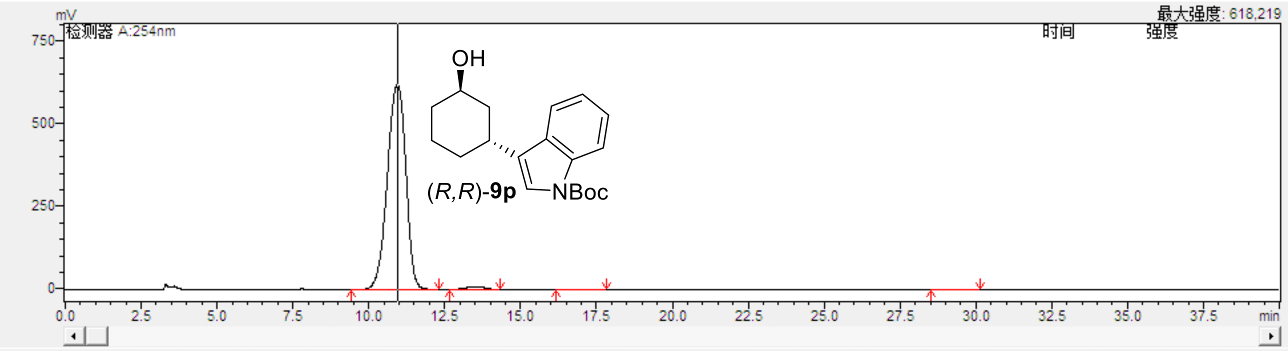

化合物表视图

ID#	名称	保留时间	峰#	面积	高度	面积%
1	RT10.937	10.937	1	26387914	620318	98.6172
2	RT13.527	13.527	2	364756	8346	1.3632
3	RT17.010	17.010	3	2028	61	0.0076
4	RT29.055	29.055	4	3229	72	0.0121

<u>**Translation of all characters (Chinese) in the above two frameworks to English is as follows:**</u>

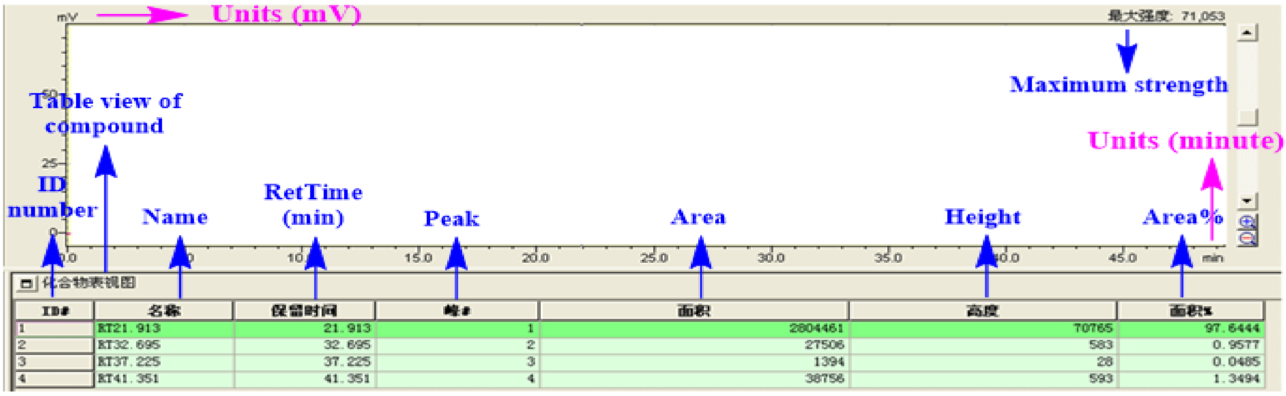

(*R*,*R*)-9q: (1*R*,3*R*)-3-([1,1'-biphenyl]-4-yl)cyclohexan-1-ol: (HPLC: Chiracel IC, detected at 254 nm, eluent: n-hexane/2-propanol = 98/2, flow rate = 1.0mL/min, 27°C).

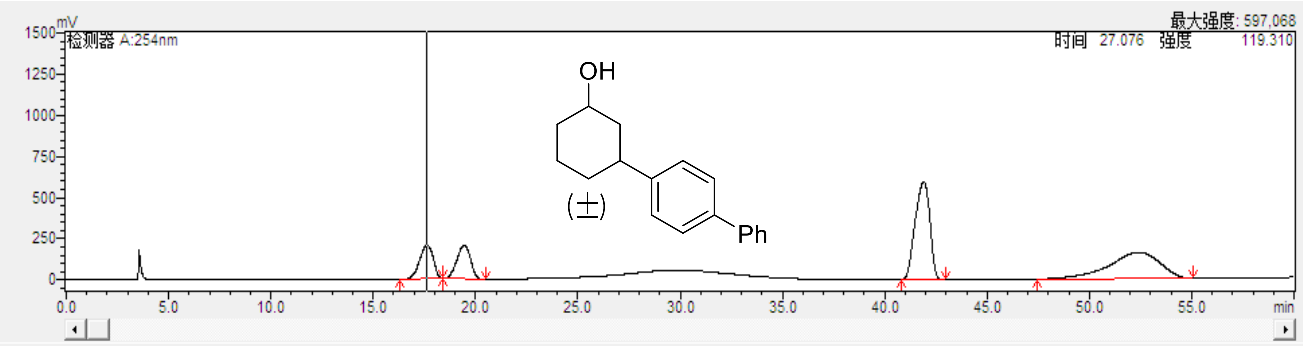

化合物表视图

ID#	名称	保留时间	峰#	面积	高度	面积%
1	RT17.630	17.630	1	9514480	203913	12.3882
2	RT19.443	19.443	2	9504021	203550	12.3746
3	RT41.900	41.900	3	29673484	594432	38.6359
4	RT52.423	52.423	4	28110884	161078	36.6013

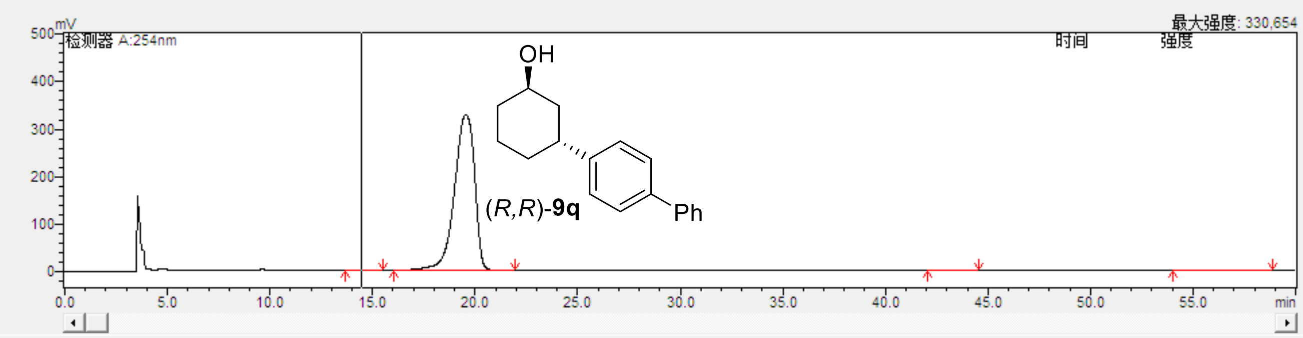

化合物表视图

ID#	名称	保留时间	峰#	面积	高度	面积%
1	RT14.427	14.427	1	3666	79	0.0163
2	RT19.546	19.546	2	22285581	326410	99.0602
3	RT43.068	43.068	3	18505	255	0.0823
4	RT56.166	56.166	4	189263	1519	0.8413

__Translation of all characters (Chinese) in the above two frameworks to English is as follows:__

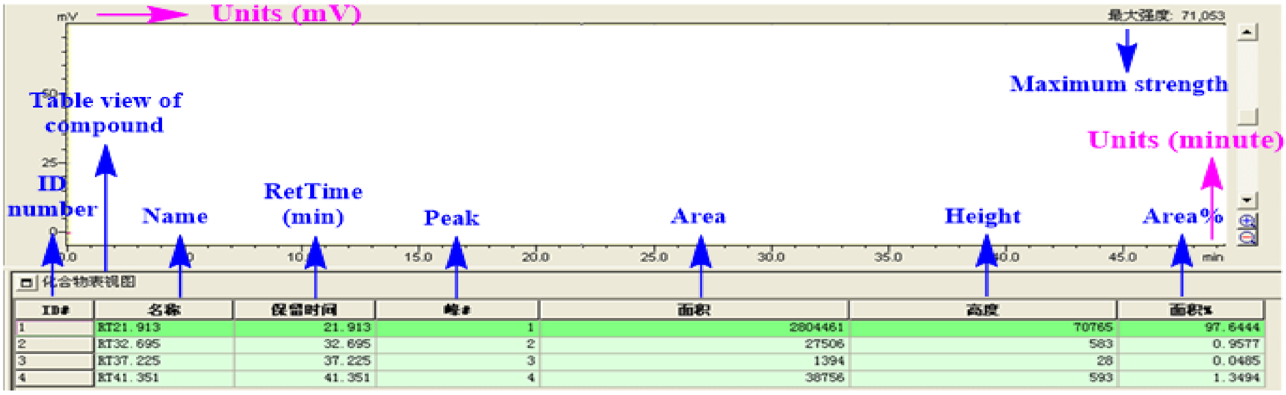

(*R*,*R*)-9r: (1*R*,3*R*)-3-(naphthalen-1-yl)cyclohexan-1-ol: (HPLC: Chiracel AD, detected at 220 nm, eluent: n-hexane/2-propanol = 98/2, flow rate = 1.0mL/min, 27°C).

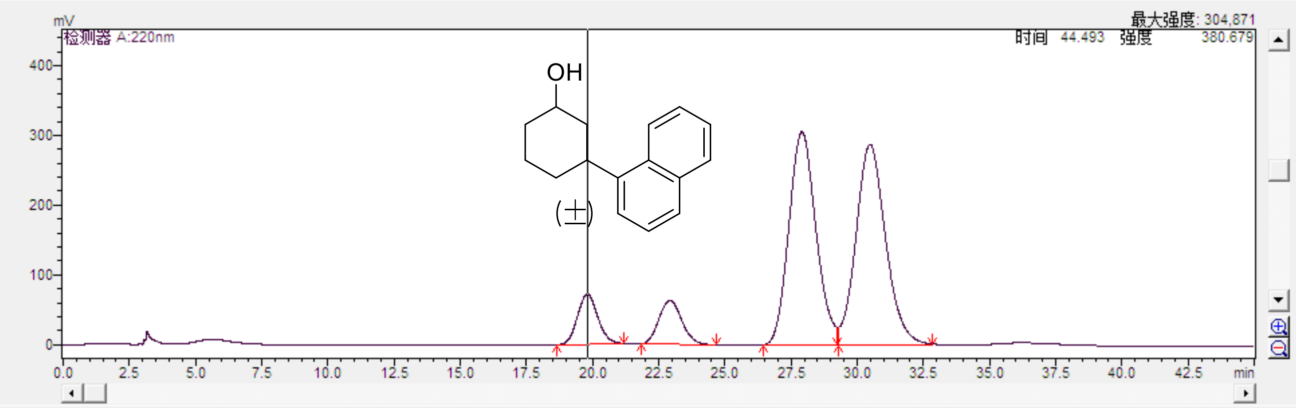

化合物表视图

ID#	名称	保留时间	峰#	面积	高度	面积%
1	RT19.803	19.803	1	3802773	71353	7.3255
2	RT22.920	22.920	2	3835154	63375	7.3879
3	RT27.906	27.906	3	21816077	305760	42.0256
4	RT30.479	30.479	4	22457369	287707	43.2610

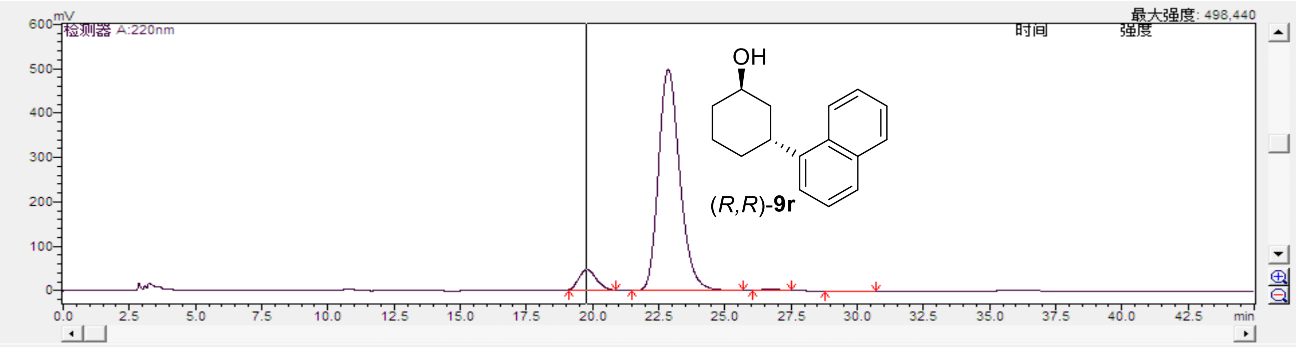

化合物表视图

ID#	名称	保留时间	峰#	面积	高度	面积%
1	RT19.788	19.788	1	2080436	45854	6.8827
2	RT22.856	22.856	2	28014264	498665	92.6800
3	RT26.695	26.695	3	128562	2567	0.4253
4	RT29.387	29.387	4	3622	99	0.0120

<u>**Translation of all characters (Chinese) in the above two frameworks to English is as follows:**</u>

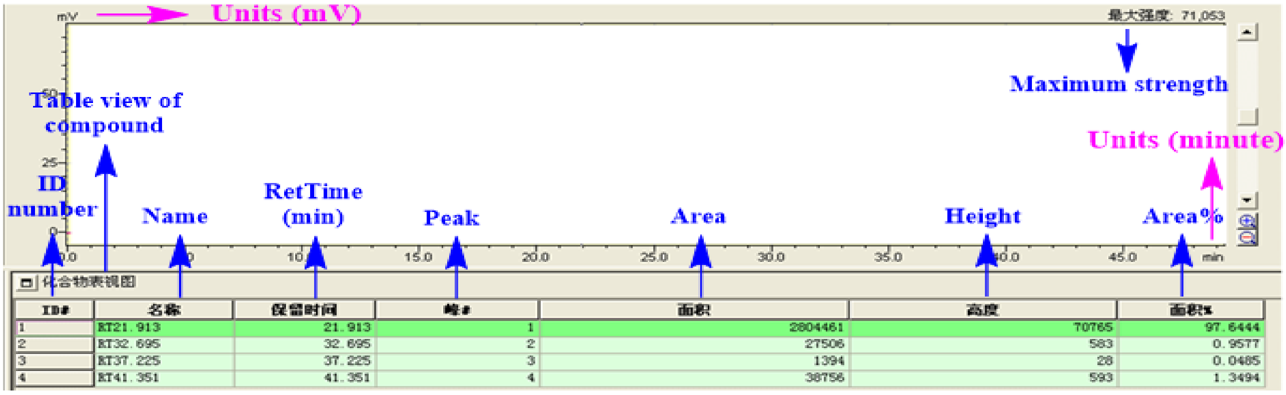

(*R*,*R*)-9s: (1*R*,3*R*)-3-(phenanthren-9-yl)cyclohexan-1-ol: (HPLC: Chiracel IC, detected at 220 nm, eluent: n-hexane/2-propanol = 98/2, flow rate = 1.0mL/min, 27°C).

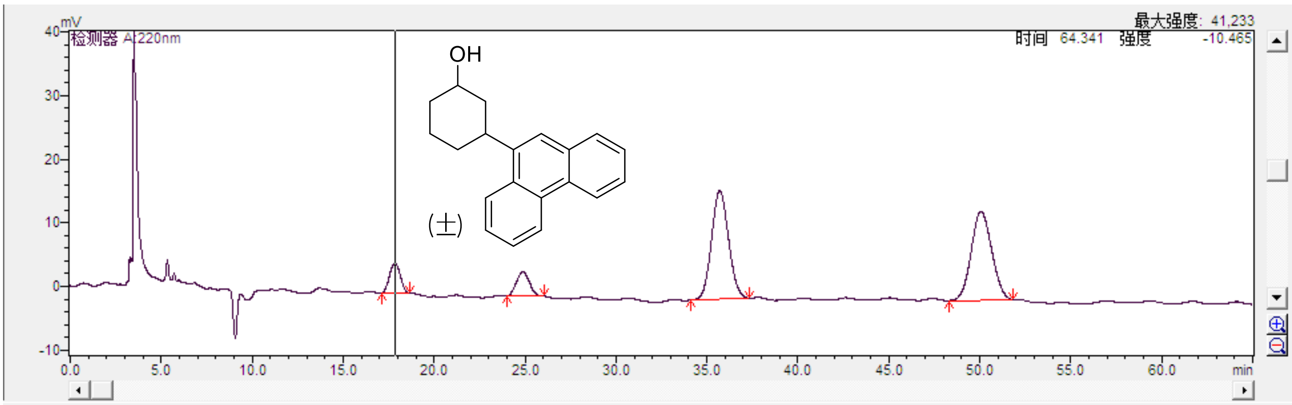

化合物表视图

ID#	名称	保留时间	峰#	面积	高度	面积%
1	RT17.841	17.841	1	193702	4676	7.5042
2	RT24.875	24.875	2	189234	3766	7.3311
3	RT35.696	35.696	3	1111966	17005	43.0788
4	RT50.060	50.060	4	1086337	13930	42.0859

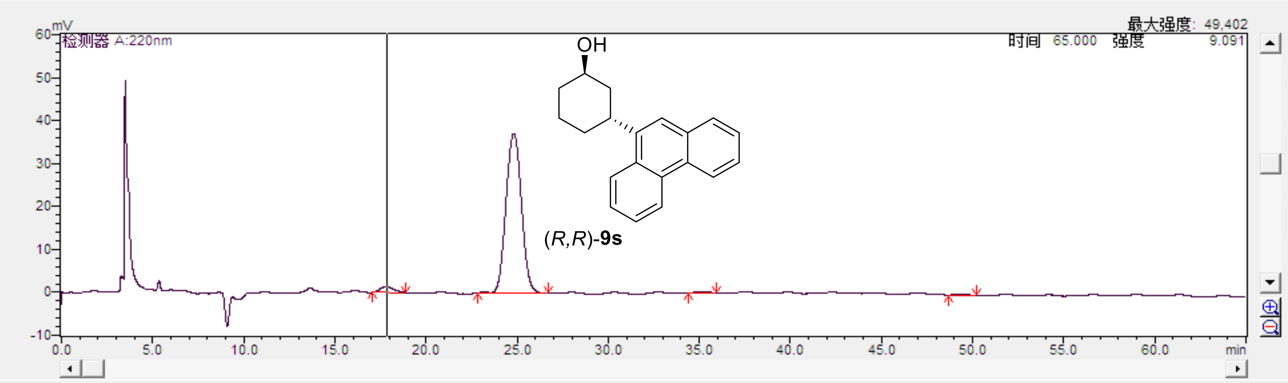

化合物表视图

ID#	名称	保留时间	峰#	面积	高度	面积%
1	RT17.842	17.842	1	68650	1301	2.9892
2	RT24.814	24.814	2	2196870	37127	95.6565
3	RT35.115	35.115	3	18663	411	0.8126
4	RT49.443	49.443	4	12440	296	0.5417

<u>Translation of all characters (Chinese) in the above two frameworks to English is as follows:</u>

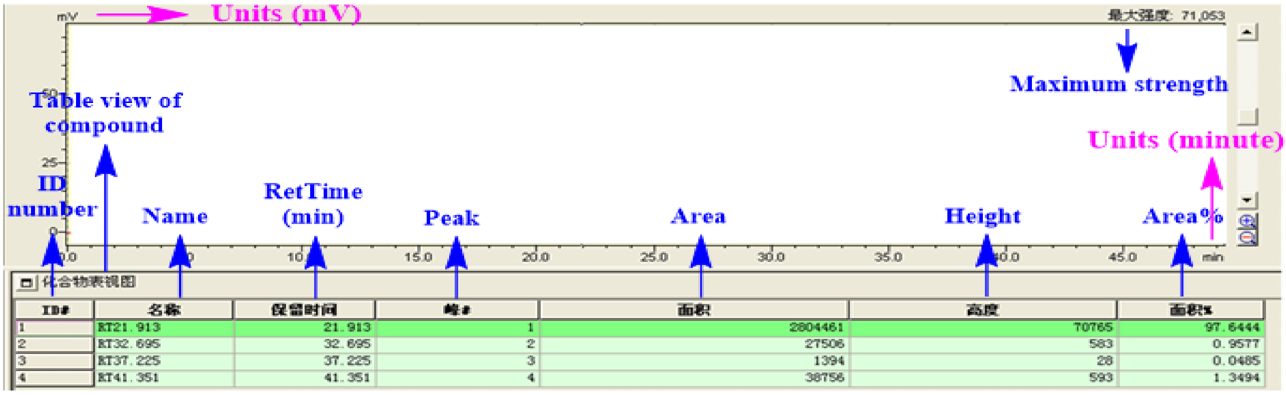

(*R*,*R*)-9t: *tert*-butyl (2*R*,4*R*)-4-hydroxy-2-phenylpiperidine-1-carboxylate: (HPLC: Chiracel OD+OD, detected at 254 nm, eluent: n-hexane/2-propanol = 98/2, flow rate = 1.0mL/min, 25°C).

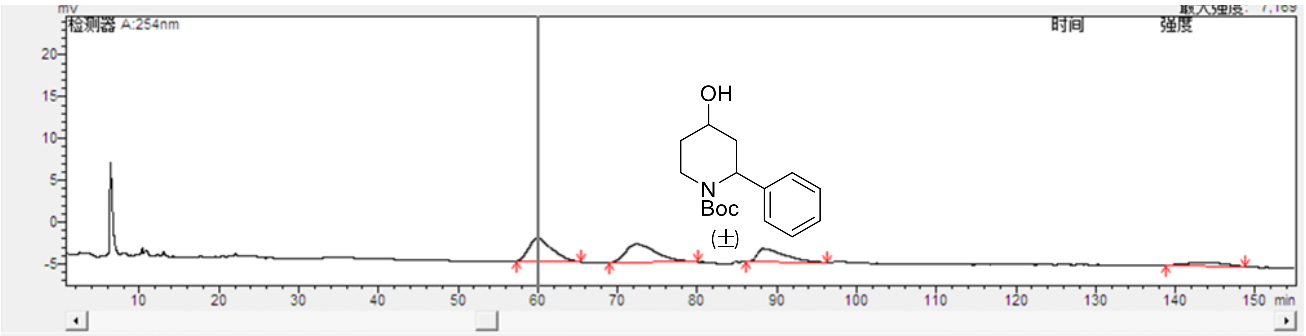

□ 化合物表视图

ID#	名称	保留时间	峰#	面积	高度	面积%
1	RT60.015	60.015	1	565071	2770	35.2510
2	RT72.346	72.346	2	602754	2213	37.6018
3	RT87.613	87.613	3	283877	11018	17.7092
4	RT142.955	142.955	4	151290	465	9.4380

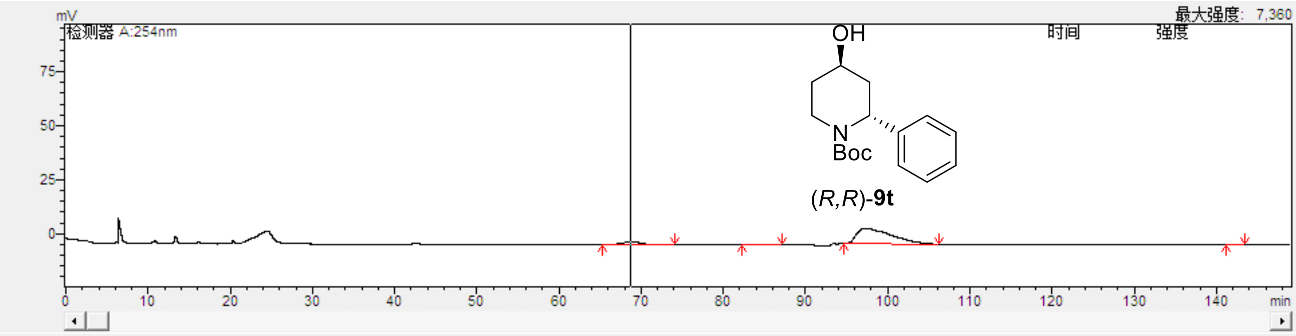

□ 化合物表视图

ID#	名称	保留时间	峰#	面积	高度	面积%
1	RT68.747	68.747	1	238250	1445	10.0767
2	RT85.318	85.318	2	22486	196	0.9510
3	RT97.302	97.302	3	2099411	7105	88.7940
4	RT141.747	141.747	4	4214	51	0.1782

<u>**Translation of all characters (Chinese) in the above two frameworks to English is as follows:**</u>

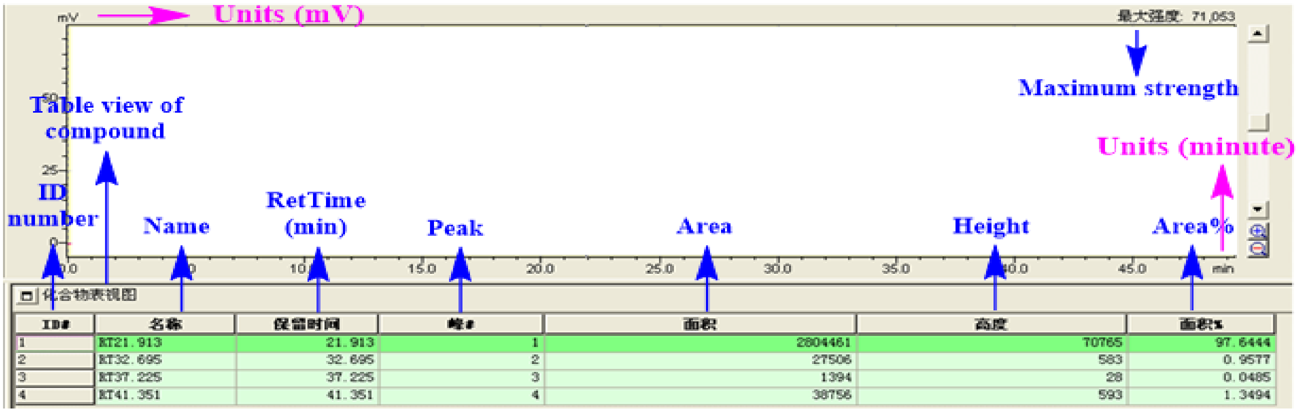

□ 化合物表视图

ID#	名称	保留时间	峰#	面积	高度	面积%
1	RT21.913	21.913	1	2804461	70765	97.6444
2	RT32.695	32.695	2	27506	583	0.9577
3	RT37.225	37.225	3	1394	28	0.0485
4	RT41.351	41.351	4	38756	593	1.3494

(*R*,*R*)-9u: (1*R*,3*R*)-3-phenylcyclopentan-1-ol: (HPLC: Chiracel OZ+OB, detected at 220 nm, eluent: n-hexane/2-propanol = 98/2, flow rate = 1.0mL/min, 25°C).

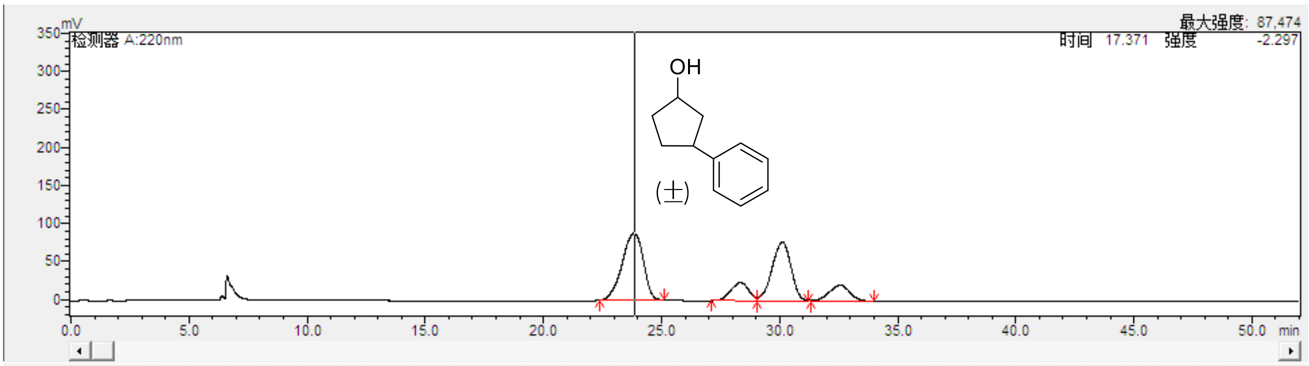

化合物表视图

ID#	名称	保留时间	峰#	面积	高度	面积%
1	RT23.840	23.840	1	5443611	87816	44.0183
2	RT28.347	28.347	2	1280710	23811	10.3561
3	RT30.114	30.114	3	4300015	77167	34.7710
4	RT32.575	32.575	4	1342349	21029	10.8546

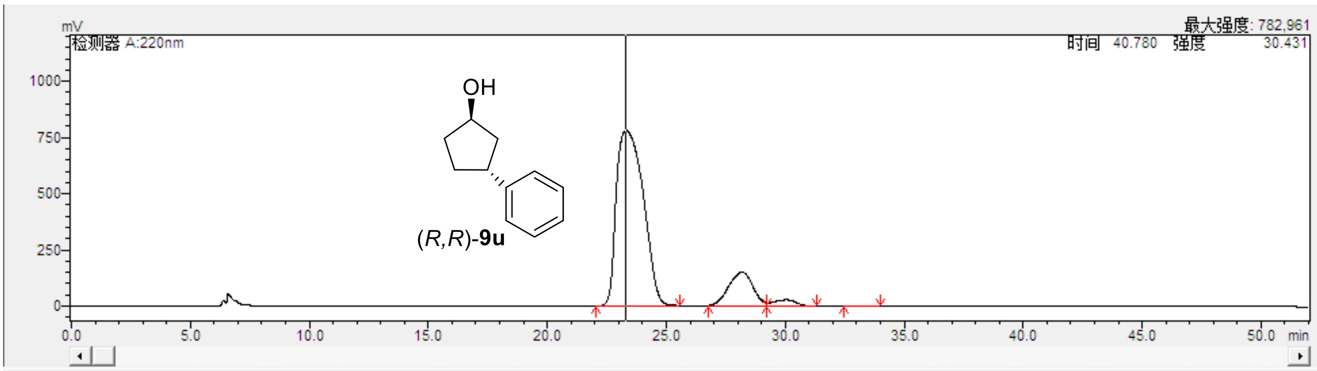

化合物表视图

ID#	名称	保留时间	峰#	面积	高度	面积%
1	RT23.316	23.316	1	61626426	781985	82.8013
2	RT28.179	28.179	2	10674880	150499	14.3428
3	RT30.036	30.036	3	2056382	29674	2.7630
4	RT33.027	33.027	4	69156	1738	0.0929

<u>Translation of all characters (Chinese) in the above two frameworks to English is as follows:</u>

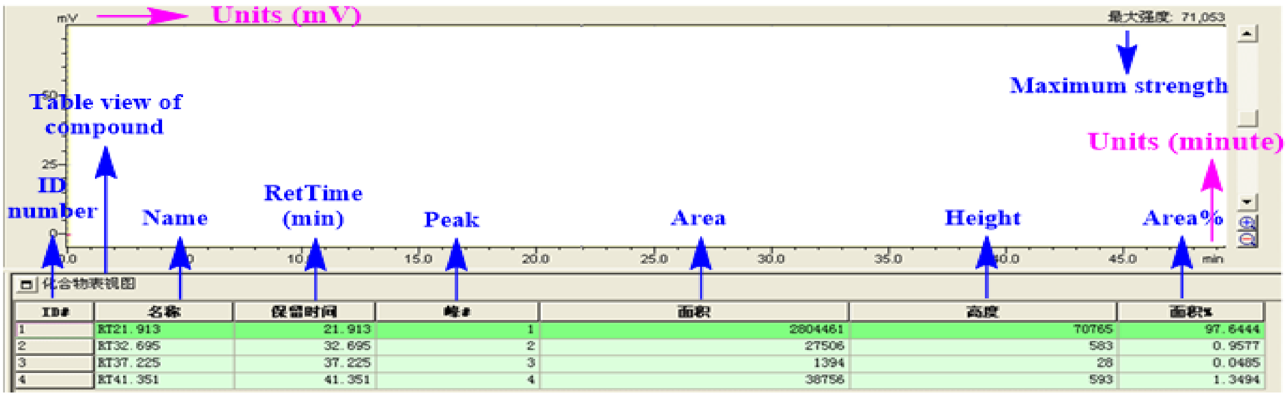

(*R*,*R*)-9v: (1*R*,3*R*)-3-phenylcycloheptan-1-ol: (HPLC: Chiracel AD, detected at 254 nm, eluent: n-hexane/2-propanol = 98/2, flow rate = 1.0mL/min, 27°C).

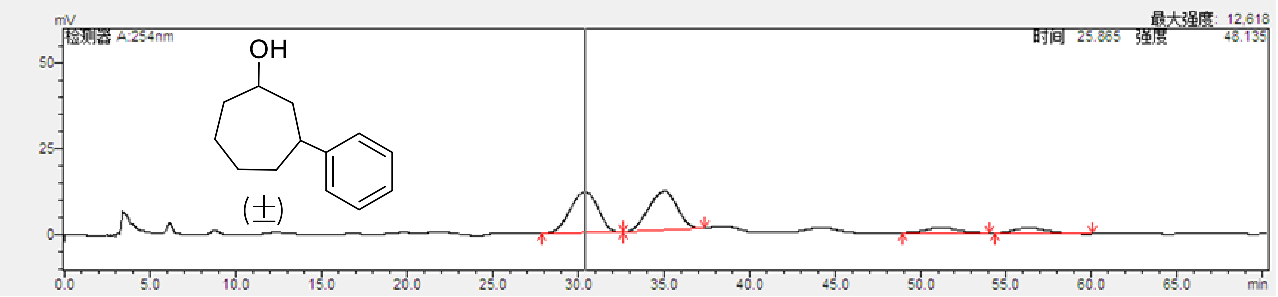

化合物表视图

ID#	名称	保留时间	峰#	面积	高度	面积%
1	RT30.405	30.405	1	1346990	11874	44.2429
2	RT35.028	35.028	2	1307893	11278	42.9587
3	RT51.293	51.293	3	189181	1524	6.2138
4	RT56.332	56.332	4	200473	1600	6.5847

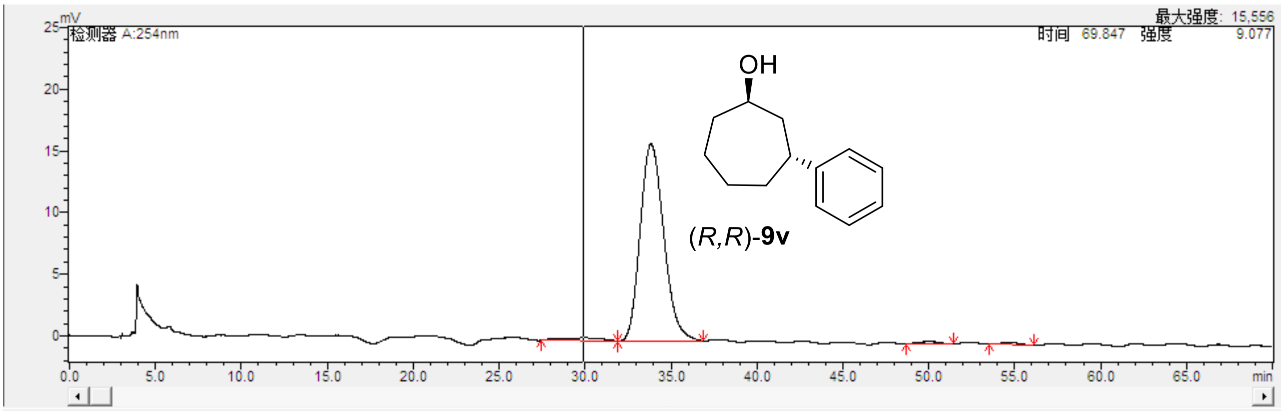

化合物表视图

ID#	名称	保留时间	峰#	面积	高度	面积%
1	RT29.904	29.904	1	38962	260	2.4411
2	RT33.834	33.834	2	1525487	15968	95.5780
3	RT50.095	50.095	3	16431	189	1.0294
4	RT54.688	54.688	4	15186	163	0.9515

<u>Translation of all characters (Chinese) in the above two frameworks to English is as follows:</u>

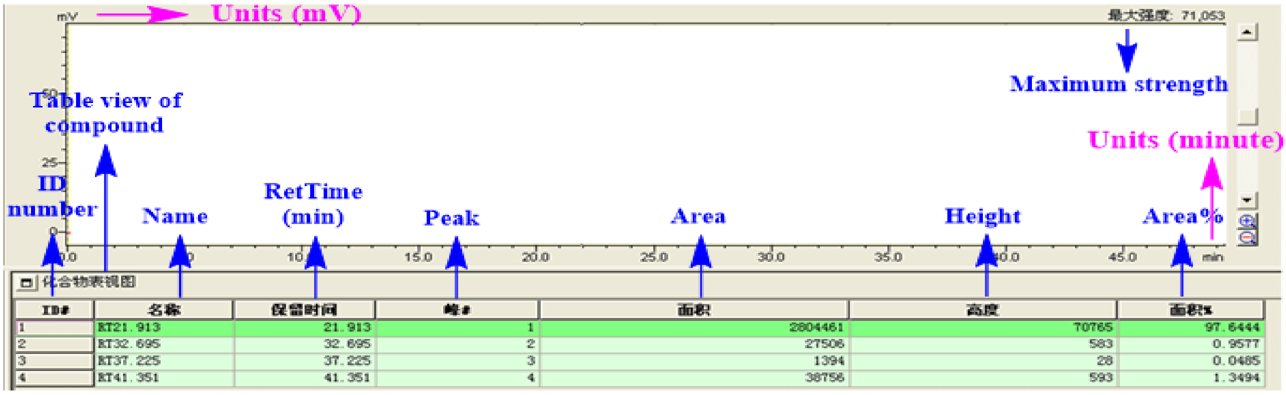

(*R*,*R*)-9w: (1*R*,3*R*)-3-(m-tolyl)cycloheptan-1-ol: (HPLC: Chiracel AS, detected at 254 nm, eluent: n-hexane/2-propanol = 98/2, flow rate = 1.0mL/min, 27°C).

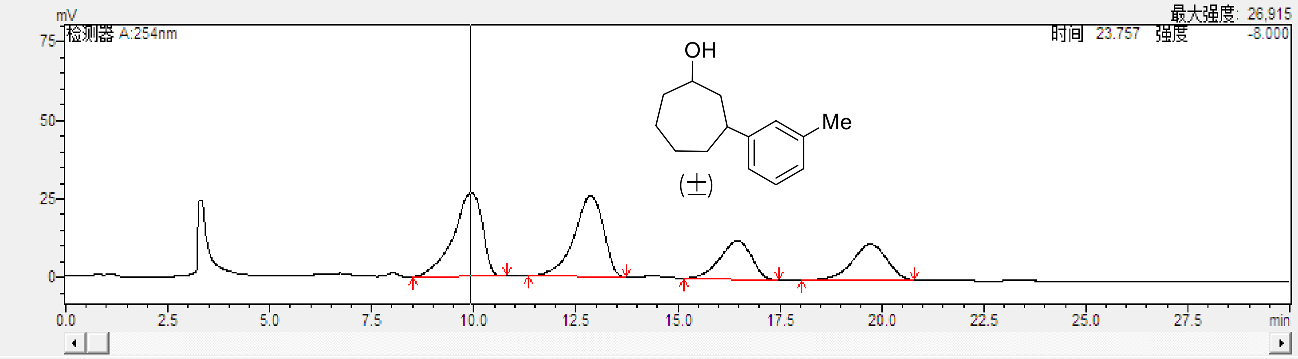

化合物表视图

ID#	名称	保留时间	峰#	面积	高度	面积%
1	RT9.926	9.926	1	1311308	26549	33.3867
2	RT12.855	12.855	2	1292679	25729	32.9124
3	RT16.461	16.461	3	656952	12274	16.7264
4	RT19.720	19.720	4	666701	11382	16.9746

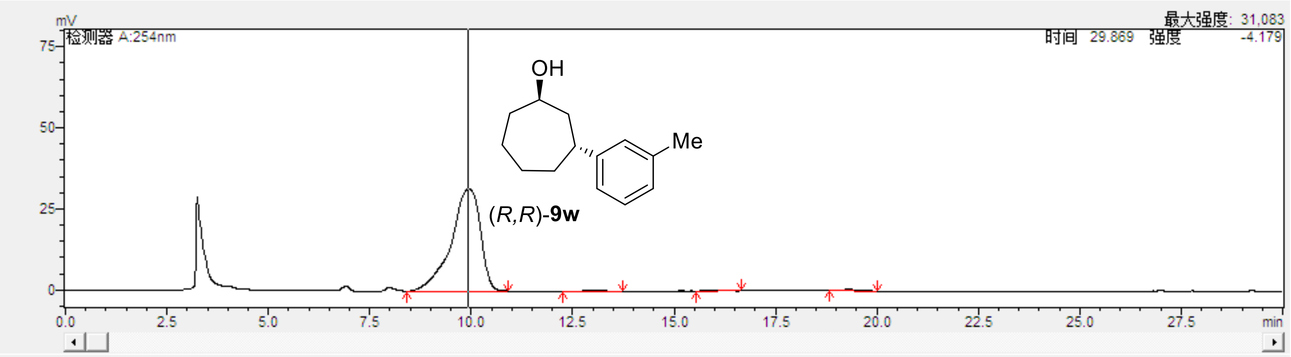

化合物表视图

ID#	名称	保留时间	峰#	面积	高度	面积%
1	RT9.933	9.933	1	1579544	31412	98.4730
2	RT12.964	12.964	2	10741	243	0.6696
3	RT16.148	16.148	3	772	28	0.0482
4	RT19.274	19.274	4	12979	403	0.8092

Translation of all characters (Chinese) in the above two frameworks to English is as follows:

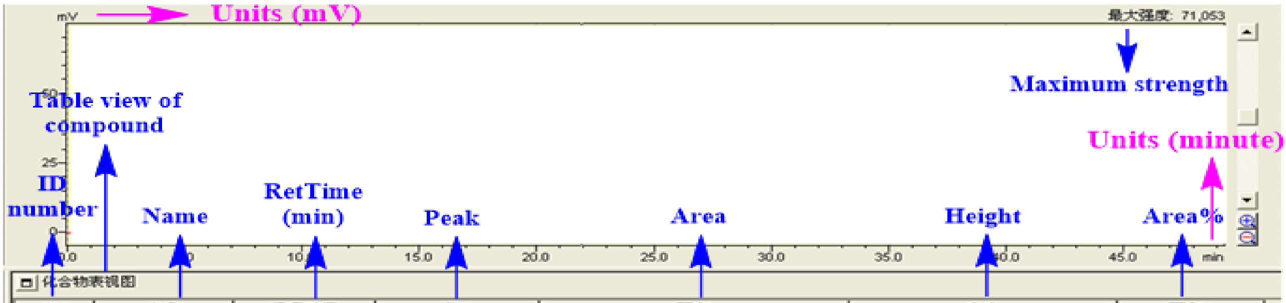

ID#	名称	保留时间	峰#	面积	高度	面积%
1	RT21.913	21.913	1	2804461	70765	97.6444
2	RT32.695	32.695	2	27506	583	0.9577
3	RT37.225	37.225	3	1394	28	0.0485
4	RT41.351	41.351	4	38756	593	1.3494

(*R*,*R*)-9x: (1*R*,3*R*)-3-(p-tolyl)cycloheptan-1-ol: (HPLC: Chiracel IC, detected at 254 nm, eluent: n-hexane/2-propanol = 98/2, flow rate = 1.0mL/min, 27°C).

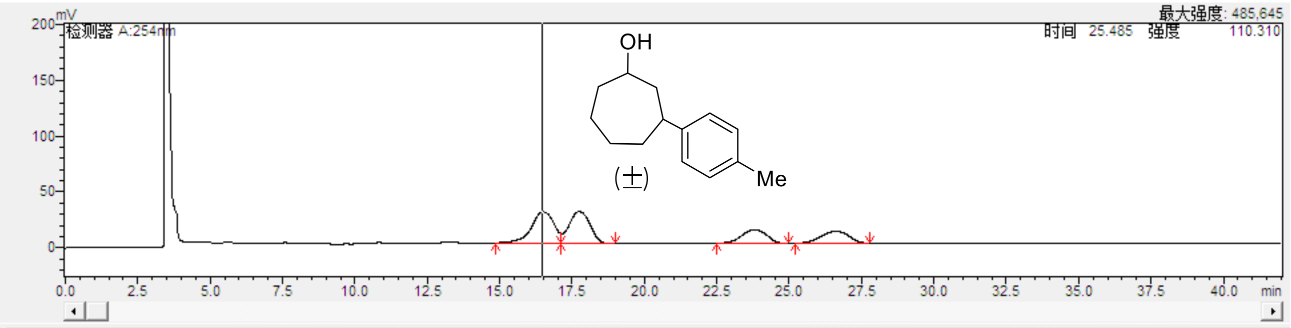

化合物表视图

ID#	名称	保留时间	峰#	面积	高度	面积%
1	RT16.489	16.489	1	1548482	28088	34.8887
2	RT17.739	17.739	2	1441807	28532	32.4852
3	RT23.805	23.805	3	728647	11895	16.4171
4	RT26.597	26.597	4	719409	10603	16.2090

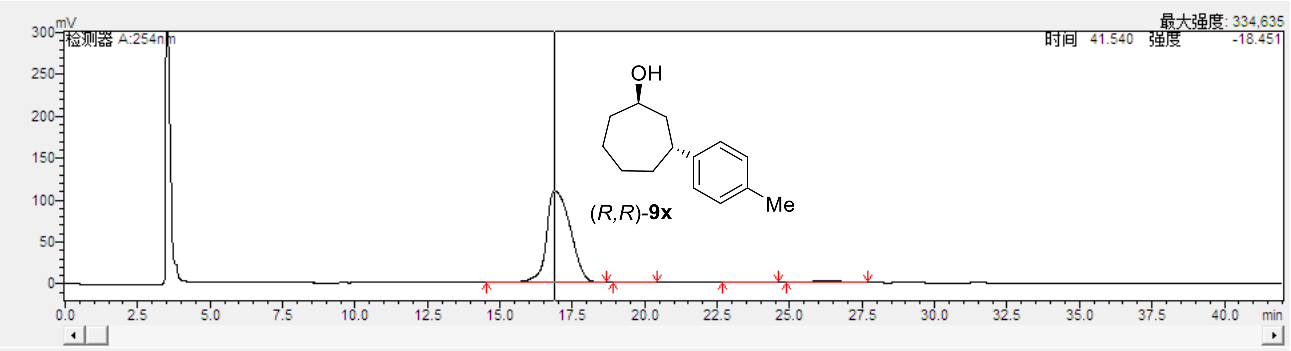

化合物表视图

ID#	名称	保留时间	峰#	面积	高度	面积%
1	RT16.893	16.893	1	6262604	109116	97.3545
2	RT19.441	19.441	2	4120	91	0.0640
3	RT23.366	23.366	3	7835	129	0.1218
4	RT26.354	26.354	4	158227	2027	2.4597

<u>**Translation of all characters (Chinese) in the above two frameworks to English is as follows:**</u>

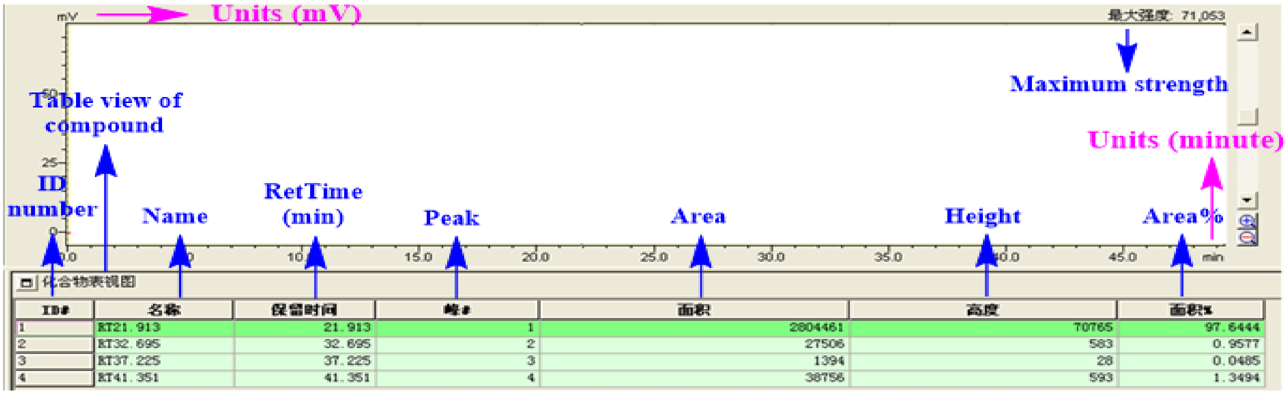

Figure S9. Characterization of chiral products.

(*R*,*R*)-9a: (1*R*,3*R*)-3-phenylcyclohexan-1-ol.

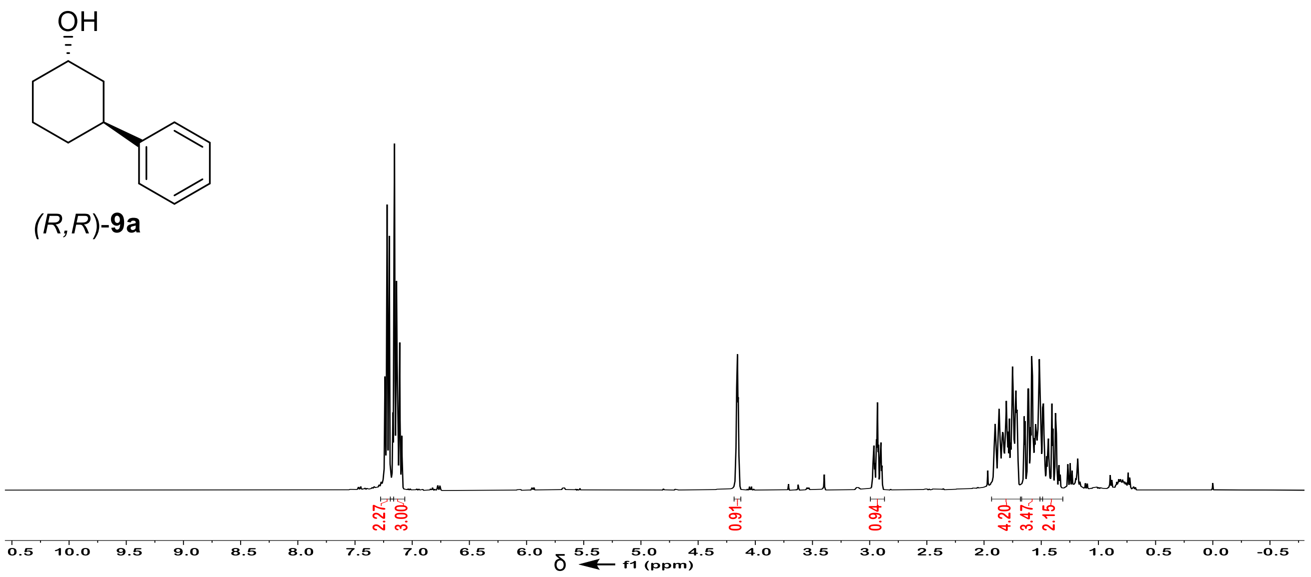

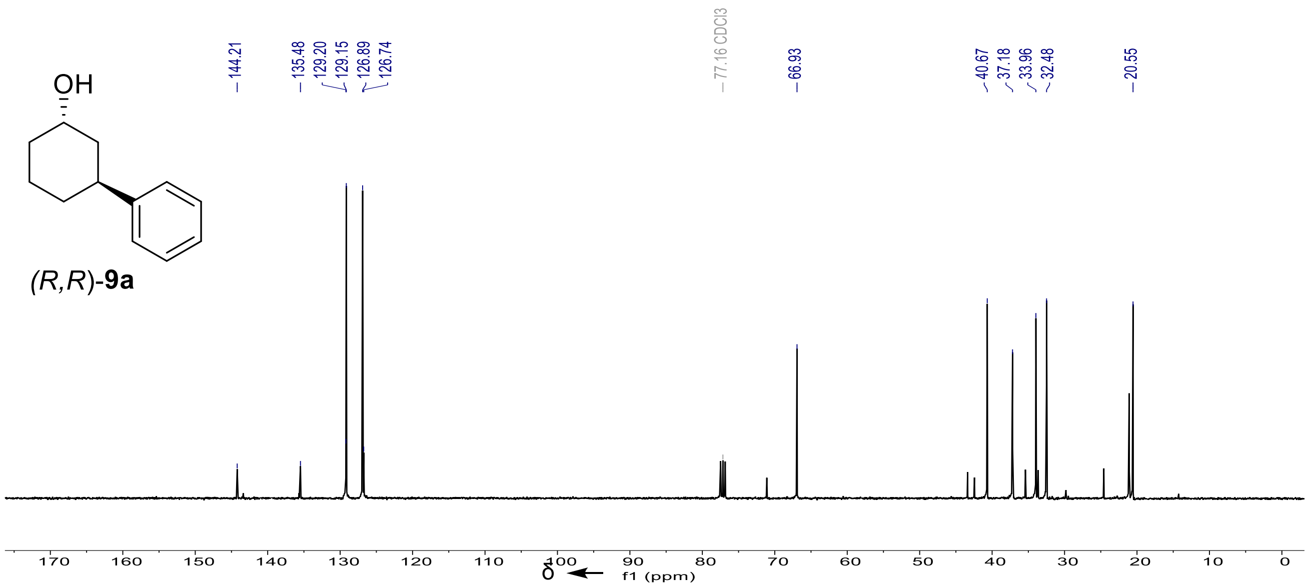

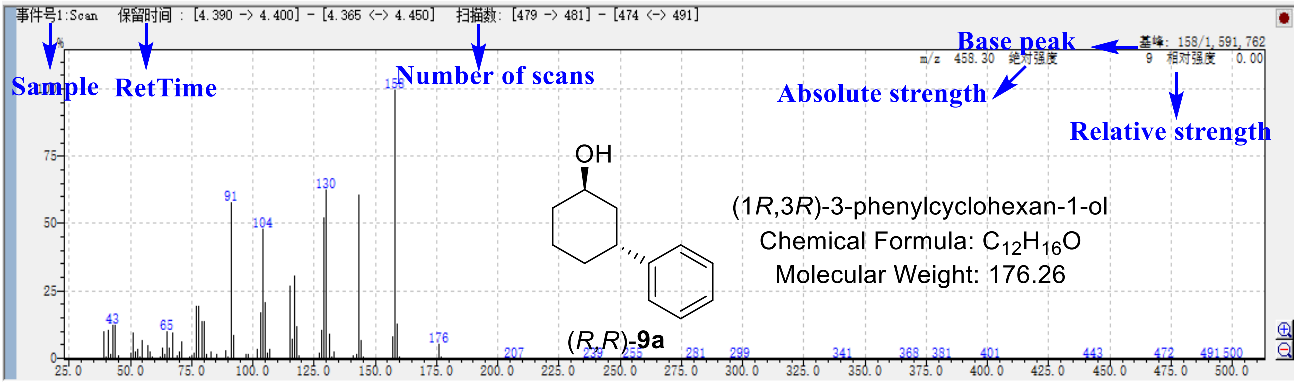

(***R***,***R***)-9b: (1***R***,3***R***)-3-(4-fluorophenyl)cyclohexan-1-ol

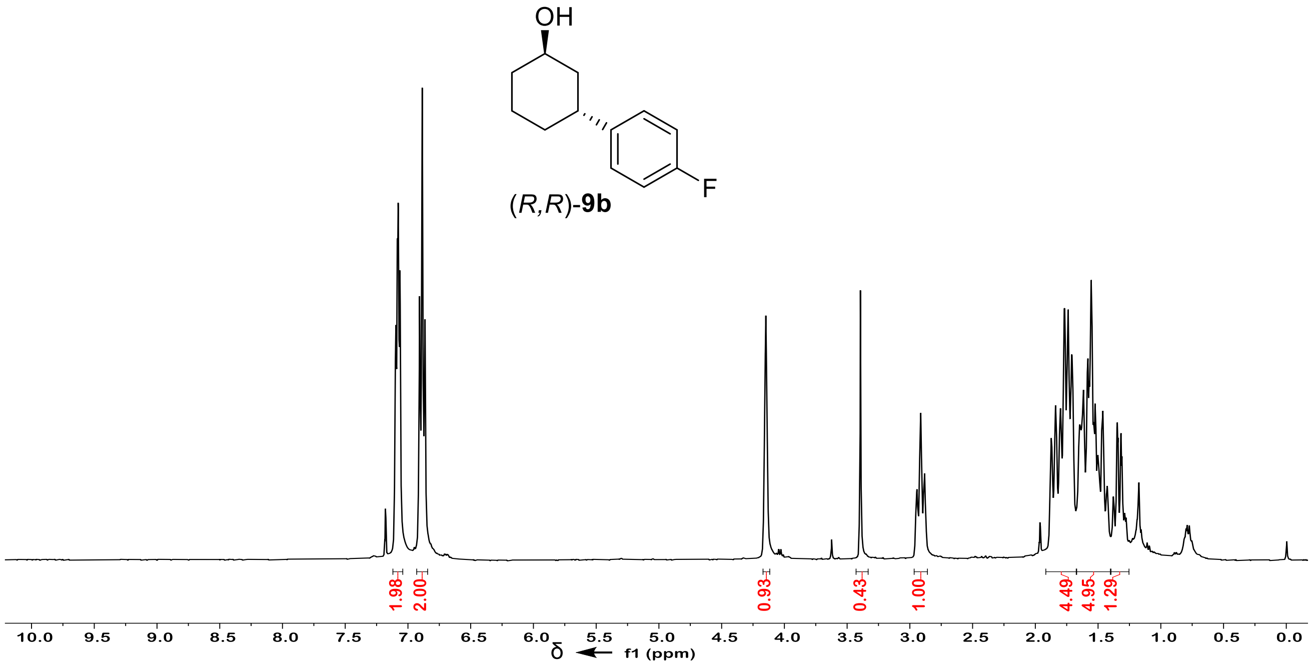

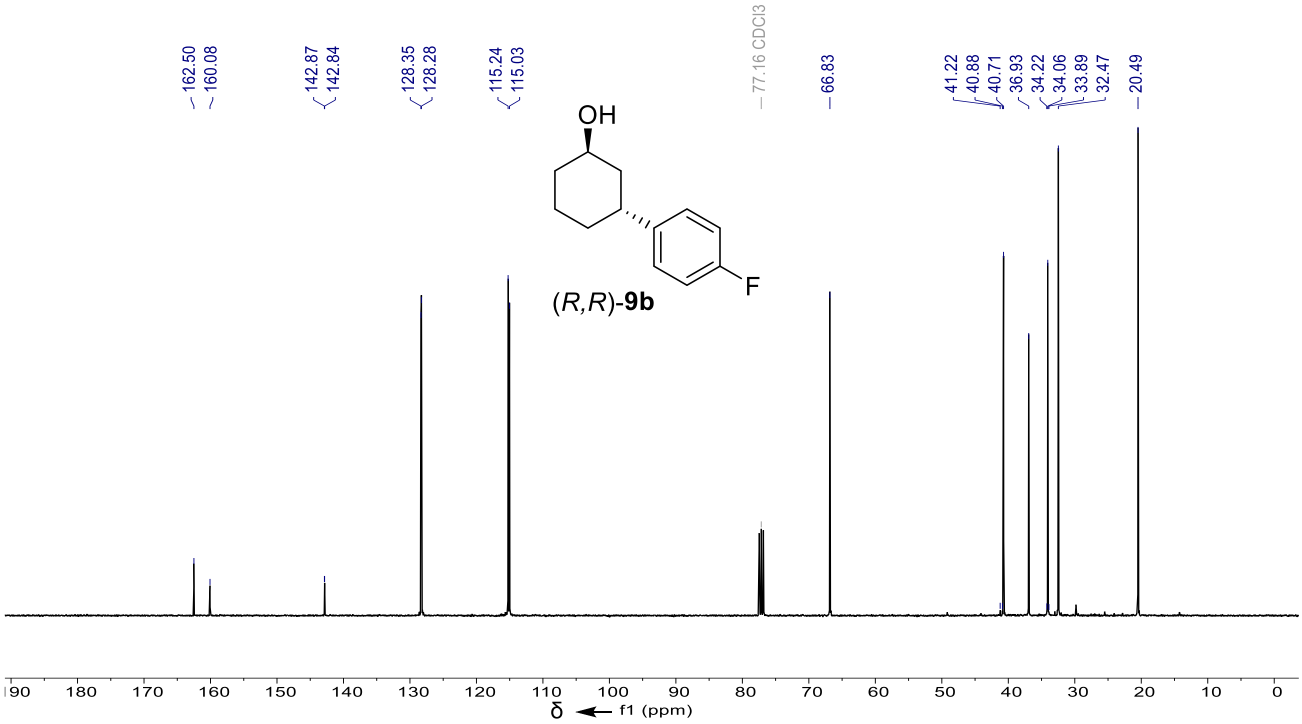

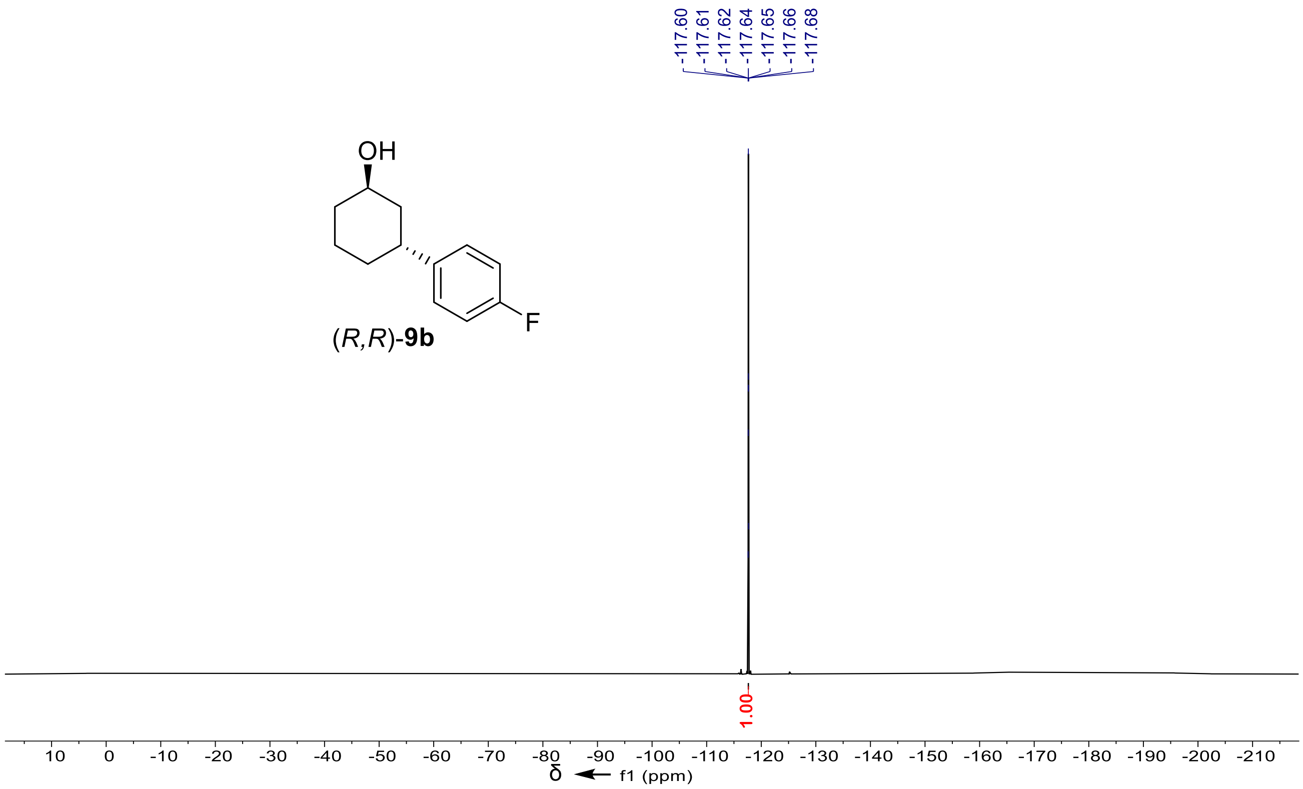
-117.60
-117.61
-117.62
-117.64
-117.65
-117.66
-117.68
OH
F
(R,R)-9b
1.00
10 0 -10 -20 -30 -40 -50 -60 -70 -80 -90 -100 -110 -120 -130 -140 -150 -160 -170 -180 -190 -200 -210
δ ← f1 (ppm)

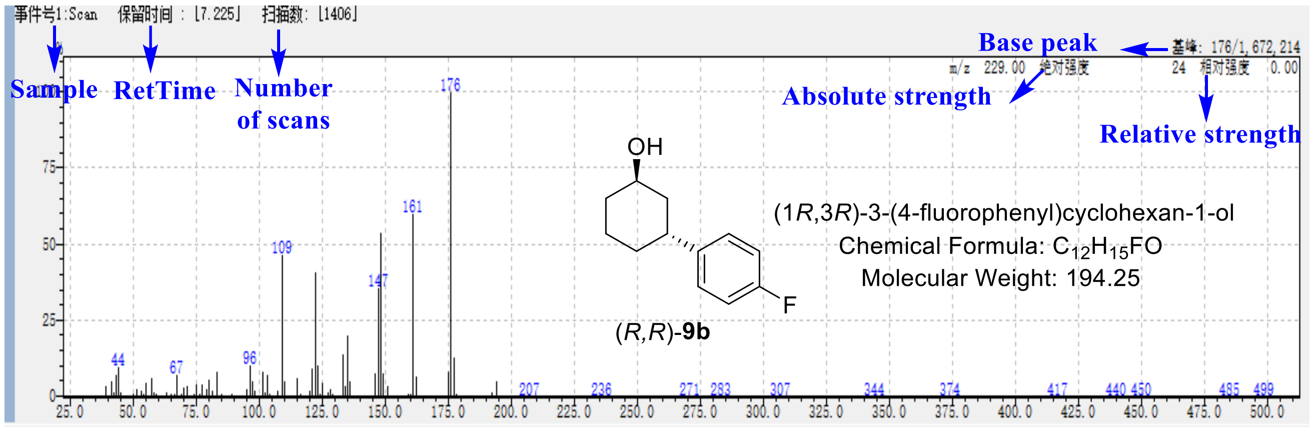
事件号1:Scan 保留时间：[7.225] 扫描数：[1406]
Sample
RetTime
Number of scans
Base peak
基峰：176/1,672,214
m/z 229.00 绝对强度
24 相对强度 0.00
Absolute strength
Relative strength
176
161
109
147
96
67
44
207 236 271 283 307 344 374 417 440 450 485 499
75
50
25
0
25.0 50.0 75.0 100.0 125.0 150.0 175.0 200.0 225.0 250.0 275.0 300.0 325.0 350.0 375.0 400.0 425.0 450.0 475.0 500.0
OH
F
(R,R)-9b
(1R,3R)-3-(4-fluorophenyl)cyclohexan-1-ol
Chemical Formula: C12H15FO
Molecular Weight: 194.25

(*R*,*R*)-9c: (1*R*,3*R*)-3-(3-chlorophenyl)cyclohexan-1-ol

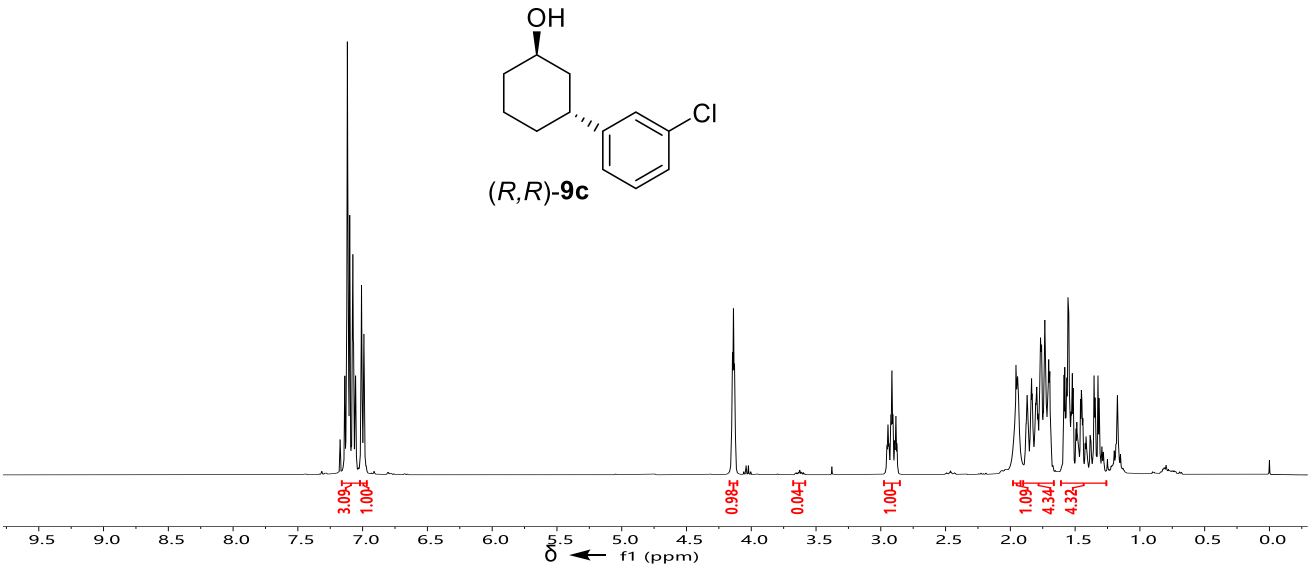

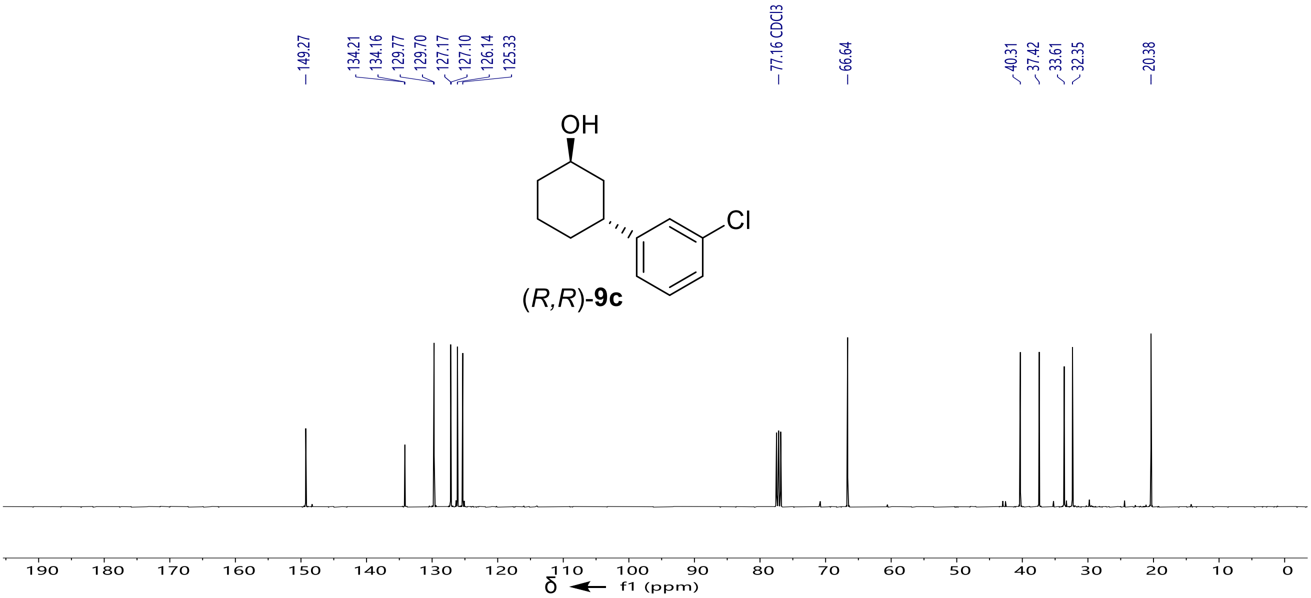

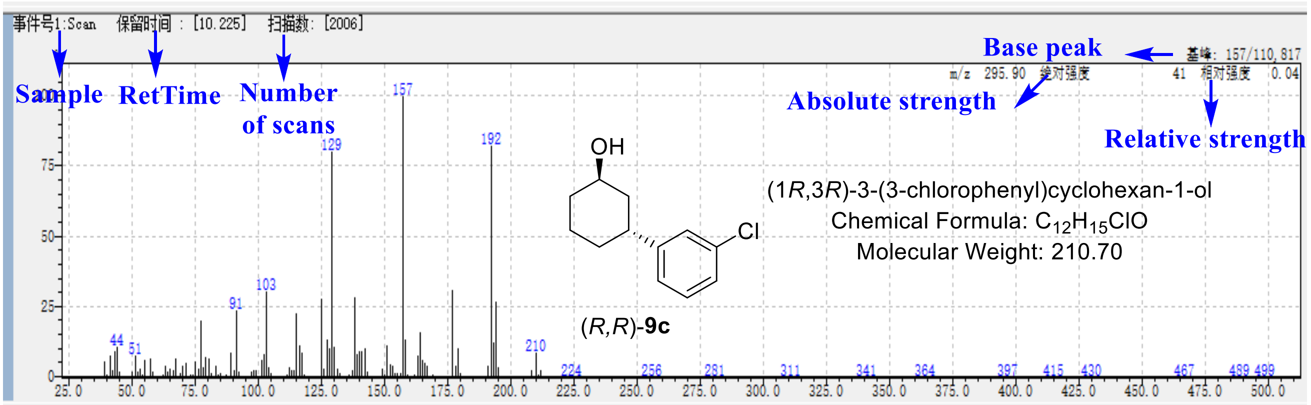

(*R*,*R*)-9d: (1*R*,3*R*)-3-(4-chlorophenyl)cyclohexan-1-ol

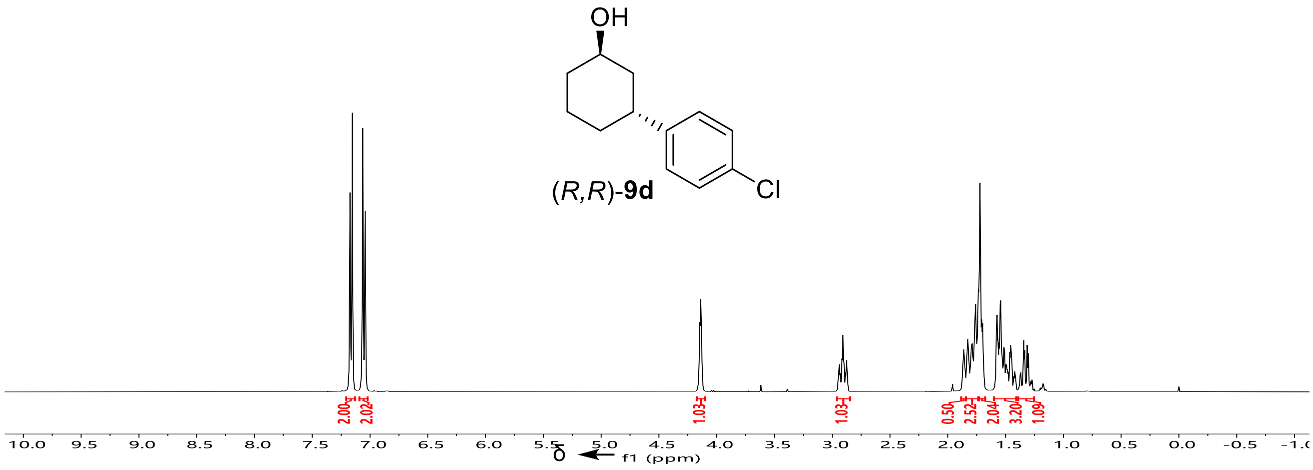

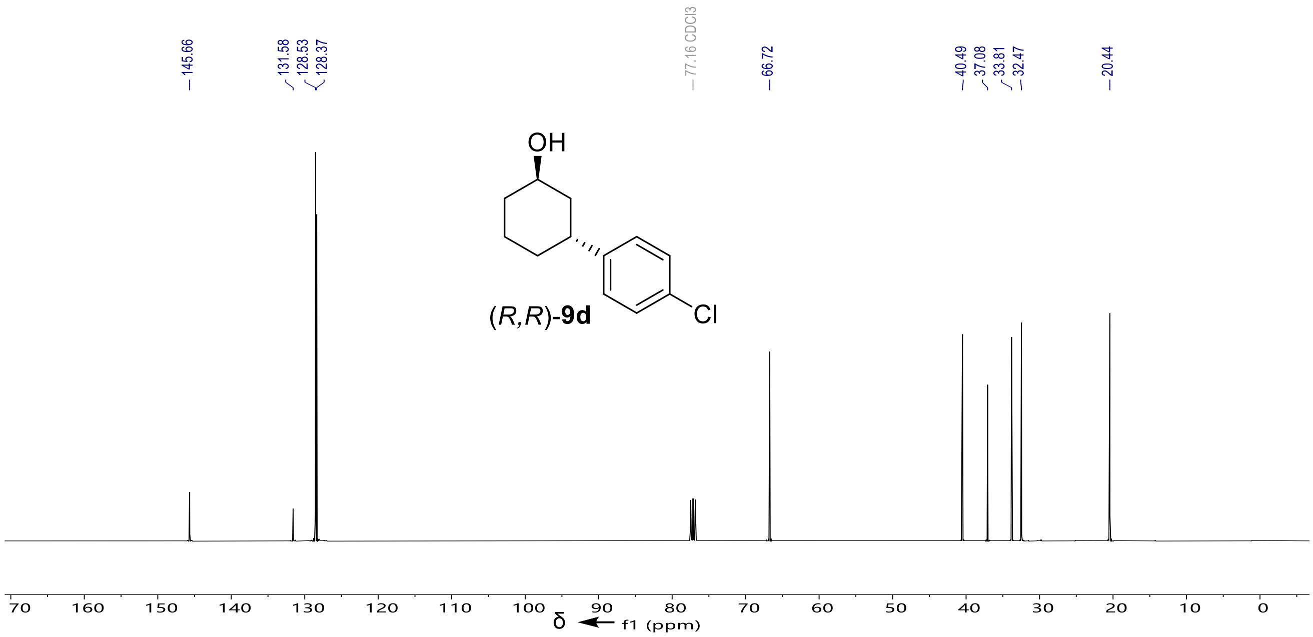

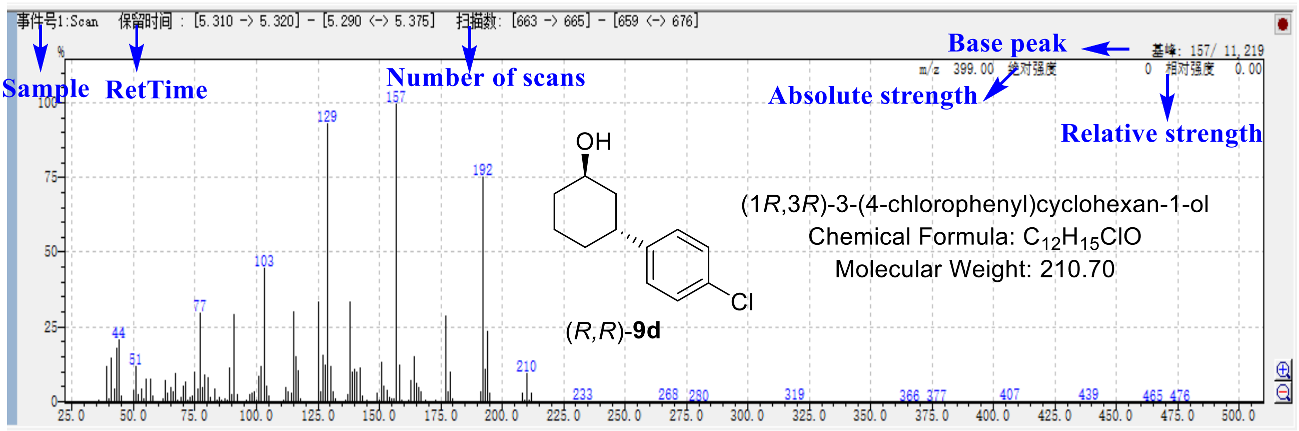

(*R*,*R*)-9e: (1*R*,3*R*)-3-(4-bromophenyl)cyclohexan-1-ol

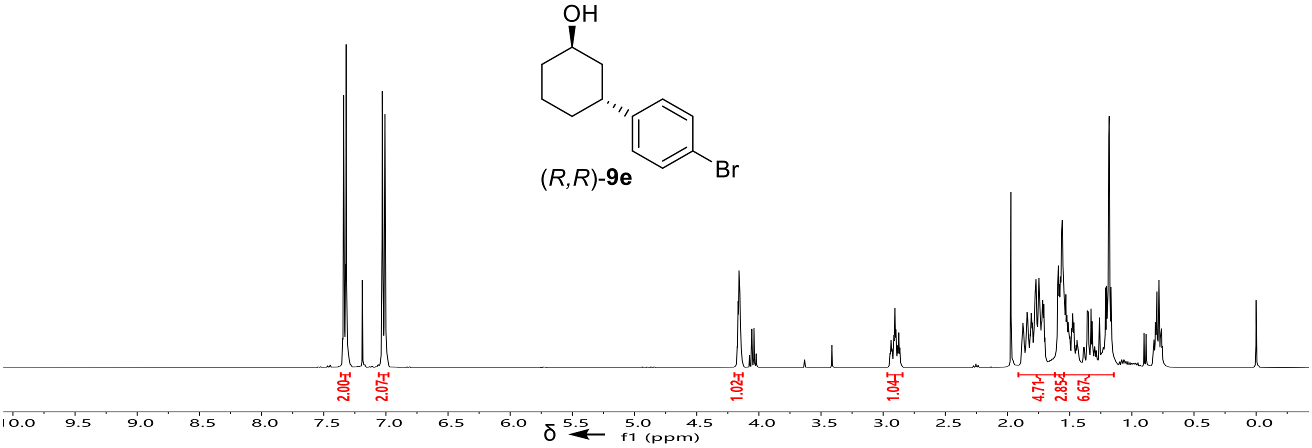

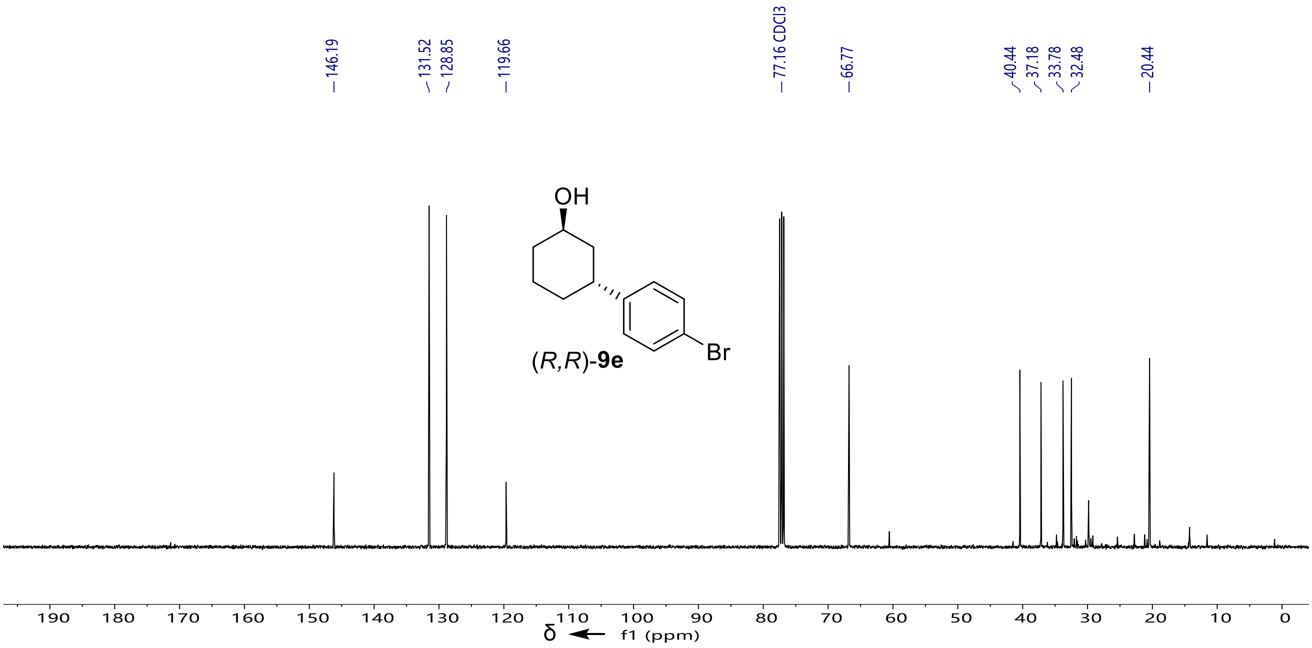

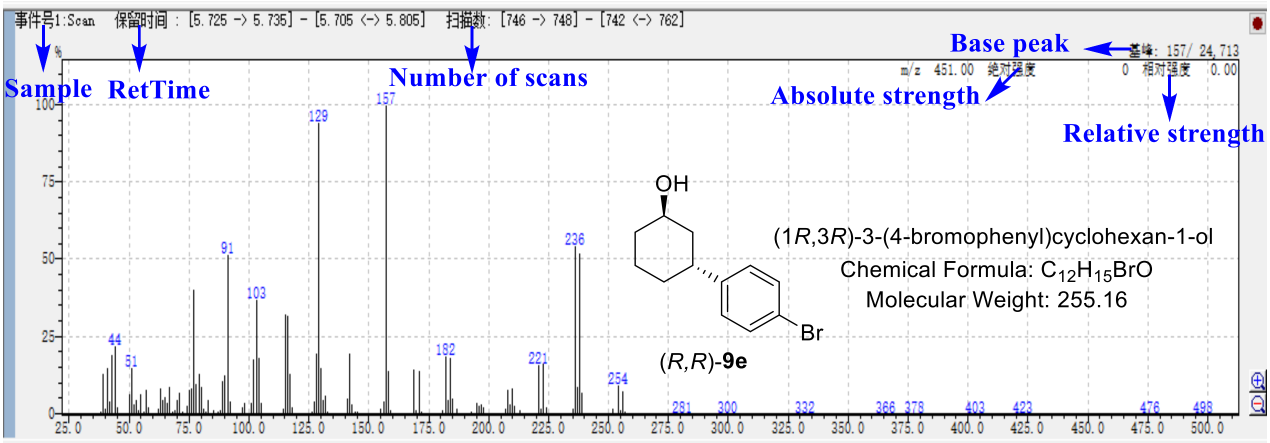

(*R*,*R*)-9f: (1*R*,3*R*)-3-(4-(trifluoromethyl)phenyl)cyclohexan-1-ol

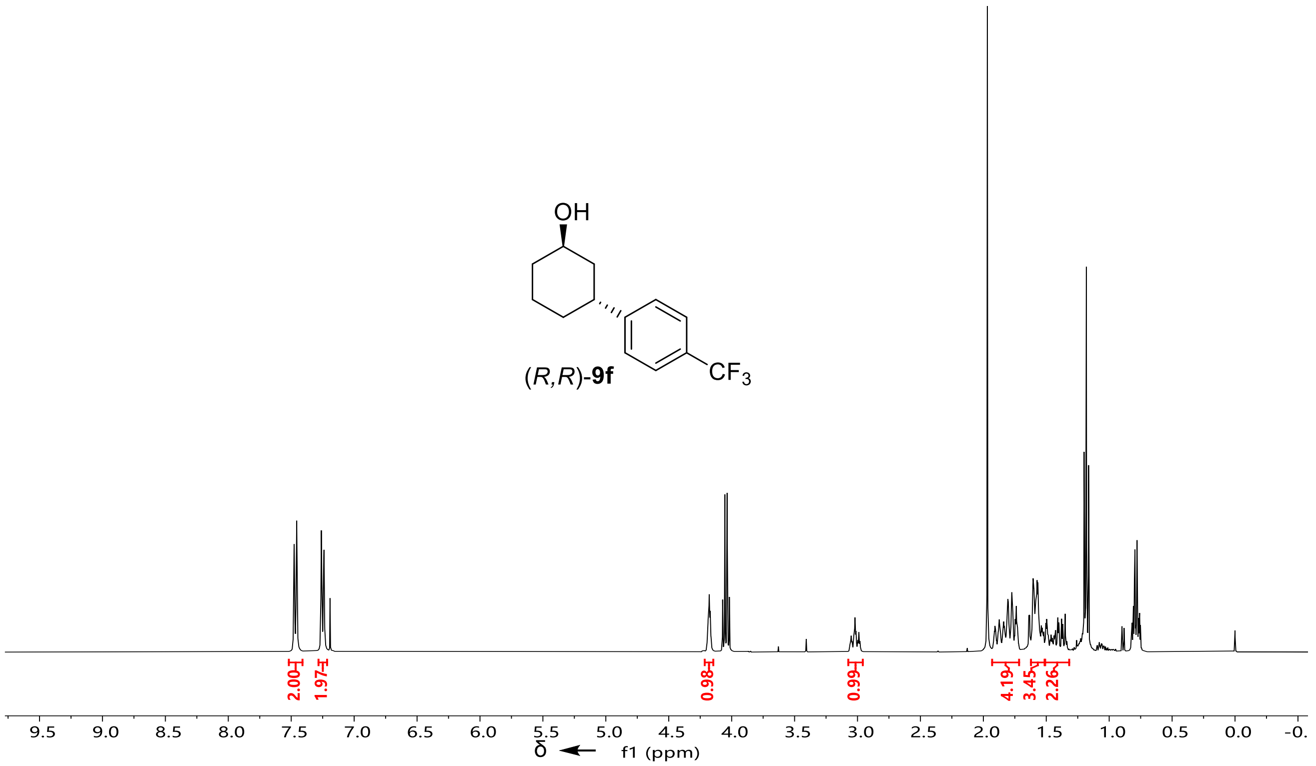

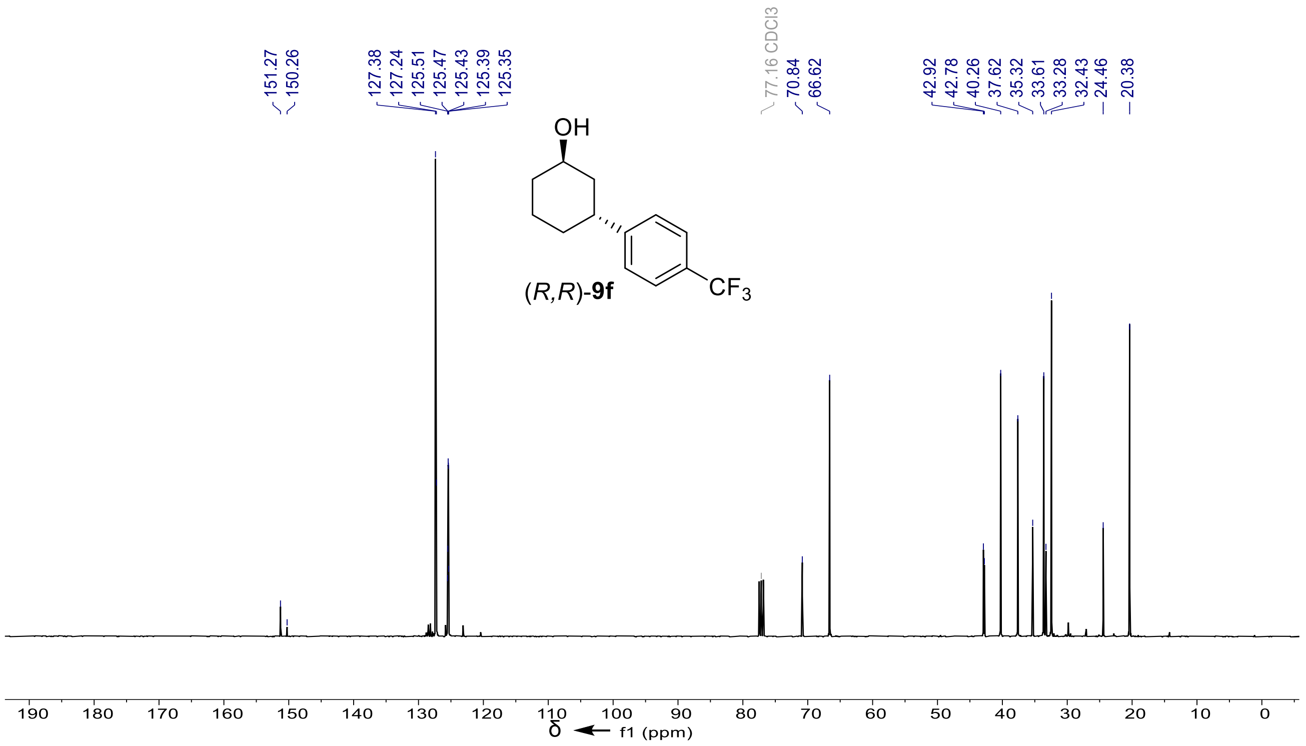

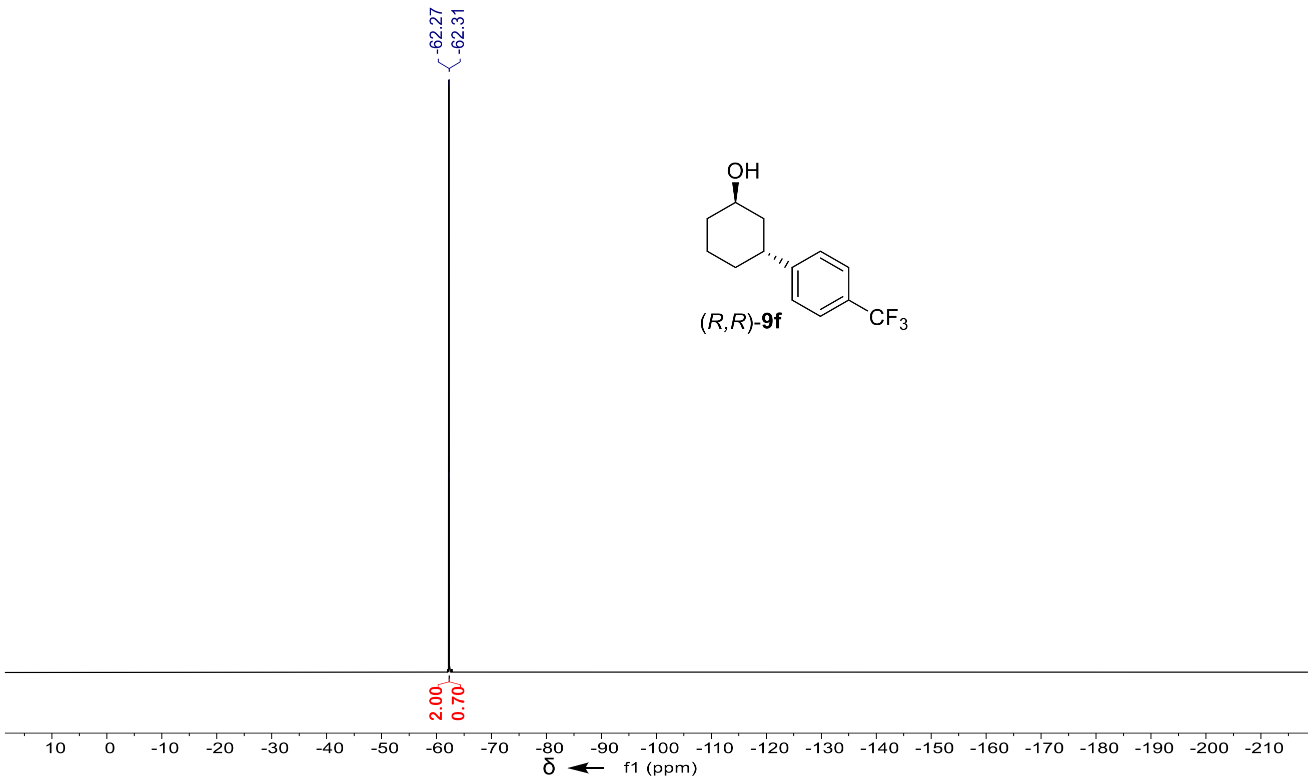
-62.27
-62.31
OH
(R,R)-9f
CF3
2.00
0.70
10
0
-10
-20
-30
-40
-50
-60
-70
-80
-90
-100
-110
-120
-130
-140
-150
-160
-170
-180
-190
-200
-210
δ
f1 (ppm)

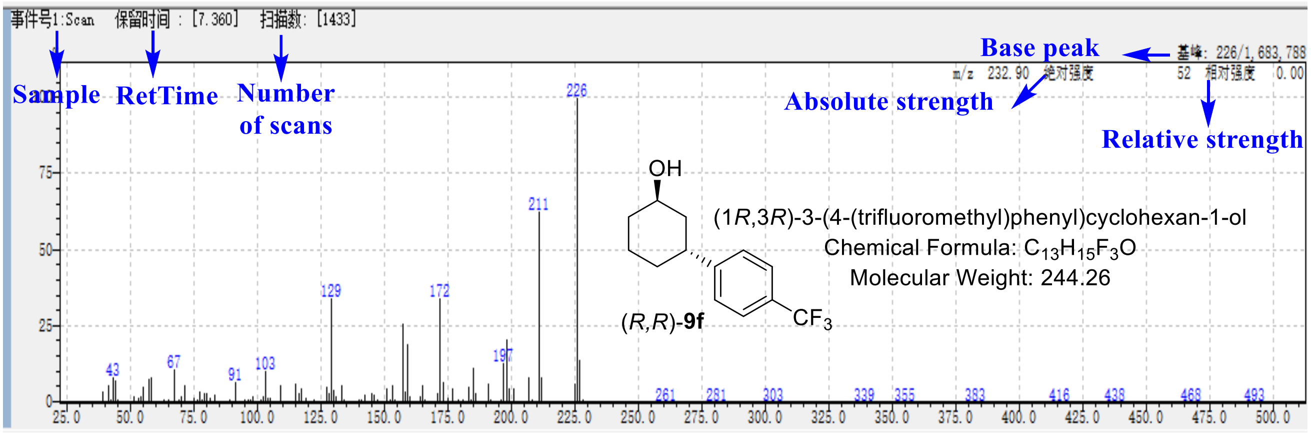
事件号1:Scan 保留时间 : [7.360] 扫描数: [1433]
Sample
RetTime
Number of scans
Base peak
基峰: 226/1,683,788
m/z 232.90 绝对强度
52 相对强度 0.00
Absolute strength
Relative strength
226
211
129
172
197
43
67
91
103
OH
(1R,3R)-3-(4-(trifluoromethyl)phenyl)cyclohexan-1-ol
Chemical Formula: C13H15F3O
Molecular Weight: 244.26
(R,R)-9f
CF3
261
281
303
339
355
383
416
438
468
493
75
50
25
0
25.0
50.0
75.0
100.0
125.0
150.0
175.0
200.0
225.0
250.0
275.0
300.0
325.0
350.0
375.0
400.0
425.0
450.0
475.0
500.0

(*R*,*R*)-9g: methyl 4-((1*R*,3*R*)-3-hydroxycyclohexyl)benzoate

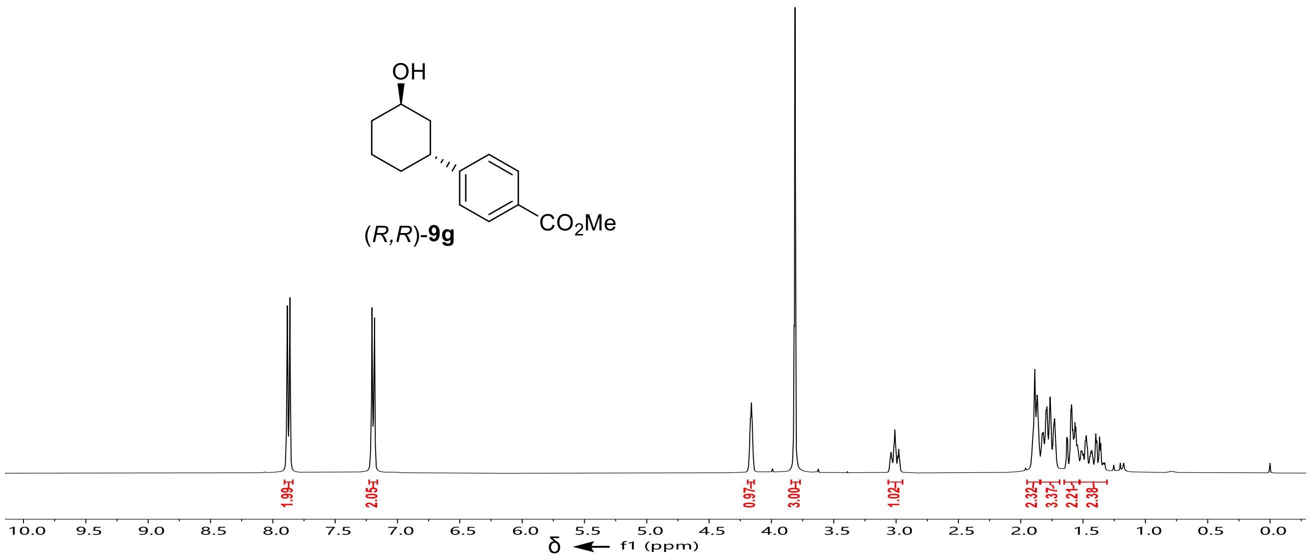

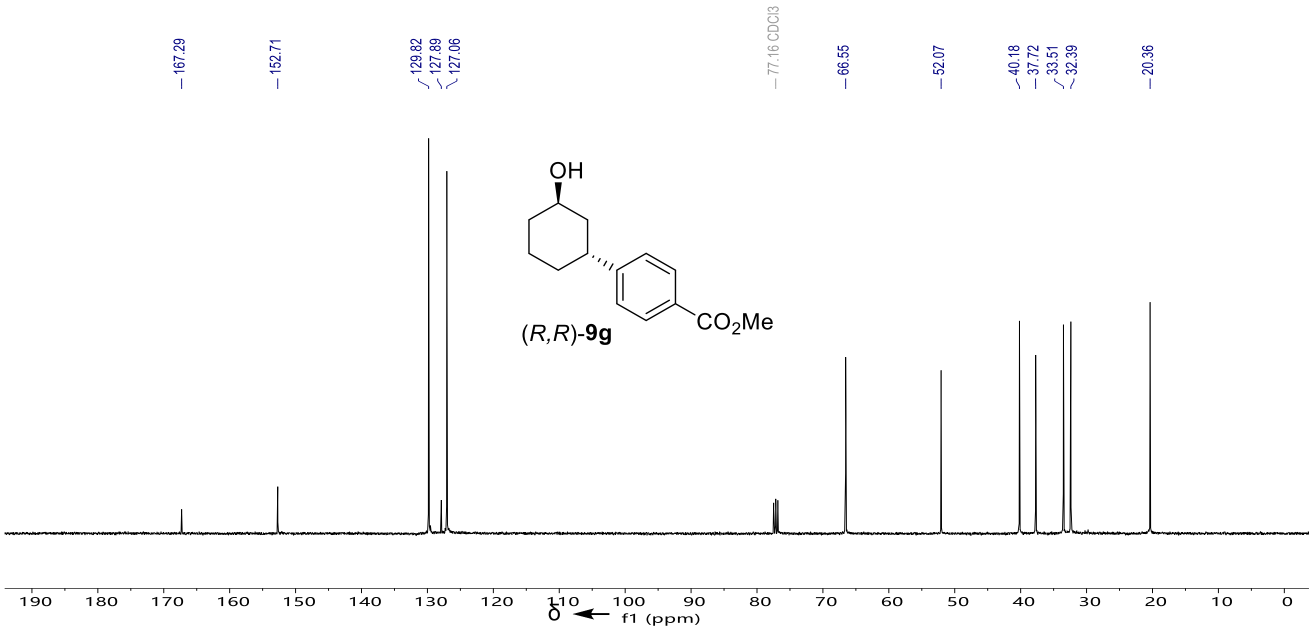

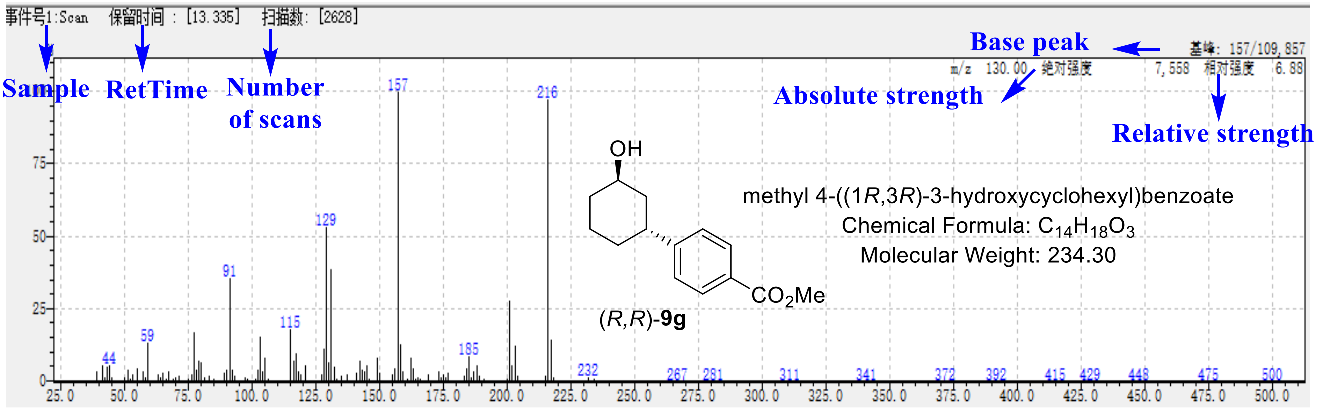

(*R*,*R*)-9h: (1*R*,3*R*)-3-(2-methoxyphenyl)cyclohexan-1-ol

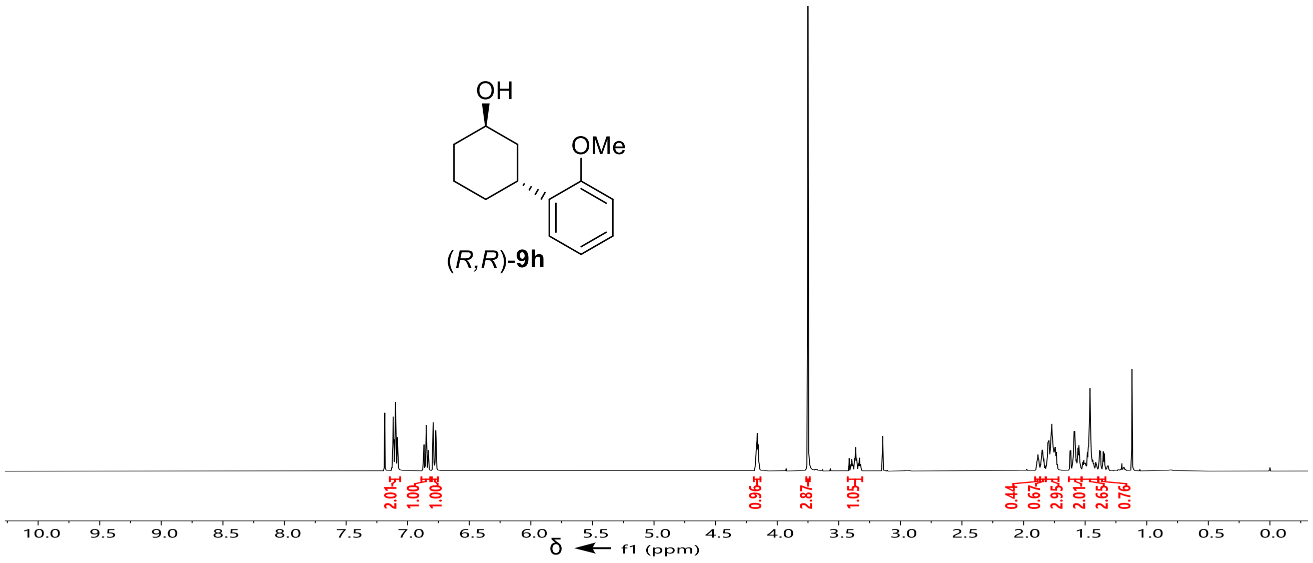

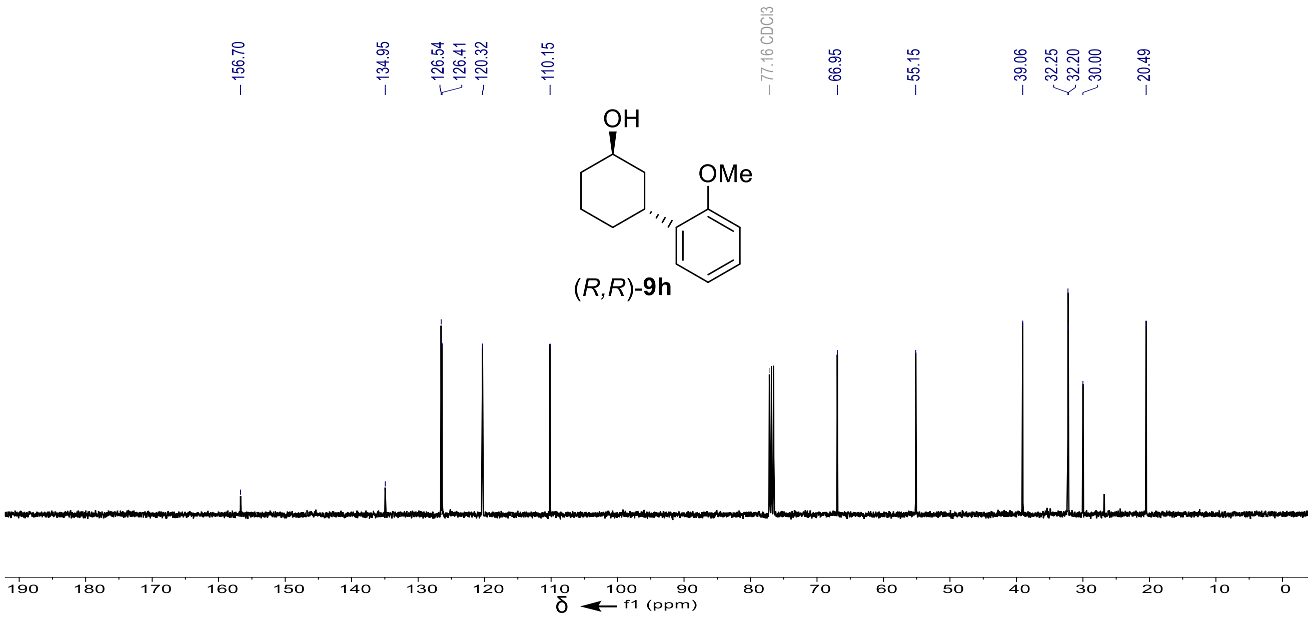

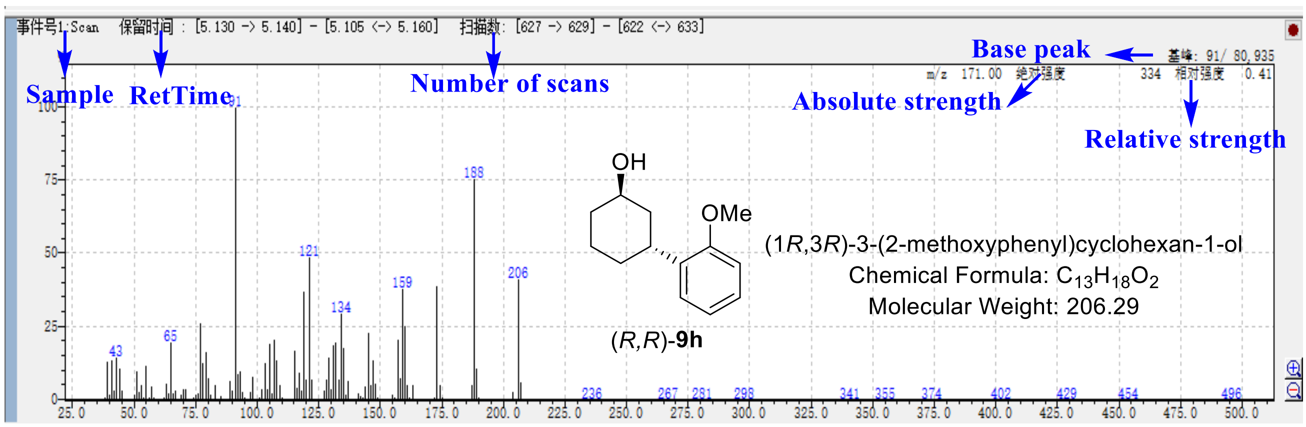

(*R*,*R*)-9i: (1*R*,3*R*)-3-(4-methoxyphenyl)cyclohexan-1-ol

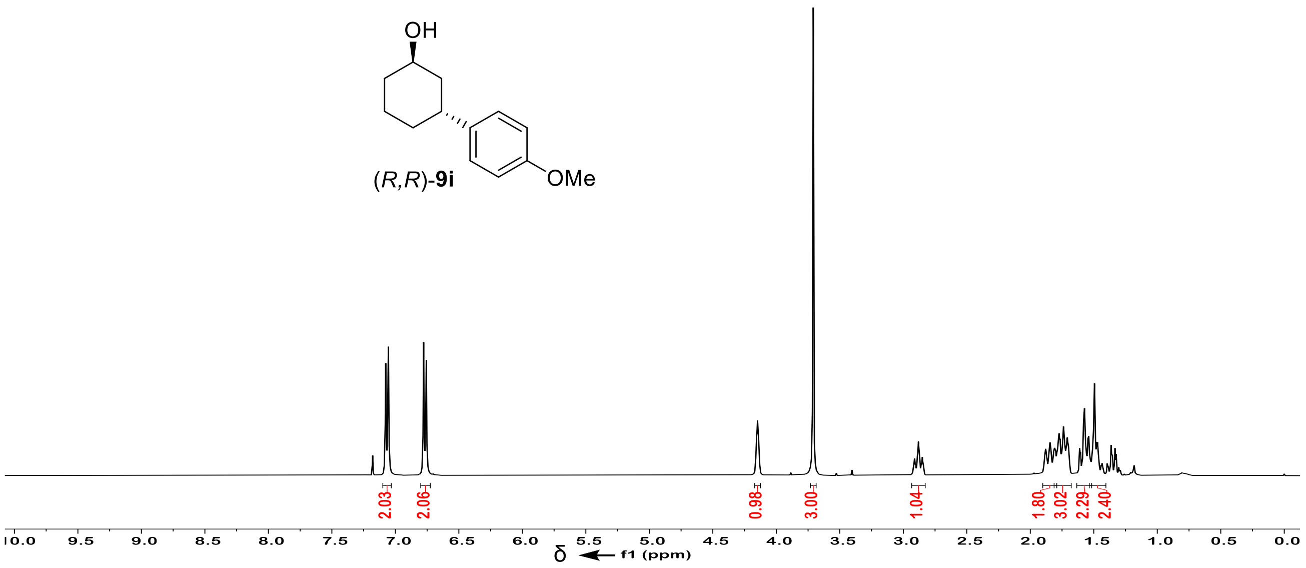

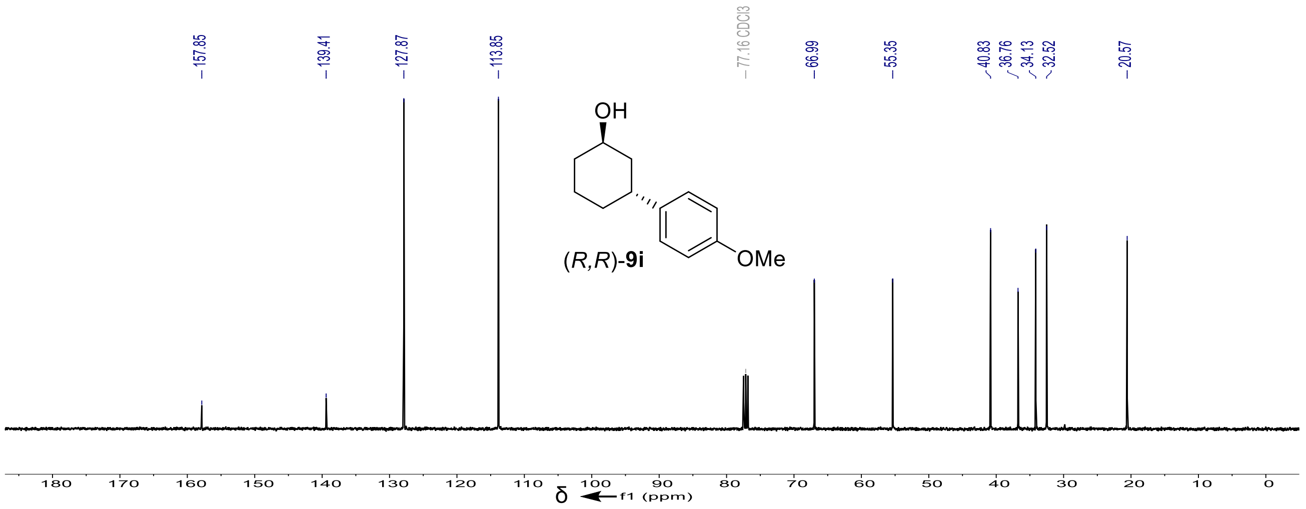

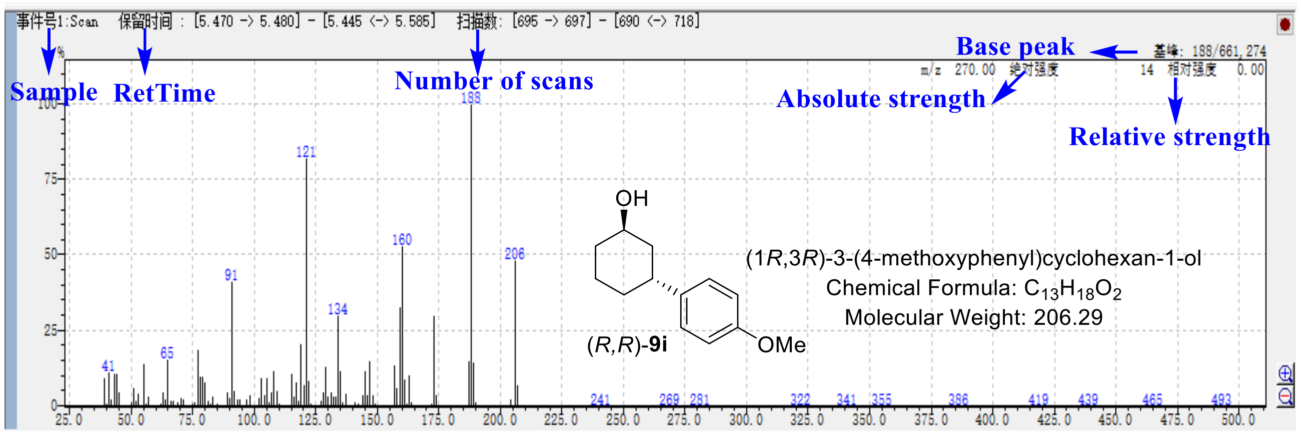

(*R*,*R*)-9j: (1*R*,3*R*)-3-(4-(methylthio)phenyl)cyclohexan-1-ol

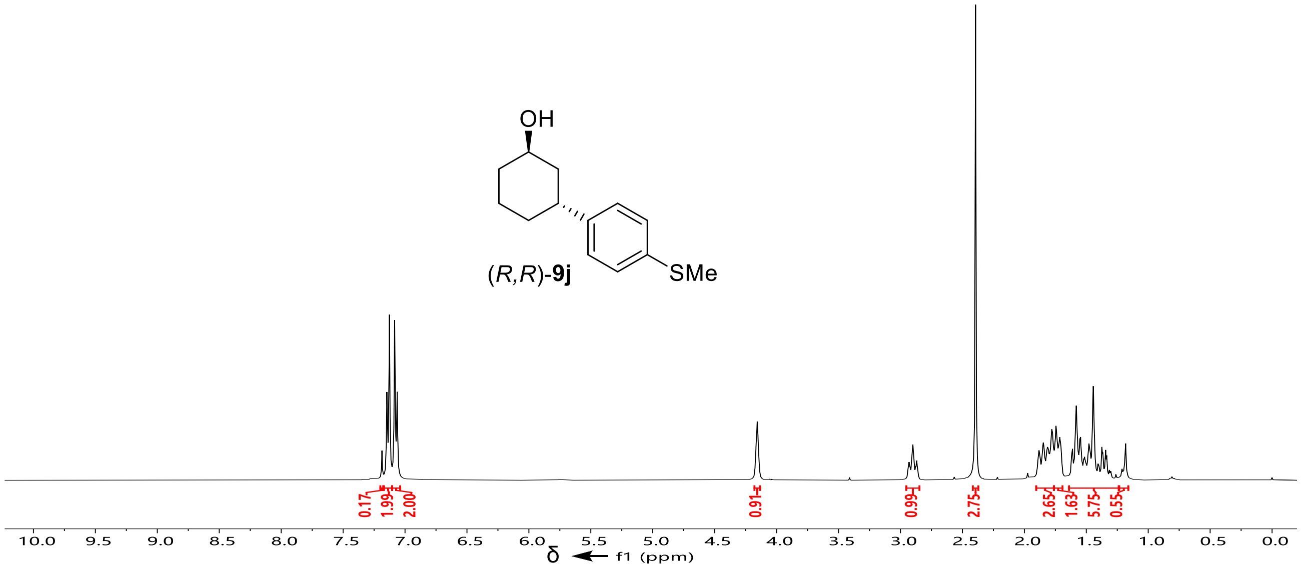

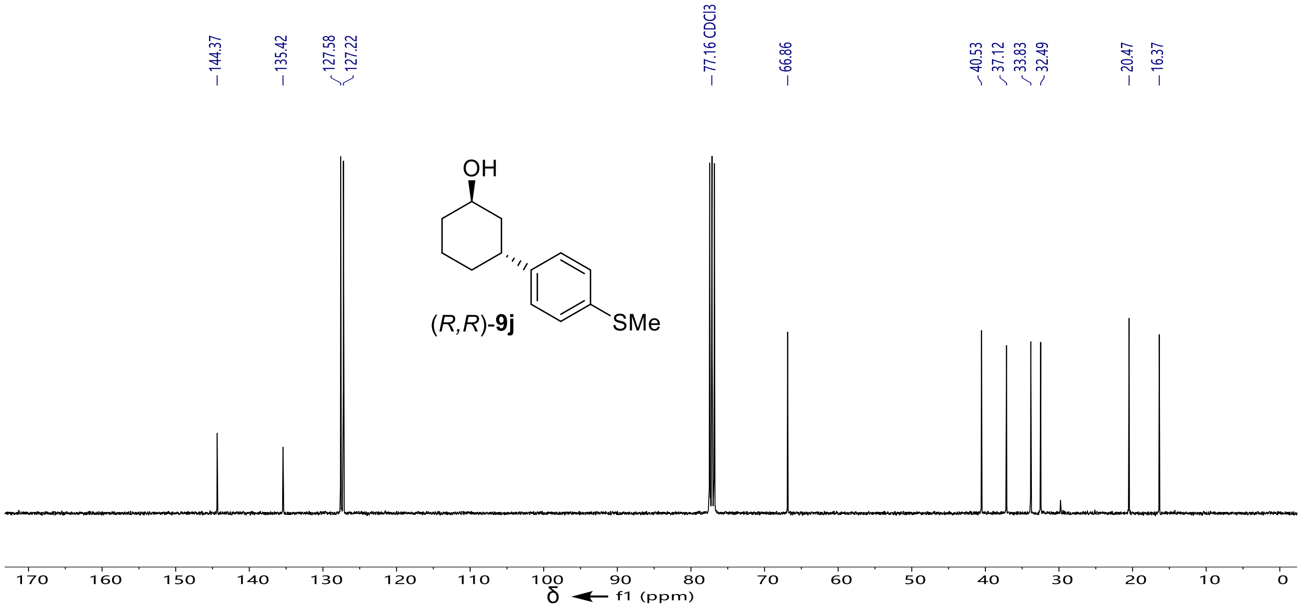

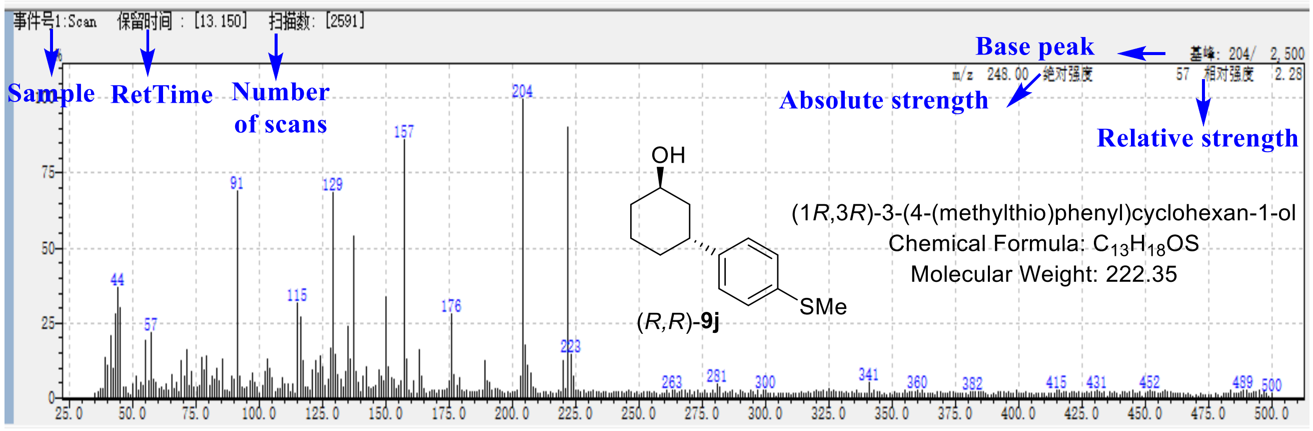

(*R*,*R*)-9k: (1*R*,3*R*)-3-(o-tolyl)cyclohexan-1-ol

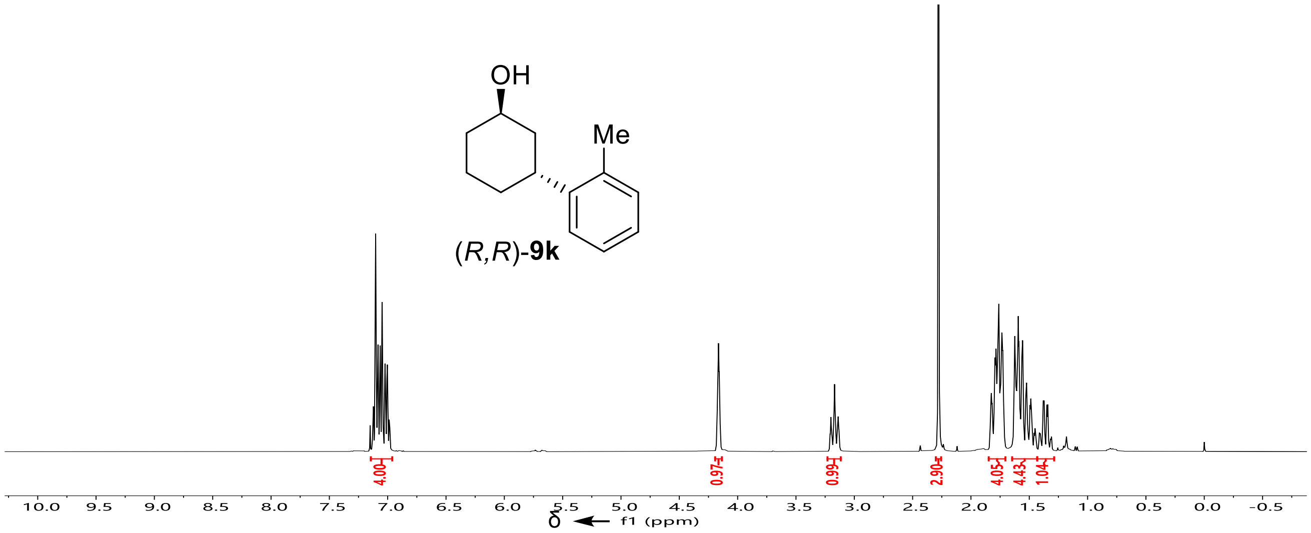

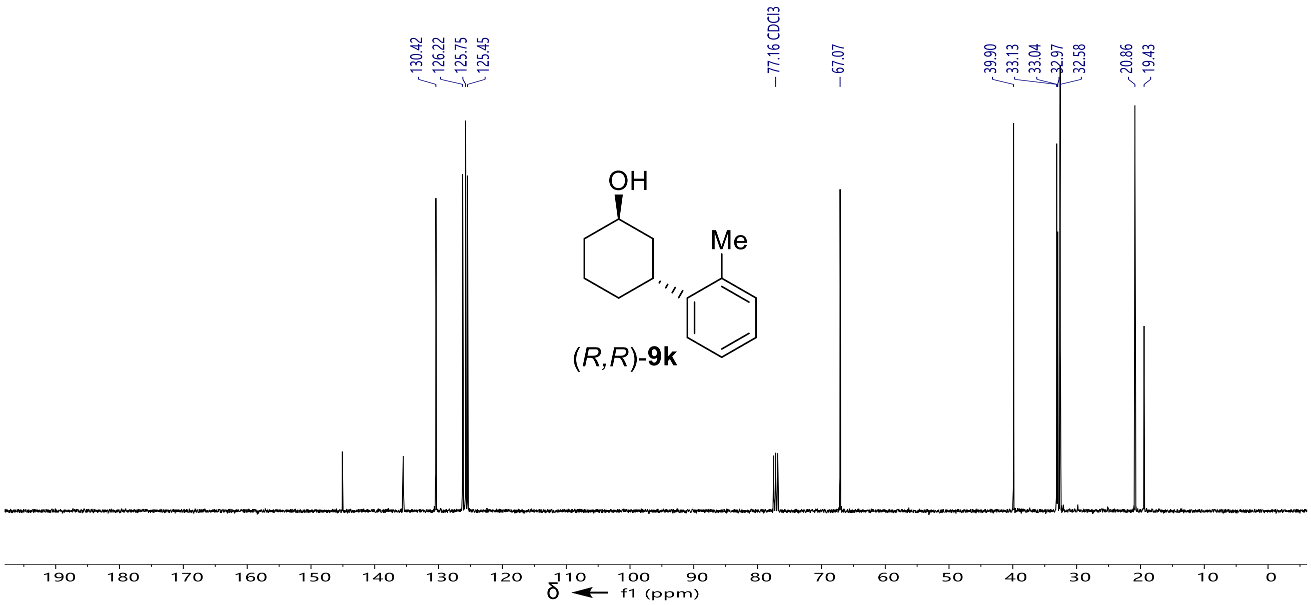

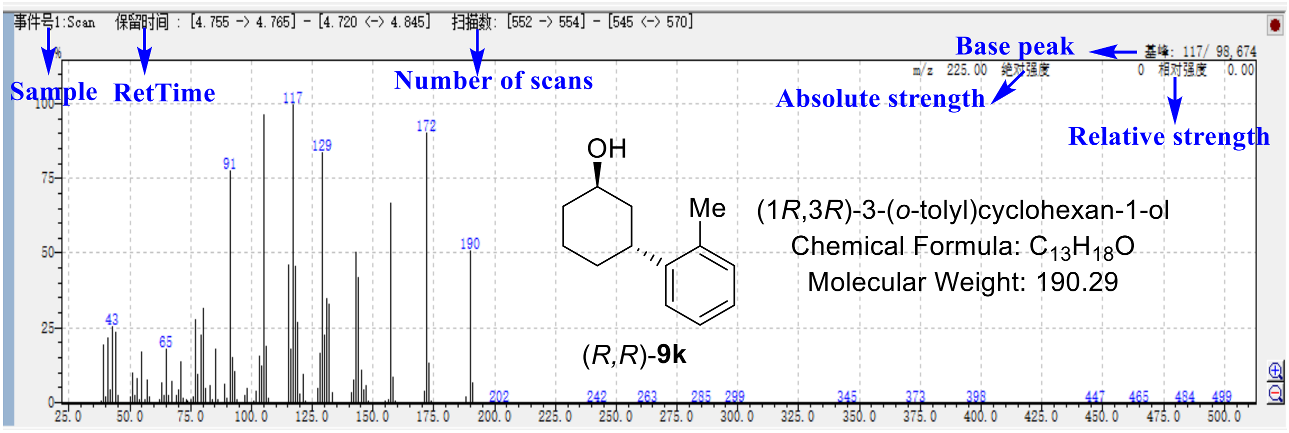

(*R*,*R*)-9l: (1*R*,3*R*)-3-(m-tolyl)cyclohexan-1-ol

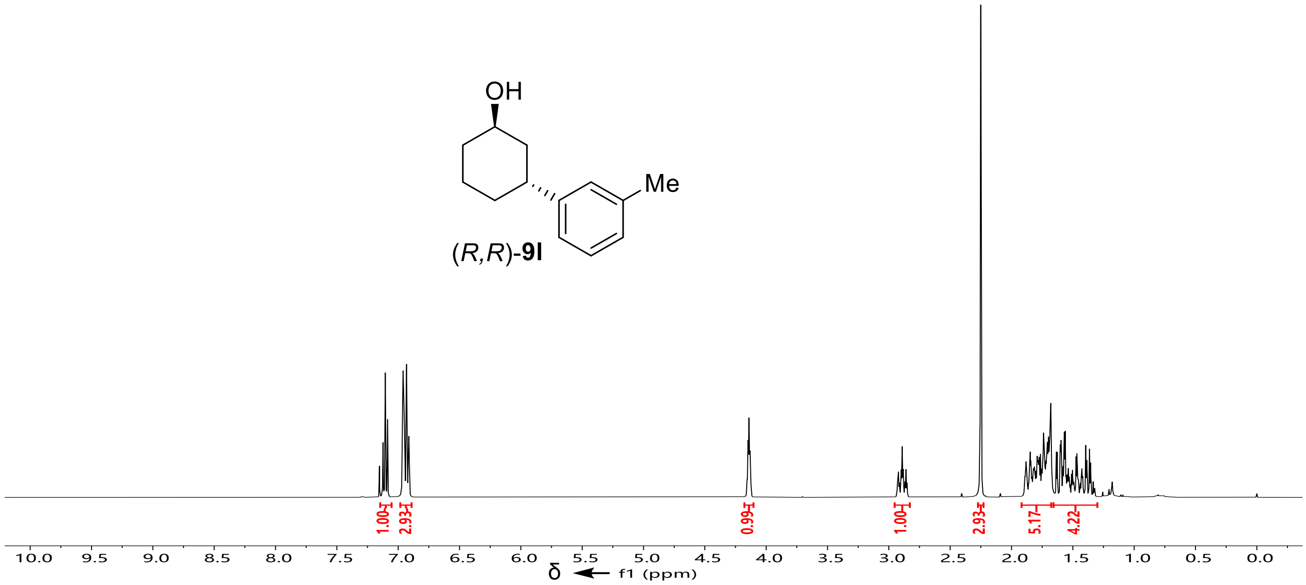

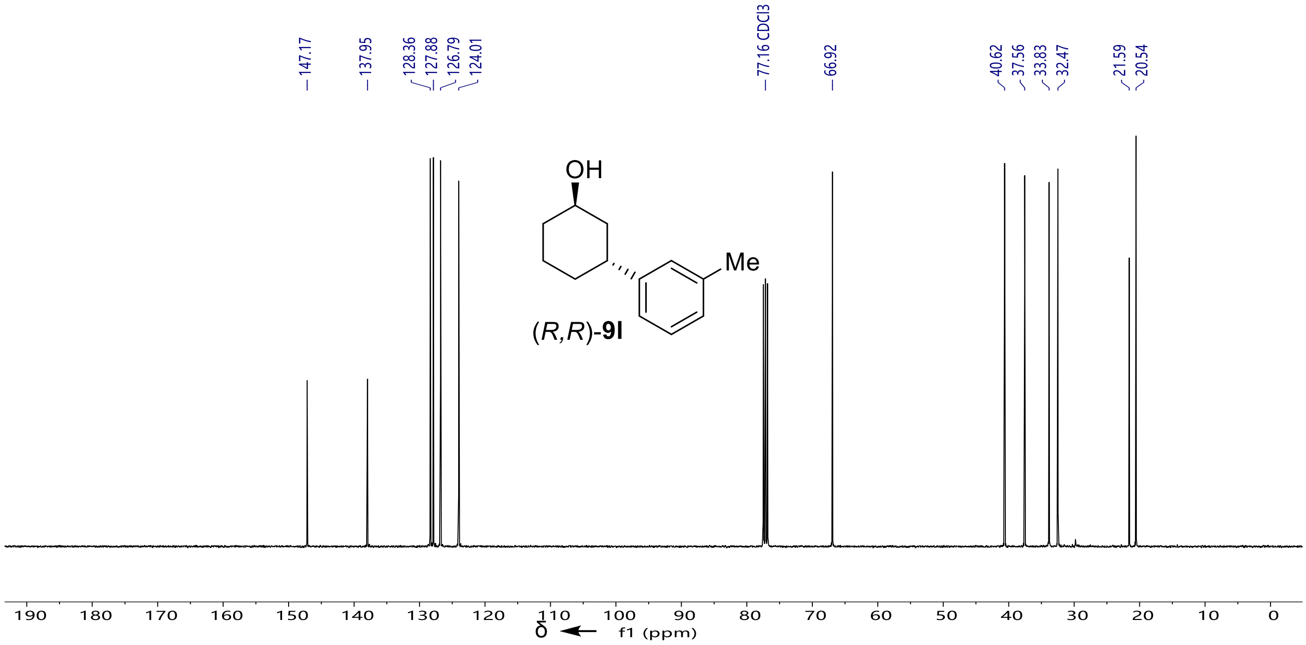

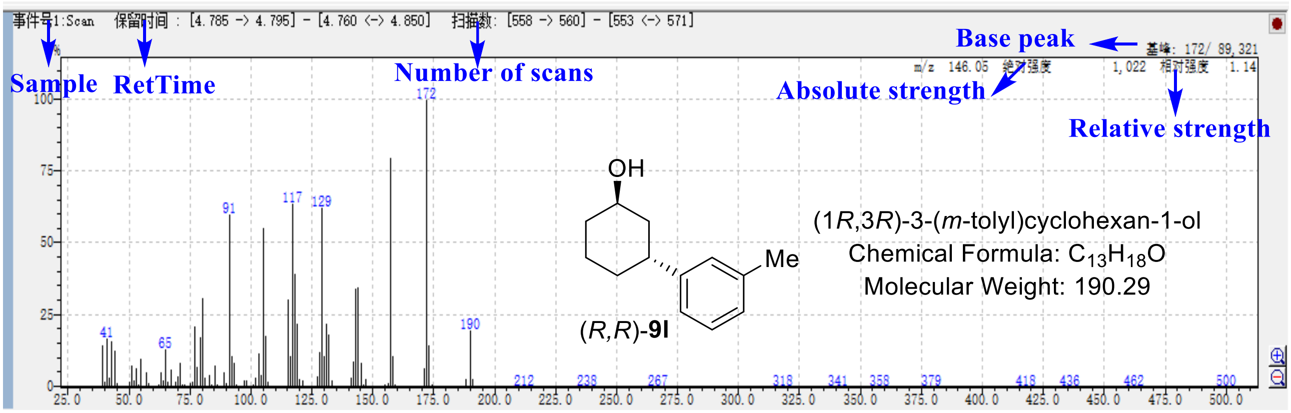

(*R*,*R*)-9m: (1*R*,3*R*)-3-(p-tolyl)cyclohexan-1-ol

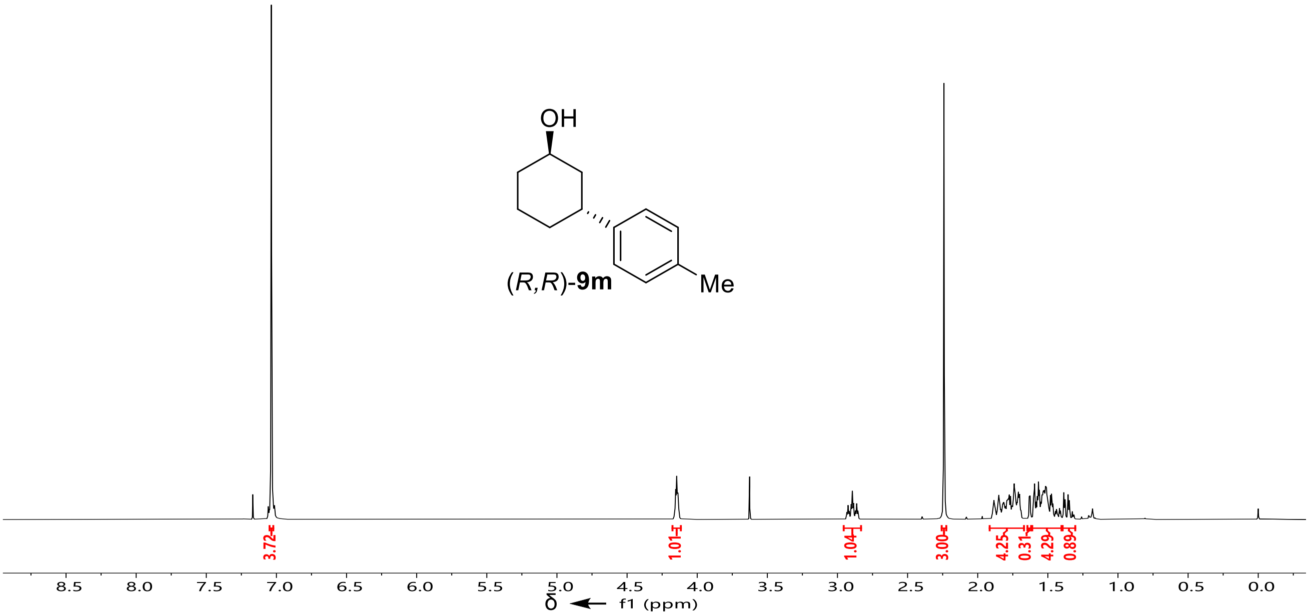

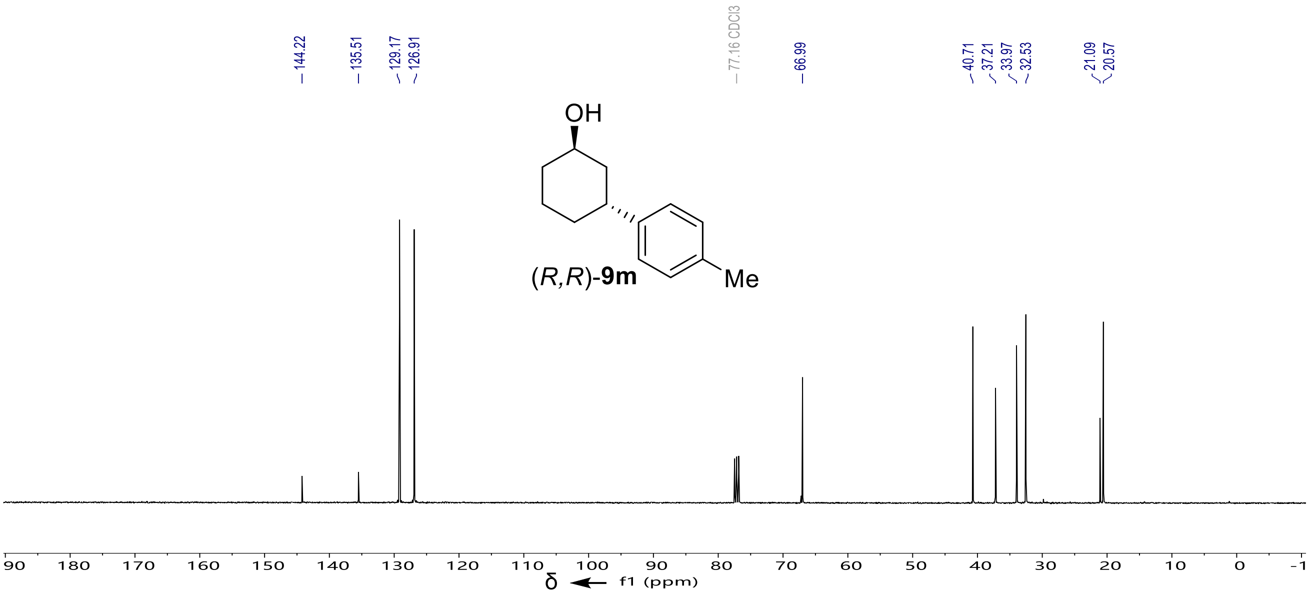

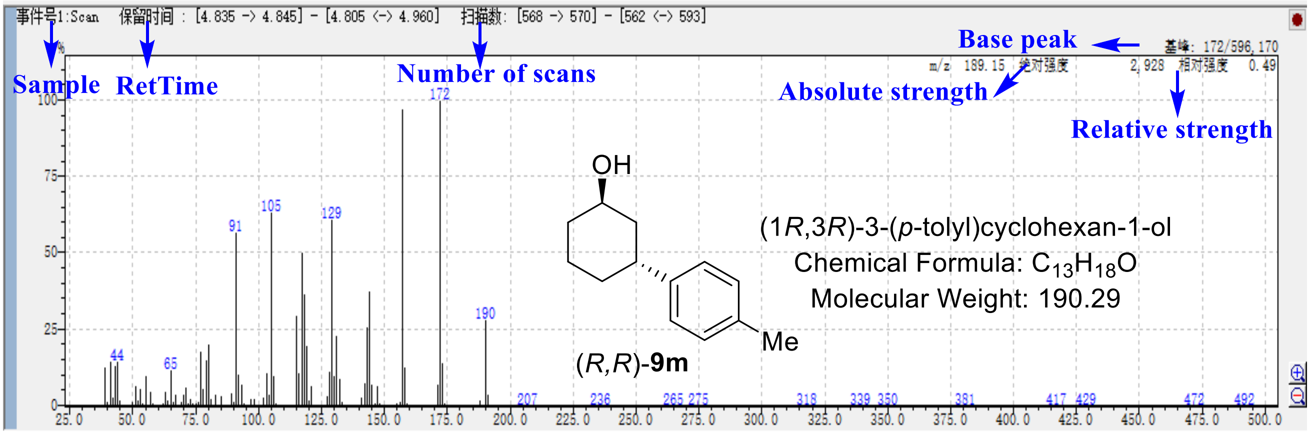

(*R*,*R*)-9n: (1*R*,3*R*)-3-(4-butylphenyl)cyclohexan-1-ol

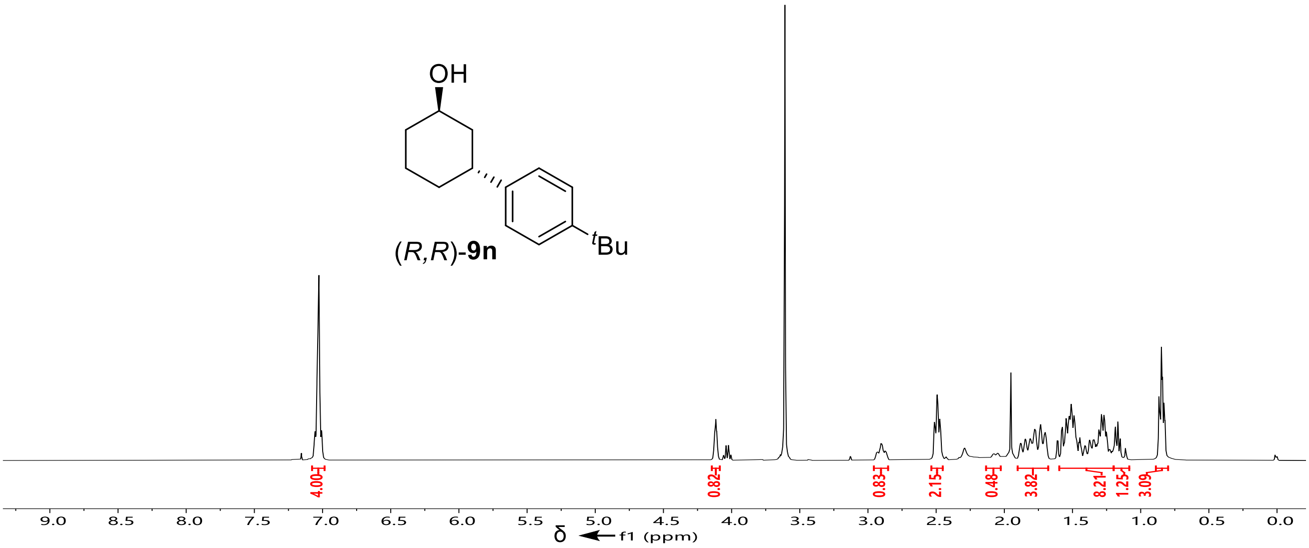

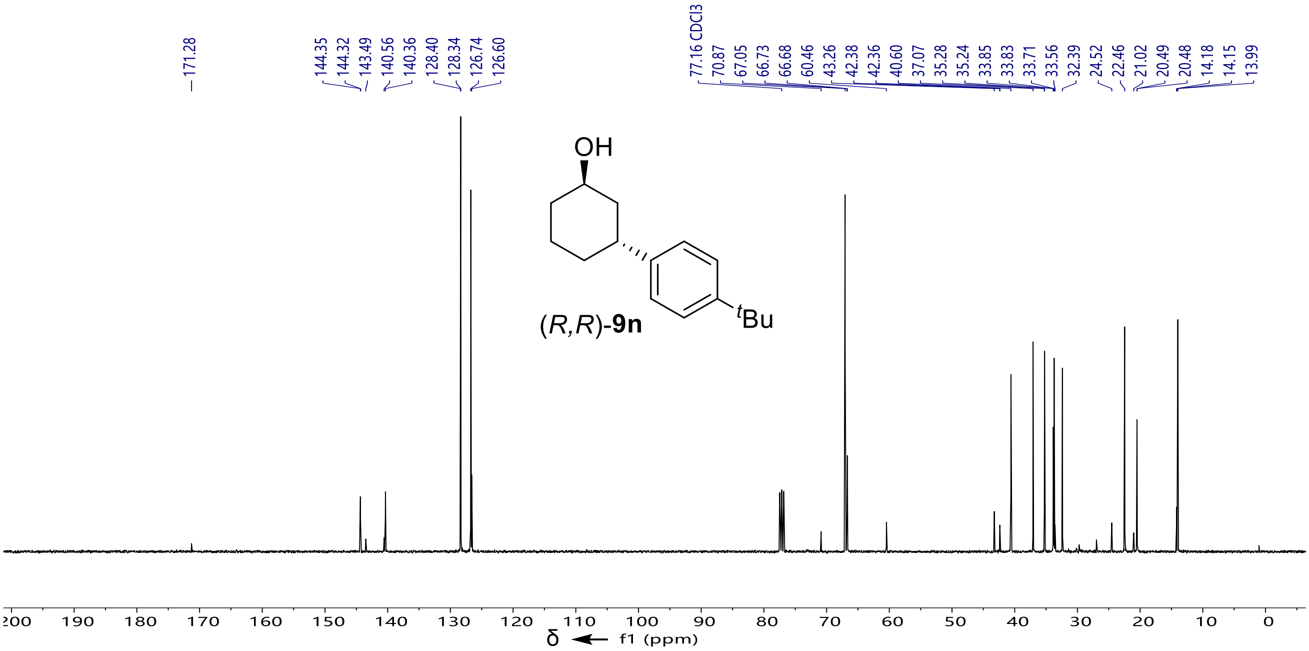

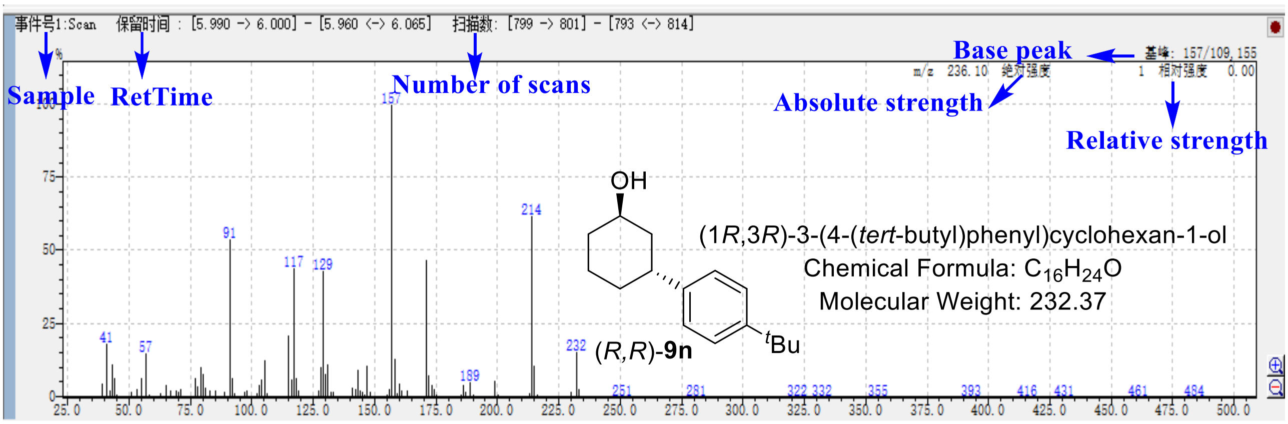

(*R*,*R*)-9o: (1*R*,3*R*)-3-(thiophen-3-yl)cyclohexan-1-ol

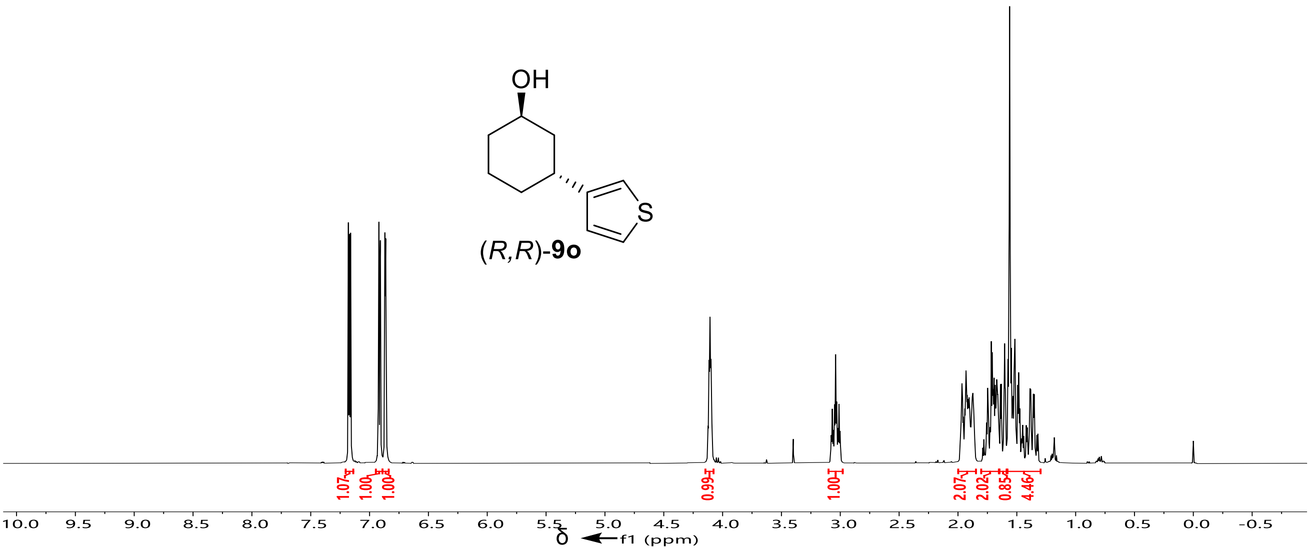

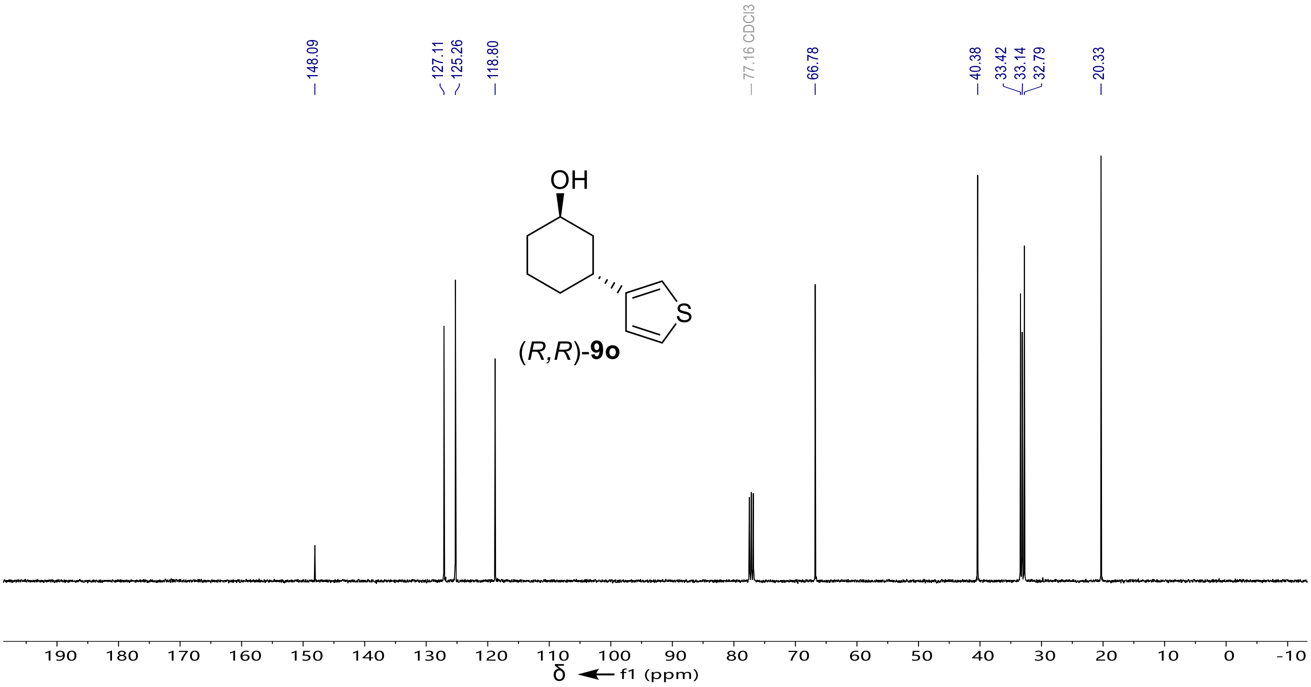

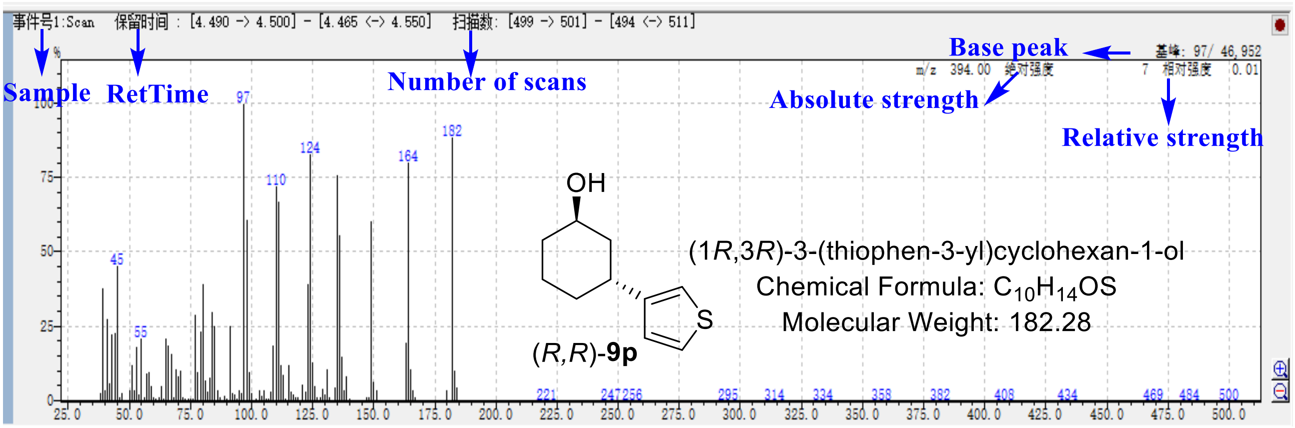

(***R***,***R***)**-9p:** ***tert*****-butyl 3-((1*****R*****,3*****R*****)-3-hydroxycyclohexyl)-1H-indole-1-carboxylate**

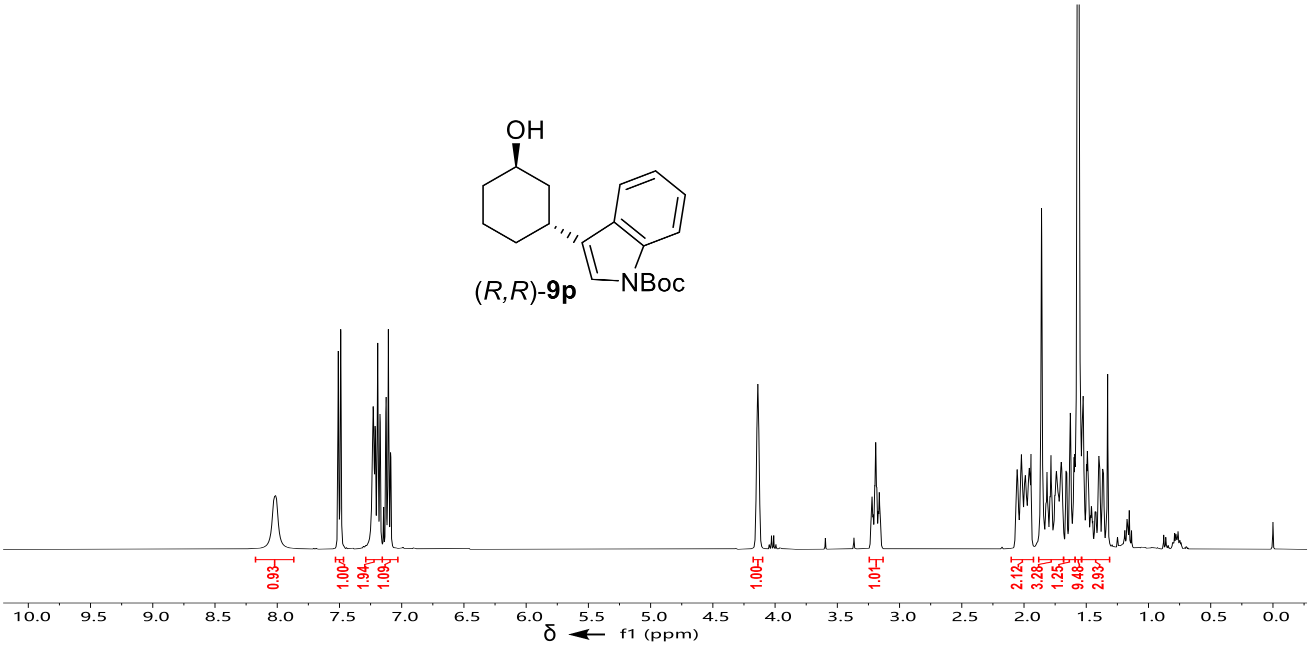

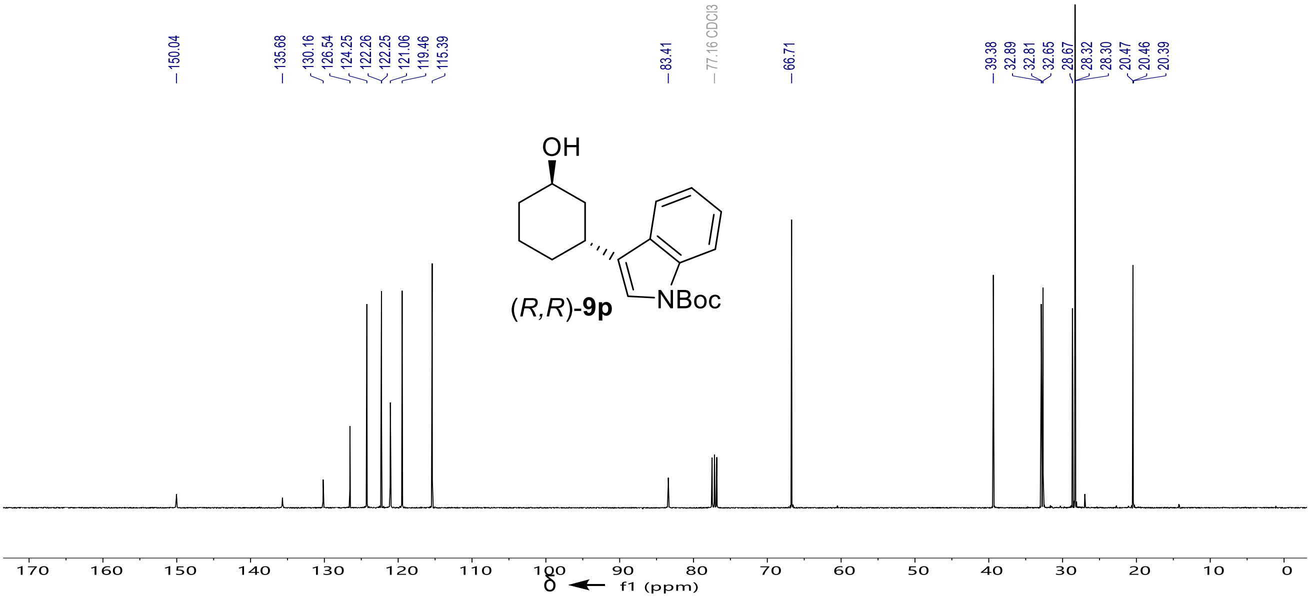

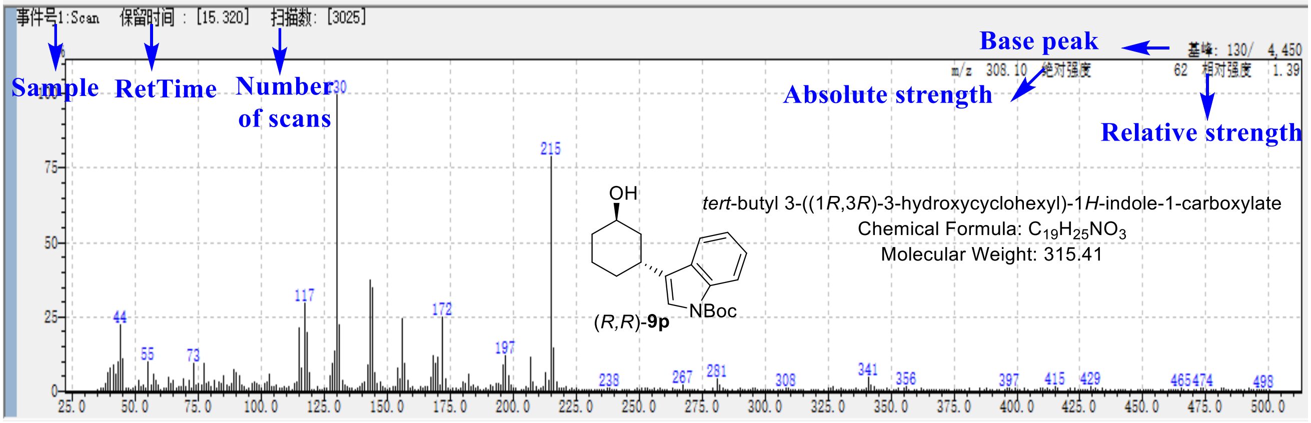

(*R*,*R*)-9q: (1*R*,3*R*)-3-([1,1'-biphenyl]-4-yl)cyclohexan-1-ol

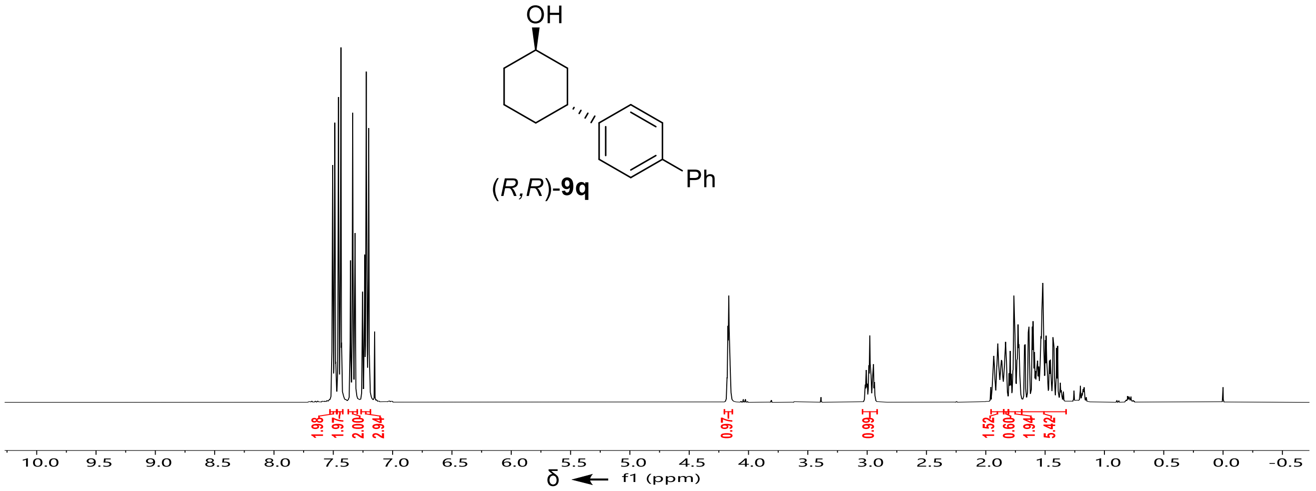

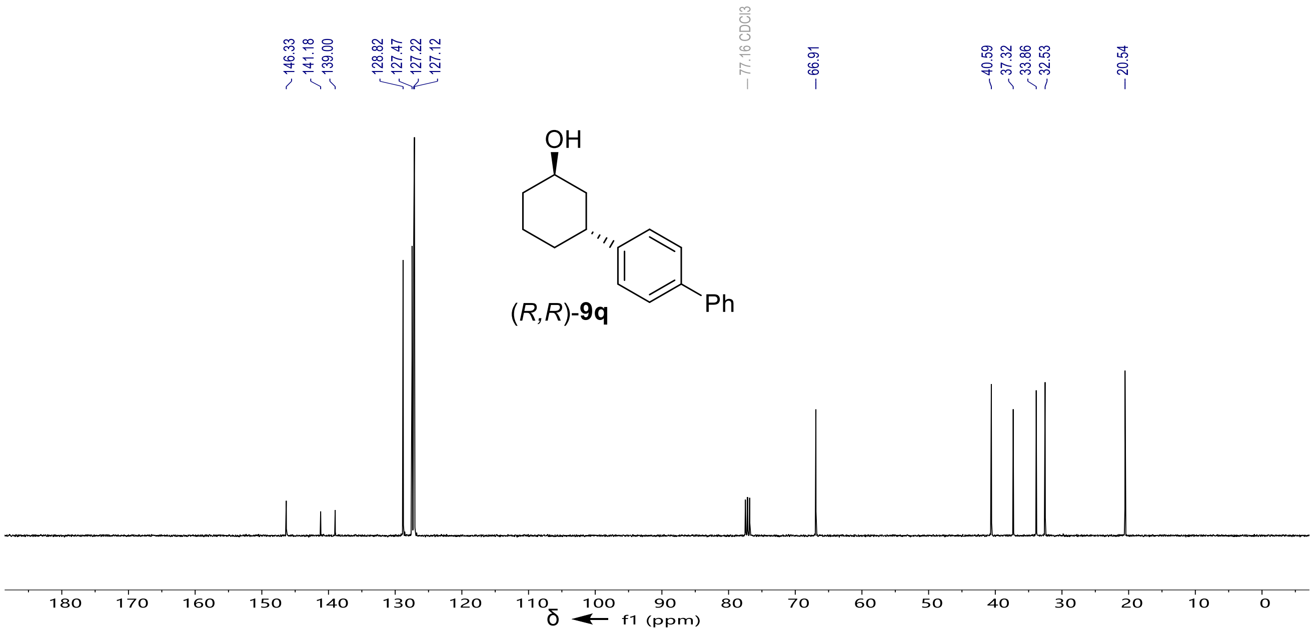

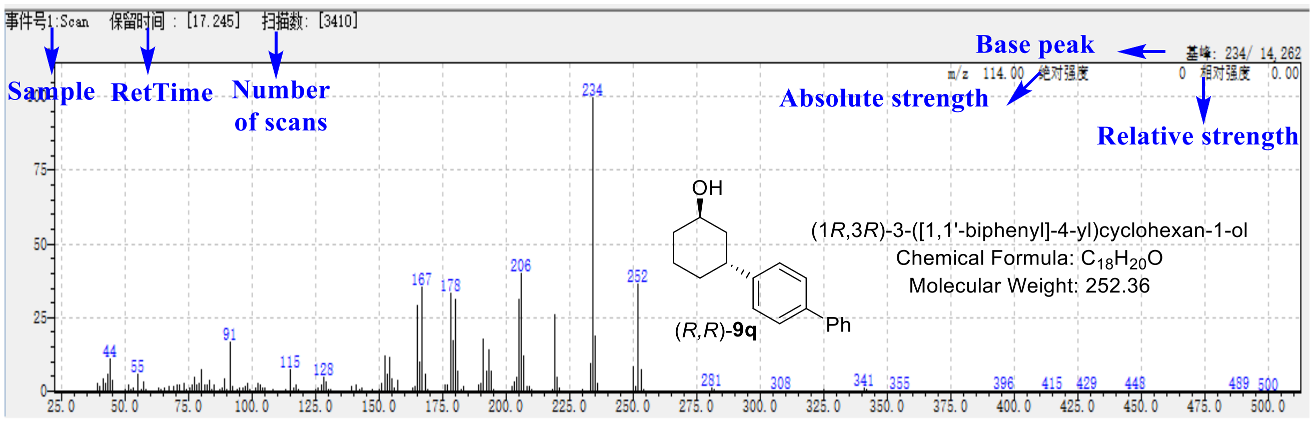

(*R*,*R*)-9r: (1*R*,3*R*)-3-(naphthalen-1-yl)cyclohexan-1-ol

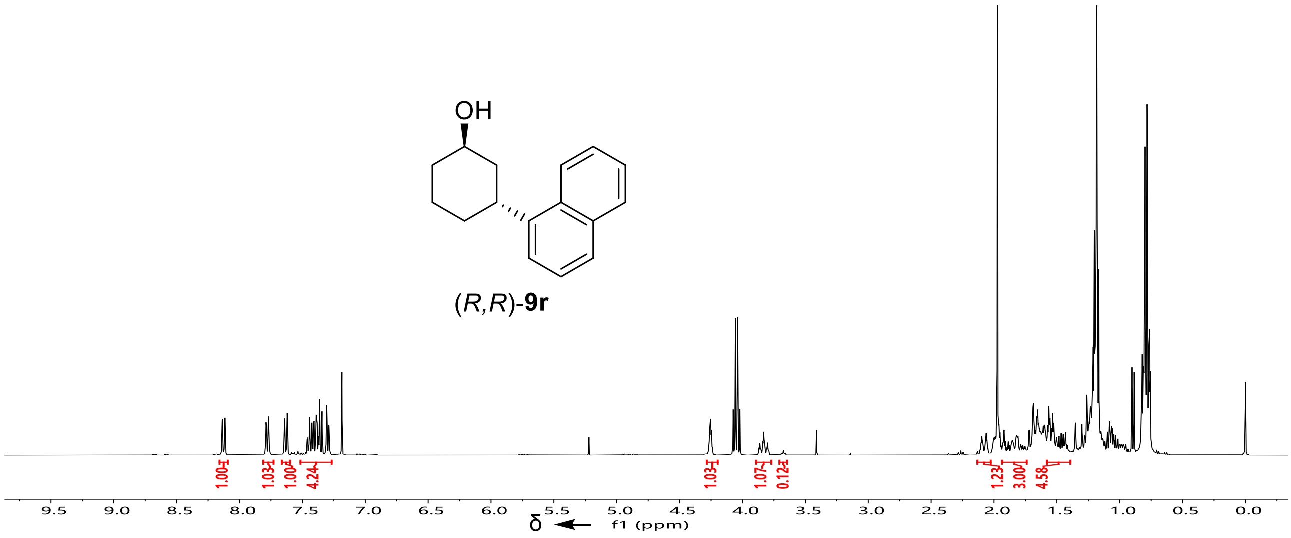

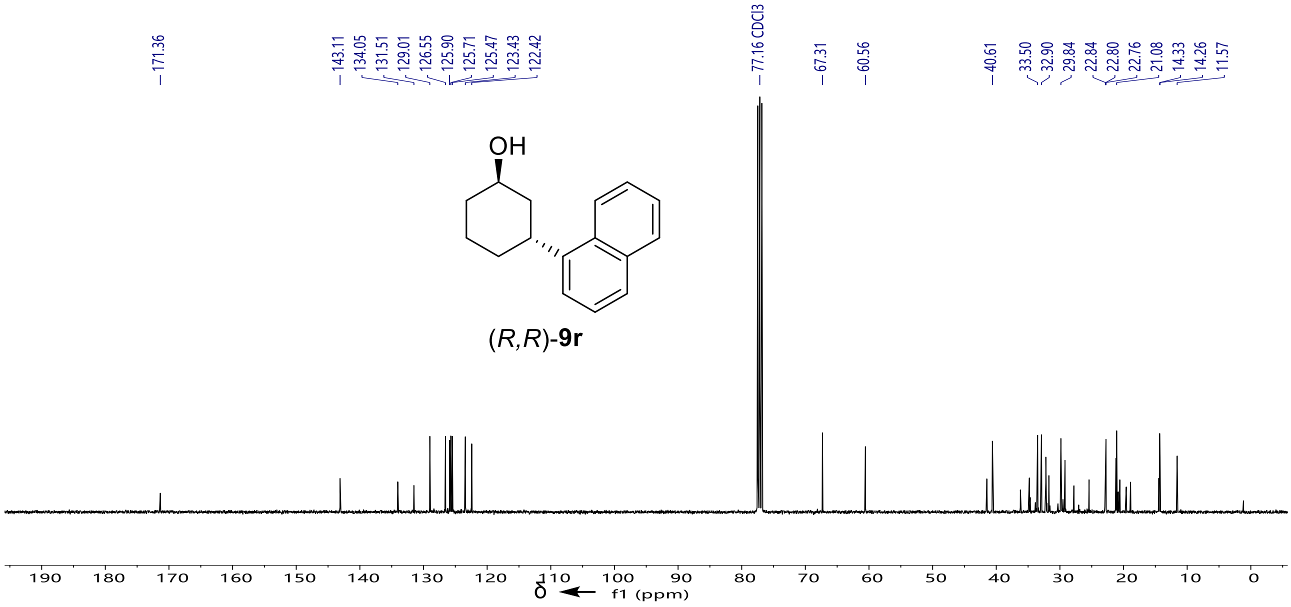

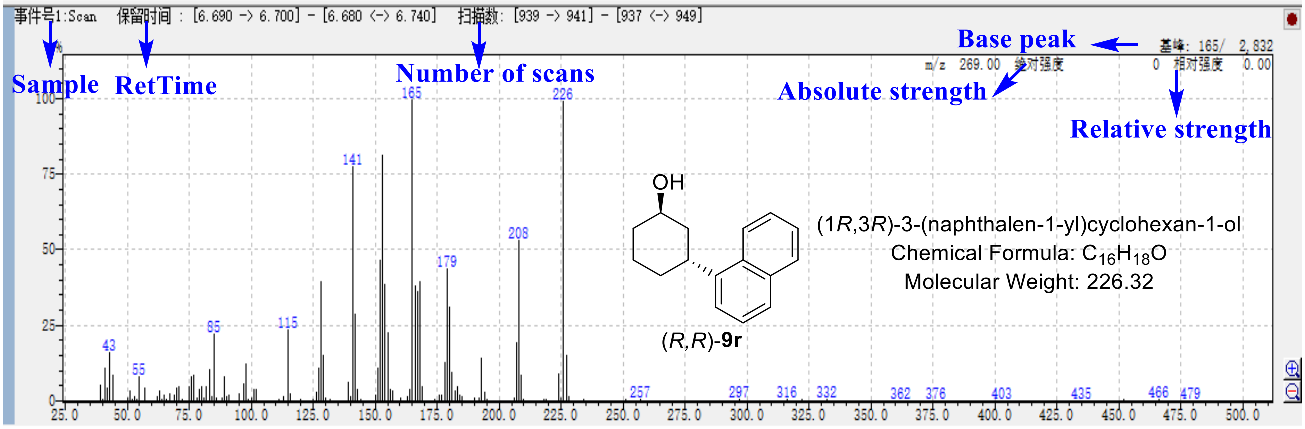

(*R*,*R*)-9s: (1*R*,3*R*)-3-(phenanthren-9-yl)cyclohexan-1-ol

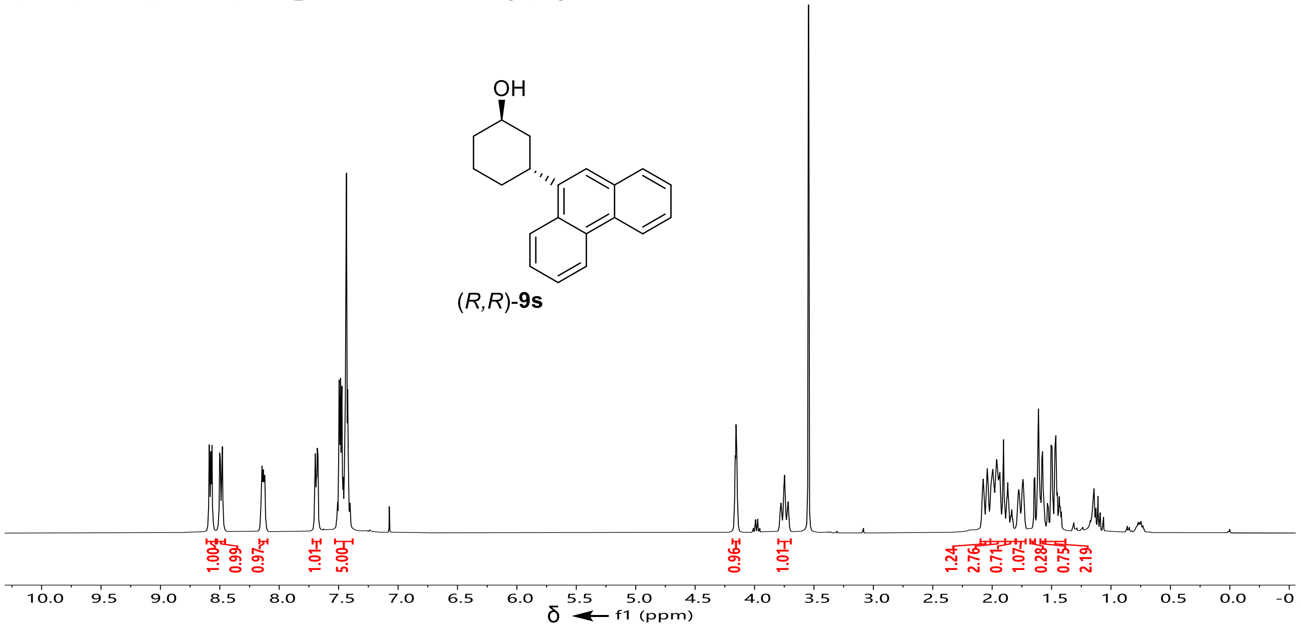

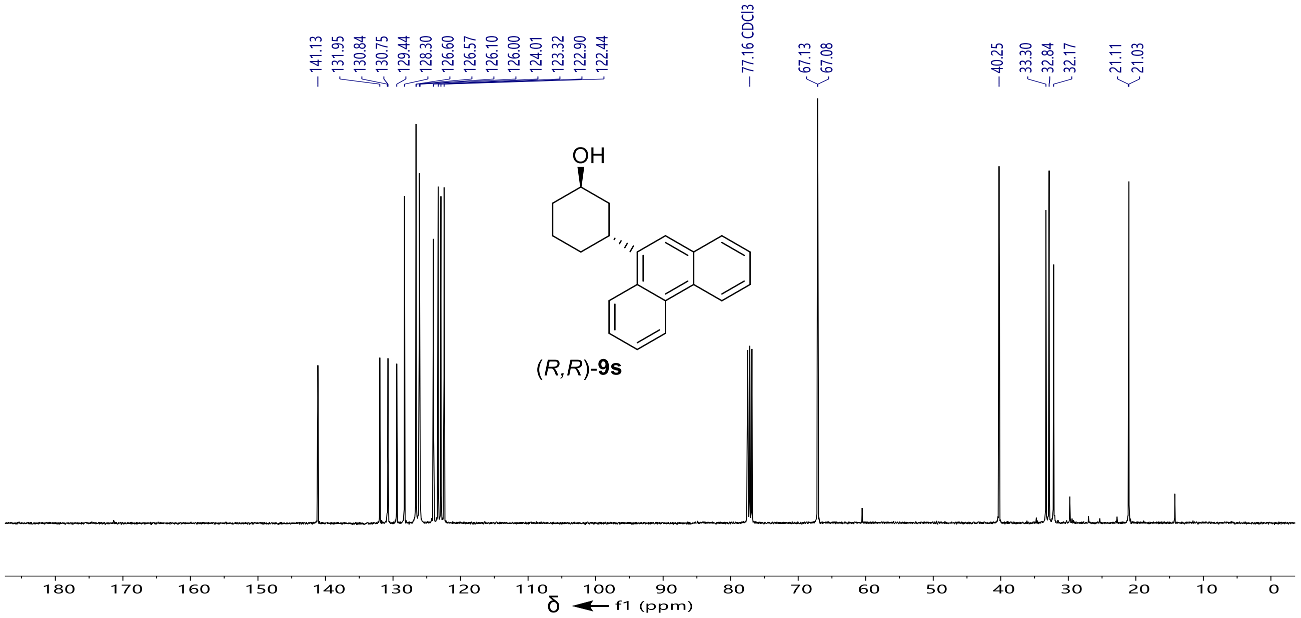

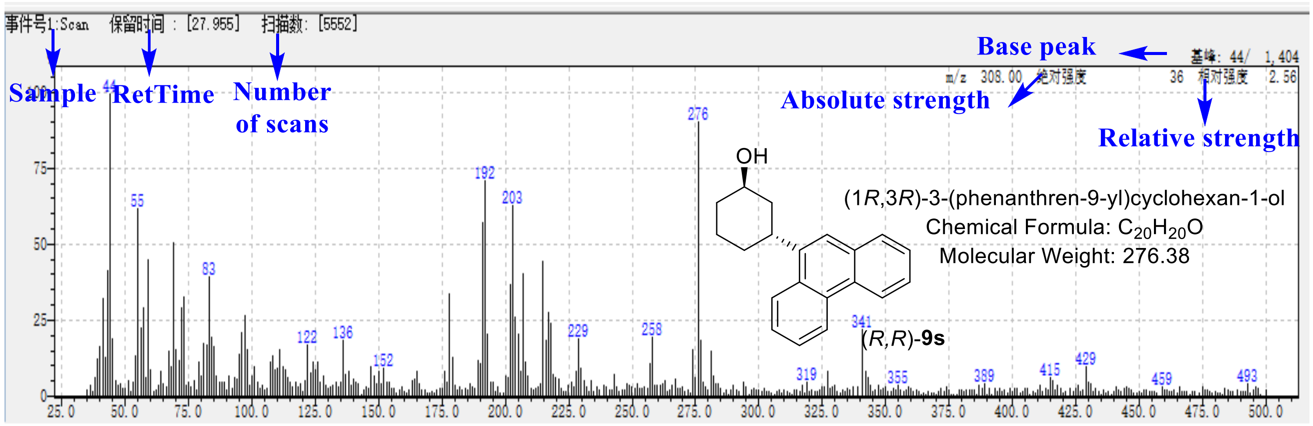

(*R*,*R*)-9t: *tert*-butyl (2*R*,4*R*)-4-hydroxy-2-phenylpiperidine-1-carboxylate

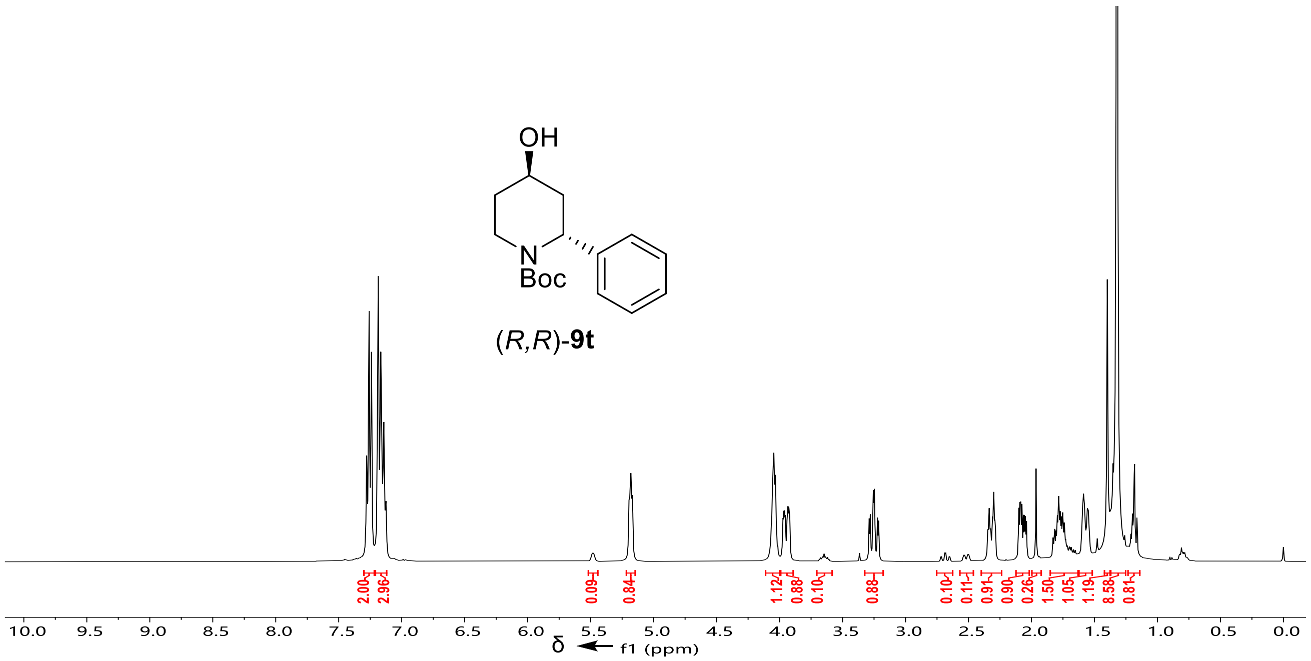

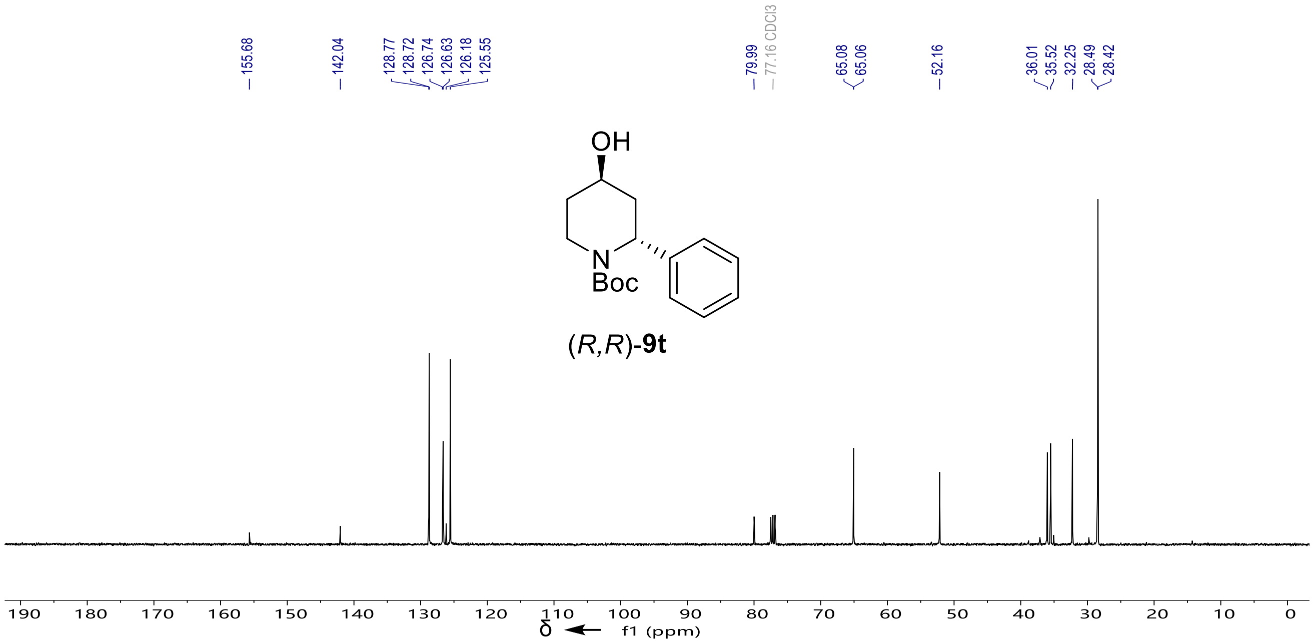

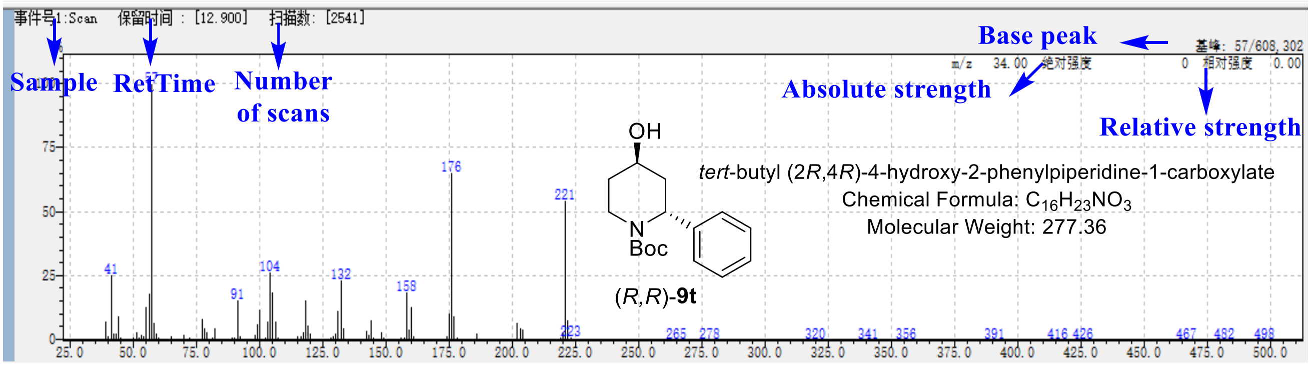

(*R*,*R*)-9t: (1*R*,3*R*)-3-phenylcyclopentan-1-ol

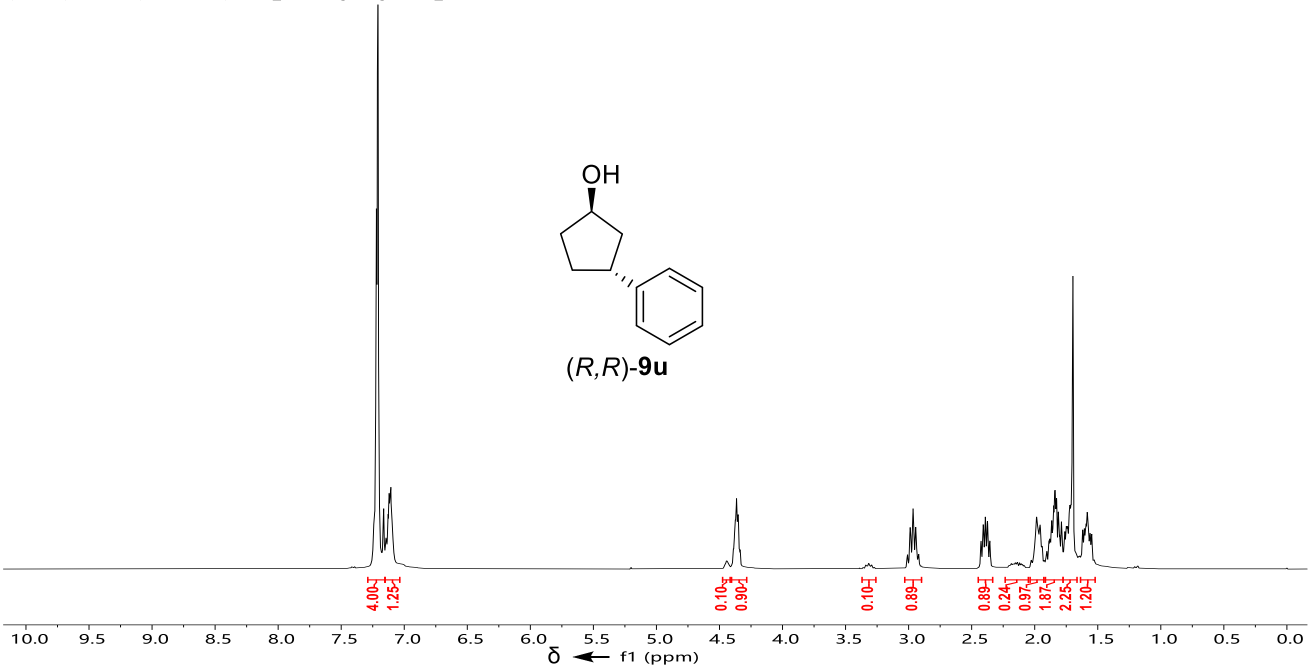

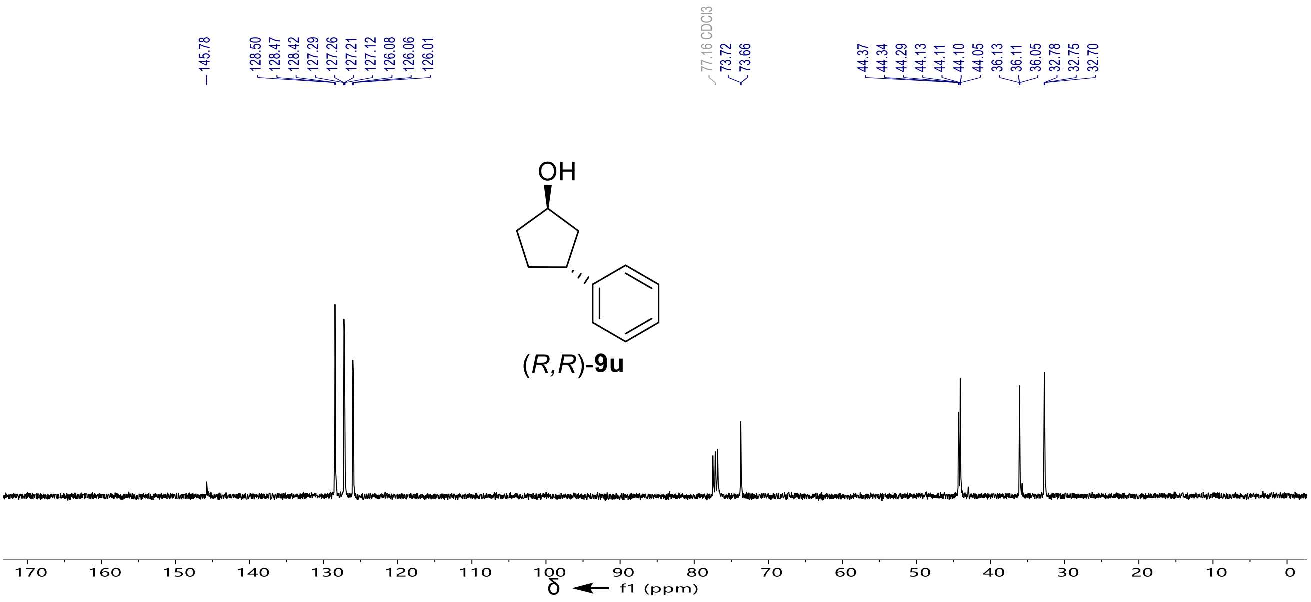

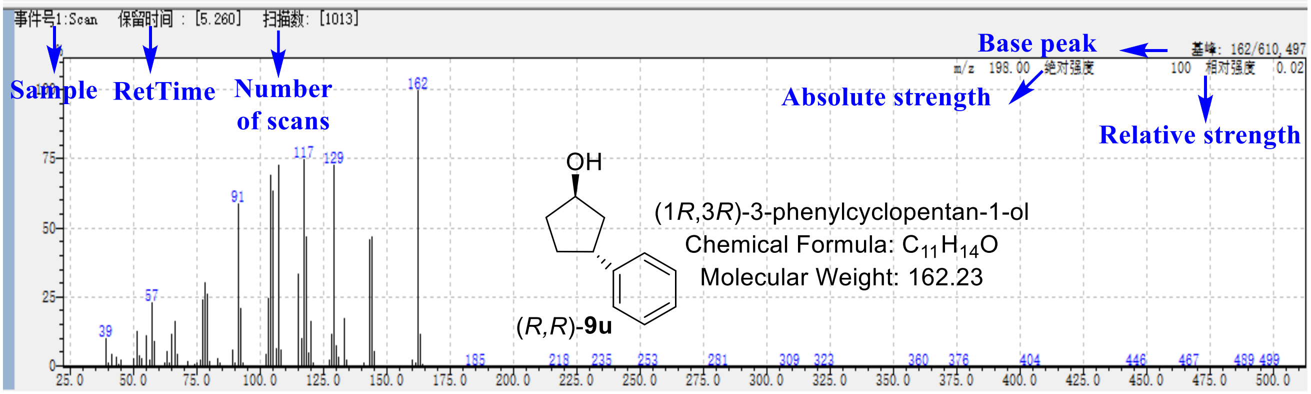

(*R*,*R*)-9v: (1*R*,3*R*)-3-phenylcycloheptan-1-ol

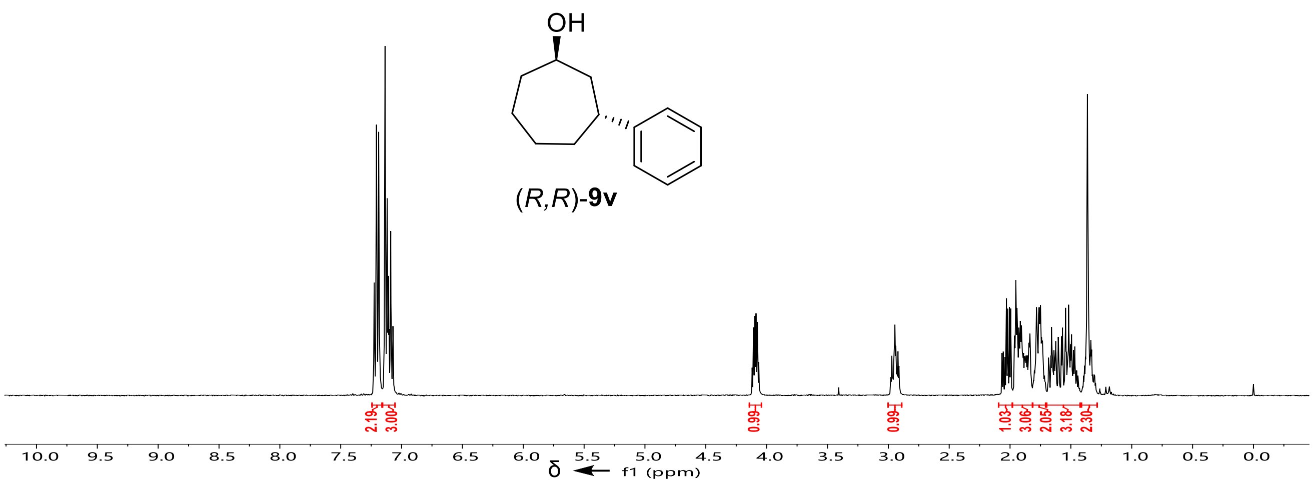

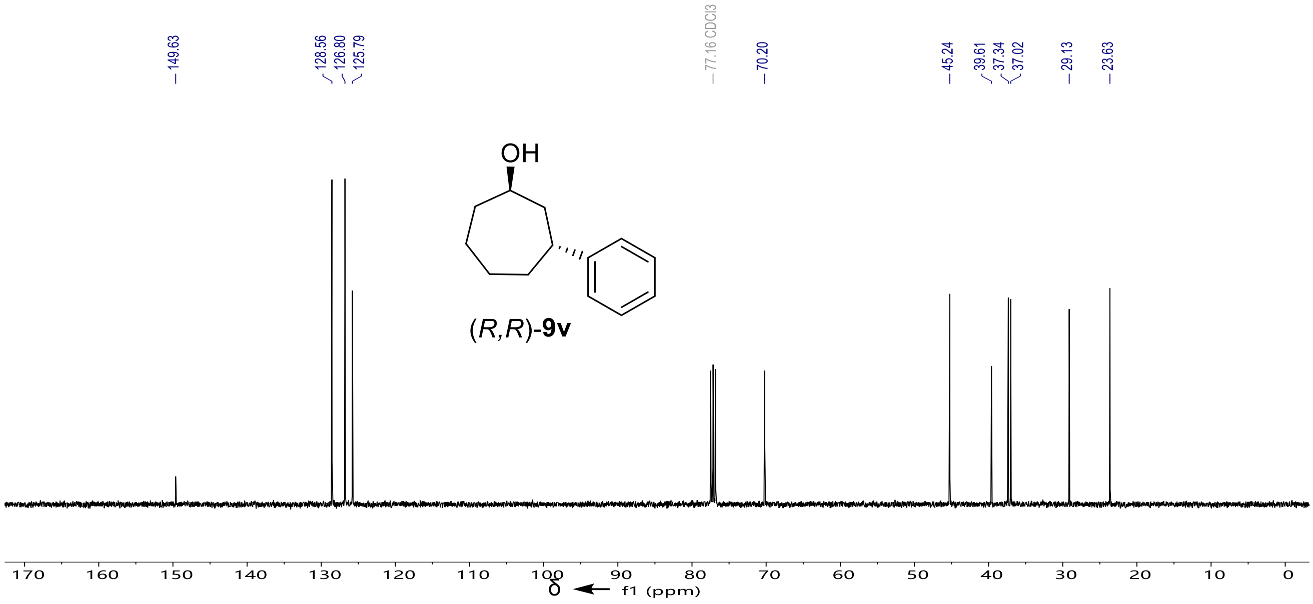

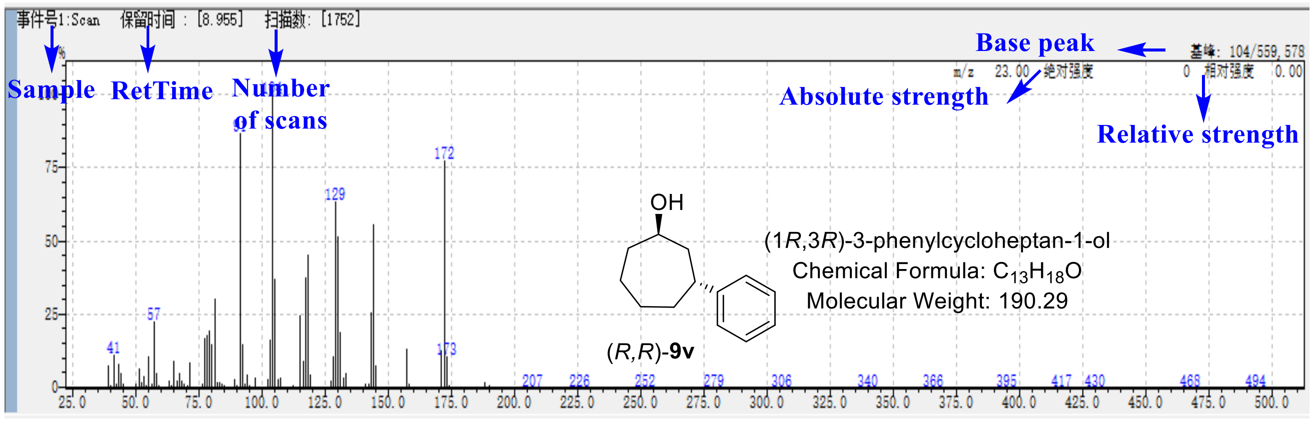

(*R*,*R*)-9w: (1*R*,3*R*)-3-(m-tolyl)cycloheptan-1-ol

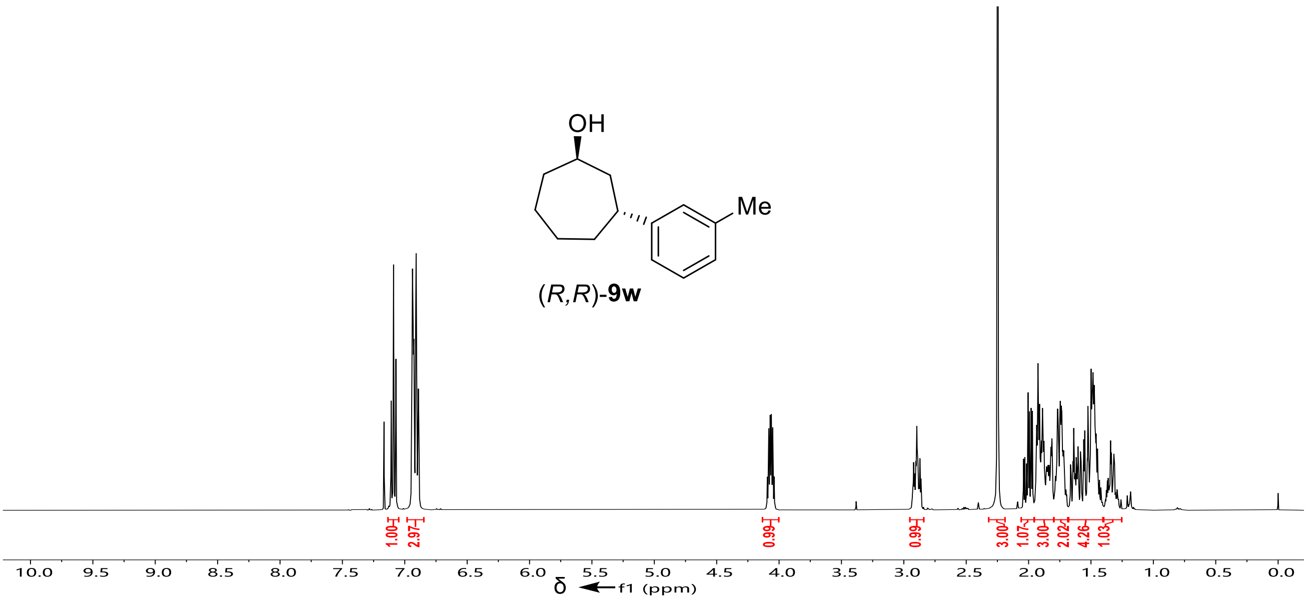

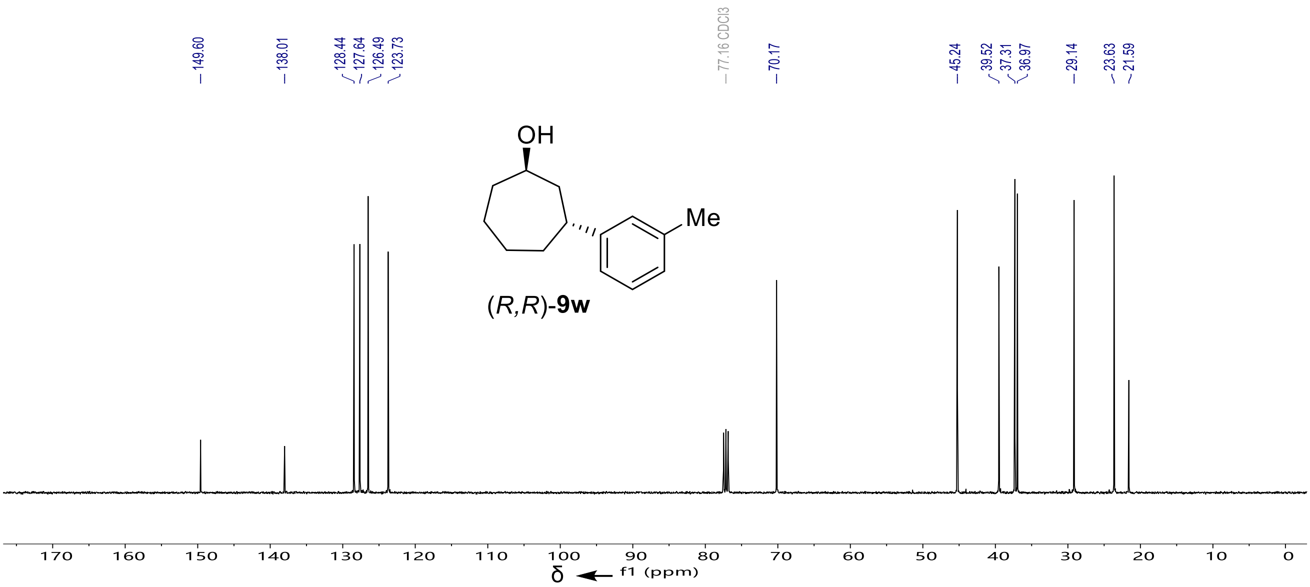

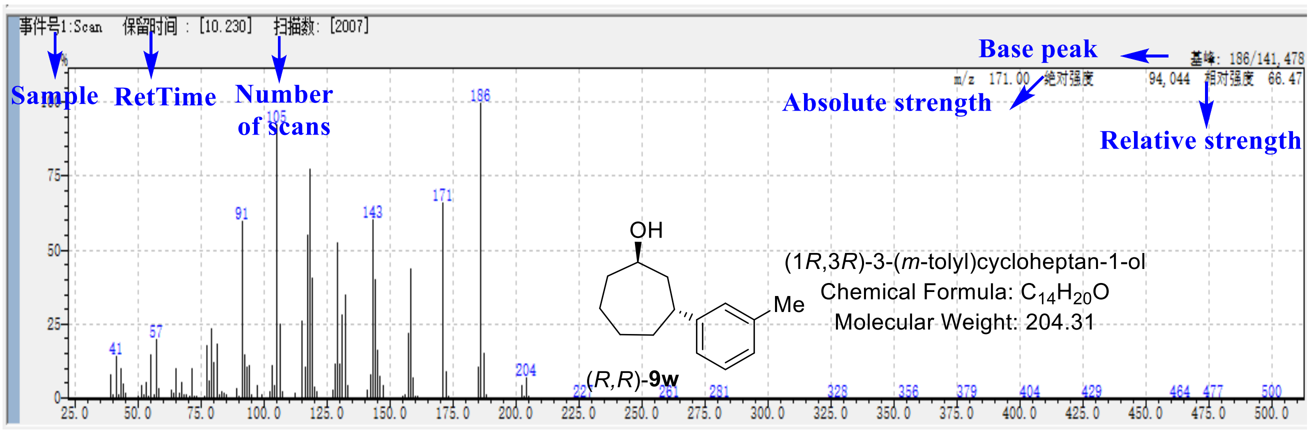

(*R*,*R*)-9x: (1*R*,3*R*)-3-(p-tolyl)cycloheptan-1-ol

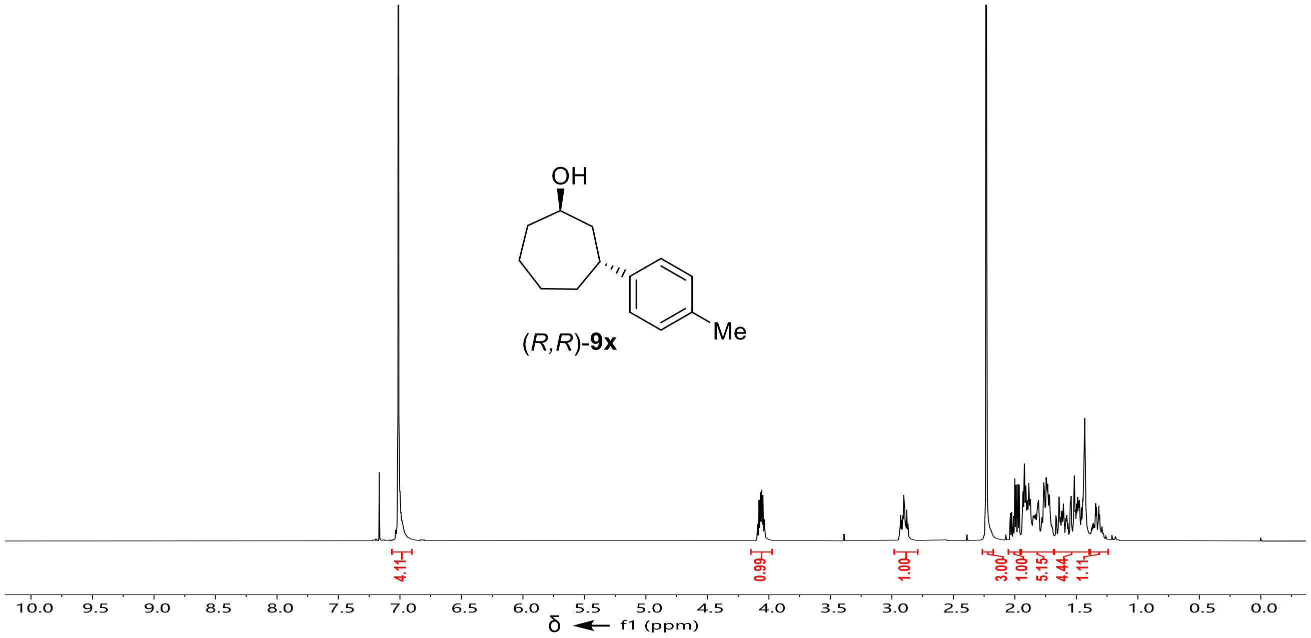

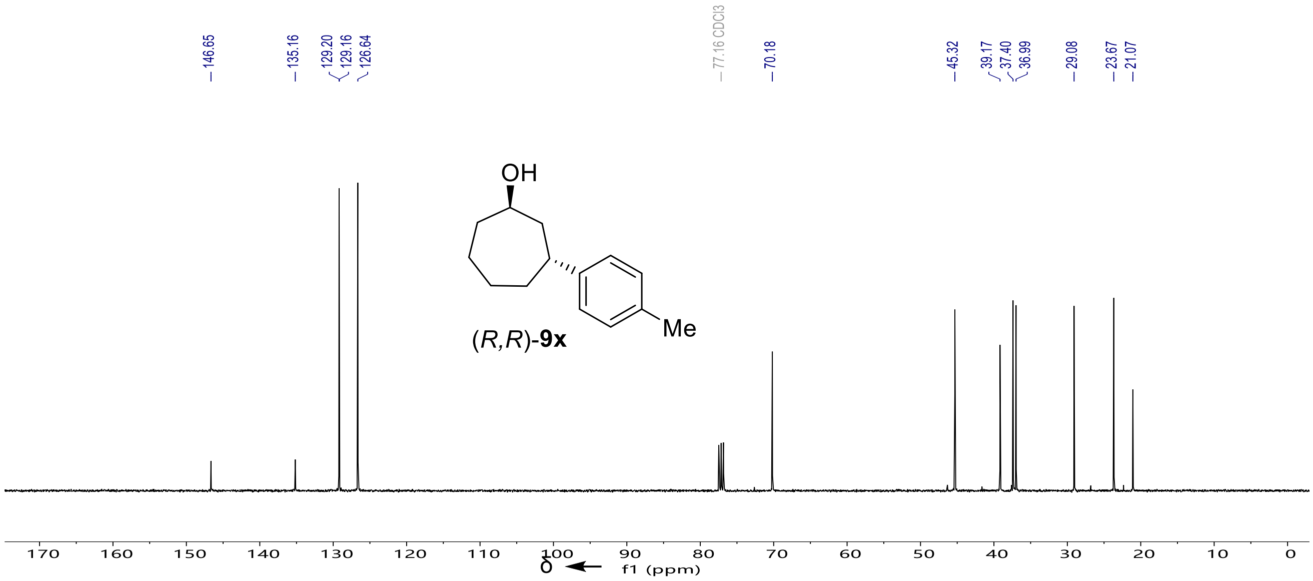

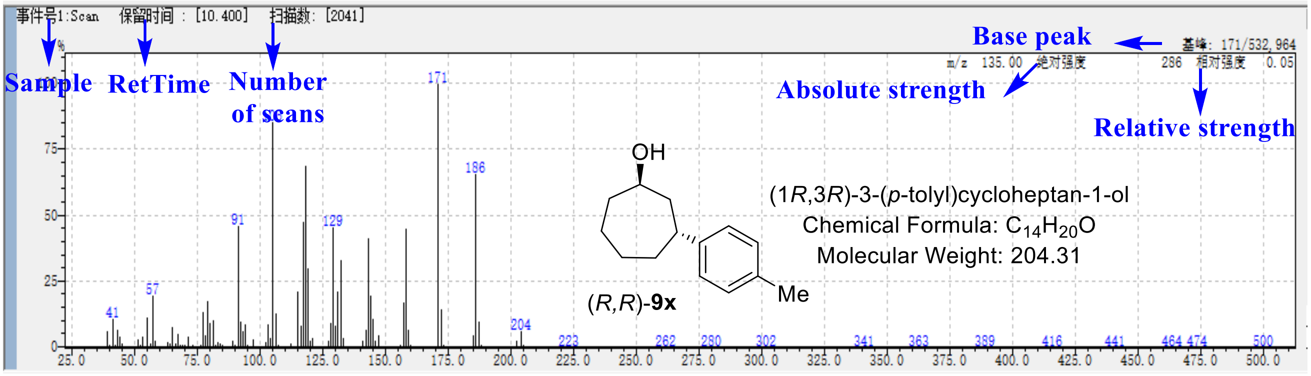

Table S3. Crystal data and structure refinement of (*R*,*R*)-**9m** (CCDC–2235252).

Identification code	(*R*,*R*)-**9m**	
Empirical formula	$C_{13}H_{18}O$	
Formula weight	190.27	
Temperature	213(2) K	
Wavelength	0.71073 Å	
Crystal system	Monoclinic	
Space group	C 2	
Unit cell dimensions	a = 22.6985(15) Å	a= 90 °.
	b = 5.2147(3) Å	b= 118.863(2) °.
	c = 22.0056(15) Å	g = 90 °.
Volume	2281.1(3) $Å^3$	
Z	8	
Density (calculated)	1.108 Mg/m^3	
Absorption coefficient	0.068 mm^{-1}	
F(000)	832	
Crystal size	0.140 x 0.110 x 0.060 mm^3	
Theta range for data collection	2.722 to 25.498 °.	
Index ranges	-25<=h<=27, -6<=k<=6, -26<=l<=26	
Reflections collected	14455	
Independent reflections	4240 [R(int) = 0.0610]	
Completeness to theta = 25.242 °	99.8 %	
Absorption correction	Semi-empirical from equivalents	
Max. and min. transmission	0.7456 and 0.5376	
Refinement method	Full-matrix least-squares on F^2	
Data / restraints / parameters	4240 / 3 / 264	
Goodness-of-fit on F^2	1.027	
Final R indices [I>2sigma(I)]	R1 = 0.0485, wR2 = 0.1044	
R indices (all data)	R1 = 0.0754, wR2 = 0.1222	
Absolute structure parameter	-0.2(10)	
Extinction coefficient	0.0081(11)	
Largest diff. peak and hole	0.136 and -0.127 e.$Å^{-3}$	

Table S4. Reusability of catalyst **5**.[a]

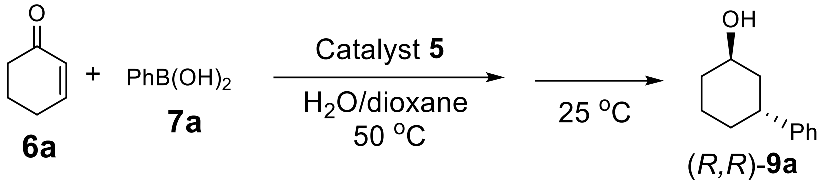

Entry	1	2	3	4	5	6	7
%Yield	77	76	75	74	72	71	63
%*ee*	96	96	96	95	96	96	95
dr	99:1	99:1	99:1	99:1	99:1	97:3	91:9

[a] Reaction conditions: The catalyst **5** (111.10 mg, 2.50 mol% of Rh-loadings and 1.84 mol% of Ru-loadings based on ICP analysis), **7a** (1.50 mmol), KOH (0.50 mmol), HCO_2Na (10.0 mmol) in 17.0 mL of H_2O/dioxane (v:v = 2:4) were added sequentially to a 10.0 mL round–bottom flask purged with nitrogen in turn. After warming to 50 ℃, a solution of **6a** (1.0 mmol) in 3.0 mL of dioxane was added dropwise to this solution and stirred at 50 ℃ for 4 h. After completion of the first transformation monitored by the thin-layer chromatography (TLC) and cooling down to 25 ℃, the mixture was allowed to react at 25 ℃ for a further 8 h. Yields were determined by ^{1}H-NMR analysis, and *ee* and *dr* values were determined by chiral HPLC analysis.

Figure S10. Reusability of catalyst **5** in the 1,4-addition/ATH cascade process of **6a** and **7a**.

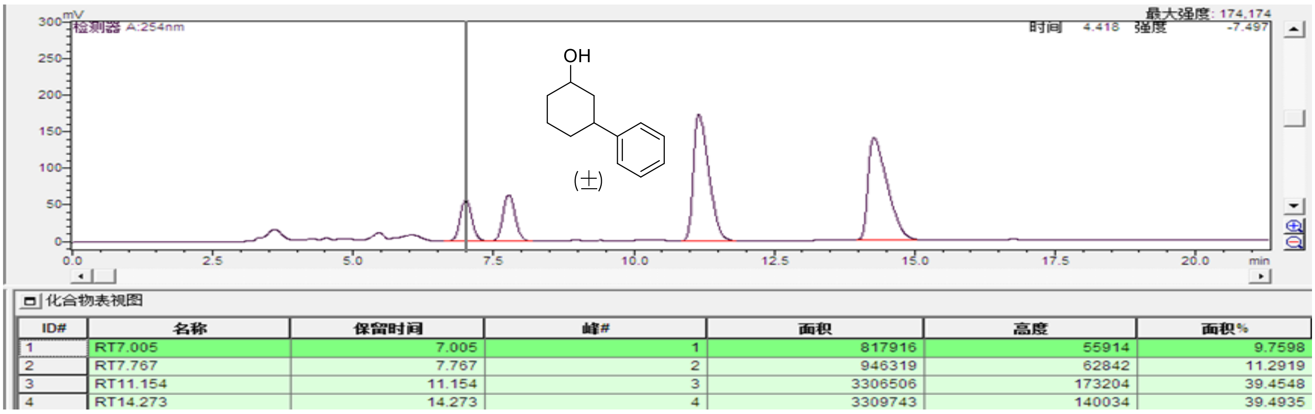

ID#	名称	保留时间	峰#	面积	高度	面积%
1	RT7.005	7.005	1	817916	55914	9.7598
2	RT7.767	7.767	2	946319	62842	11.2919
3	RT11.154	11.154	3	3306506	173204	39.4548
4	RT14.273	14.273	4	3309743	140034	39.4935

<u>**Translation of all characters (Chinese) in the above all frameworks to English is as follows:**</u>

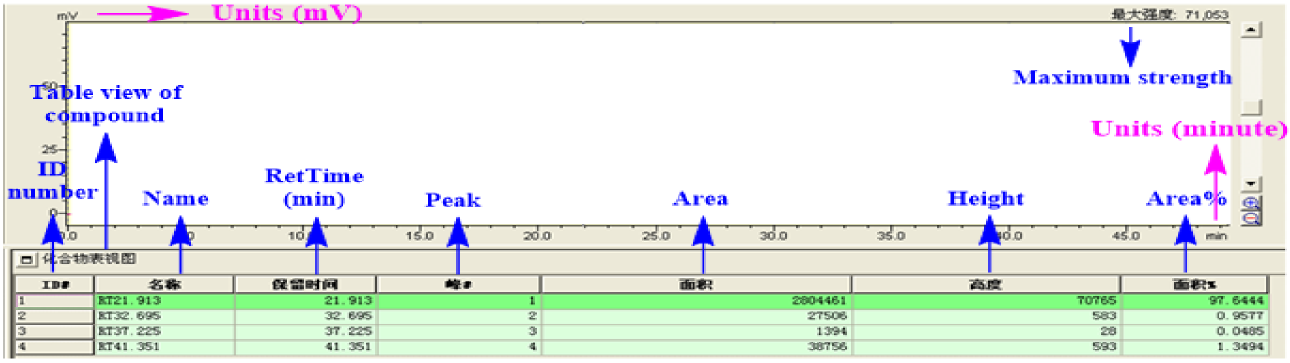

ID#	名称	保留时间	峰#	面积	高度	面积%
1	RT21.913	21.913	1	2804461	70765	97.6444
2	RT32.695	32.695	2	27506	583	0.9577
3	RT37.225	37.225	3	1394	28	0.0485
4	RT41.351	41.351	4	38756	593	1.3494

Recycle 1

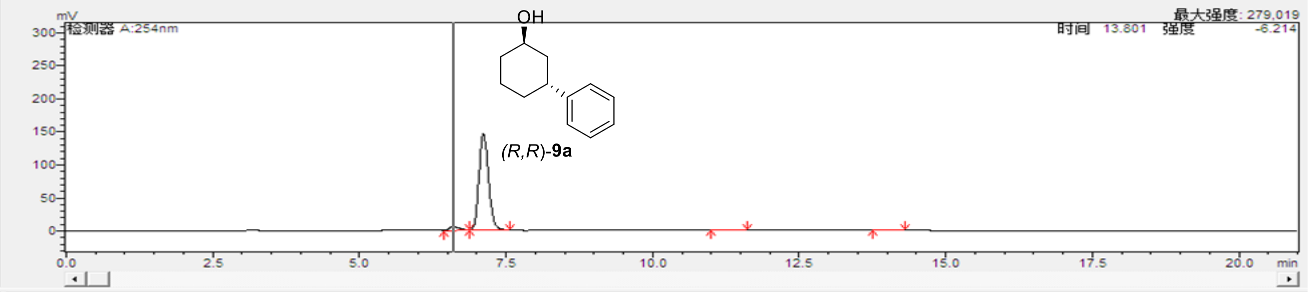

□ 化合物表视图

ID#	名称	保留时间	峰#	面积	高度	面积%
1	RT6.592	6.592	1	68489	5997	3.8817
2	RT7.110	7.110	2	1683693	146623	95.4247
3	RT11.576	11.576	3	4692	181	0.2659
4	RT14.292	14.292	4	7547	346	0.4277

Recycle 2.

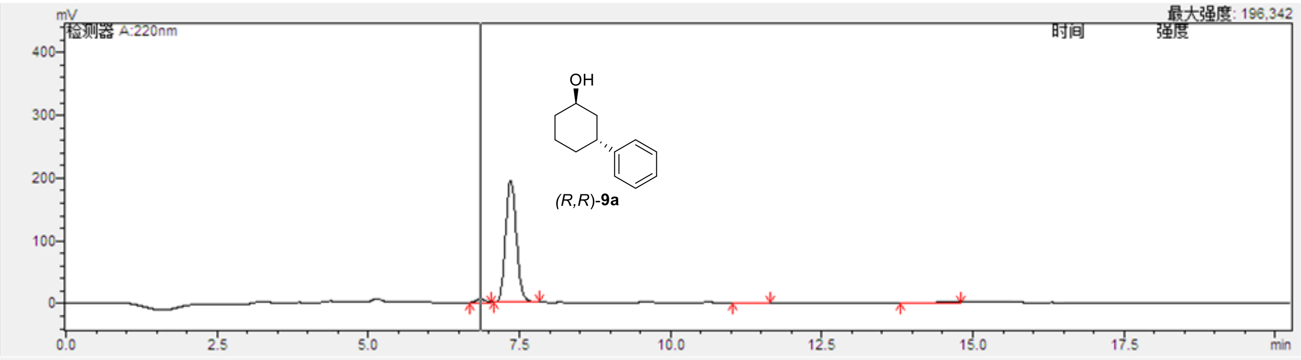

□ 化合物表视图

ID#	名称	保留时间	峰#	面积	高度	面积%
1	RT6.854	6.854	1	64467	5656	2.6045
2	RT7.353	7.353	2	2386142	193883	96.4001
3	RT11.361	11.361	3	2937	140	0.1187
4	RT14.565	14.565	4	21701	553	0.8767

<u>Translation of all characters (Chinese) in the above all frameworks to English is as follows:</u>

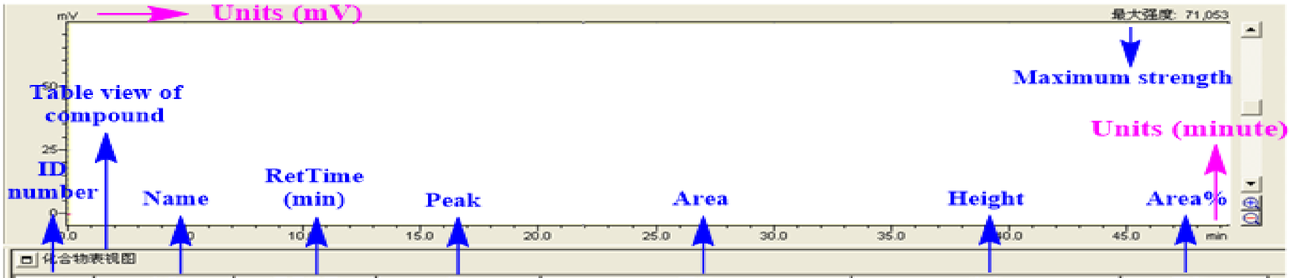

ID#	名称	保留时间	峰#	面积	高度	面积%
1	RT21.913	21.913	1	2804461	70765	97.6444
2	RT32.695	32.695	2	27506	583	0.9577
3	RT37.225	37.225	3	1394	28	0.0485
4	RT41.351	41.351	4	38756	593	1.3494

Recycle 3.

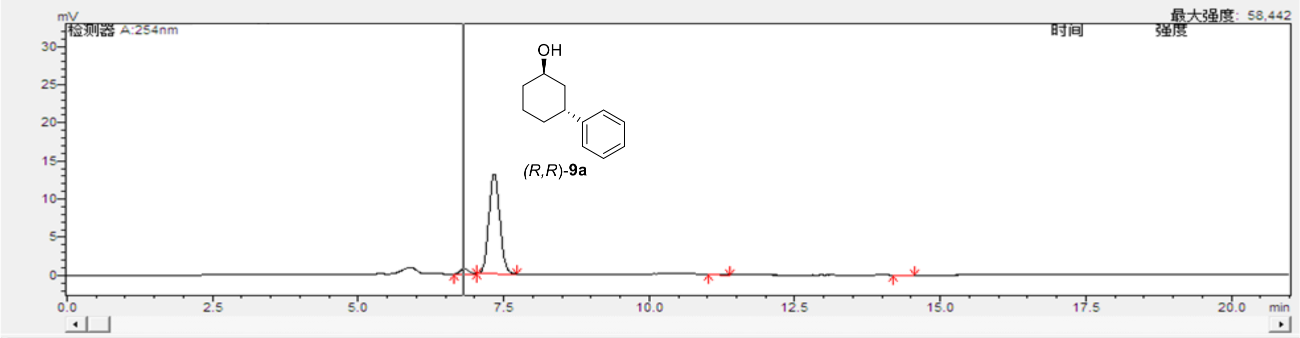

化合物表视图

ID#	名称	保留时间	峰#	面积	高度	面积%
1	RT6.818	6.818	1	8394	787	4.6318
2	RT7.334	7.334	2	170766	13061	94.2310
3	RT11.039	11.039	3	1529	79	0.8437
4	RT14.230	14.230	4	532	32	0.2935

Recycle 4.

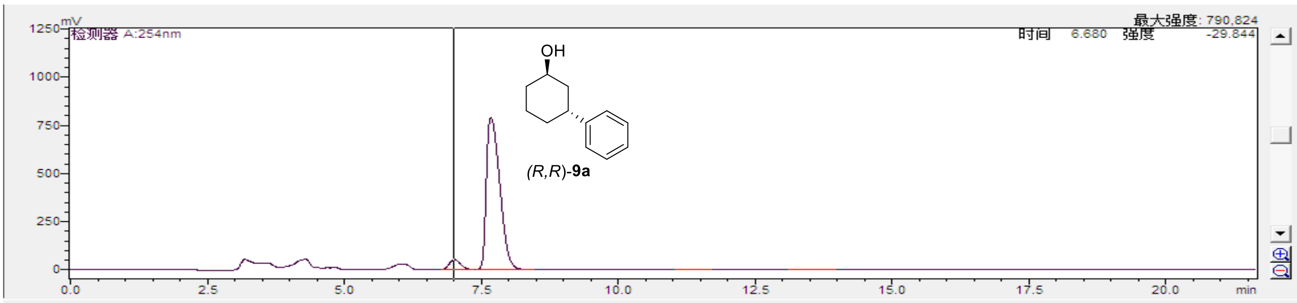

化合物表视图

ID#	名称	保留时间	峰#	面积	高度	面积%
1	RT7.008	7.008	1	738600	51927	5.2014
2	RT7.674	7.674	2	13377541	789385	94.2070
3	RT11.300	11.300	3	34749	1868	0.2447
4	RT13.448	13.448	4	49267	2856	0.3469

<u>Translation of all characters (Chinese) in the above all frameworks to English is as follows:</u>

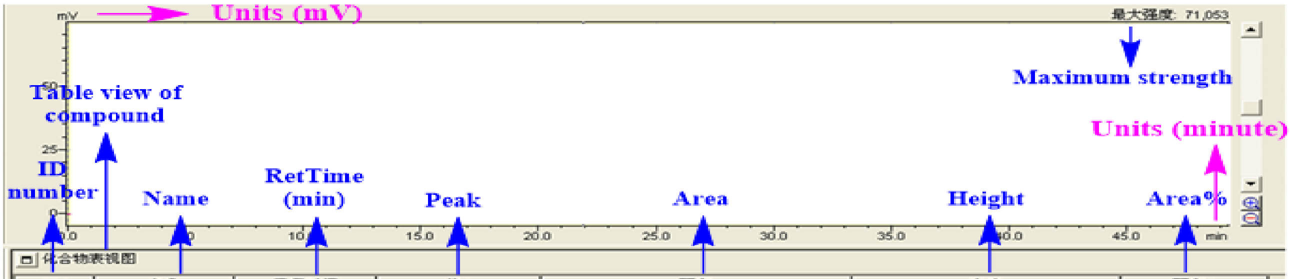

ID#	名称	保留时间	峰#	面积	高度	面积%
1	RT21.913	21.913	1	2804461	70765	97.6444
2	RT32.695	32.695	2	27506	583	0.9577
3	RT37.225	37.225	3	1394	28	0.0485
4	RT41.351	41.351	4	38756	593	1.3494

Recycle 5.

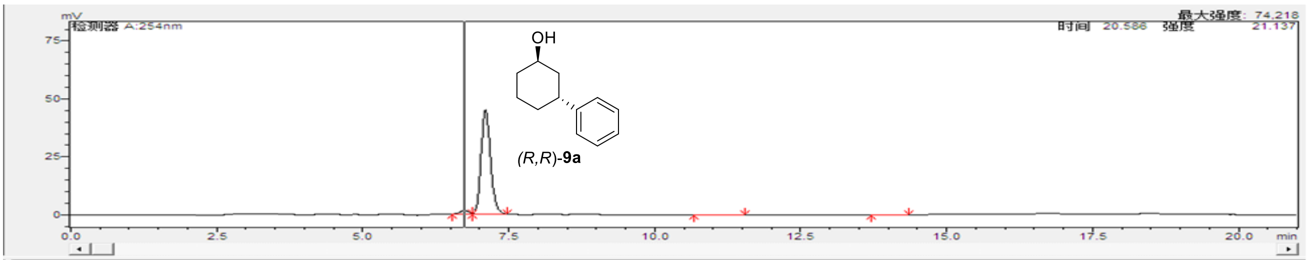

化合物表视图

ID#	名称	保留时间	峰#	面积	高度	面积%
1	RT6.738	6.738	1	16770	1597	3.1646
2	RT7.098	7.098	2	512576	45154	96.7291
3	RT11.040	11.040	3	332	22	0.0626
4	RT14.076	14.076	4	231	16	0.0436

Recycle 6

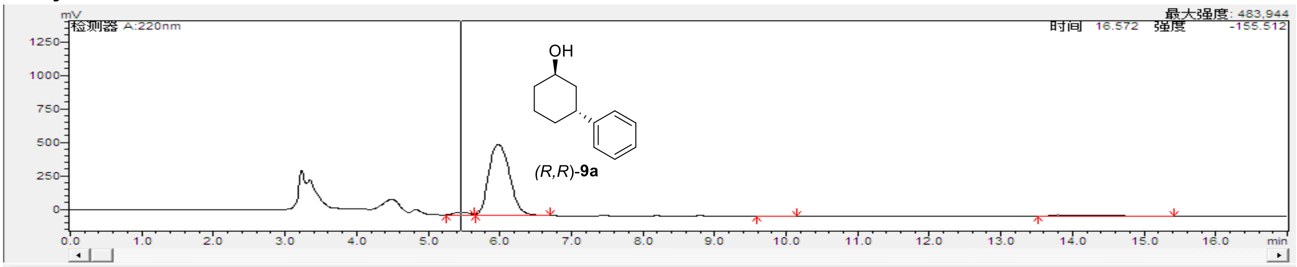

化合物表视图

ID#	名称	保留时间	峰#	面积	高度	面积%
1	RT5.455	5.455	1	296421	20957	2.6031
2	RT5.974	5.974	2	10718928	527717	94.1319
3	RT9.835	9.835	3	19000	1331	0.1669
4	RT13.790	13.790	4	352784	9018	3.0981

Recycle 7

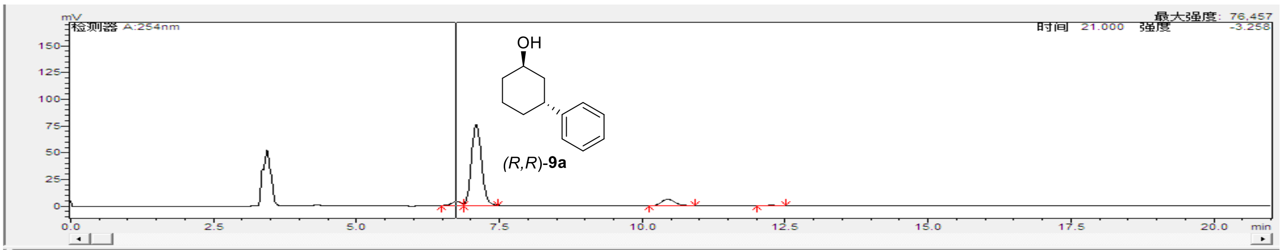

化合物表视图

ID#	名称	保留时间	峰#	面积	高度	面积%
1	RT6.747	6.747	1	48217	4024	4.3849
2	RT7.091	7.091	2	941970	75728	85.6647
3	RT10.443	10.443	3	101151	6090	9.1989
4	RT12.262	12.262	4	8264	531	0.7515

Translation of all characters (Chinese) in the above all frameworks to English is as follows:

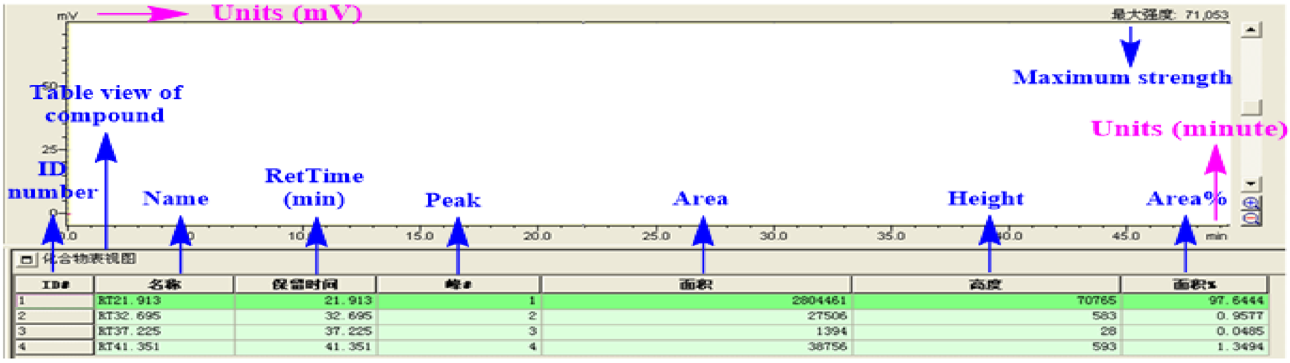

Figure S11. Contrastive ^{1}H-NMR spectra for the deuterium labeling experiments.

(a) The standard ^{1}H-NMR spectrum of (*R*)-**8a**.

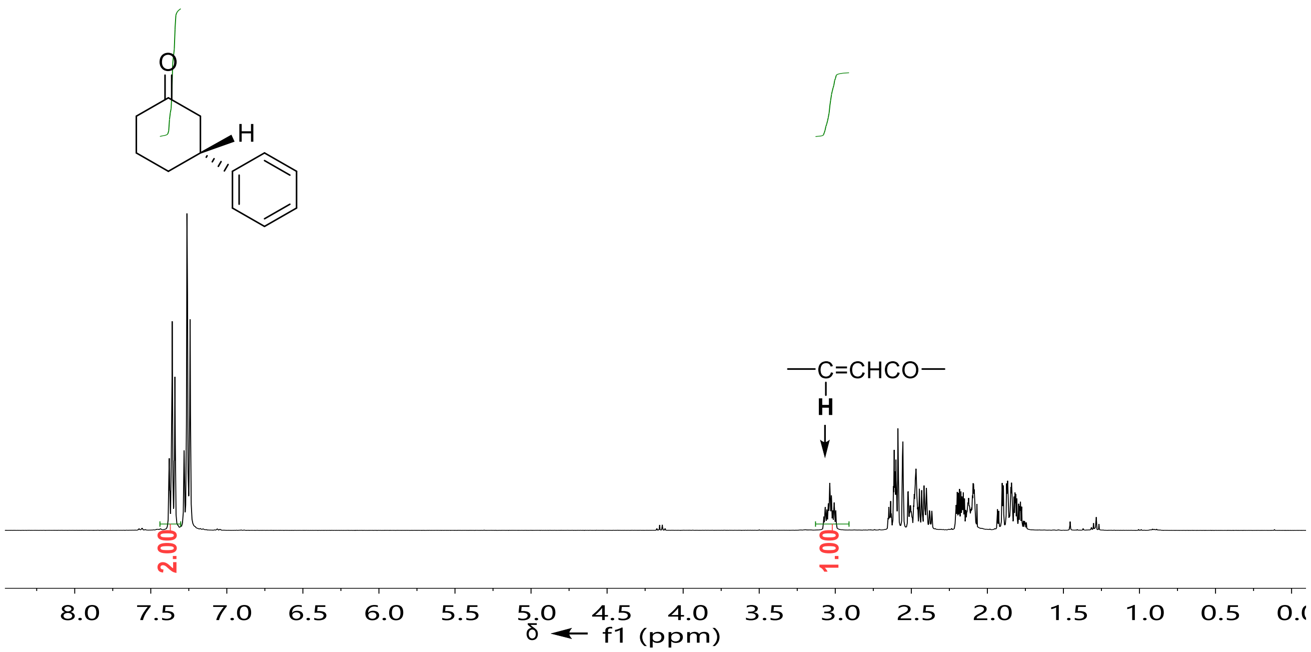

(b) The ^{1}H-NMR spectrum of (*R*)-**8a-*d*$_1$** in the **5**-catalyzed 1,4-addition reaction of **7a** and **6a**-*d$_1$* in deuterated D_2O/DMSO-d_6.

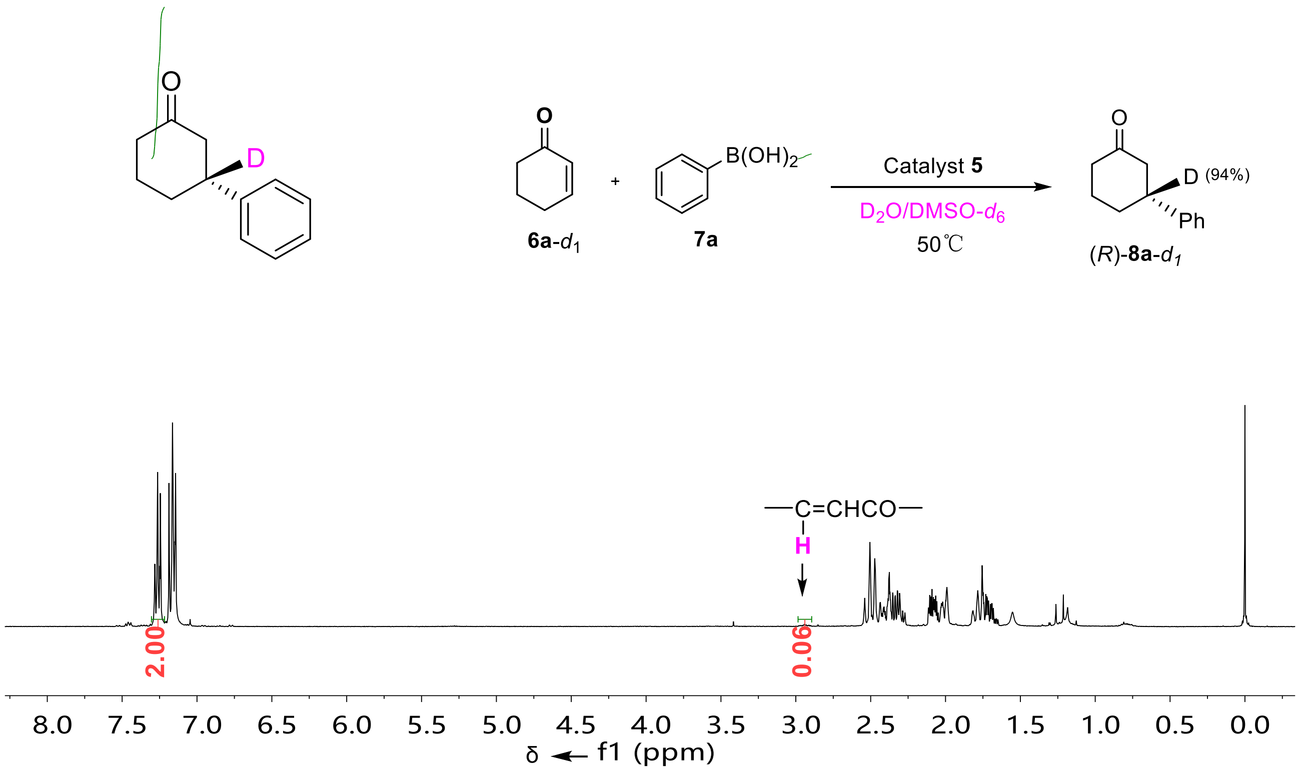

(c) The standard ^{1}H-NMR spectrum of (*R,R*)-**9a**.

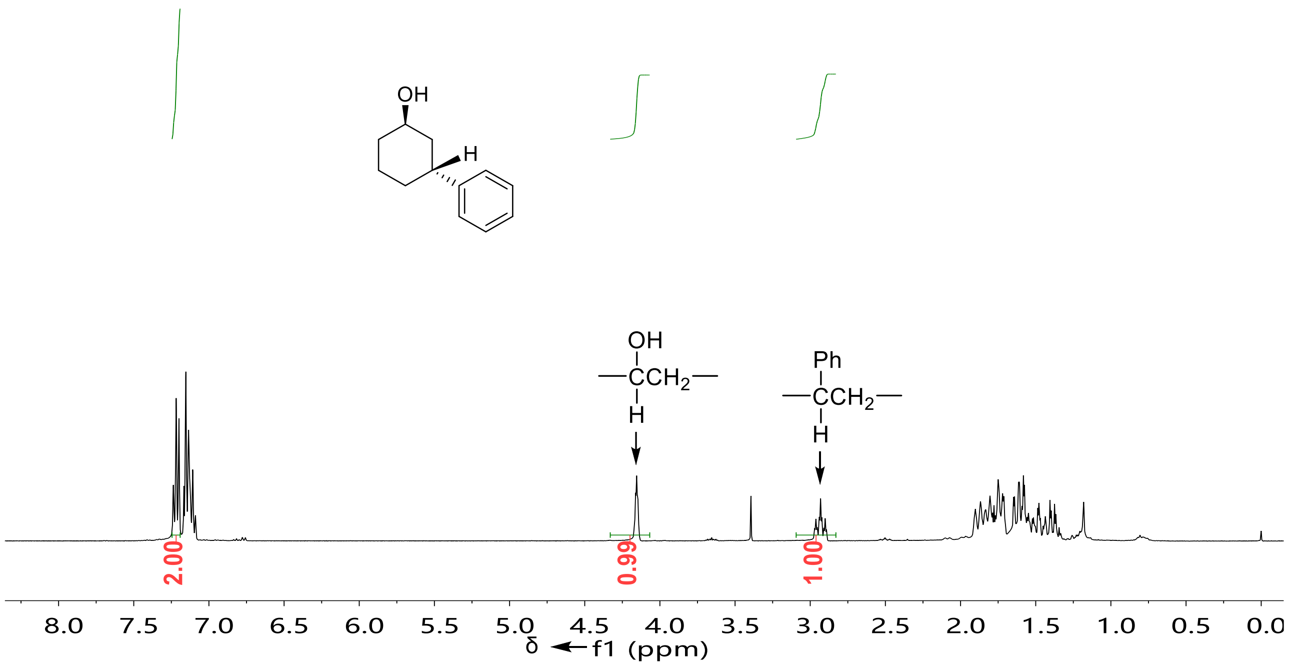

(d) The ^{1}H-NMR spectrum of (*R,R*)-**9a-*d*₃** in the **5**-catalyzed cascade reaction of **6a-*d*₁** and **7a** in the deuterated D_2O/DMSO-d_6.

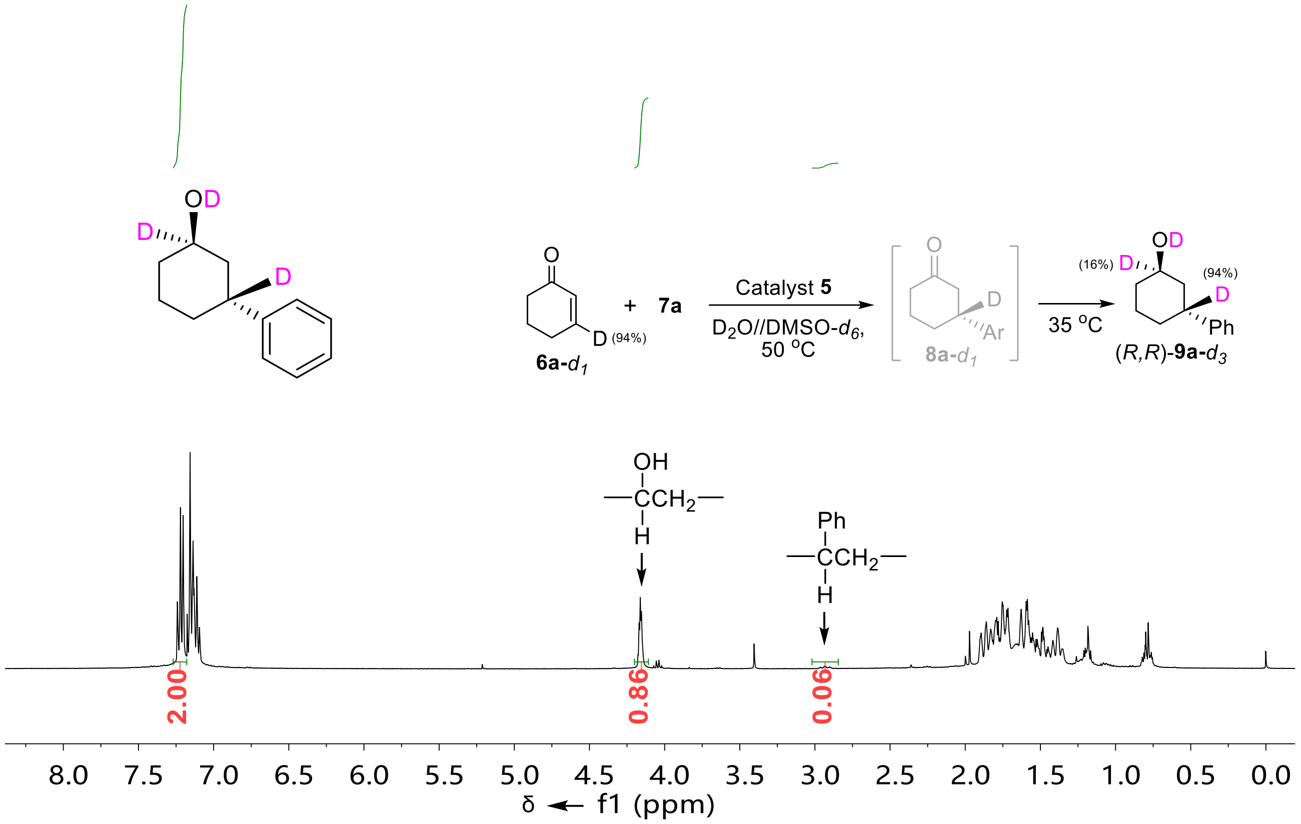

(e) The ^{1}H-NMR spectrum of (*R*,*R*)-**9a-*d*$_1$** in the **5**-catalyzed ATH reaction of (*R*)-**8a-*d*$_1$** in deuterated H_2O/DMSO**.**

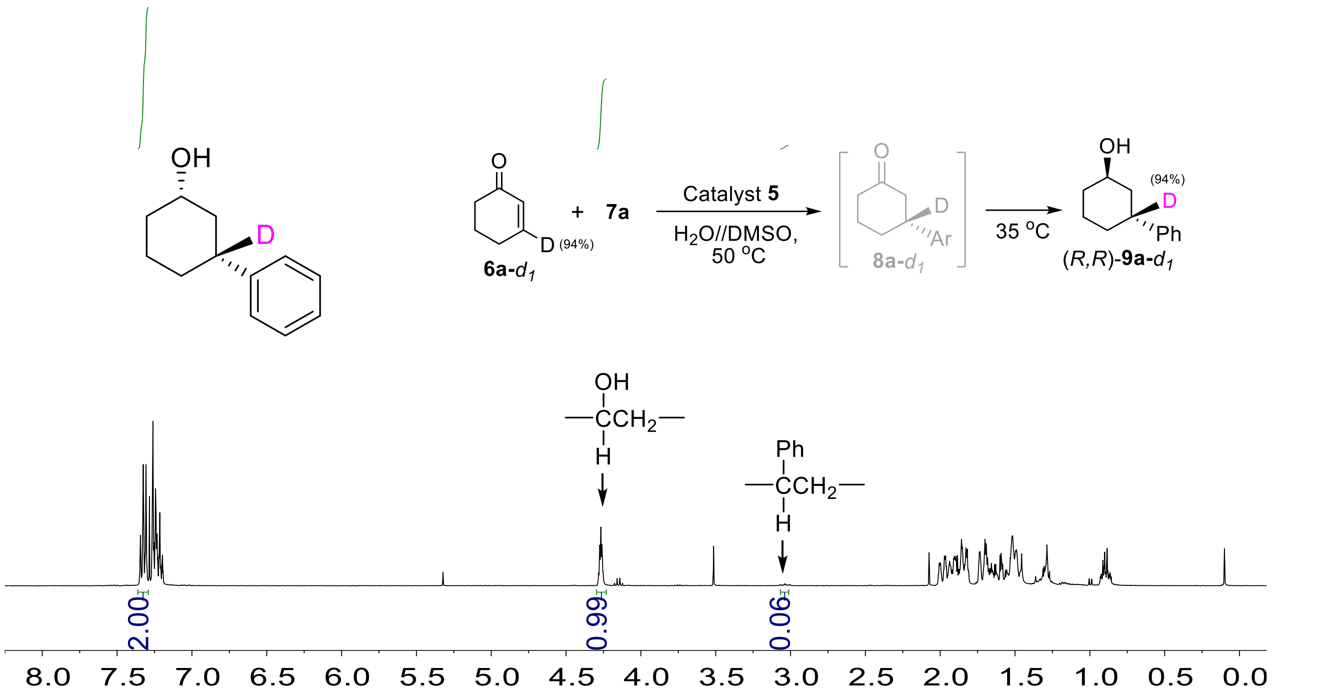

Supplement: Supplementary file 1 — Supplementary Information [file 42004_2023_1085_MOESM1_ESM.pdf]
