# Supplementary material for: Bridging the incompatibility gap in dual asymmetric catalysis over a thermoresponsive hydrogel-supported catalyst
Source: Commun Chem. 2024 Jan 3;7:2. doi: 10.1038/s42004-023-01085-z (PMC10764871; doi:10.1038/s42004-023-01085-z)
Supplement: Supplementary file 2 — Description of Additional Supplementary Files [file 42004_2023_1085_MOESM2_ESM.pdf]

Description of Additional Supplementary Files

File name: Supplementary Data 1

Description: CIF file of the crystal structure of (R,R)-9m

File name: Supplementary Data 2

Description: checks of CIF file of the crystal structure of (R,R)-9m
