# Supplementary material for: Bridging the incompatibility gap in dual asymmetric catalysis over a thermoresponsive hydrogel-supported catalyst
Source: Commun Chem. 2024 Jan 3;7:2. doi: 10.1038/s42004-023-01085-z (PMC10764871; doi:10.1038/s42004-023-01085-z)
Supplement: Supplementary file 4 — Supplementary Data 2 [file 42004_2023_1085_MOESM4_ESM.pdf]

checkCIF (basic structural check) running

Datablock: mo_d8v22555_0m for (*R*,*R*)-9m

Bond precision:	C-C = 0.0053 A		Wavelength=0.71073
Cell:	a=22.6985(15)	b=5.2147(3)	c=22.0056(15)
	alpha=90	beta=118.863(2)	gamma=90
Temperature:	213 K		

	Calculated	Reported
Volume	2281.2(3)	2281.1(3)
Space group	C 2	C 2
Hall group	C 2y	C 2y
Moiety formula	C13 H18 O	?
Sum formula	C13 H18 O	C13 H18 O
Mr	190.27	190.27
Dx,g cm-3	1.108	1.108
Z	8	8
Mu (mm-1)	0.068	0.068
F000	832.0	832.0
F000'	832.33	
h,k,lmax	27,6,26	27,6,26
Nref	4262[2383]	4240
Tmin,Tmax	0.991,0.996	0.538,0.746
Tmin'	0.991	

Correction method= # Reported T Limits: Tmin=0.538 Tmax=0.746 AbsCorr = MULTI-SCAN

Data completeness= 1.78/0.99 Theta(max)= 25.498

R(reflections)= 0.0485(3128) wR2(reflections)= 0.1222(4240)

S = 1.027 Npar= 264

The following ALERTS were generated. Each ALERT has the format

test-name_ALERT_alert-type_alert-level.

Click on the hyperlinks for more details of the test.

Alert level C

STRVA01_ALERT_4_C Flack test results are meaningless.

From the CIF: _refine_ls_abs_structure_Flack -0.200

From the CIF: _refine_ls_abs_structure_Flack_su 1.000

PLAT220_ALERT_2_C NonSolvent Resd 2 C Ueq(max)/Ueq(min) Range 3.2 Ratio

PLAT222_ALERT_3_C NonSolvent Resd 1 H Uiso(max)/Uiso(min) Range 4.7 Ratio

PLAT222_ALERT_3_C NonSolvent Resd 2 H Uiso(max)/Uiso(min) Range 5.4 Ratio

PLAT241_ALERT_2_C High 'MainMol' Ueq as Compared to Neighbors of C9 Check

PLAT242_ALERT_2_C Low 'MainMol' Ueq as Compared to Neighbors of C7 Check

PLAT242_ALERT_2_C Low 'MainMol' Ueq as Compared to Neighbors of C10 Check

PLAT334_ALERT_2_C Small <C-C> Benzene Dist. C7 -C12 . 1.37 Ang.

PLAT340_ALERT_3_C Low Bond Precision on C-C Bonds .... 0.00532 Ang.

PLAT910_ALERT_3_C Missing # of FCF Reflection(s) Below Theta(Min). 5 Note

---

Alert level G

PLAT002_ALERT_2_G Number of Distance or Angle Restraints on AtSite 4 Note

PLAT032_ALERT_4_G Std. Uncertainty on Flack Parameter Value High . 1.000 Report

PLAT172_ALERT_4_G The CIF-Embedded .res File Contains DFIX Records 1 Report

PLAT791_ALERT_4_G Model has Chirality at C4 (Sohnke SpGr) R Verify

And 3 other PLAT791 Alerts

More ...

PLAT860_ALERT_3_G Number of Least-Squares Restraints .... 3 Note

PLAT883_ALERT_1_G No Info/Value for _atom_sites_solution_primary . Please Do !

PLAT916_ALERT_2_G Hooft y and Flack x Parameter Values Differ by . 0.20 Check

PLAT933_ALERT_2_G Number of HKL-OMIT Records in Embedded .res File 4 Note

PLAT965_ALERT_2_G The SHELXL WEIGHT Optimisation has not Converged Please Check

PLAT967_ALERT_5_G Note: Two-Theta Cutoff Value in Embedded .res .. 51.0 Degree

PLAT978_ALERT_2_G Number C-C Bonds with Positive Residual Density. 0 Info

---

0 **ALERT level A** = Most likely a serious problem - resolve or explain

0 **ALERT level B** = A potentially serious problem, consider carefully

10 **ALERT level C** = Check. Ensure it is not caused by an omission or oversight

14 **ALERT level G** = General information/check it is not something unexpected

1 ALERT type 1 CIF construction/syntax error, inconsistent or missing data

10 ALERT type 2 Indicator that the structure model may be wrong or deficient

5 ALERT type 3 Indicator that the structure quality may be low

7 ALERT type 4 Improvement, methodology, query or suggestion

1 ALERT type 5 Informative message, check
